# Supplementary material for: Regulating the UAS/GAL4 system in adult Drosophila with Tet-off GAL80 transgenes
Source: PeerJ. 2017 Dec 14;5:e4167. doi: 10.7717/peerj.4167 (PMC5733373; doi:10.7717/peerj.4167)
Supplement: Supplemental Information 1 — Supplemental text, figures and tables (datasets and statistics). [file peerj-05-4167-s001.pdf]

24B expression pattern during aging 3
Methods 3
Results 3
pDJ146 construction 4
pDJ136 subcloning 4
pDJ138 subcloning 4
pDJ140 subcloning 5
pDJ143 subcloning 5
pDJ146 subcloning 5
pDJ147 construction 6
pDJ141 subcloning 6
pDJ144 subcloning 6
pDJ159 subcloning 6
pDJ160 subcloning 6
pDJ147 subcloning 7
Quantification of tTA pre-adult expression 7
Methods 7
Results 7
Midlife Induction 8
Methods 8
Results 8
Kinetics of Reestablishment 9
Methods 9
Results 10
References 11
Supplemental figures 12
Supplemental Figure 1: 24B GAL4-driven UAS-lacZ expression pattern during aging at 25°C. 13
Supplemental Figure 2: Construction of pDJ136. 14
Supplemental Figure 3: Construction of pDJ138. 15
Supplemental Figure 4: Construction of pDJ139. 16
Supplemental Figure 5: Construction of pDJ140. 17
Supplemental Figure 6: Construction of pDJ143. 18
Supplemental Figure 7: Construction of pDJ146. 19
Supplemental Figure 8: Construction of pDJ141. 20
Supplemental Figure 9: Construction of pDJ144. 21
Supplemental Figure 10: Construction of pDJ159. 22
Supplemental Figure 11: Construction of pDJ160. 23
Supplemental Figure 12: Construction of pDJ147. 24
Supplemental Figure 13: Grim lethality assay of 1 copy DJ146 insertion treated with ampicillin. 25
Supplemental Figure 14: Grim lethality assay of 1 copy DJ146 insertion treated with ampicillin and tetracycline. 26
Supplemental Figure 15: Grim lethality assay of 1 copy DJ147 insertion treated with ampicillin. 27
Supplemental Figure 16: Grim lethality assay of 1 copy DJ147 insertion treated with ampicillin and tetracycline. 28
Supplemental Figure 17: Grim lethality assay of 2 copies of DJ146 insertion treated with ampicillin. 29

Supplemental Figure 18: Grim lethality assay of 2 copies of DJ146 insertion treated with ampicillin and tetracycline. 30
Supplemental Figure 19: Grim lethality assay of 2 copies of DJ147 insertion treated with ampicillin. 31
Supplemental Figure 20: Grim lethality assay of 2 copies of DJ147 insertion treated with ampicillin and tetracycline. 32
Supplemental Figure 21: Grim lethality assay of 1 copy of DJ147 insertion and 1 copy of DJ146 insertion treated with ampicillin. 33
Supplemental Figure 22: Grim lethality assay of 1 copy of DJ147 insertion and 1 copy of DJ146 insertion treated with ampicillin and tetracycline. 34
Supplemental Figure 23: Grim lethality assay of 3 copies of DJ146 insertion treated with ampicillin. 35
Supplemental Figure 24: Grim lethality assay of 3 copies of DJ146 insertion treated with ampicillin and tetracycline. 36
Supplemental Figure 25: Grim lethality assay of 1 copy of DJ147 insertion and 2 copies of DJ146 insertion treated with ampicillin. 37
Supplemental Figure 26: Grim lethality assay of 1 copy of DJ147 insertion and 2 copies of DJ146 insertion treated with ampicillin and tetracycline. 38
Supplemental Figure 27: Grim lethality assay of 4 copies of DJ146 insertion treated with ampicillin. 39
Supplemental Figure 28: Grim lethality assay of 3 copies of DJ147 insertion treated with ampicillin and tetracycline. 40
Supplemental Figure 29: Grim lethality assay of 2 copies of DJ147 insertion and 2 copies of DJ146 insertion treated with ampicillin. 41
Supplemental Figure 30: Grim lethality assay of 2 copies of DJ147 insertion and 2 copies of DJ146 insertion treated with ampicillin and tetracycline. 42
Supplemental Figure 31: Relief of repression of GAL4 transcriptional activity by GAL80, as measured by grim lethality assay in the presence of inducer. 43
Supplemental Figure 32: DJ146 and DJ147 tTA transcriptional activity during development measured using a tetO-lacZ reporter. 44
Supplemental Figure 33: GAL4 transcriptional activity in 2x146 + 2x147 flies that were fed tetracycline starting at 20 days of age. 45
Supplemental Figure 34: Tissue distribution of lacZ expression in 30 days old flies fed 0 or 100μg/ ml of tetracycline beginning at 20 days of age. 46
Supplemental Figure 35: Kinetics of reestablishing repression of GAL4 transcriptional activity. 47

Supplemental tables 48

Supplemental Table 1: UAS-grim lethality assay data set. 48
Supplemental Table 2: UAS-grim lethality assay statistical analysis (t-tests). 59
Supplemental Table 3: UAS-grim lethality assay statistical analysis (t-tests). 78
Supplemental Table 4: UAS-lacZ assay L1 data set. 80
Supplemental Table 5: UAS-lacZ assay L3 data set. 97
Supplemental Table 6: UAS-lacZ assay EP data set. 115
Supplemental Table 7: UAS-lacZ assay LP data set. 133
Supplemental Table 8: UAS-lacZ assay statistical analysis (t-tests). 151
Supplemental Table 9: Repression ability of 2x146 + 2x147 in adults fed through life. 154
Supplemental Table 10: Induction abilities of 2x146 + 2x147 in adults fed through life. 155
Supplemental Table 11: Repression ability of 2x146 + 2x147 in adults fed starting at 20 days old. 156
Supplemental Table 12: Induction abilities of 2x146 + 2x147 in adults fed starting at 20 days old. 157
Supplemental Table 13: GAL4 driver survey data set. 158

Reagents table 176

24B expression pattern during aging

Methods

Experimental animals were obtained from a cross between the w[*]; P{w[+mC]=UAS-lacZ.B}Bg4-2-4b (Bloomington stock 1777) and the w[*]; P{w[+mW.hs]=GawB}how[24B] (Bloommington stock 1767) strains. Negative control animals were obtained from a cross between the w[*]; P{w[+mC]=UAS-lacZ.B}Bg4-2-4b and a w[*] strain. Age-synchronized male and female cohorts (25-30 individuals per vial) were collected from the resulting progenies under nitrogen anesthesia. Cohorts were kept at 25˚C and transferred to vials with fresh food at least twice a week. CPRG and X-gal assays were performed as described in the main text.

Results

A quantitative analysis of 24B GAL4 expression during aging was performed by measuring ß-galactosidase activity with the CPRG assay as a function of age (Supplemental Figure 1A). The results show that the expression is the highest during the first 24h after emergence before dropping by half at 3 days and remaining constant afterward. The profile of expression is extremely similar between genders. The same analysis yielded enzyme activities mostly between 2000 and 7500 ΔmOD/min/fly for the DJ694 driver (Seroude *et al.* 2002) and between 500 and 1600 ΔmOD/min/fly for the MHC driver in absence of RU486 inducer (Poirier *et al.* 2008). With an activity mostly around 2000 ΔmOD/min/fly, the 24B driver is expressing the second highest level of GAL4 in aging flies.

X-gal-stained cryo-sections were generated to determine the localization of 24B GAL4 expression in adults as a function of age (Supplemental Figure 1B). No staining could be detected in the negative control animals (data not shown). In freshly eclosed males, GAL4 is expressed in all muscle tissues but a lower level of expression is evident in the flight muscles. GAL4 is also expressed in the salivary glands and in the abdominal fat but no expression is visible in the nervous and digestive systems. At all subsequent ages examined, the

expression in muscle tissues has decreased and is almost absent in the flight muscles except for a subset of the vertical indirect wing muscles. The loss of expression in the flight muscles is therefore mainly responsible for the drop in expression observed with the CPRG assay. It is worth noting that GAL4 expression is also visible in the testis and most oenocytes although the expression in the later is almost absent in the oldest animals tested.

pDJ146 construction

pDJ136 subcloning

The pTub-GAL80 plasmid (Lee and Luo 1999) was used as a PCR template with the DJ111 (5'-GAAAAGATACTTGAGGTTCG-3') and DJ112 (5'-GTCAGAATTCTCTAGAGCAACATGGACTACAACAAG-3') primers to obtain a 365bp fragment containing the 5' end of the GAL80 coding region as well as introducing *EcoRI* and *XbaI* restriction sites upstream of the start codon (Supplemental Figure 2). After *EcoRI* digestion, the PCR fragment was ligated to the 9445bp *EcoRI* fragment from pTub-GAL80 to obtain the 9777bp pDJ136 plasmid. The orientation of the insert and the structure of the plasmid were determined by *XbaI* digestion. The sequences amplified during the PCR were sequenced with DJ111 and DJ112. This strategy removed the *tubulin 1α* promoter sequences from the pTub-GAL80 and allows the excision of the complete GAL80 coding region with *XbaI*.

pDJ138 subcloning

The GAL80 coding region from pDJ136 was inserted into the pJY2000 (Stebbins and Yin 2001) using *XbaI* to create the 12853bp pDJ138 plasmid (Supplemental Figure 3). The orientation of the insert and the structure of the plasmid were determined by *BglII* digestion. This plasmid has the GAL80 coding region with ß-globin poly A signals under the control of a *TetO* promoter surrounded by the scs and scs' chromatin boundaries elements.

pDJ139 subcloning

A linker containing multiple restriction sites was generated from blunting the product obtained by hybridizing the DJ117 (5'-AGTCTAGATAAGATCTGAGCTCTCCGCGGATGCGGCCGCTAGAA-3') and DJ118 (5'-ATATGCATCTCGAGGAATTCTAGCGGCCGCATCCGCGGAGAG-3') oligonucleotides (complementary sequences greyed)(Supplemental Figure 4). *XbaI* an *NsiI* were used to remove the GAL80 coding region and the *mini-white* gene from the pDJ136 and insert the multiple restriction sites to create the 4485bp pDJ139 plasmid. The presence of the insert and the structure of the plasmid were determined by *XhoI* digestion. The functionality of each site introduced with the linker was confirmed by restriction digest.

pDJ140 subcloning

The *tubulin 1α* promoter from the pTub-GAL80 (Lee and Luo 1999) was subcloned into the pDJ139 with *EcoRI* and *NotI* to obtain the 7104bp pDJ140 plasmid (Supplemental Figure 5). The presence of the insert and the structure of the plasmid were confirmed by *SacII* and *EcoRI/NotI* digestions. pDJ140 increases the diversity of restriction sites available for cloning between the *tubulin 1α* promoter and the SV40 poly A region.

pDJ143 subcloning

The BamHI fragment from the pMS12 (Stebbins and Yin 2001) containing the tetracycline-responsive transactivator (tTA) was ligated to the *BglII* site of the pDJ140 to generate the 8384bp pDJ143 plasmid (Supplemental Figure 6). The orientation of the insert and the structure of the plasmid were determined by *XbaI* digestion.

pDJ146 subcloning

The *tubulin 1α* promoter–tTA-SV40 section of the pDJ143 was inserted between the *TetO* promoter and the scs' insulator element of the pDJ138 using *XhoI* (Supplemental Figure 7). The orientation of the insert and the structure of the resulting 17506bp pDJ146 plasmid were confirmed by *XhoI* and *XbaI* digestions.

pDJ147 construction

pDJ141 subcloning

The *actin5C* promoter from the pMS12 (Stebbins and Yin 2001) was subcloned into the pDJ139 with *SacI* and *NotI* to obtain the 7000bp pDJ141 plasmid (Supplemental Figure 8). The presence of the insert and the structure of the plasmid were confirmed by *AatII* and *NotI/SacI* digestions. pDJ141 increases the diversity of restriction sites available for cloning between the *actin5C* promoter and the SV40 poly A region.

pDJ144 subcloning

The BamHI fragment from the pMS12 (Stebbins and Yin 2001) containing the tetracycline-responsive transactivator (tTA) was ligated to the *BglII* site of the pDJ141 to generate the 8280bp pDJ144 plasmid (Supplemental Figure 9). The orientation of the insert and the structure of the plasmid were determined by *XbaI* digestion.

pDJ159 subcloning

Despite many attempts, we could not get the *actin5C* promoter-tTA-SV40 *XhoI* fragment from the pDJ144 inserted in the pDJ138. We therefore relocated the *XhoI* in pDJ138 between the ß-globin poly A signals and the scs insulator element. The *XhoI* site was removed from the pDJ138 by digestion with XhoI, filling with T4 polymerase and circularization with DNA ligase to obtain the 12857bp pDJ158. An adapter generated by the annealing of the DJ188 (5'-GGCCTCGAGTGCAGC-3') and DJ189 (5'-GGCCGCTGCACTCGA-3') oligonucleotides (complementary sequences greyed) was ligated to the *NotI* site of the pDJ158 to create the 12872bp pDJ159 plasmid (Supplemental Figure 10).

pDJ160 subcloning

We still could not get the *actin5C* promoter-tTA-SV40 *XhoI* fragment from the pDJ144 inserted in the pDJ159. We therefore modified the pDJ144 to allow the excision of the *actin5C* promoter-tTA-SV40 cassette with *NotI* (Supplemental Figure 11). An adapter generated by the annealing of the DJ192 (5'-

GCGGCCGCATGCA-3’) and DJ193 (5’-TGCGGCCGCTGCA-3’) oligonucleotides (complementary sequences greyed) was ligated to the *PstI* site of the pDJ144 to create the 8293bp pDJ160 plasmid.

pDJ147 subcloning

The *actin5C 1α* promoter–tTA-SV40 section of the pDJ160 was inserted between the ß-globin poly A signals and the scs insulator element of the pDJ159 using *NotI* (Supplemental Figure 12). The orientation of the insert and the structure of the resulting 17425bp pDJ147 plasmid were confirmed by *XhoI* and *SpeI* digestions.

Quantification of tTA pre-adult expression

Methods

Experimental animals were obtained by crossing tetO-lacZ females by UAS-grim, UAS-grim; DJ1077, UAS-grim; 2.1, and UAS-grim; 2.1, DJ1077 males. Wandering L3 progeny were identified and selected for L3 samples. Early pupae were identified (white) and the following day selected (yellow). Late pupae were identified and selected when the wings and eyes were fully developped. All samples were processed with the CPRG assay as described in the Materials and Methods.

Results

To investigate why an additional copy of DJ147 rather than another copy of DJ146 leads to better repression, males carrying 1 copy of DJ146, 1 copy of DJ147 or 1 copy of both DJ146 and DJ147 were crossed to females carrying a tetO-lacZ reporter gene (Supplemental Figure 32). The level of *lacZ* activity was measured at the L3 larval, early pupal and late pupal stages using the CPRG assay. The DJ146 transgene displayed higher *lacZ* activity than the DJ147 transgene at all stages tested. The combination of 1 copy of DJ146 with 1 copy of DJ147 did not result in a significant increase of lacZ activity except in late pupae.

Midlife Induction

Methods

DJ694 and w^{1118} males were crossed with UAS-lacZ females while DJ694; 3.3 + DJ1077 males were crossed with UAS-lacZ; 3.3 + DJ1077 females. Age-synchronized cohorts of the resulting adult progeny were obtained by emptying cultures and collecting newly emerged flies within 48 h. Approximately 350 males and 350 females from each cross were collected under nitrogen anesthesia. Males and females were maintained separately at 25°C at a density of 25-30 flies per vial and raised on standard fly food until 20 days of age. At 20 days, each gender was randomly subdivided in 3 cohorts, each of which was provided food containing a different concentration of tetracycline (0, 1, or 100μg/ml). Fresh food was provided by transferring to new vials twice a week. Individuals were randomly removed from each cohort at 22, 25, 30 and 40 days of age and were processed for the CPRG assay. For the X-gal staining, flies were removed and processed at 30 days of age.

Results

Individuals carrying the UAS-lacZ reporter alone or in combination with the DJ694 driver served as negative and positive controls, respectively. The level of *lacZ* expression was measured by CPRG assay at 2, 5, 10 and 20 days after induction (22, 25, 30, 40 days of age). At each age, all inducer concentrations (0, 1, and 100μg/ml) for each experimental and control genotype were measured simultaneously (Supplemental Figure 33). Comparisons between experimental and control animals were performed to determine the repressive ability of GAL80 (Supplemental Table 11). In both sexes the presence of the GAL80 transgenes significantly reduced *lacZ* expression but never dropped it to the level of the negative control. The level of repression in males started to decrease at the same age and comparable magnitude as observed in the throughout-life induction experiments. Similarly, females displayed less repression than males.

Next the inducibility was examined. The different inducer concentrations were compared to determine the number of inducing concentrations and how many different levels of expression were achieved over the ages tested (Supplemental Table 12). As seen in the induction throughout life assay, two days of treatment with either inducer concentration was insufficient to induce *lacZ* expression in either sex. 5 days after induction both concentrations induced *lacZ* expression and resulted in a single level of expression in males. While in females there was no induction until 10 days of treatment and only with the highest concentration.
After 10 days of treatment (30 days of age), flies were sectioned and stained with X-gal (Supplemental Figure 34). The staining reactions were shorter than in the throughout-life induction experiments because of the higher GAL4 expression at this age with the DJ694 driver. In the absence of GAL4, no expression is detected in any tissue of either sex. In animals carrying the GAL80 transgenes, *lacZ* expression is strongly reduced compared to the positive control animals (DJ694) but some expression remains in the thoracic flight muscles and the head, and it is higher in the females than the males. Animals fed tetracycline show induction of *lacZ* expression in the abdominal, labial and leg muscles. Induction is clearly visible in the thoracic muscles of the males but not in the females.

Kinetics of Reestablishment

Methods

DJ694 and w^{1118} males were crossed with UAS-lacZ females while DJ694; 3.3 + DJ1077 males were crossed with UAS-lacZ; 3.3 + DJ1077 females. Age-synchronized cohorts of the resulting adult progeny were obtained by emptying cultures and collecting newly emerged flies within 48 h. Flies were treated with tetracycline (0, 1, or 100μg/ml) for the first five days of life. At five days of age the flies on the tetracycline food were either maintained on the treated food (positive controls) or placed on food containing no tetracycline (experimental genotypes).

Flies raised on food containing no tetracycline since adult emergence served as negative controls. The lacZ activity was quantified in all flies after 5 days (10 days of age), 9 days (14 days old), and 20 days (25 days old) with the CPRG assay as described in the Materials and Methods. The activities of the animals carrying GAL80 transgenes were normalized to the level of age matched animals carrying the driver and UAS-lacZ transgenes (no GAL80) with the same tetracycline treatment (Supplemental Figure 35). Statistical t-test analysis was performed to compare the experimental genotypes to the positive and negative controls.

Results

In males, 5 days placement of the 1μg/ml flies onto food with no tetracycline resulted in a significant decrease in lacZ activity compared to the positive control (1μg/ml 10 day old flies), but was not sufficient to reduce the level of lacZ activity to the levels seen in the negative control (0μg/ml 10 day old flies). No difference in lacZ activity was detected between flies maintained on the 100μg/ml tetracycline treatment for the full 10 days and those switched to food containing no tetracycline at day 5. After 9 days on food with no tetracycline in both the 1μg/ml and 100μg/ml experimental males there was a significant decrease in the lacZ activity compared to the positive control, but was insufficient in reducing the level of lacZ activity to the levels seen in the negative control. However, after 20 days on food containing no tetracycline, the level of lacZ activity in male flies treated with 1μg/ml of tetracycline for the first five days of life was significantly lower than the positive control and indistinguishable from the negative control . In contrast the 100μg/ml males showed a significant decrease in lacZ activity compared to the positive control, but had not reduced the level of lacZ activity to the levels seen in the negative control. In females, at no age was the lacZ activity significantly reduced from the level of that of the positive control.

References

Lee, T., and L. Luo, 1999 Mosaic analysis with a repressible cell marker for studies of gene function in neuronal morphogenesis. Neuron 22**:** 451-461.

Poirier, L., A. Shane, J. Zheng and L. Seroude, 2008 Characterization of the Drosophila gene-switch system in aging studies: a cautionary tale. Aging Cell 7**:** 758-770.

Seroude, L., T. Brummel, P. Kapahi and S. Benzer, 2002 Spatio-temporal analysis of gene expression during aging in *Drosophila melanogaster*. Aging Cell 1**:** 47-56.

Stebbins, M. J., and J. C. P. Yin, 2001 Adaptable doxycycline-regulated gene expression systems for Drosophila. Gene 270**:** 103-111.

Supplemental figures

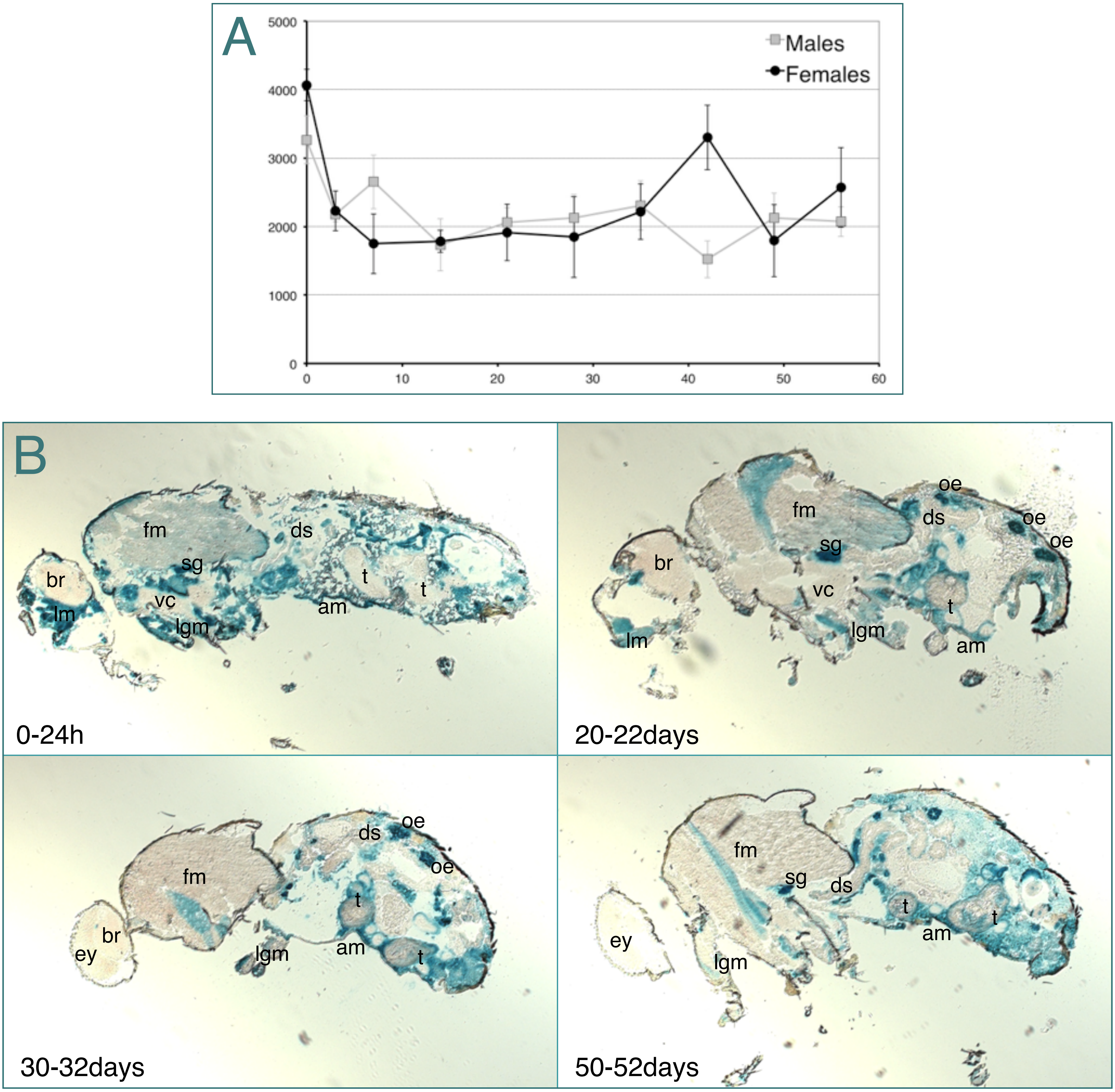

Supplemental Figure 1 24B GAL4-driven UAS-lacZ expression pattern during aging at 25˚C. (A) Quantitative analysis across age with the CPRG assay. Each time point is the average of 5 single-fly extracts. X-axis: age in days from pupal emergence. Y-axis: ß-galactosidase activity in ΔmOD562/min/fly. Error bars represent 2 standard deviations. (B) Qualitative analysis across age with the X-gal assay. am: abdominal muscles. br: brain. ds: digestive system. ey: eye. fm: flight muscles. lgm: leg muscles. lm: labium muscles. oe: oenocytes. sg: salivary glands. t: testis. vc: ventral chord.

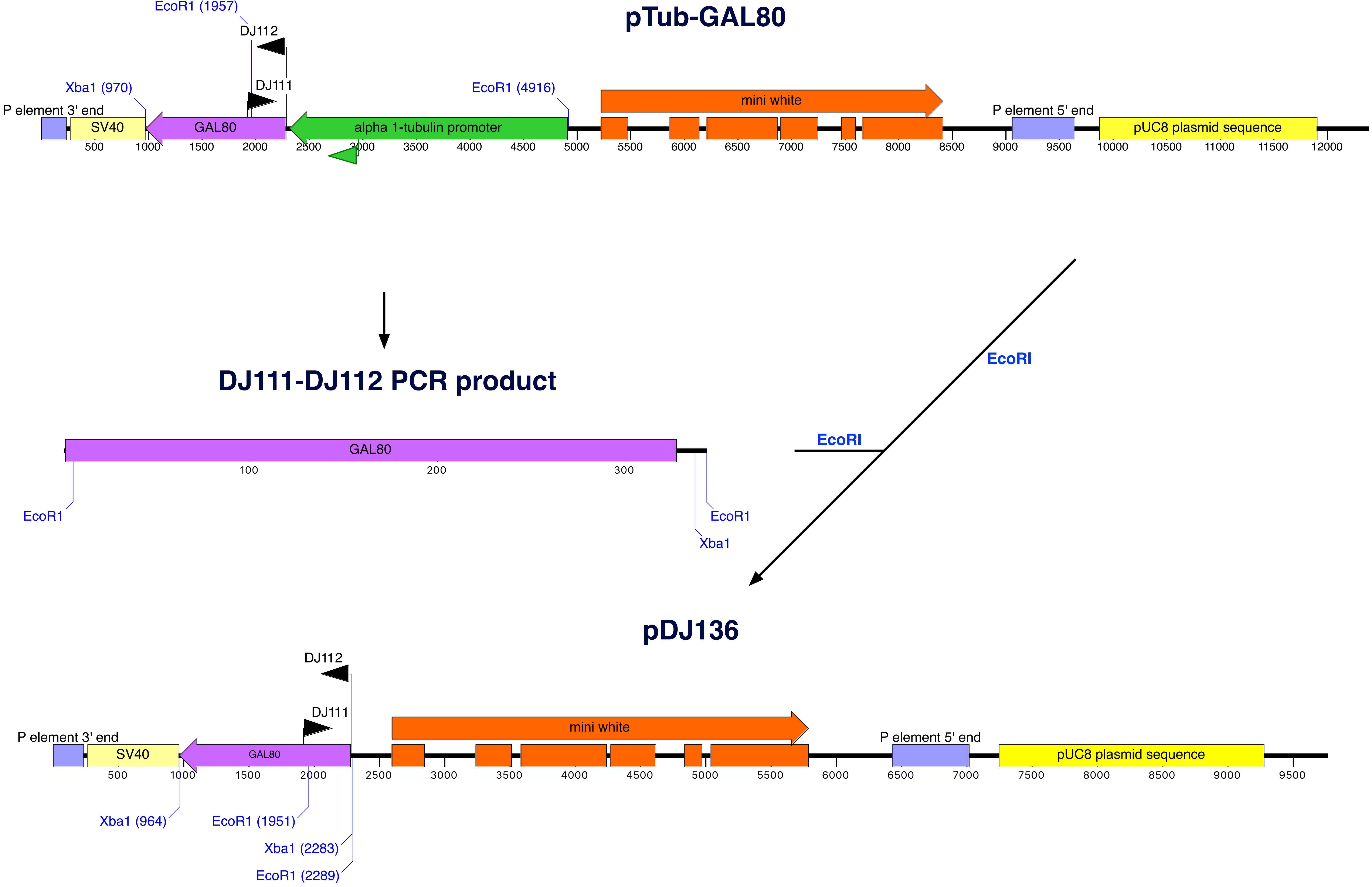

Supplemental Figure 2 Construction of pDJ136. Arrows above sequence indicate the location of primers used for PCR or sequencing. Arrow below sequence indicates the start of transcription of a promoter.

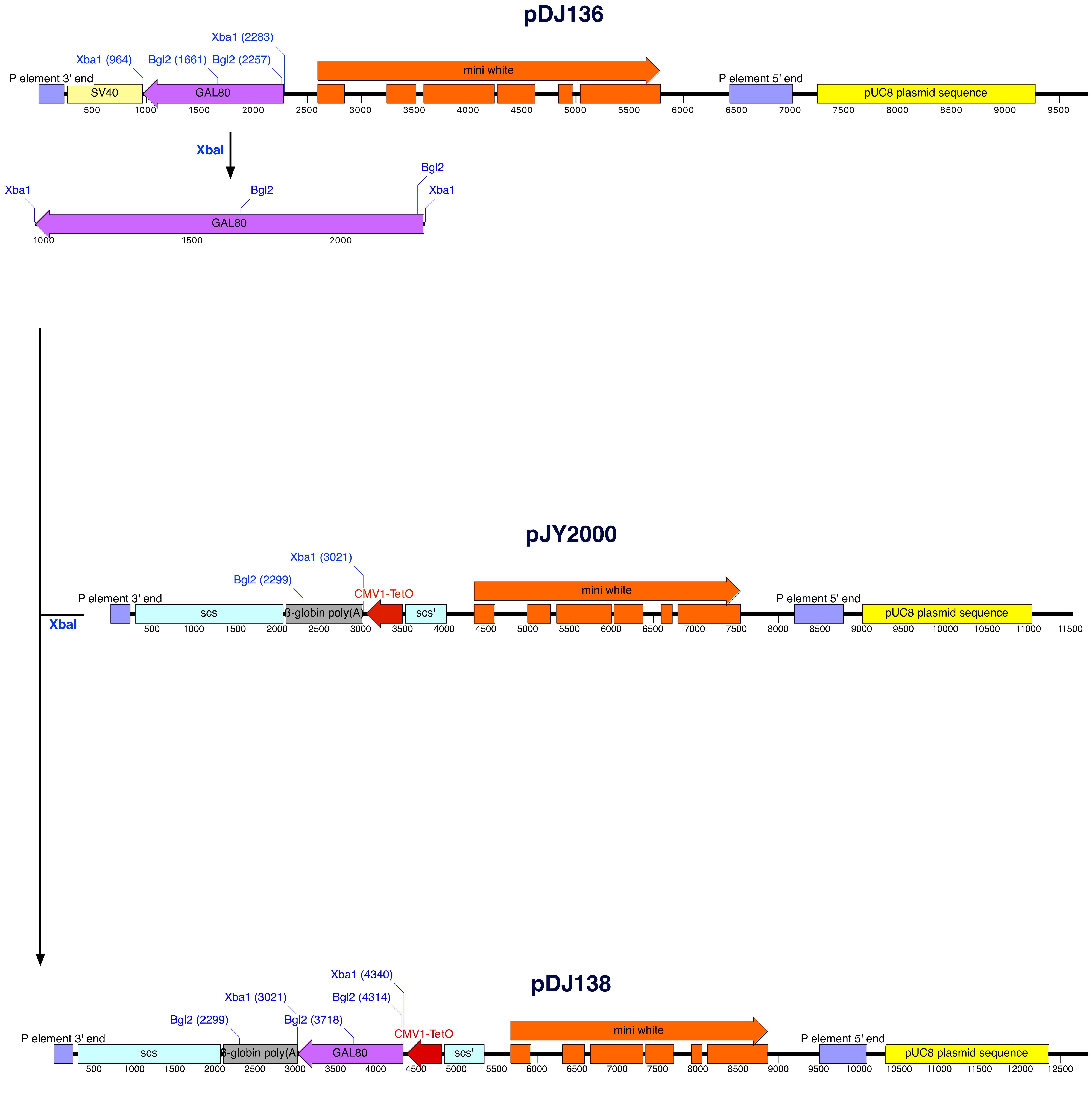

Supplemental Figure 3 Construction of pDJ138. scs: specialized chromatin structures.

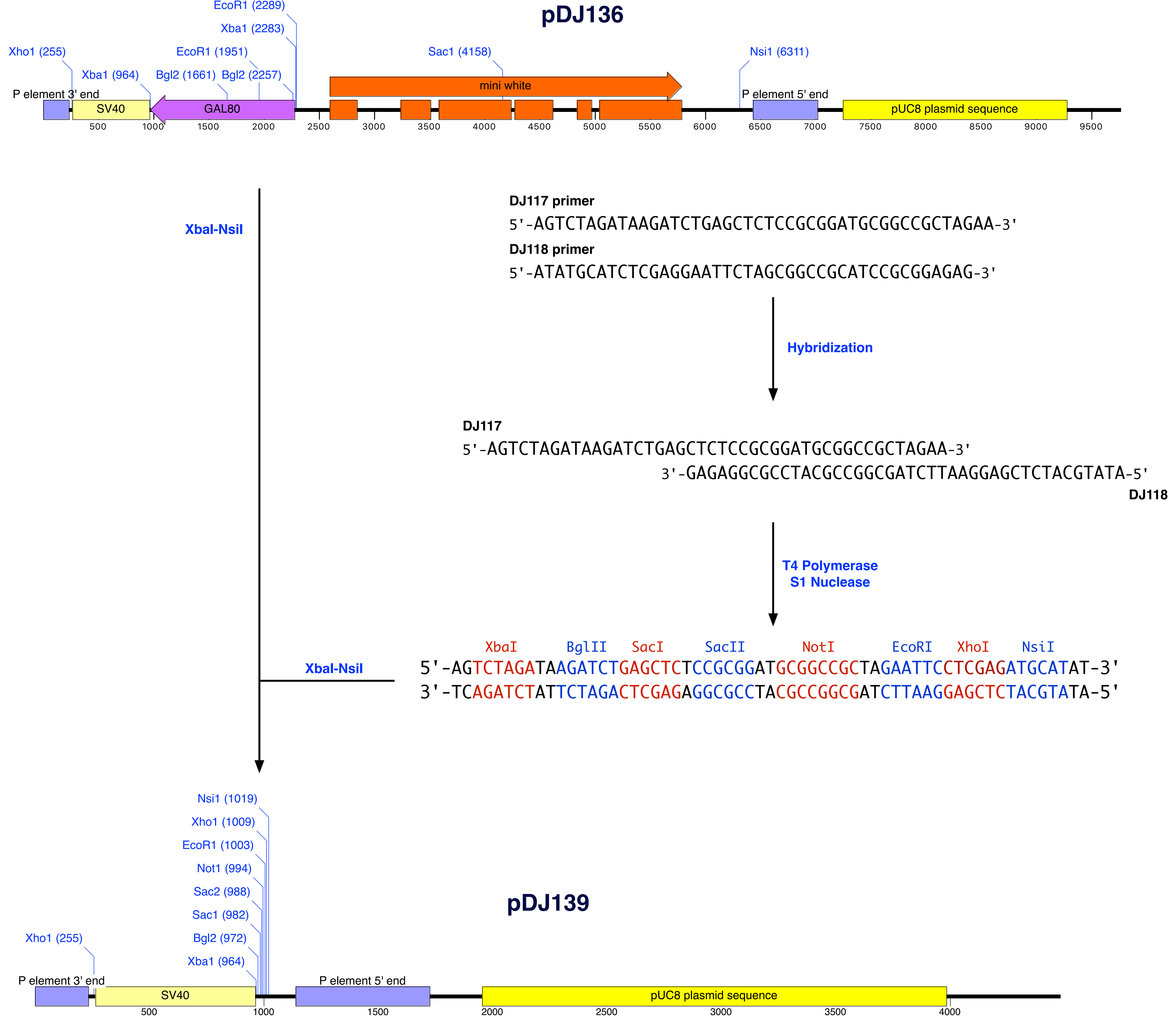

Supplemental Figure 4 Construction of pDJ139.

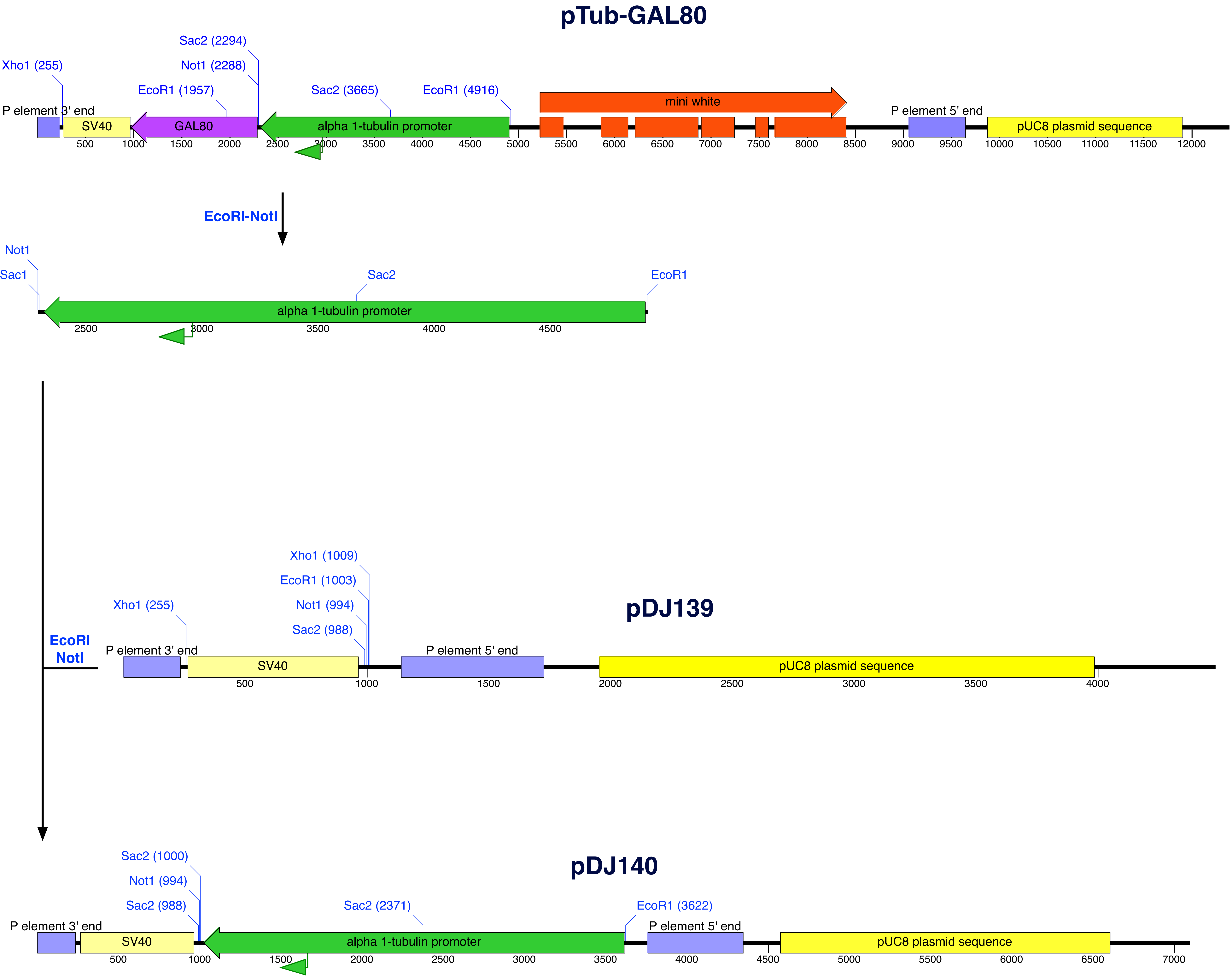

Supplemental Figure 5 Construction of pDJ140. Green arrow indicates the start of transcription.

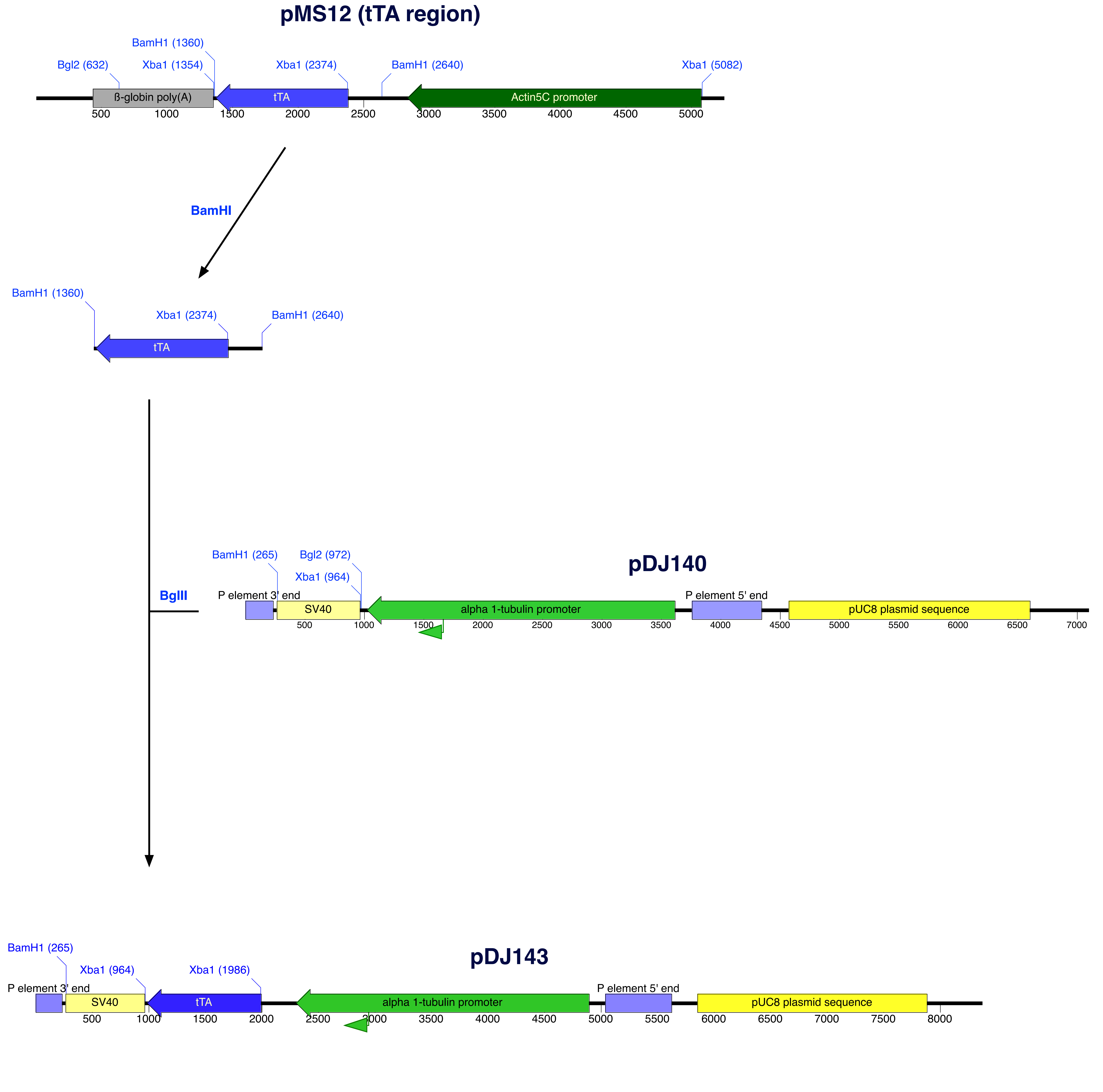

Supplemental Figure 6 Construction of pDJ143. Green arrow indicates the start of transcription.

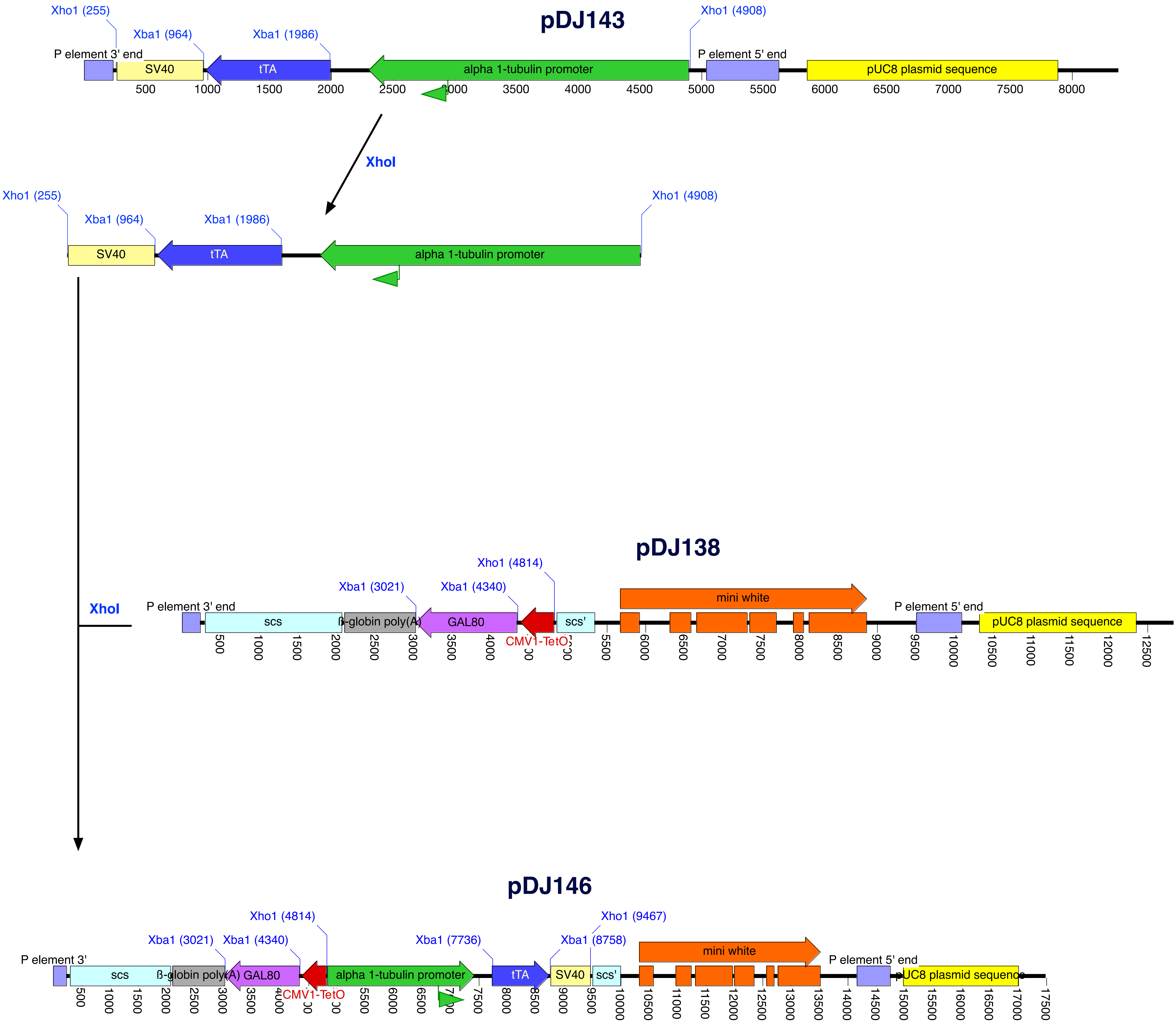

Supplemental Figure 7 Construction of pDJ146. Green arrow indicates the start of transcription.

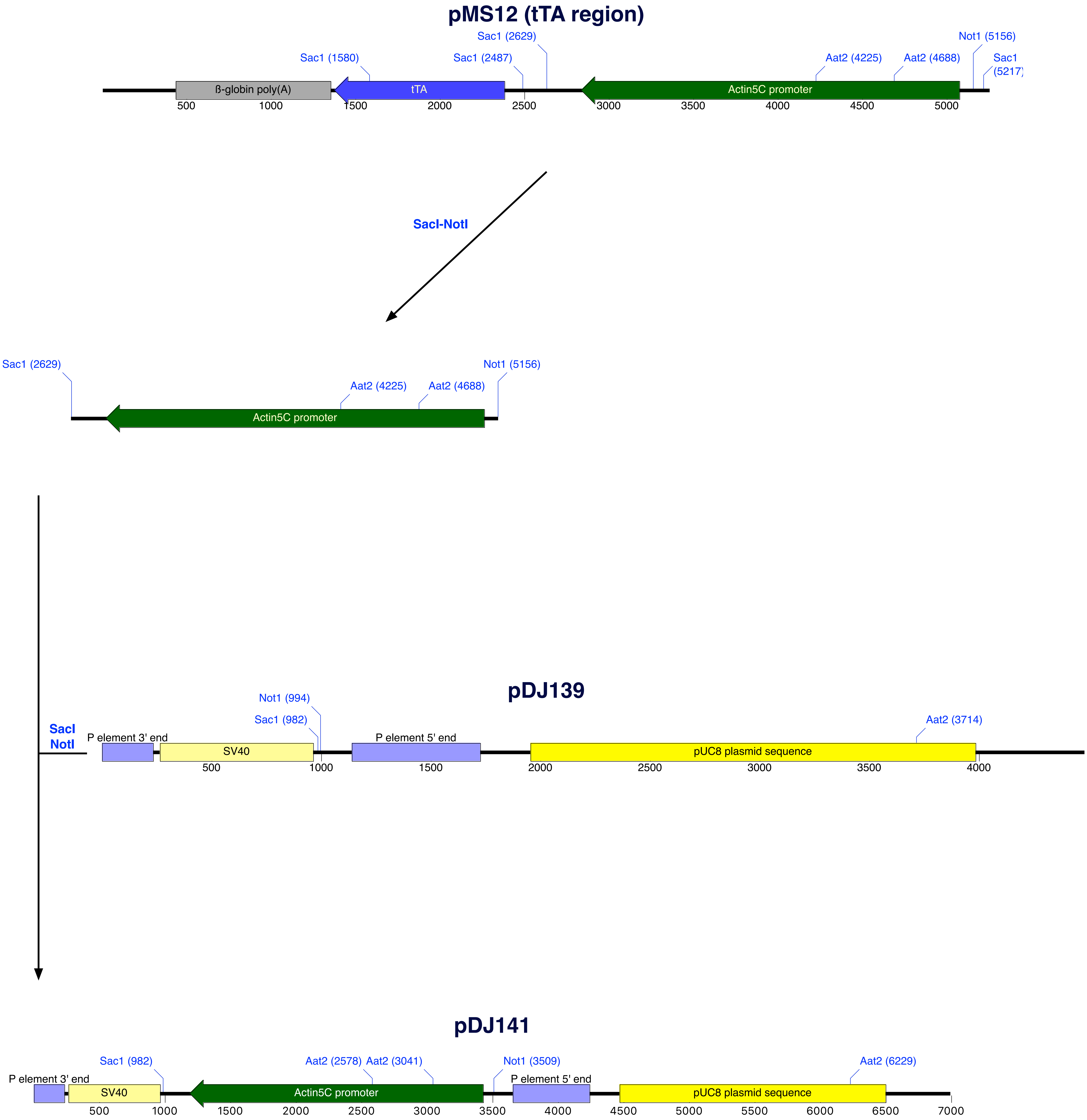

Supplemental Figure 8 Construction of pDJ141.

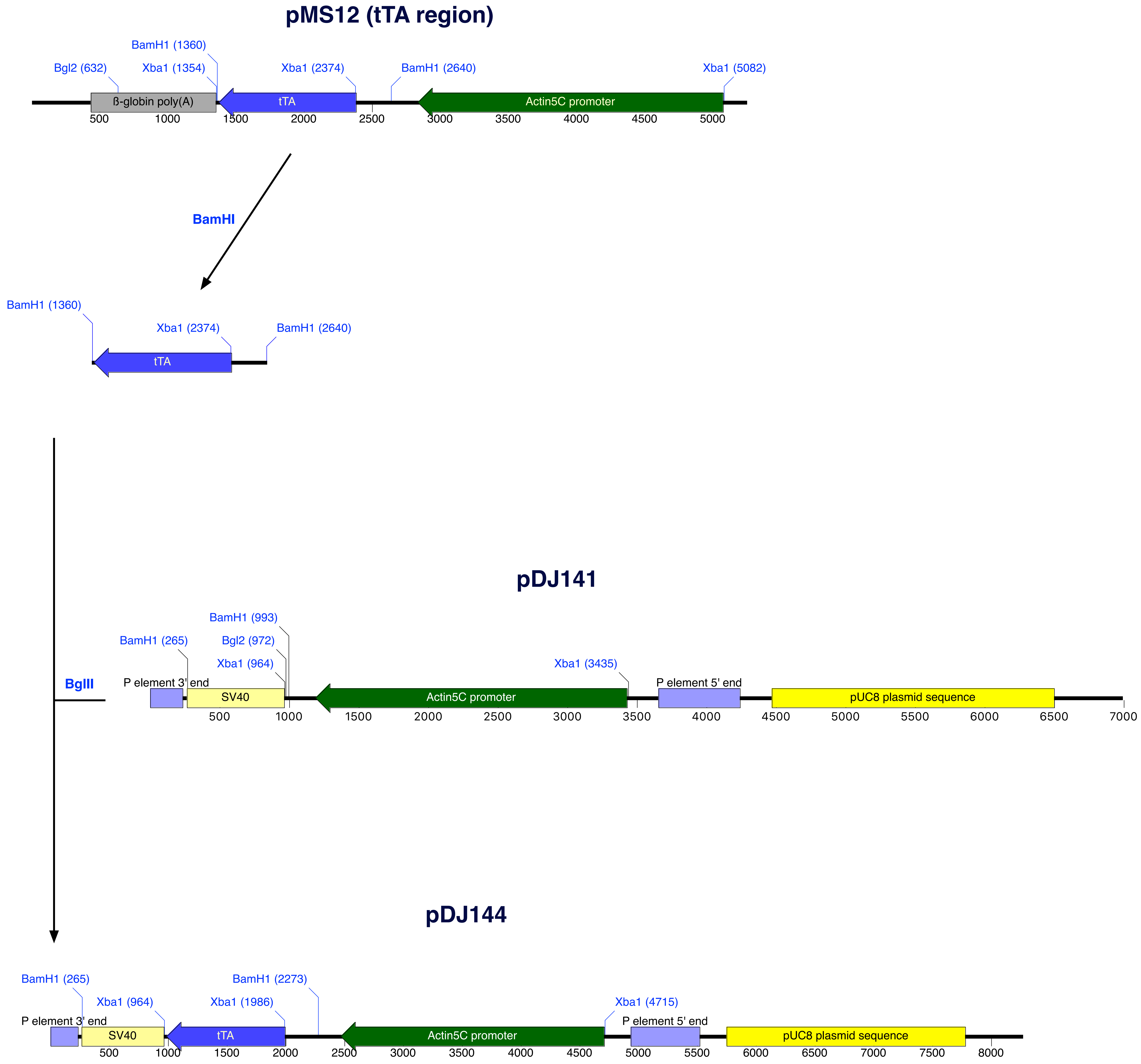

Supplemental Figure 9 Construction of pDJ144.

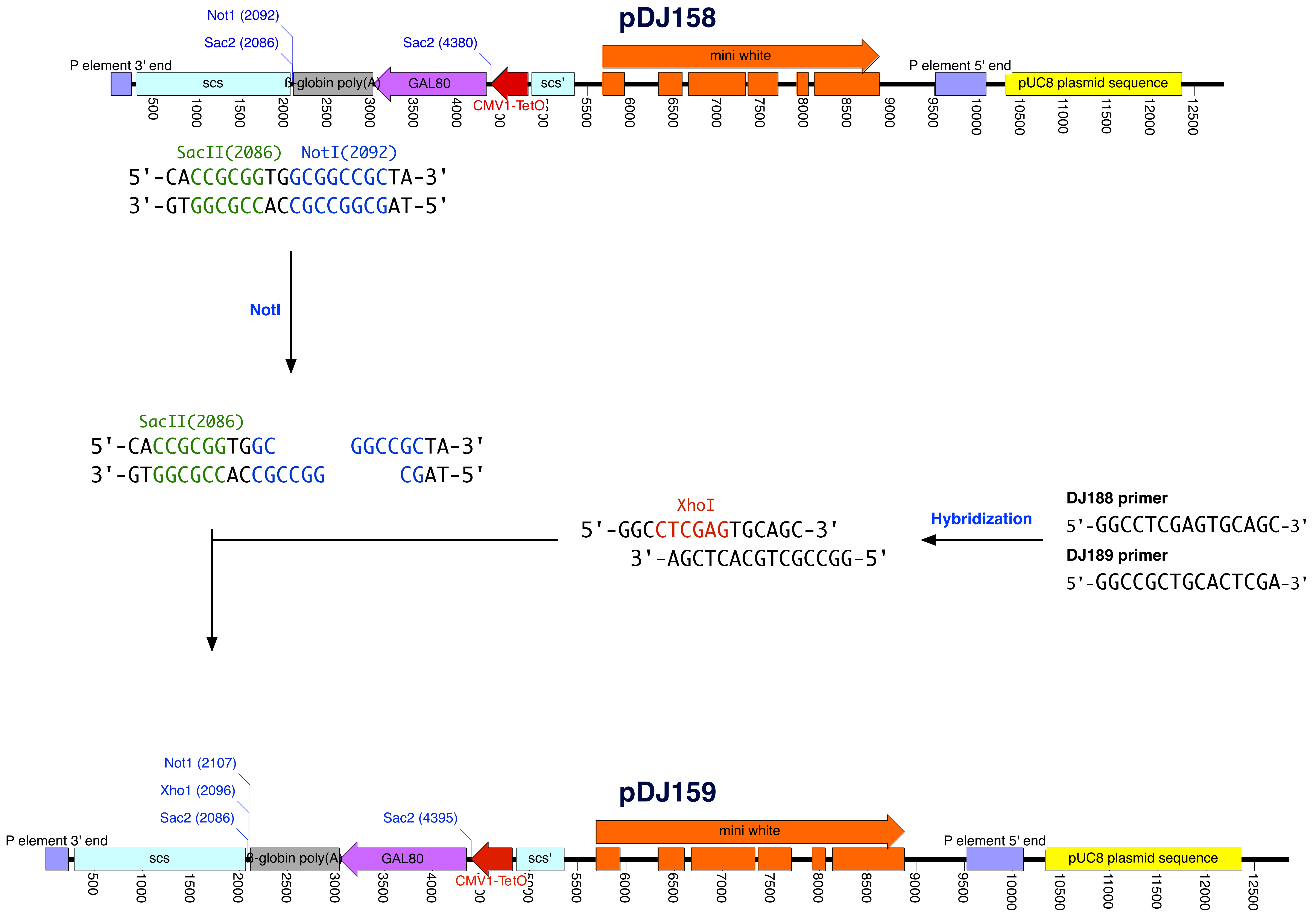

Supplemental Figure 10 Construction of pDJ159. pDJ158 is a derivative of pDJ138 lacking the *XhoI* between the *TetO* promoter and the scs' insulator element.

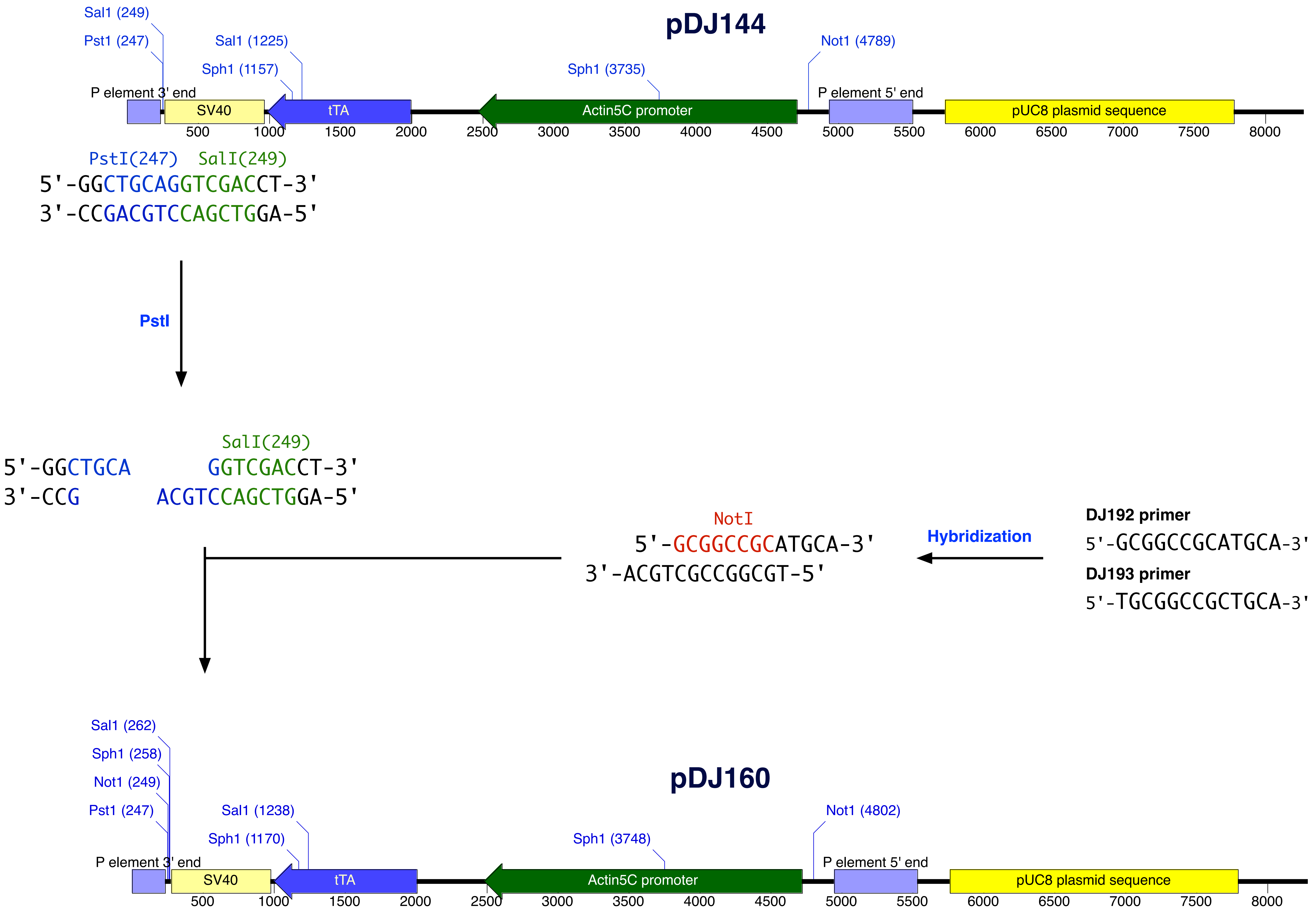

Supplemental Figure 11 Construction of pDJ160.

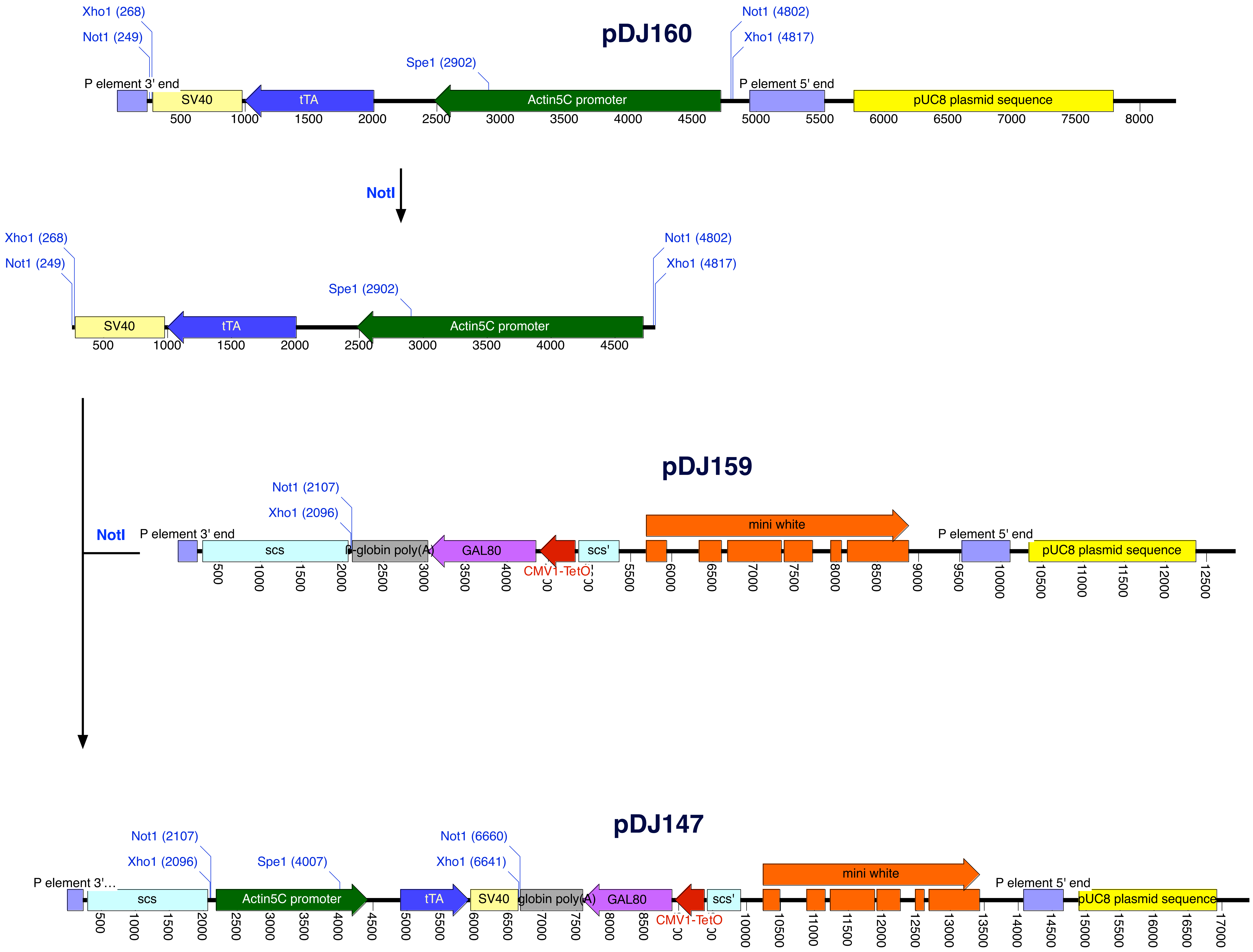

Supplemental Figure 12 Construction of pDJ147.

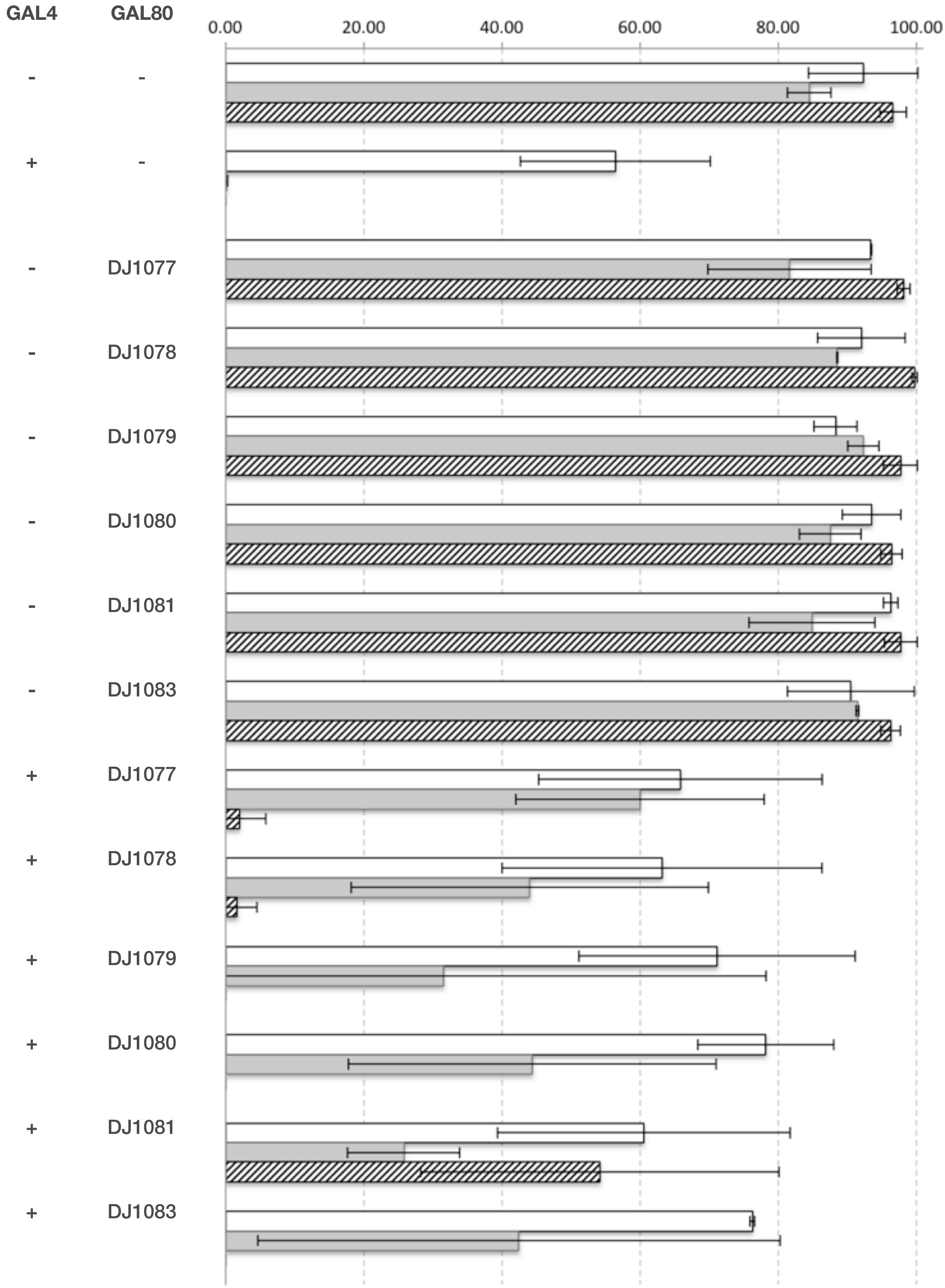

Supplemental Figure 13 Grim lethality assay of 1 copy DJ146 insertions treated with ampicillin (30µg/ml). Horizontal axes shows average percent survival (% survival calculated as a % of the previous developmental stage). White bars indicate % of L1 larvae, grey bars indicate % of pupae, and striped bars indicate % of adults. Errror bars represent ±2SD. Vertical axes indicates genotype, all genotypes contain a UAS-grim transgene but differ by the presence or absence of GAL4 and GAL80 insertion. GAL4 column displays presence (+) or absence (-) of GAL4 in the genotype, and the GAL80 column indicates the DJ146 insertion or an absence (-) of GAL80.

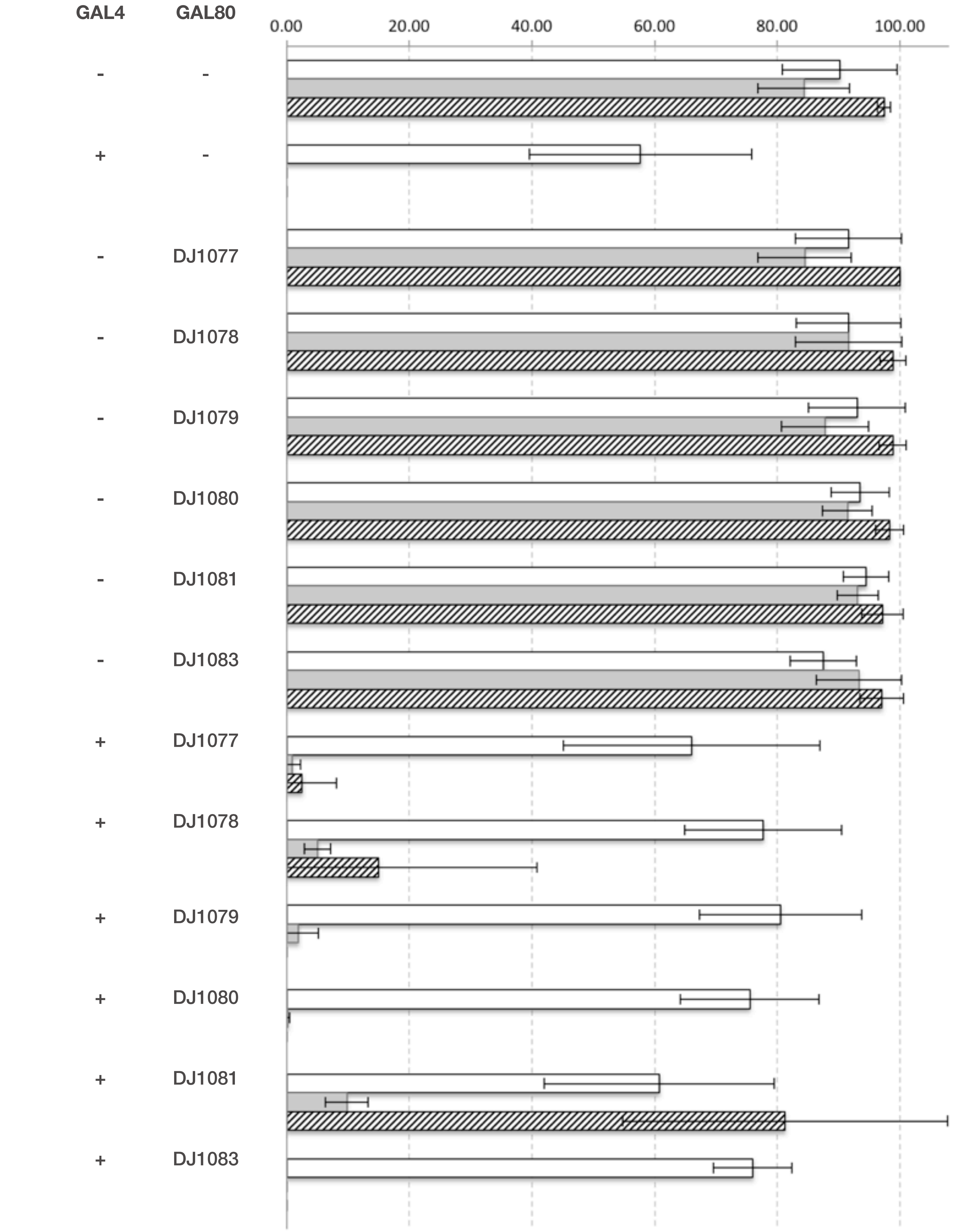

Supplemental Figure 14 Grim lethality assay of 1 copy DJ146 insertions treated with ampicillin (30µg/ml) and tetracycline (50µg/ml). Horizontal axes shows average percent survival (% survival calculated as a % of the previous developmental stage). White bars indicate % of L1 larvae, grey bars indicate % of pupae, and striped bars indicate % of adults Errror bars represent ±2SD. Vertical axes indicates genotype, all genotypes contain a UAS-grim transgene but differ by the presence or absence of GAL4 and GAL80 insertion. GAL4 column displays presence (+) or absence (-) of GAL4 in the genotype, and the GAL80 column indicates the DJ146 insertion or an absence (-) of GAL80.

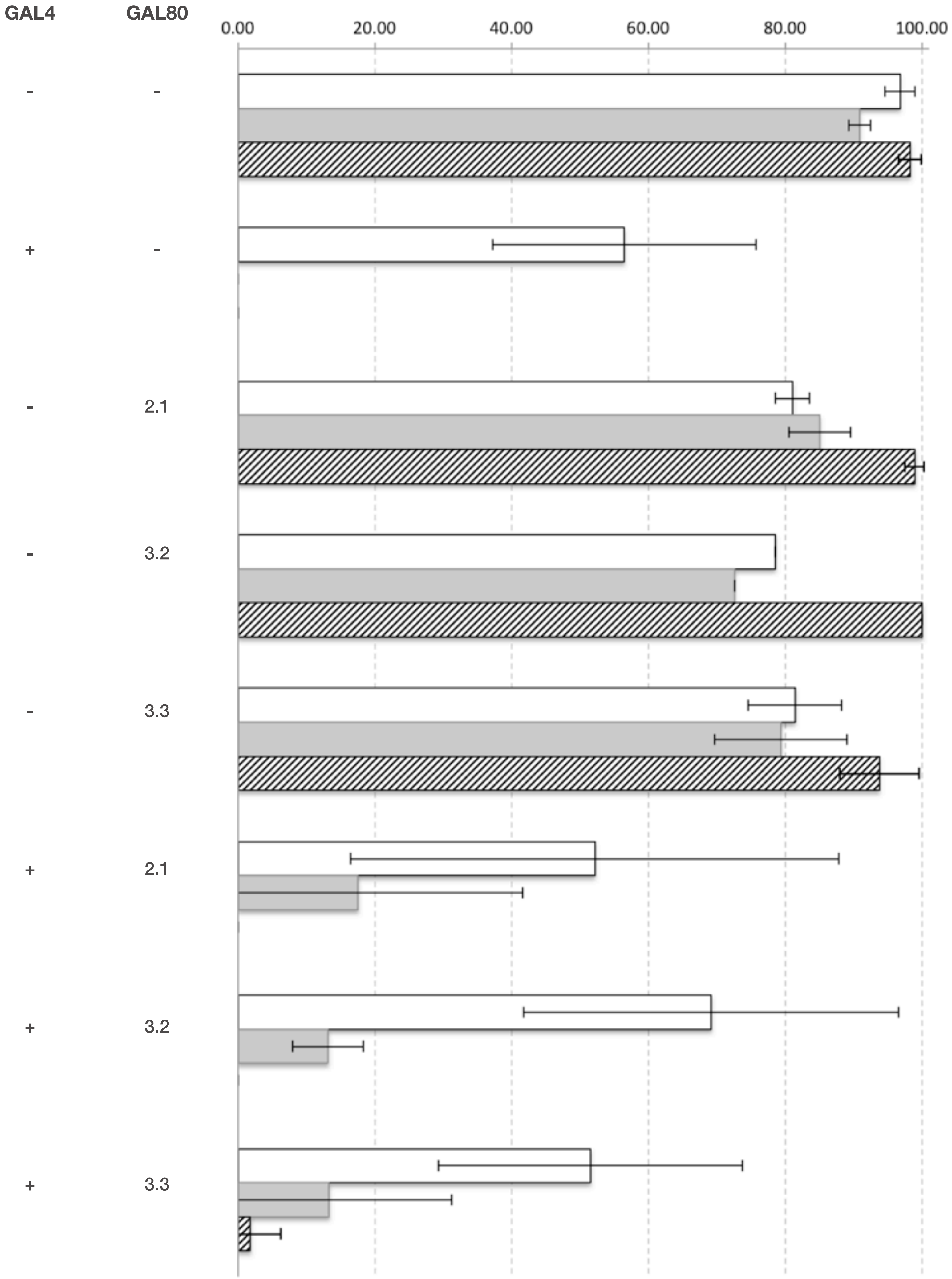

Supplemental Figure 15 Grim lethality assay of 1 copy of DJ147 insertion treated with ampicillin (30µg/ml). Horizontal axes shows average percent survival (% survival calculated as a % of the previous developmental stage). White bars indicate % of L1 larvae, grey bars indicate % of pupae, and striped bars indicate % of adults. Errror bars represent ±2SD. Vertical axes indicates genotype, all genotypes contain a UAS-grim transgene but differ by the presence or absence of GAL4 and GAL80 insertion. GAL4 column displays presence (+) or absence (-) of GAL4 in the genotype, and the GAL80 column indicates the DJ147 insertion or an absence (-) of GAL80.

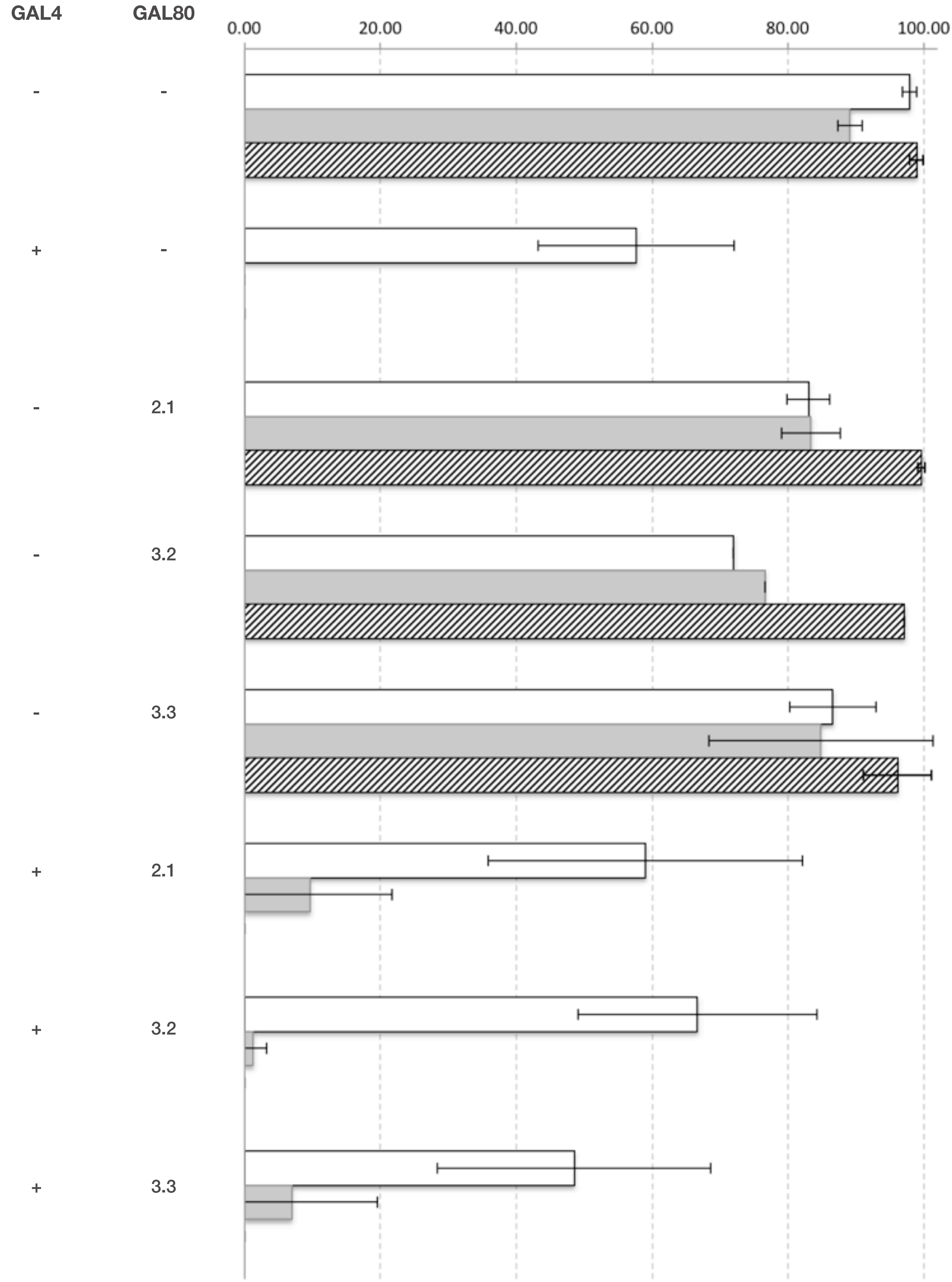

Supplemental Figure 16 Grim lethality assay of 1 copy of DJ147 insertion treated with ampicillin (30µg/ml) and tetracycline (50µg/ml). Horizontal axes shows average percent survival (% survival calculated as a % of the previous developmental stage). White bars indicate % of L1 larvae, grey bars indicate % of pupae, and striped bars indicate % of adults. Errror bars represent ±2SD. Vertical axes indicates genotype, all genotypes contain a UAS-grim transgene but differ by the presence or absence of GAL4 and GAL80 insertion. GAL4 column displays presence (+) or absence (-) of GAL4 in the genotype, and the GAL80 column indicates the DJ147 insertion or an absence (-) of GAL80.

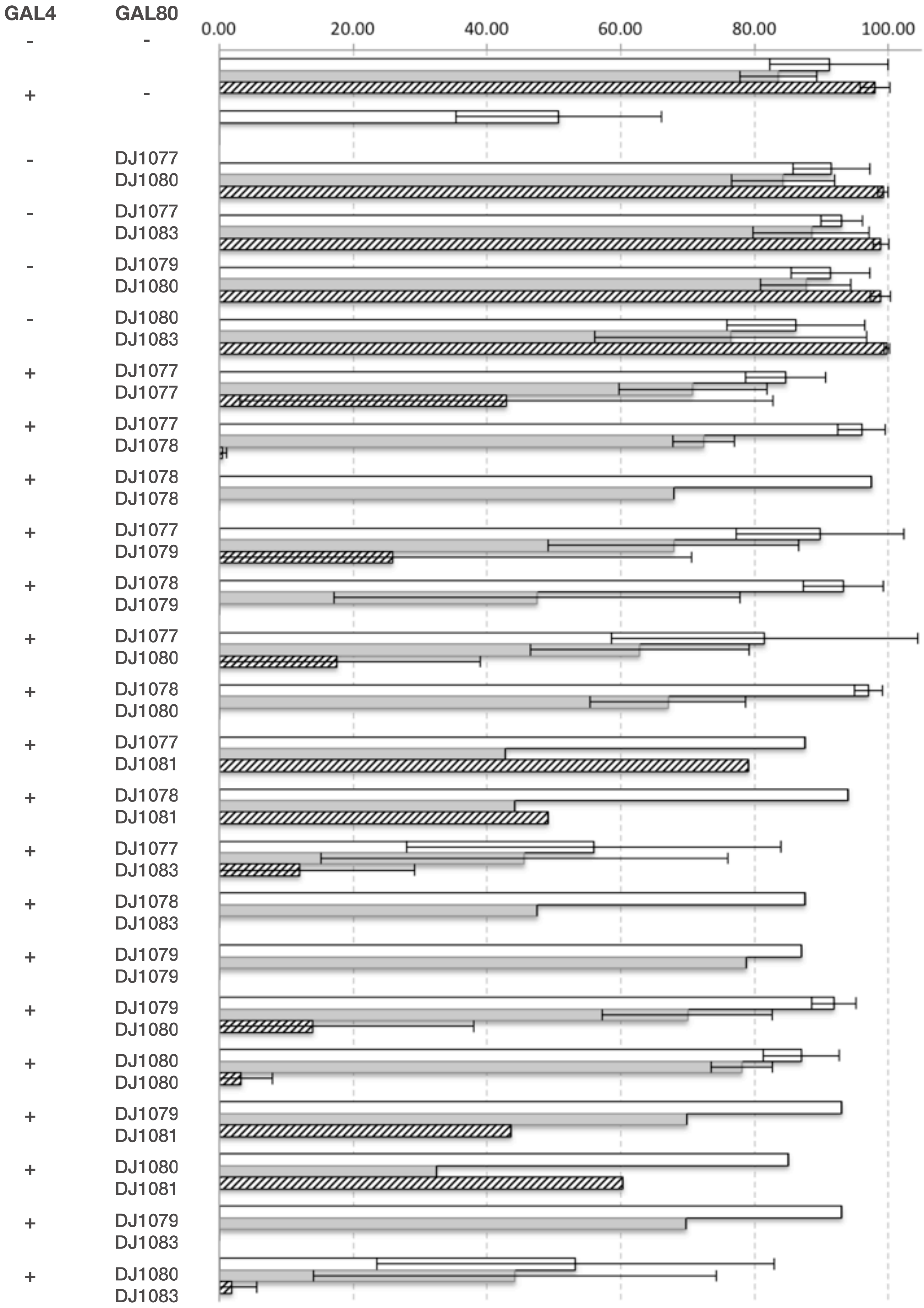

Supplemental Figure 17 Grim lethality assay of 2 copies of DJ146 insertions treated with ampicillin (30µg/ml). Horizontal axes shows average percent survival (% survival calculated as a % of the previous developmental stage). White bars indicate % of L1 larvae, grey bars indicate % of pupae, and striped bars indicate % of adults. Errror bars represent ±2SD. Vertical axes indicates genotype, all genotypes contain a UAS-grim transgene but differ by the presence or absence of GAL4 and GAL80 insertion. GAL4 column displays presence (+) or absence (-) of GAL4 in the genotype, and the GAL80 column indicates the DJ146 insertions or an absence (-) of GAL80.

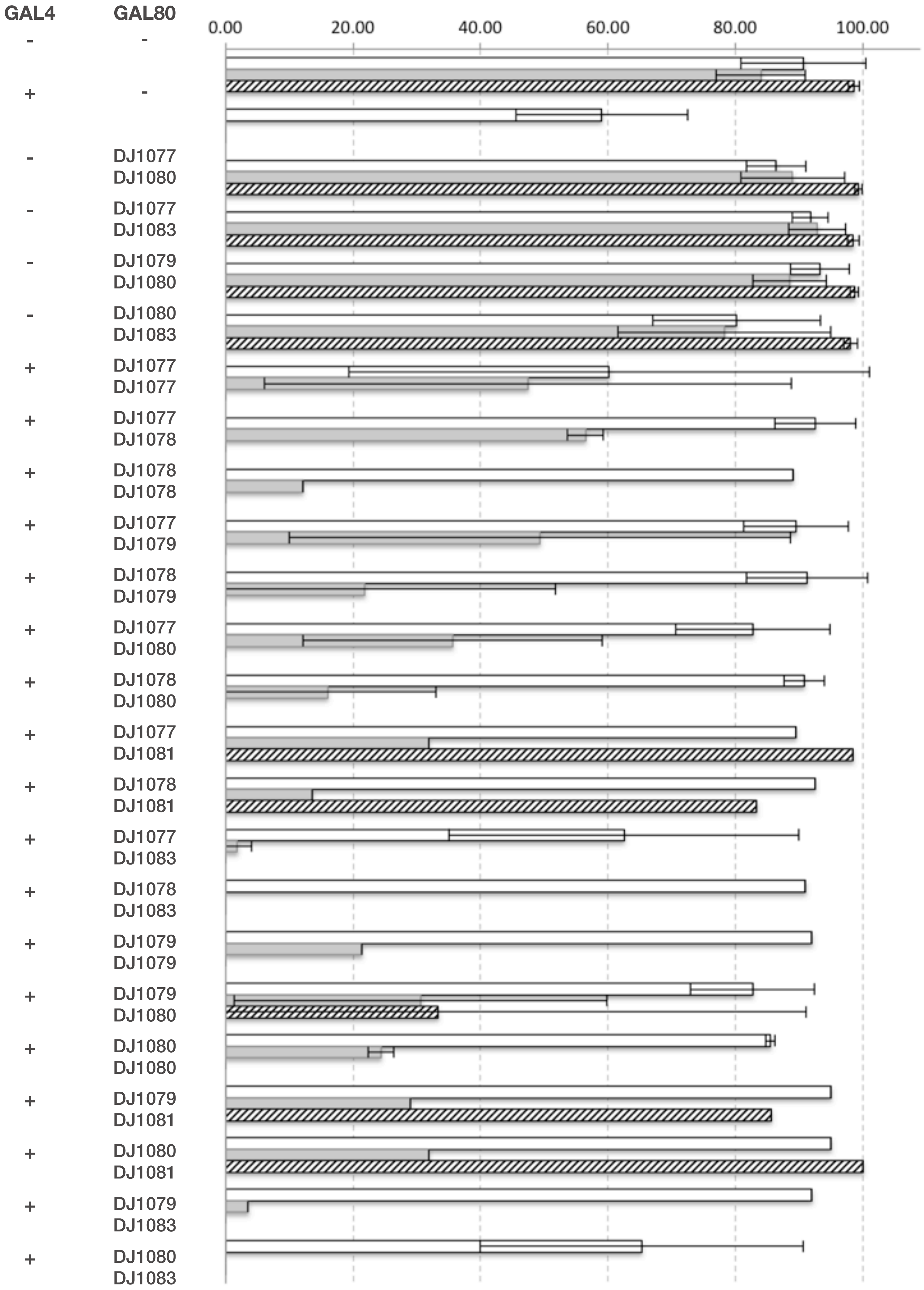

Supplemental Figure 18 Grim lethality assay of 2 copies of DJ146 insertions treated with ampicillin (30µg/ml) and tetracycline (50µg/ml). Horizontal axes shows average percent survival (% survival calculated as a % of the previous developmental stage). White bars indicate % of L1 larvae, grey bars indicate % of pupae, and striped bars indicate % of adults. Errror bars represent ±2SD. Vertical axes indicates genotype, all genotypes contain a UAS-grim transgene but differ by the presence or absence of GAL4 and GAL80 insertion. GAL4 column displays presence (+) or absence (-) of GAL4 in the genotype, and the GAL80 column indicates the DJ146 insertions or an absence (-) of GAL80.

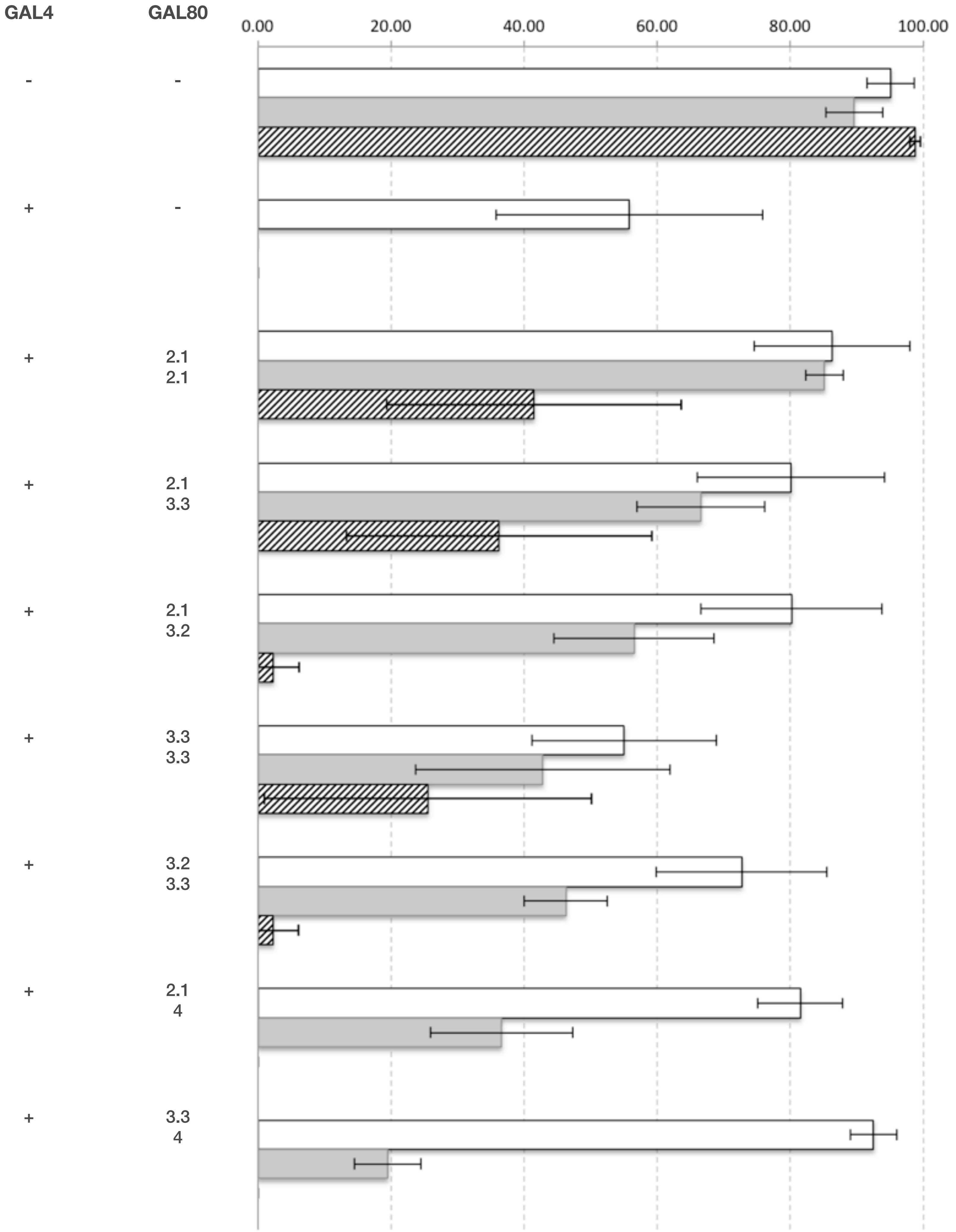

Supplemental Figure 19 Grim lethality assay of 2 copies of DJ147 insertions treated with ampicillin (30µg/ml). Horizontal axes shows average percent survival (% survival calculated as a % of the previous developmental stage). White bars indicate % of L1 larvae, grey bars indicate % of pupae, and striped bars indicate % of adults. Errror bars represent ±2SD. Vertical axes indicates genotype, all genotypes contain a UAS-grim transgene but differ by the presence or absence of GAL4 and GAL80 insertion. GAL4 column displays presence (+) or absence (-) of GAL4 in the genotype, and the GAL80 column indicates the DJ147 insertions or an absence (-) of GAL80.

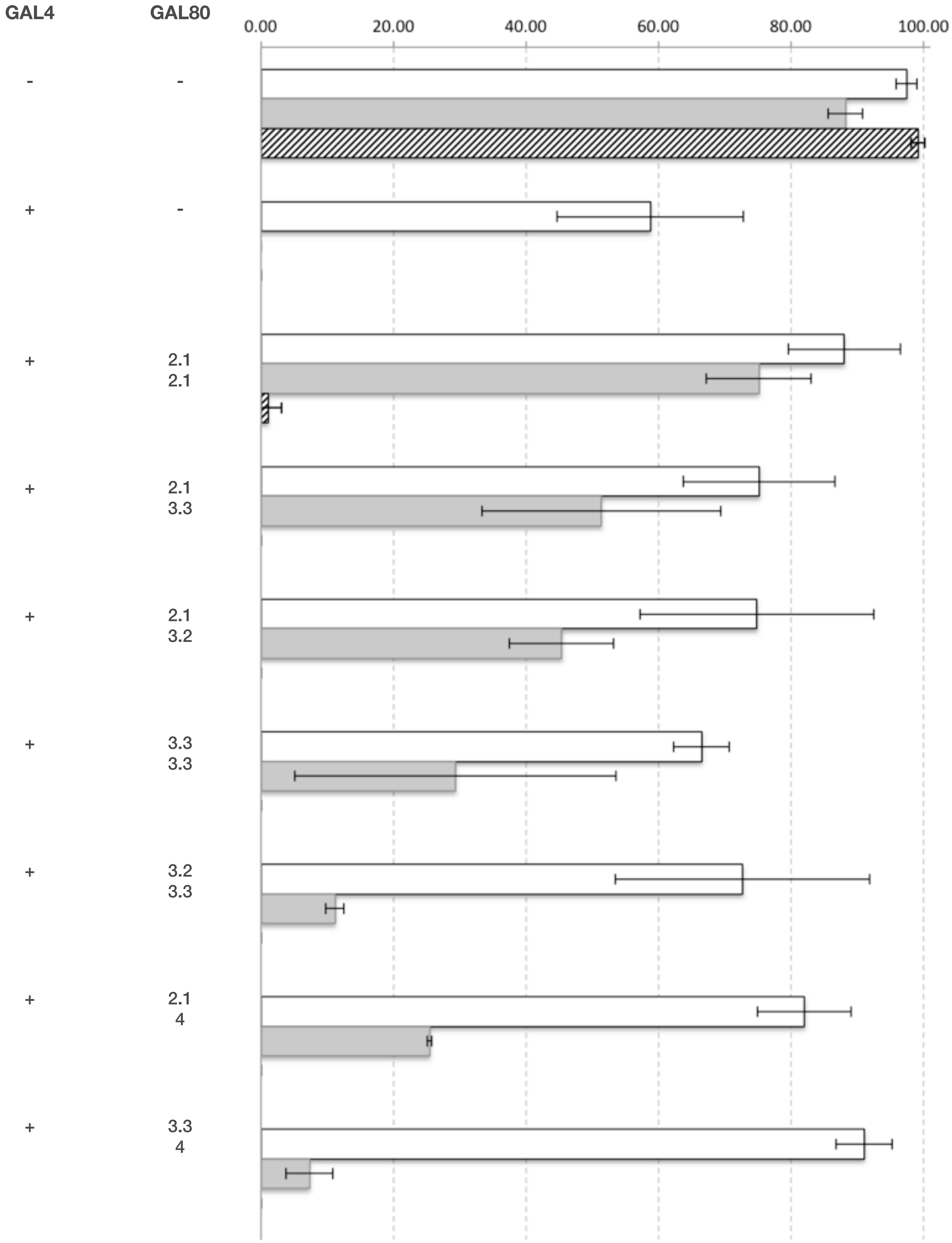

Supplemental Figure 20 Grim lethality assay of 2 copies of DJ147 insertions treated with ampicillin (30µg/ml) and tetracycline (50µg/ml). Horizontal axes shows average percent survival (% survival calculated as a % of the previous developmental stage). White bars indicate % of L1 larvae, grey bars indicate % of pupae, and striped bars indicate % of adults. Errror bars represent ±2SD. Vertical axes indicates genotype, all genotypes contain a UAS-grim transgene but differ by the presence or absence of GAL4 and GAL80 insertion. GAL4 column displays presence (+) or absence (-) of GAL4 in the genotype, and the GAL80 column indicates the DJ147 insertions or an absence (-) of GAL80.

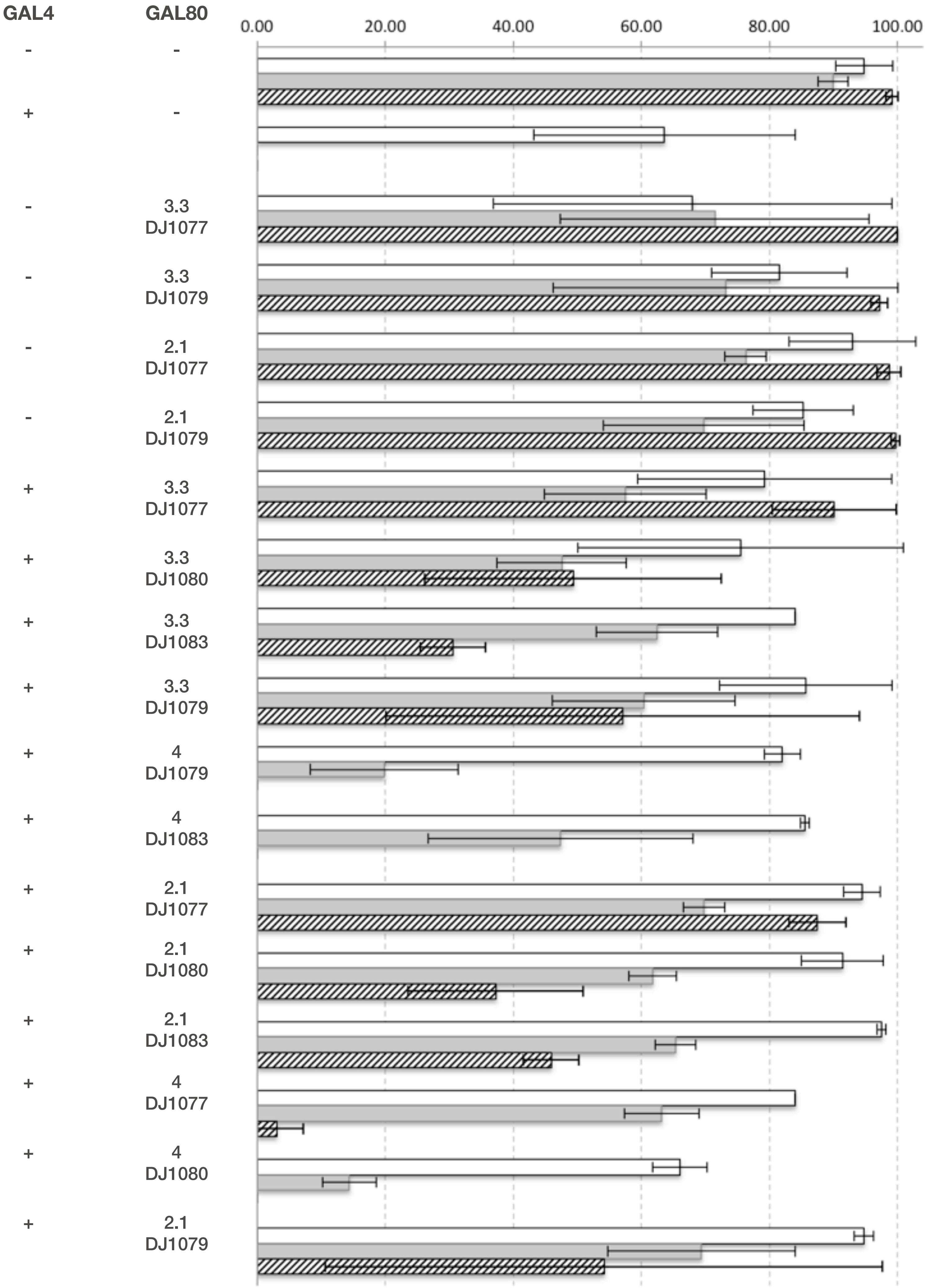

Supplemental Figure 21 Grim lethality assay of 1 copy of DJ147 insertion and 1 copy of DJ146 insertion treated with ampicillin (30µg/ml). Horizontal axes shows average percent survival (% survival calculated as a % of the previous developmental stage). White bars indicate % of L1 larvae, grey bars indicate % of pupae, and striped bars indicate % of adults. Errror bars represent ±2SD. Vertical axes indicates genotype, all genotypes contain a UAS-grim transgene but differ by the presence or absence of GAL4 and GAL80 insertion. GAL4 column displays presence (+) or absence (-) of GAL4 in the genotype, and the GAL80 column indicates the DJ147 and DJ146 insertions or an absence (-) of GAL80.

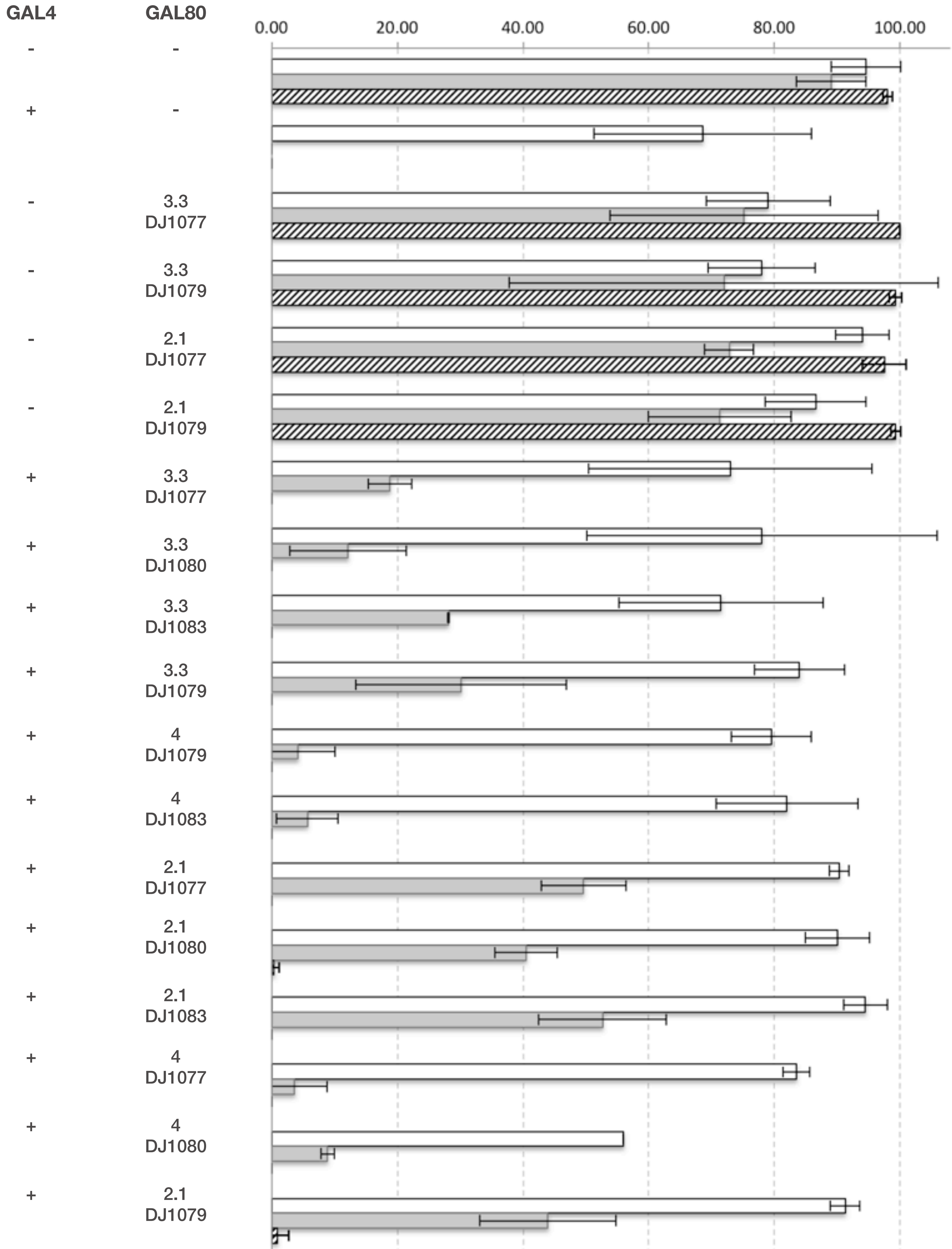

Supplemental Figure 22 Grim lethality assay of 1 copy of DJ147 insertion and 1 copy of DJ146 insertion treated with ampicillin (30µg/ml) and tetracycline (50µg/ml). Horizontal axes shows average percent survival (% survival calculated as a % of the previous developmental stage). White bars indicate % of L1 larvae, grey bars indicate % of pupae, and striped bars indicate % of adults. Errror bars represent ±2SD. Vertical axes indicates genotype, all genotypes contain a UAS-grim transgene but differ by the presence or absence of GAL4 and GAL80 insertion. GAL4 column displays presence (+) or absence (-) of GAL4 in the genotype, and the GAL80 column indicates the DJ147 and DJ146 insertions or an absence (-) of GAL80.

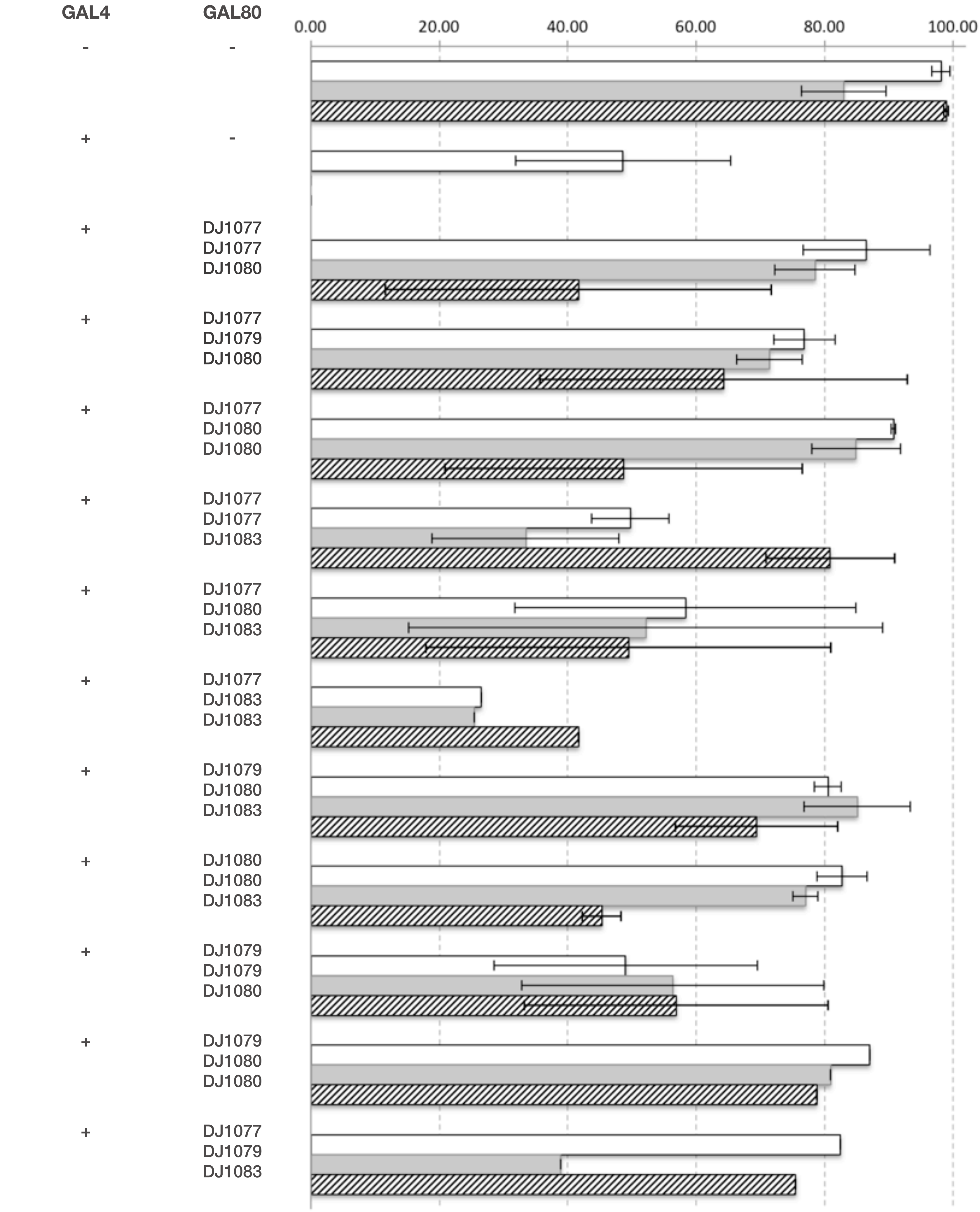

Supplemental Figure 23 Grim lethality assay of 3 copies of DJ146 insertions treated with ampicillin (30μg/ml). Horizontal axes shows average percent survival (% survival calculated as a % of the previous developmental stage). White bars indicate % of L1 larvae, grey bars indicate % of pupae, and striped bars indicate % of adults. Errror bars represent ±2SD. Vertical axes indicates genotype, all genotypes contain a UAS-grim transgene but differ by the presence or absence of GAL4 and GAL80 insertion. GAL4 column displays presence (+) or absence (-) of GAL4 in the genotype, and the GAL80 column indicates the DJ146 insertions or an absence (-) of GAL80.

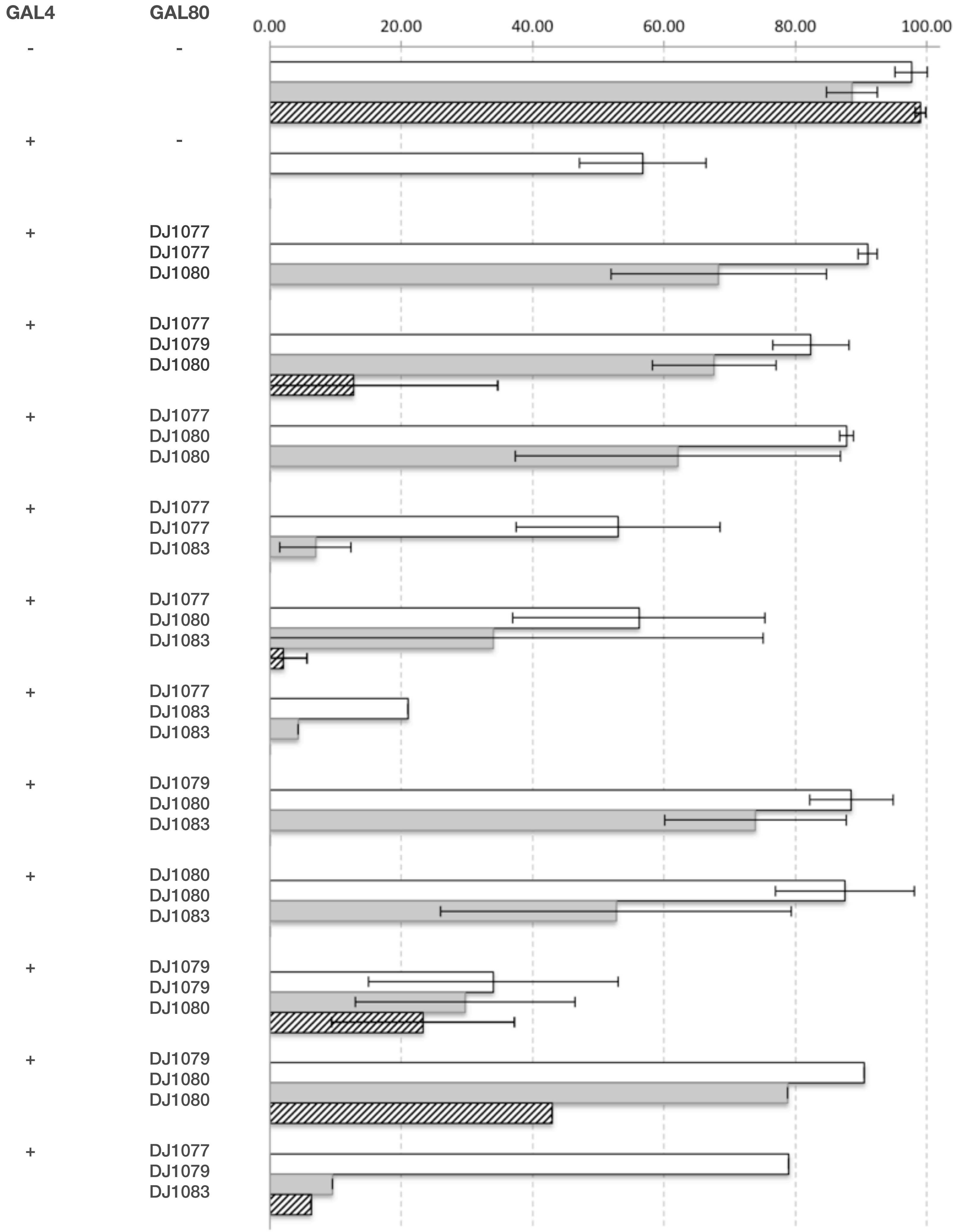

Supplemental Figure 24 Grim lethality assay of 3 copies of DJ146 insertions treated with ampicillin (30µg/ml) and tetracycline (50µg/ml). Horizontal axes shows average percent survival (% survival calculated as a % of the previous developmental stage). White bars indicate % of L1 larvae, grey bars indicate % of pupae, and striped bars indicate % of adults. Errror bars represent ±2SD. Vertical axes indicates genotype, all genotypes contain a UAS-grim transgene but differ by the presence or absence of GAL4 and GAL80 insertion. GAL4 column displays presence (+) or absence (-) of GAL4 in the genotype, and the GAL80 column indicates the DJ146 insertions or an absence (-) of GAL80.

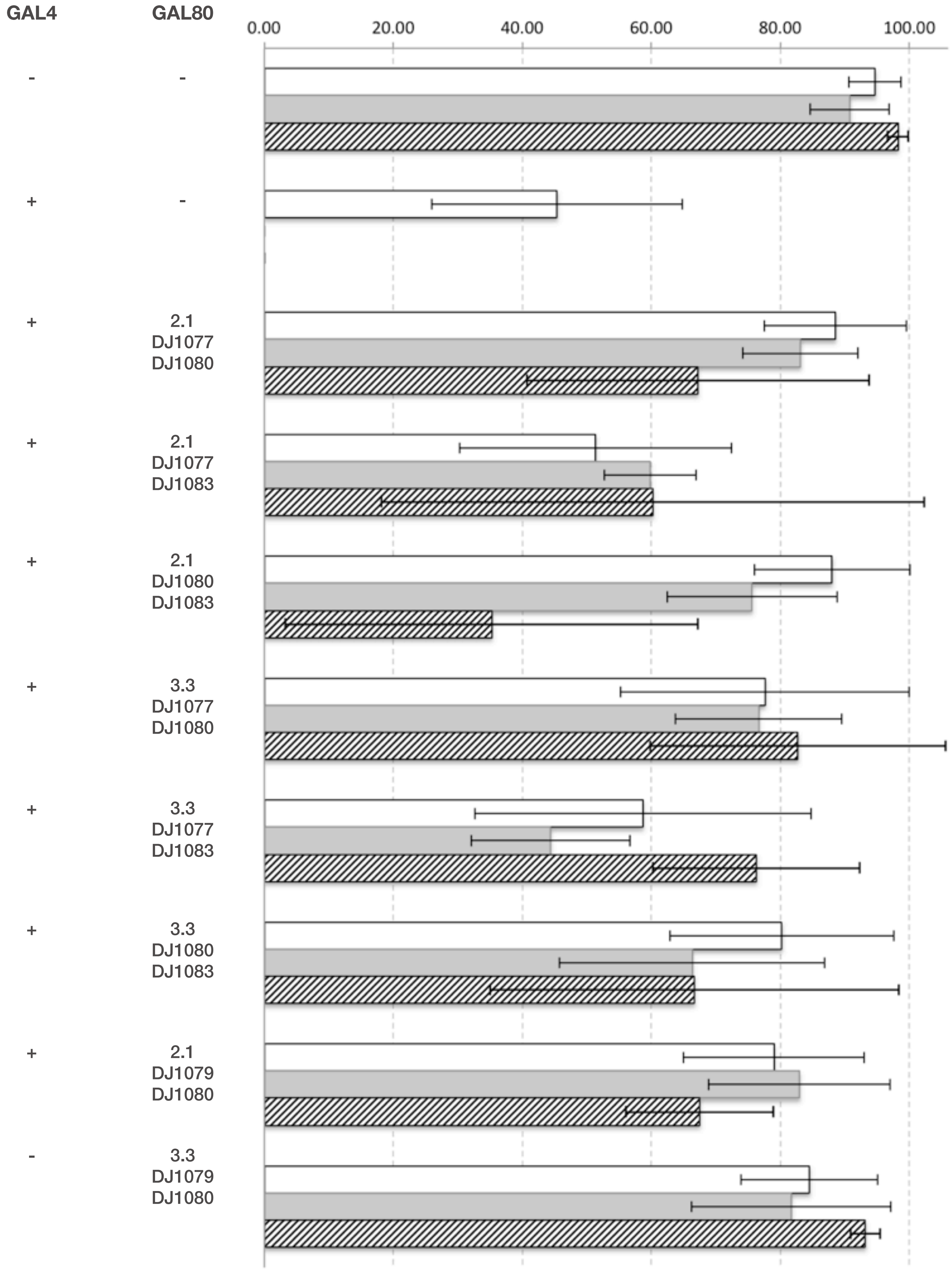

Supplemental Figure 25 Grim lethality assay of 1 copy of DJ147 insertions and 2 copies of DJ146 insertions treated with ampicillin (30µg/ml). Horizontal axes shows average percent survival (% survival calculated as a % of the previous developmental stage). White bars indicate % of L1 larvae, grey bars indicate % of pupae, and striped bars indicate % of adults. Errror bars represent ±2SD. Vertical axes indicates genotype, all genotypes contain a UAS-grim transgene but differ by the presence or absence of GAL4 and GAL80 insertion. GAL4 column displays presence (+) or absence (-) of GAL4 in the genotype, and the GAL80 column indicates the DJ146 and DJ147 insertions or an absence (-) of GAL80.

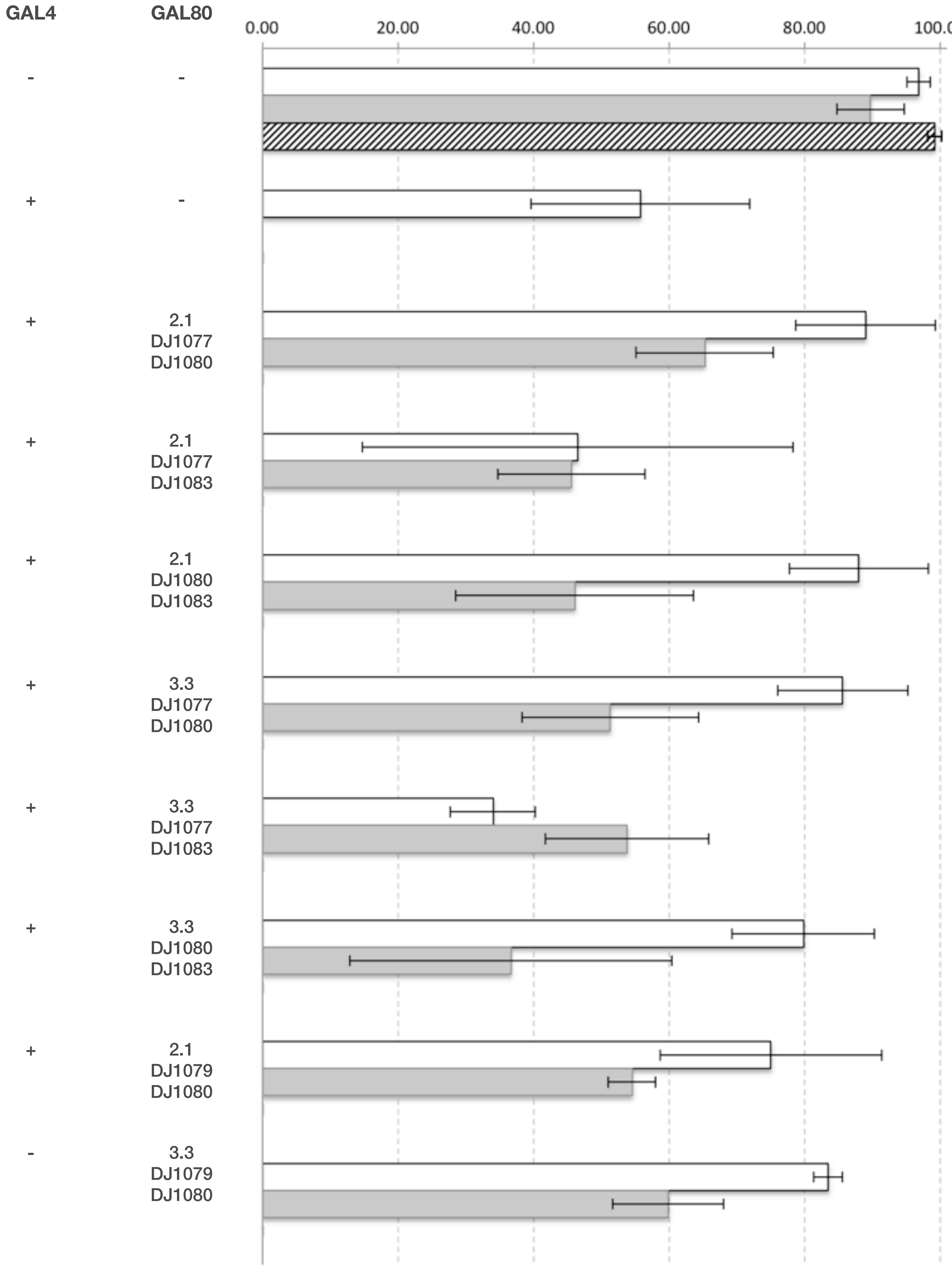

Supplemental Figure 26 Grim lethality assay of 1 copy of DJ147 insertions and 2 copies of DJ146 insertions treated with ampicillin (30μg/ml) and tetracycline (50μg/ml). Horizontal axes shows average percent survival (% survival calculated as a % of the previous developmental stage). White bars indicate % of L1 larvae, grey bars indicate % of pupae, and striped bars indicate % of adults. Errror bars represent ±2SD. Vertical axes indicates genotype, all genotypes contain a UAS-grim transgene but differ by the presence or absence of GAL4 and GAL80 insertion. GAL4 column displays presence (+) or absence (-) of GAL4 in the genotype, and the GAL80 column indicates the DJ146 and DJ147 insertions or an absence (-) of GAL80.

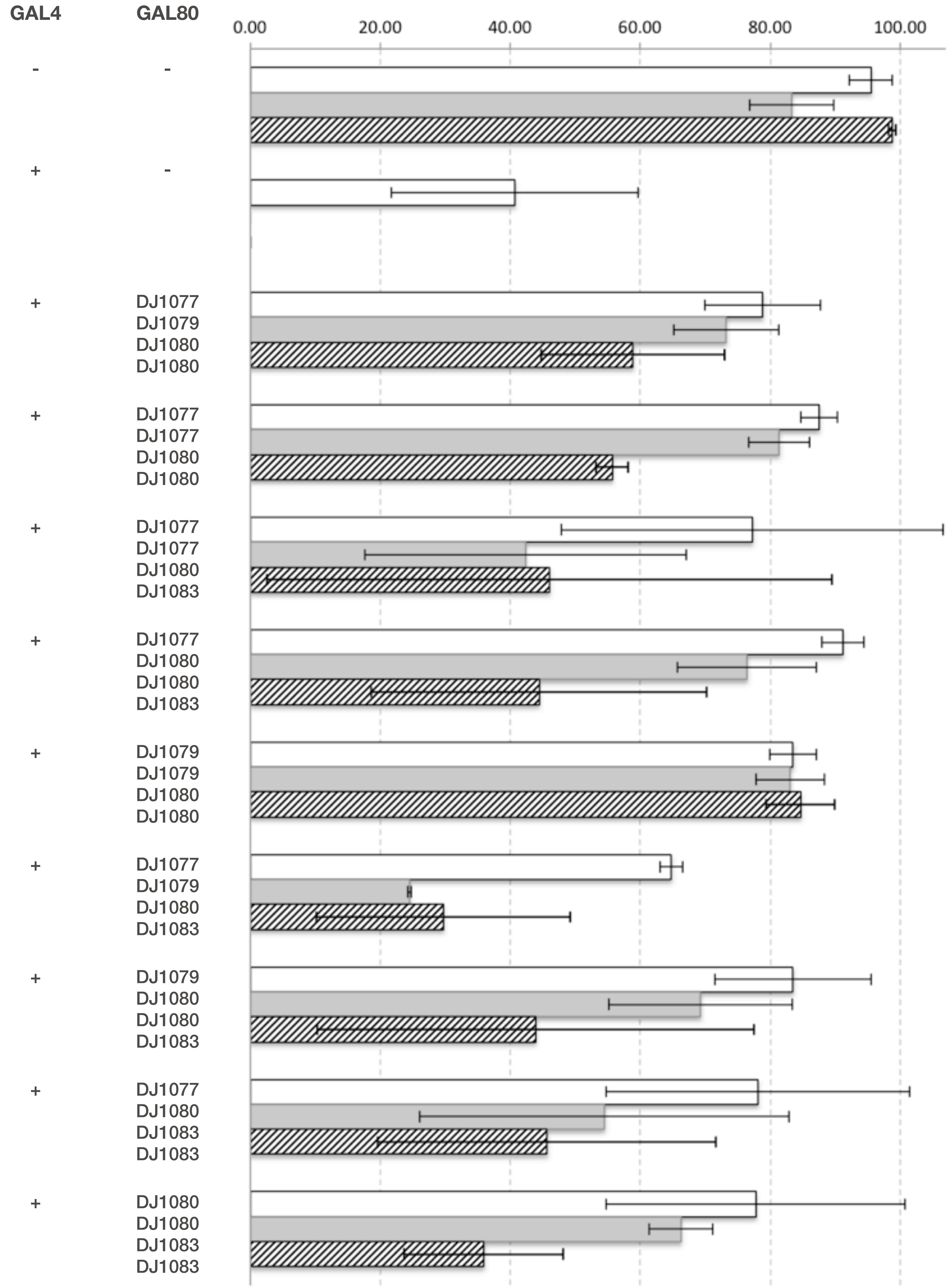

Supplemental Figure 27 Grim lethality assay of 4 copies of DJ146 insertions treated with ampicillin (30µg/ml). Horizontal axes shows average percent survival (% survival calculated as a % of the previous developmental stage). White bars indicate % of L1 larvae, grey bars indicate % of pupae, and striped bars indicate % of adults. Errror bars represent ±2SD. Vertical axes indicates genotype, all genotypes contain a UAS-grim transgene but differ by the presence or absence of GAL4 and GAL80 insertion. GAL4 column displays presence (+) or absence (-) of GAL4 in the genotype, and the GAL80 column indicates the DJ146 insertions or an absence (-) of GAL80.

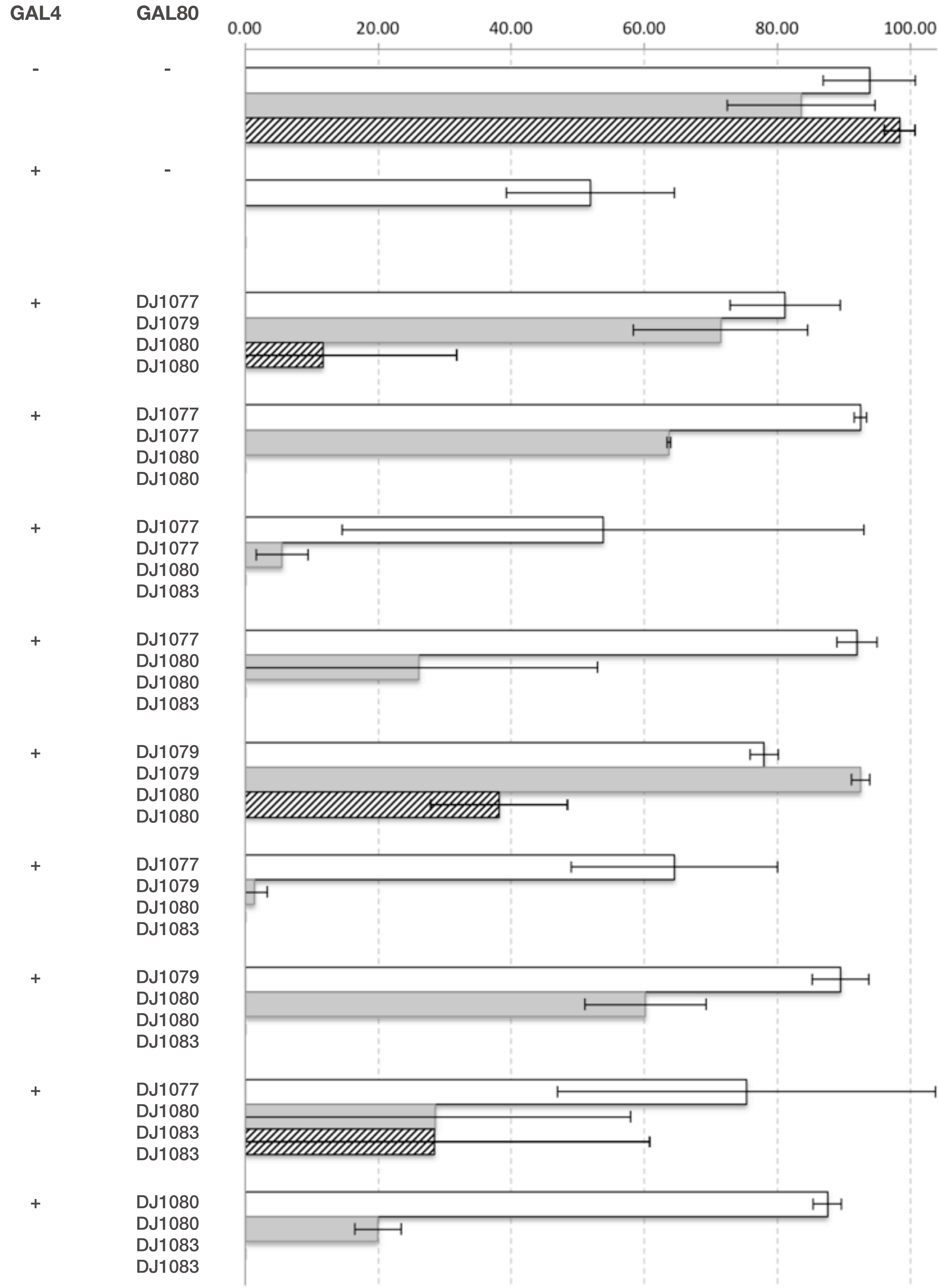

Supplemental Figure 28 Grim lethality assay of 4 copies of DJ146 insertions treated with ampicillin (30µg/ml) and tetracycline (50µg/ml). Horizontal axes shows average percent survival (% survival calculated as a % of the previous developmental stage). White bars indicate % of L1 larvae, grey bars indicate % of pupae, and striped bars indicate % of adults. Errror bars represent ±2SD. Vertical axes indicates genotype, all genotypes contain a UAS-grim transgene but differ by the presence or absence of GAL4 and GAL80 insertion. GAL4 column displays presence (+) or absence (-) of GAL4 in the genotype, and the GAL80 column indicates the DJ146 insertions or an absence (-) of GAL80.

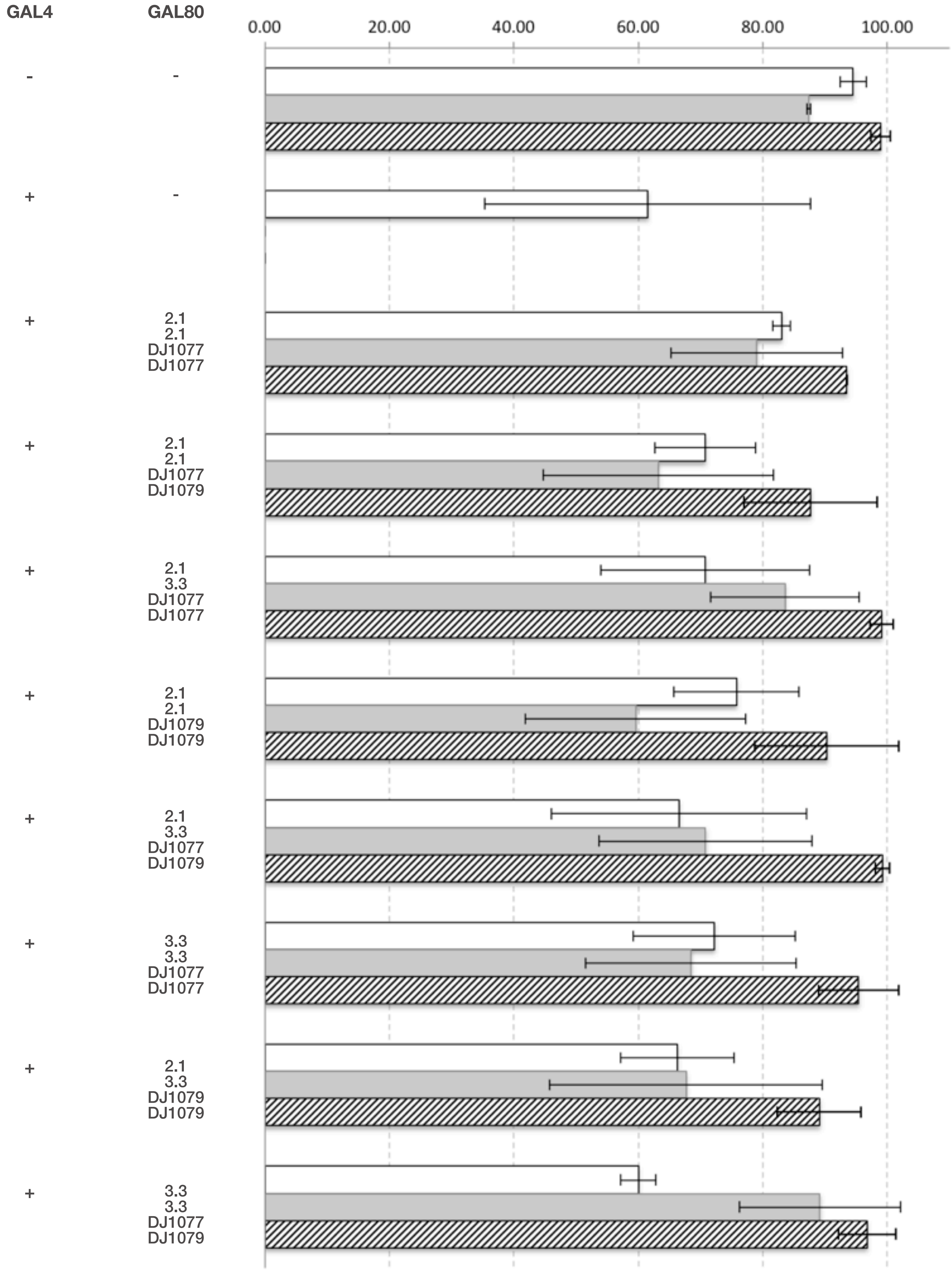

Supplemental Figure 29 Grim lethality assay of 2 copies of DJ147 insertions and 2 copies of DJ146 insertions treated with ampicillin (30µg/ml). Horizontal axes shows average percent survival (% survival calculated as a % of the previous developmental stage). White bars indicate % of L1 larvae, grey bars indicate % of pupae, and striped bars indicate % of adults. Errror bars represent ±2SD. Vertical axes indicates genotype, all genotypes contain a UAS-grim transgene but differ by the presence or absence of GAL4 and GAL80 insertion. GAL4 column displays presence (+) or absence (-) of GAL4 in the genotype, and the GAL80 column indicates the DJ147 and DJ146 insertions or an absence (-) of GAL80.

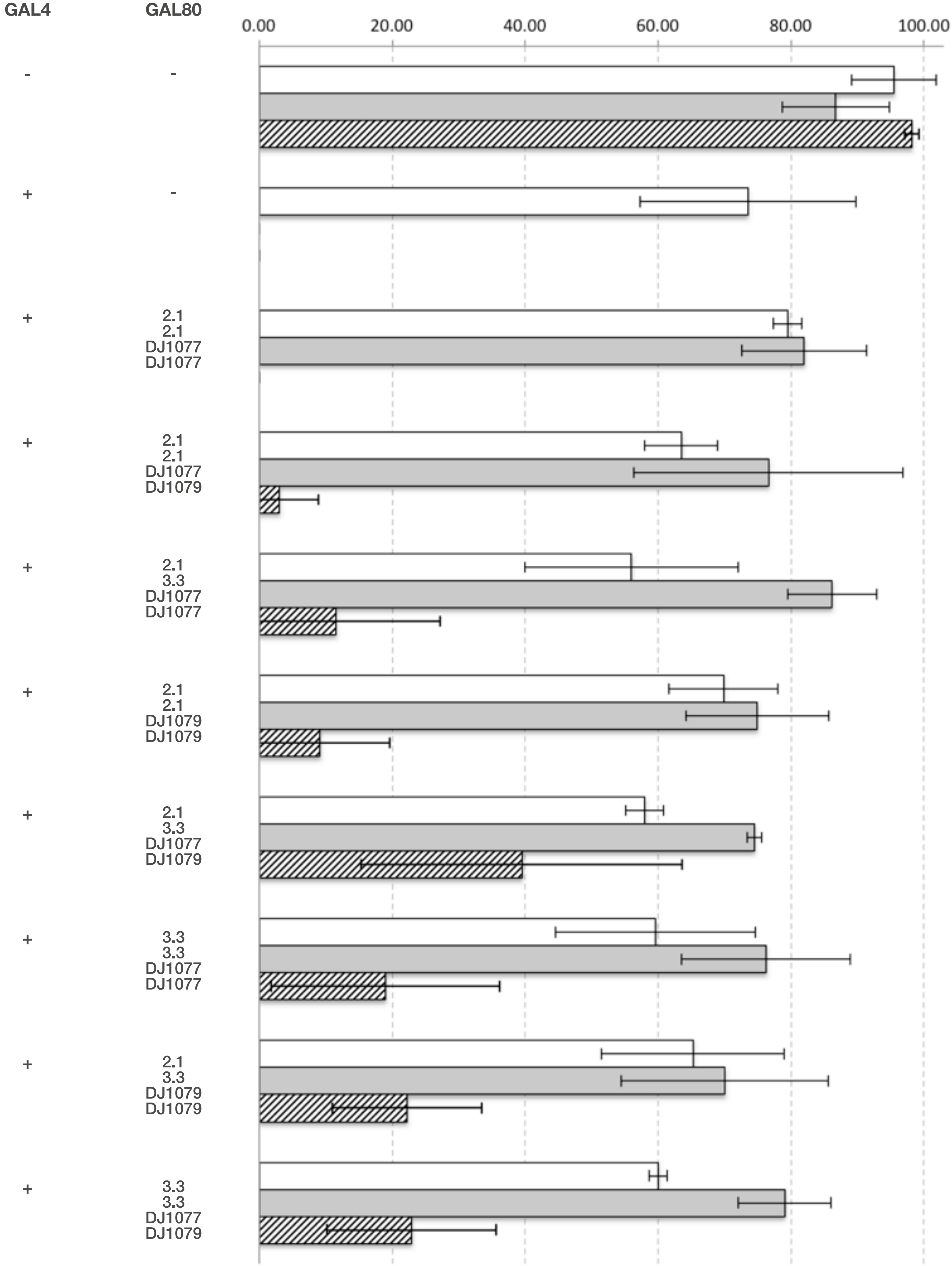

Supplemental Figure 30 Grim lethality assay of 2 copies of DJ147 insertions and 2 copies of DJ146 insertions treated with ampicillin (30μg/ml) and tetracycline (50μg/ml). Horizontal axes shows average percent survival (% survival calculated as a % of the previous developmental stage). White bars indicate % of L1 larvae, grey bars indicate % of pupae, and striped bars indicate % of adults. Errror bars represent ±2SD. Vertical axes indicates genotype, all genotypes contain a UAS-grim transgene but differ by the presence or absence of GAL4 and GAL80 insertion. GAL4 column displays presence (+) or absence (-) of GAL4 in the genotype, and the GAL80 column indicates the DJ147 and DJ146 insertions or an absence (-) of GAL80.

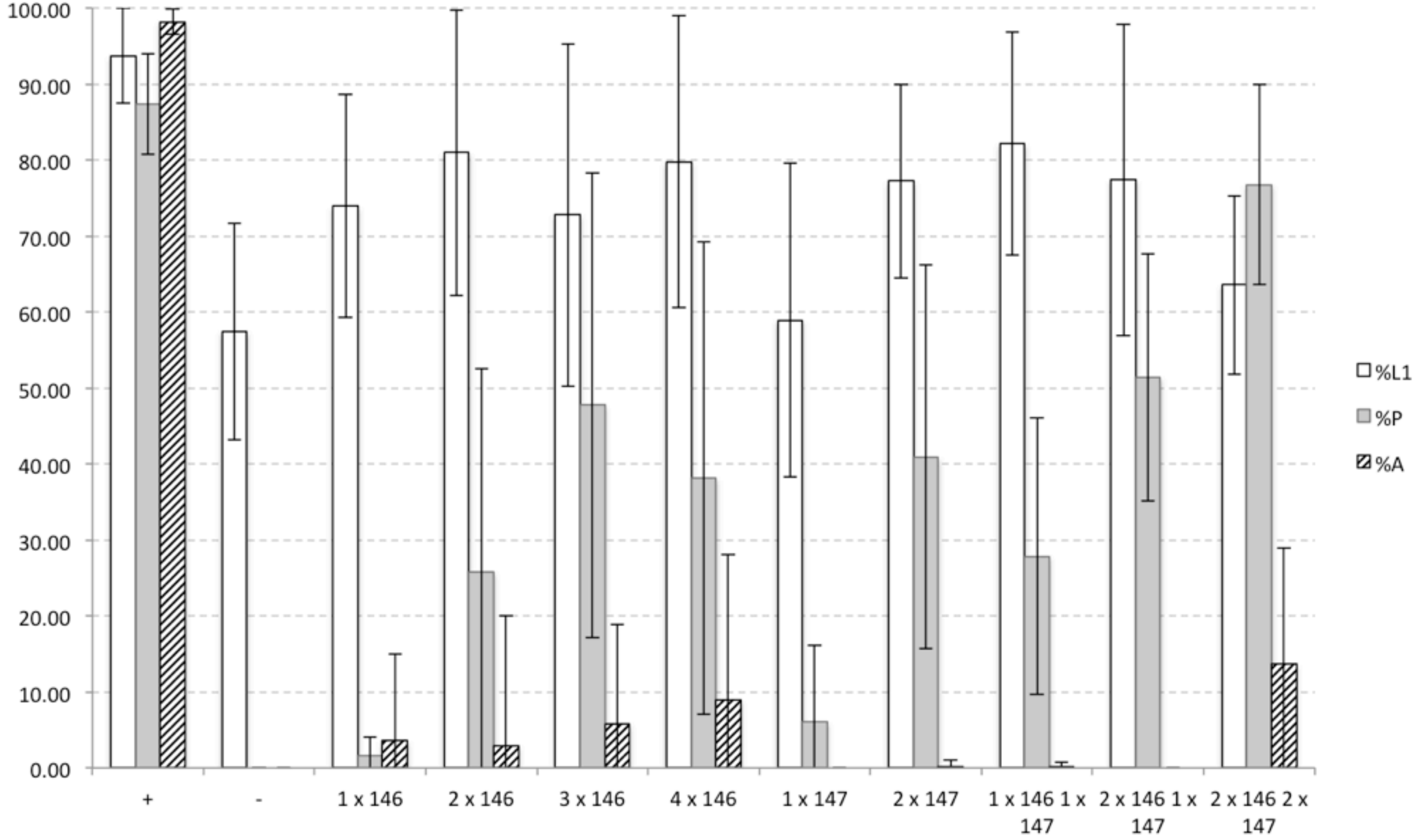

Supplemental Figure 31 Relief of repression of GAL4 transcriptional activity by GAL80, as measured by grim lethality assay in the presence of inducer. Individuals are treated with tetracycline (50μg/ml) and ampicillin (30μg/ml) throughout development. Vertical axis represents average percent survival. White bars indicate percent L1. Grey bars indicate percent pupae. Striped bars indicate percent adults. Positive control animals (+) carry the UAS-grim transgene in the absence of GAL4 driver (UAS-grim/+). Negative control animals (-) carry the UAS-grim transgene and the GAL4 driver (DJ694/UAS-grim). Experimental animals carry the UAS-grim transgene, the GAL4 driver and GAL80 transgene(s). Error bars denote ±2SD.

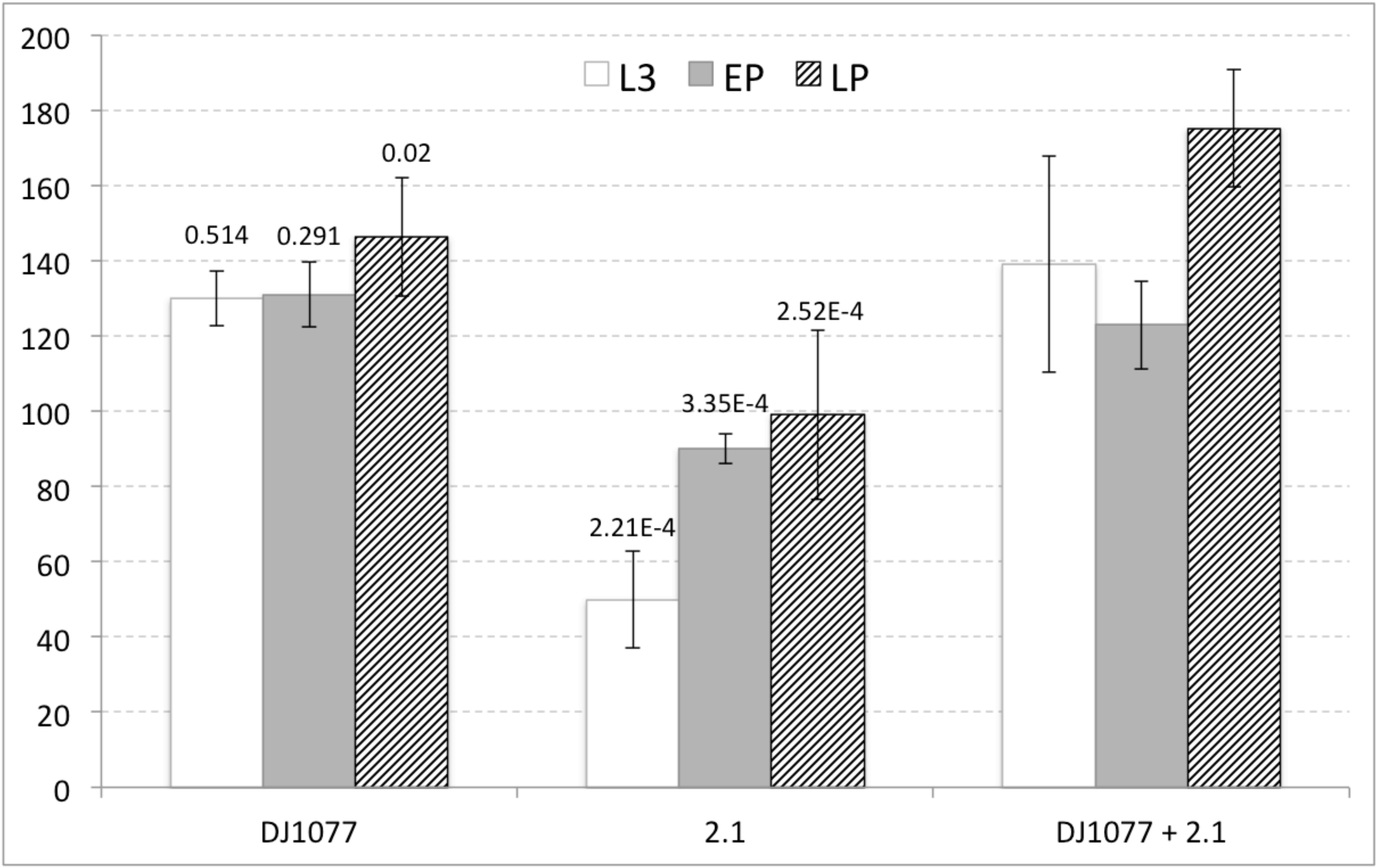

Supplemental Figure 32 DJ146 and DJ147 tTA transcriptional activity during development measured using a tetO-lacZ reporter. The y-axis indicates the lacZ encoded ß-galactosidase average specific activity (ΔmOD562nm/min/μg protein). The x-axis indicates genotypes with one copy DJ146 (DJ1077), one copy DJ147 (2.1) and one copy of each (DJ1077 + 2.1). White bars represent measurements of L3 larvae, grey bars represent the measurements of early pupae and striped bars represent measurements of late pupae. Error bars represent ±2SD (n=5 per genotype, per stage). T-test statistical analysis was performed between the one copy genotypes and the two copy genotype for each measured stage, the p-values are indicated above the corresponding one copy bar.

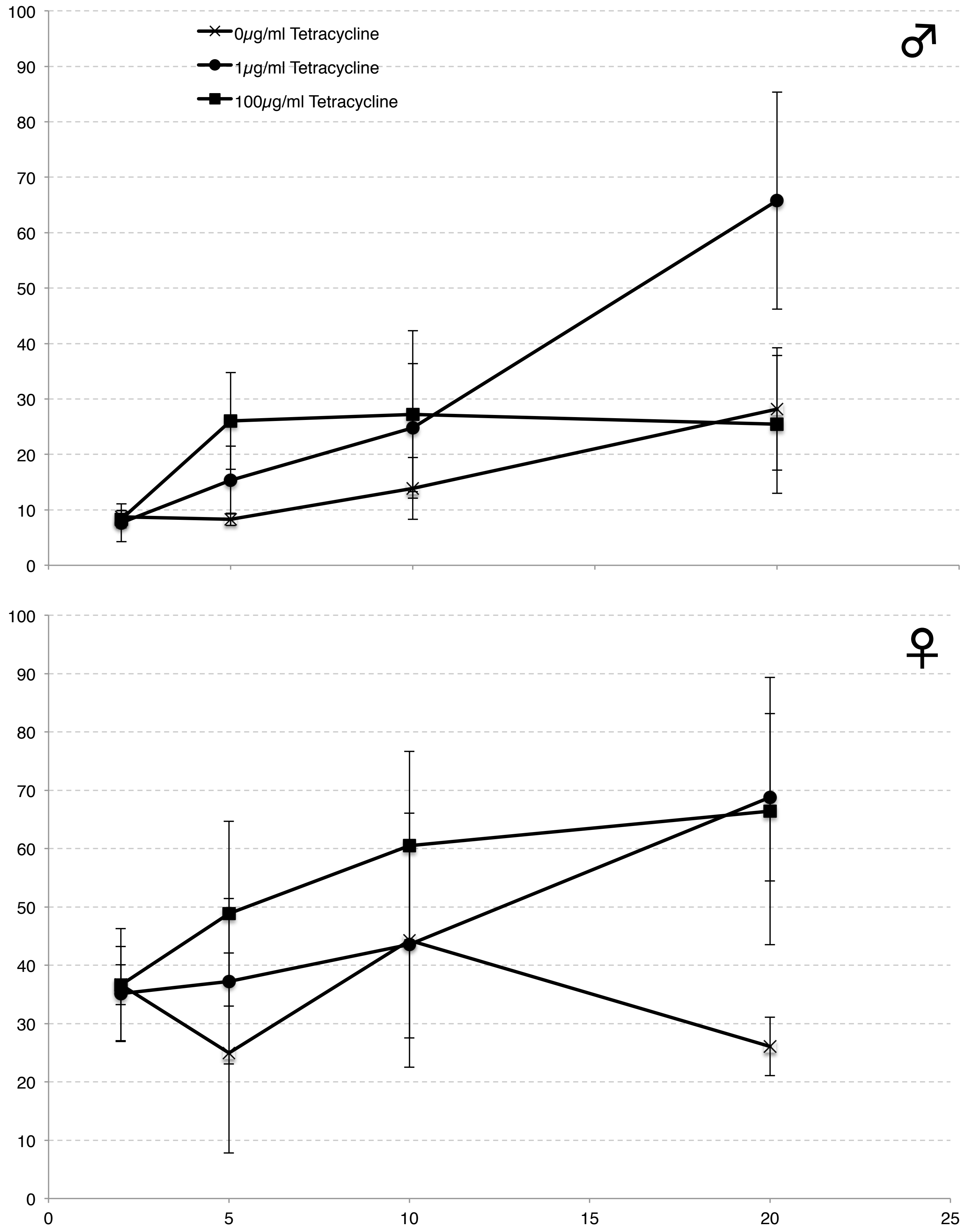

Supplemental Figure 33 GAL4 transcriptional activity in 2x146 + 2x147 flies that were fed tetracycline (0, 1, or 100µg/ml) starting at 20 days of age. The y-axis shows lacZ-encoded ß-galactosidase activity normalized as a percentage of the positive control. ß-galactosidase activity was measured as ΔmOD562nm per minute per fly. The x-axis shows the days on tetracycline (length of induction). Error bars represent ±SD (n=5 per tetracycline treatment per age).

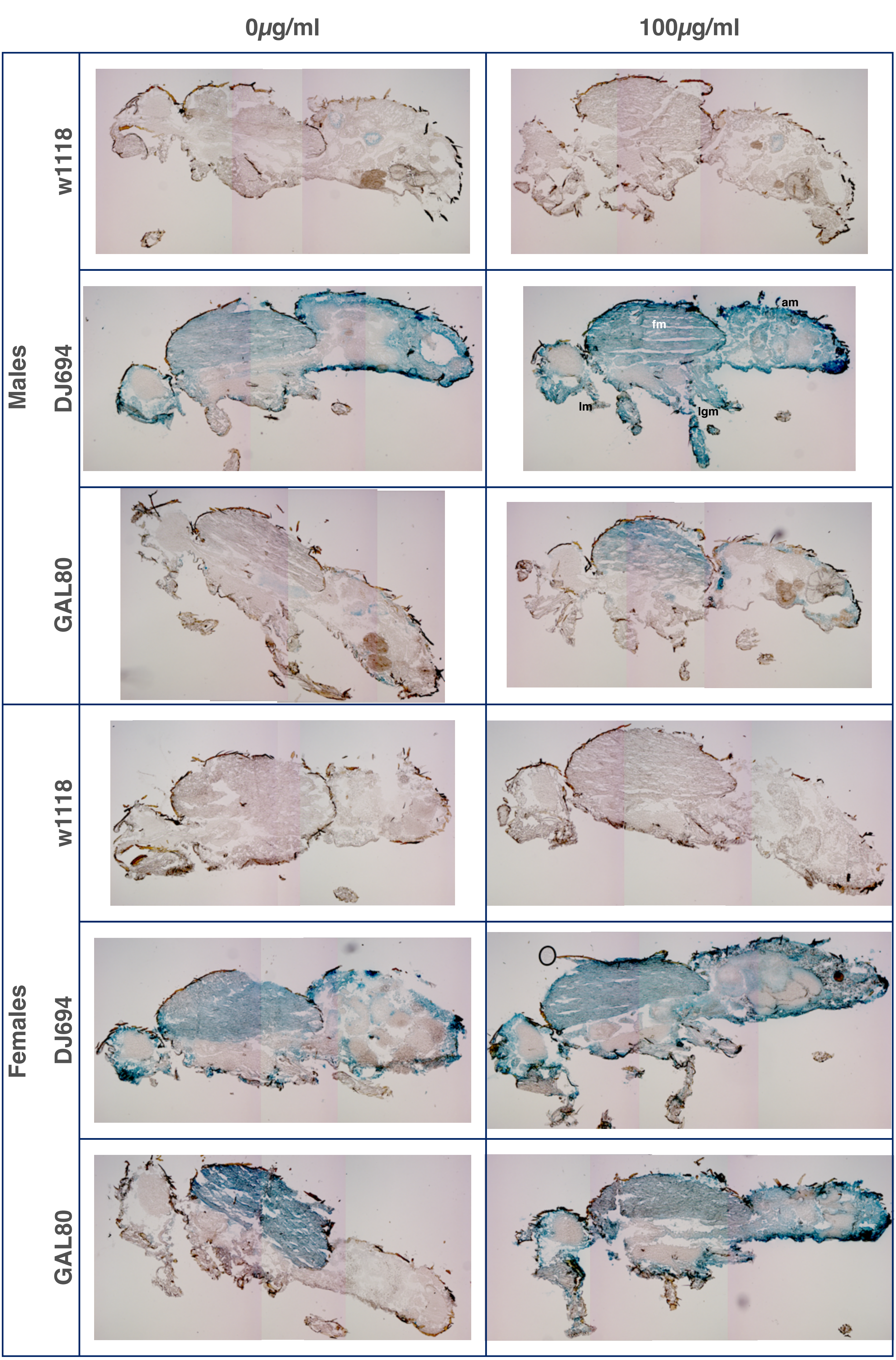

Supplemental Figure 34 Tissue distribution of lacZ expression in 30 days old GAL80 male and female flies fed 0 or 100μg/ml of tetracycline beginning at 20 days of age. w1118 refers to negative control flies (w1118/+;UAS-lacZ/+). DJ694 refers to positive control flies (DJ694/UAS-lacZ). GAL80 refers to experimental flies with four copies of GAL80 (2X146 + 2X147) (DJ694/UAS-lacZ;3.3,DJ1077). Several pictures of the same section were stitched together to generate whole-fly views. lm: labial muscle; fm: flight muscle; lgm: leg muscle; am: abdominal muscle wall.

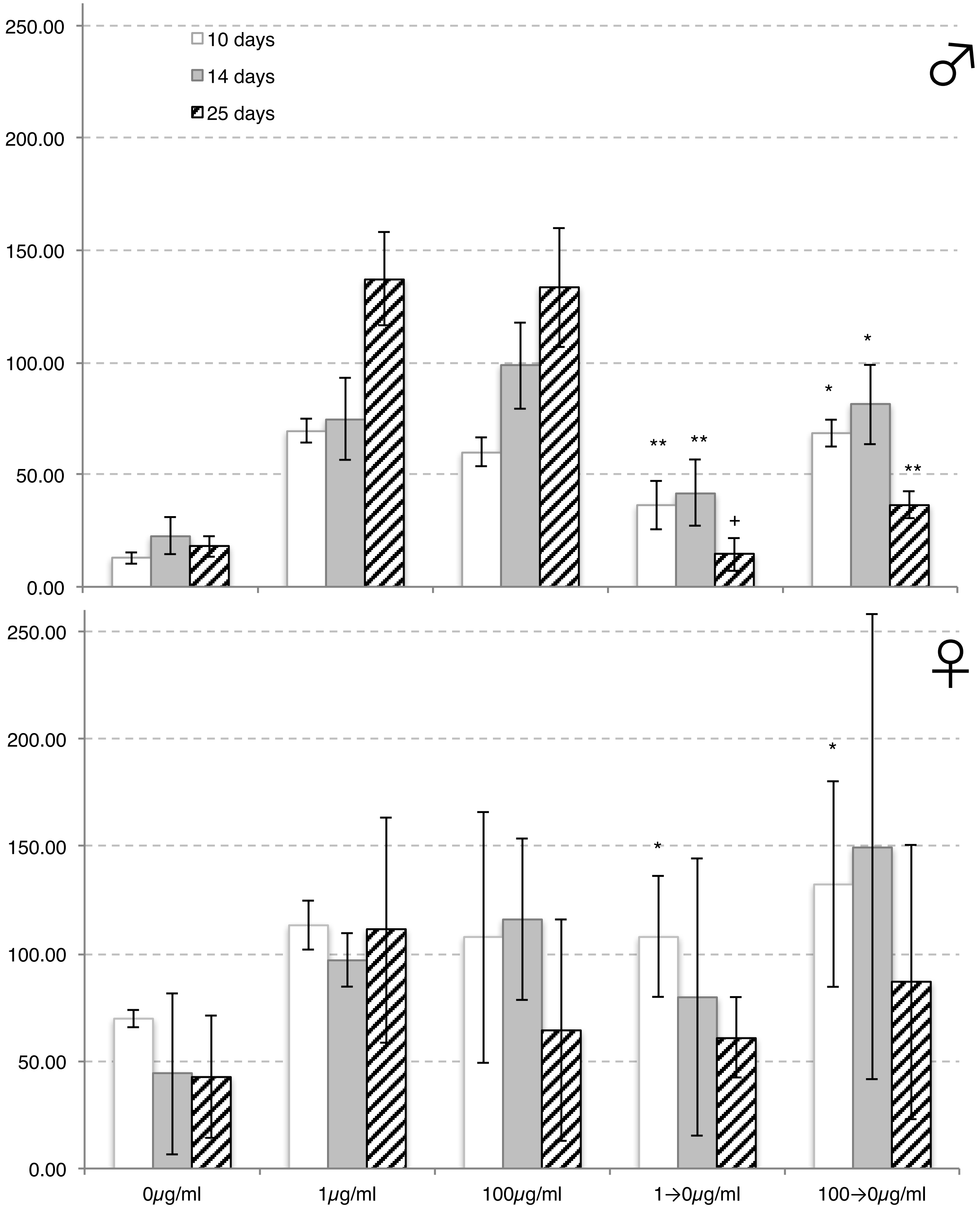

Supplemental Figure 35 Kinetics of reestablishing repression of GAL4 transcriptional activity. Negative controls (0μg/ml) were flies maintained on food containing no tetracycline for the duration of the experiment. Positive controls (1 and 100μg/ml) were flies maintained on food containing 1 and 100μg/ml of tetracycline for the duration of the experiment, repectively. Experimentals (1→0 and 100→0μg/ml) were positive control flies switched to food with no tetracycline at 5 days of age. GAL4 transcriptional activity was measured at 10, 14, and 25 days of age. The y-axis shows the lacZ-encoded ß-galactosidase activity normalized to the level seen with age and treatment matched DJ694 X UAS-lacZ flies. ß-galactosidase activity was measured as ΔmOD562nm/min/fly. *denotes significant difference ($p<0.05$) from the negative control; **denotes significance from both the positive and negative controls; + denotes significance from the positive control. Error bars represent ±SD (n=5 per tetracycline treatment per age).

Supplemental tables

Supplemental Table 1: UAS-grim lethality assay data set.

%: percentage of individuals remaining from the previous stage (average of at least 4 plates); SD: standard deviation.

Parents		Date	%L1	SD	%P	SD	%A	SD	%L1	SD	%P	SD	%A	SD
Male	Female		AMP						AMP/TET					
			**DJ694 + 0 GAL80**											
DJ694	UAS-grim	04/13/2005	58.5	11.1	0.0	0.0	0.0	0.0						
		04/20/2005	54.0	8.6	0.0	0.0	0.0	0.0						
		04/27/2005	42.0	20.3	0.0	0.0	0.0	0.0	80.5	5.8	0.0	0.0	0.0	0.0
		05/04/2005	68.0	5.2	0.0	0.0	0.0	0.0	73.5	10.0	0.0	0.0	0.0	0.0
		05/11/2005	68.5	11.0	0.7	1.9	0.0	0.0	52.0	6.8	0.0	0.0	0.0	0.0
		05/11/2005	66.0	12.0	0.0	0.0	0.0	0.0	57.0	3.8	0.0	0.0	0.0	0.0
		05/18/2005	29.5	6.8	0.0	0.0	0.0	0.0	25.0	27.6	0.0	0.0	0.0	0.0
		04/11/2006	72.0	8.8	0.0	0.0	0.0	0.0	73.5	8.0	0.0	0.0	0.0	0.0
		01/26/2006	45.0	4.8	0.0	0.0	0.0	0.0	44.5	4.7	0.0	0.0	0.0	0.0
		04/19/2006	59.0	6.3	0.0	0.0	0.0	0.0	79.5	7.5	0.0	0.0	0.0	0.0
		04/25/2006	62.0	9.1	0.0	0.0	0.0	0.0	58.5	9.1	0.0	0.0	0.0	0.0
		05/02/2006	57.5	9.1	0.0	0.0	0.0	0.0	69.5	8.3	0.0	0.0	0.0	0.0
		05/09/2006	36.5	8.9	0.0	0.0	0.0	0.0	56.0	9.3	0.0	0.0	0.0	0.0
		05/16/2006	49.5	11.3	0.0	0.0	0.0	0.0	61.5	8.5	0.0	0.0	0.0	0.0
		05/24/2006	35.5	8.7	0.0	0.0	0.0	0.0	47.5	9.2	0.0	0.0	0.0	0.0
		05/30/2006	50.5	9.8	0.0	0.0	0.0	0.0	51.5	7.8	0.0	0.0	0.0	0.0
		06/06/2006	24.5	7.2	0.0	0.0	0.0	0.0	47.5	11.0	0.0	0.0	0.0	0.0
		06/13/2006	24.0	11.1	0.0	0.0	0.0	0.0	32.0	3.0	0.0	0.0	0.0	0.0
		06/20/2006	69.0	8.5	0.0	0.0	0.0	0.0	62.5	7.4	0.0	0.0	0.0	0.0
		09/26/2006	61.0	17.3	0.0	0.0	0.0	0.0	47.5	13.3	0.0	0.0	0.0	0.0
		10/05/2006	36.0	10.3	0.0	0.0	0.0	0.0	38.0	10.6	0.0	0.0	0.0	0.0
		10/12/2006	36.0	10.3	0.0	0.0	0.0	0.0	38.0	10.6	0.0	0.0	0.0	0.0
		10/19/2006	37.0	6.0	0.0	0.0	0.0	0.0	52.0	5.7	0.0	0.0	0.0	0.0
		10/19/2006	37.0	3.0	0.0	0.0	0.0	0.0	52.0	2.8	0.0	0.0	0.0	0.0
		10/23/2006	73.0	13.2	0.0	0.0	0.0	0.0	63.0	3.8	0.0	0.0	0.0	0.0
		10/23/2006	73.0	6.6	0.0	0.0	0.0	0.0	63.0	1.9	0.0	0.0	0.0	0.0
		11/02/2006	34.0	14.0	0.0	0.0	0.0	0.0	57.0	12.8	0.0	0.0	0.0	0.0
		11/02/2006	34.0	7.0	0.0	0.0	0.0	0.0	57.0	6.4	0.0	0.0	0.0	0.0
		11/16/2006	61.0	5.7	0.0	0.0	0.0	0.0	63.0	1.0	0.0	0.0	0.0	0.0
		11/23/2006	42.0	4.8	0.0	0.0	0.0	0.0	51.0	6.0	0.0	0.0	0.0	0.0
		12/07/2006	88.0	3.3	0.0	0.0	0.0	0.0	89.0	2.5	0.0	0.0	0.0	0.0
		12/14/2006	63.0	2.5	0.0	0.0	0.0	0.0	56.0	4.9	0.0	0.0	0.0	0.0
		12/14/2006	44.0	3.7	0.0	0.0	0.0	0.0	51.0	3.4	0.0	0.0	0.0	0.0
		05/18/2005	60.5	6.9	0.0	0.0	0.0	0.0	55.5	9.4	0.0	0.0	0.0	0.0
		11/27/2007	80.0	3.3	0.0	0.0	0.0	0.0	85.0	6.0	0.0	0.0	0.0	0.0
		12/18/2007	43.0	6.8	0.0	0.0	0.0	0.0	62.0	14.8	0.0	0.0	0.0	0.0
			52.1	3.9	0.0	0.3	0.0	0.0	57.4	4.9	0.0	0.0	0.0	0.0
			**DJ1077**											
w	UAS-grim;DJ1077	04/13/2005	93.5	3.7	73.3	10.9	98.8	9.8						
		05/11/2005	93.3	7.0	90.0	6.0	97.5	2.7	91.6	8.6	84.5	7.6	100.0	0.0
			93.4	0.1	81.6	11.8	98.2	0.9	91.6	8.6	84.5	7.6	100.0	0.0
			**DJ1078**											
w	UAS-grim;DJ1078	04/13/2005	96.5	3.3	88.5	9.5	99.5	1.4						
		05/11/2005	87.5	9.7	88.6	8.3	100.0	0.0	91.6	8.6	91.6	8.7	98.9	2.1
			92.0	6.4	88.5	0.1	99.7	0.4	91.6	8.6	91.6	8.7	98.9	2.1
			**DJ1079**											
w	UAS-grim;DJ1079	04/13/2005	90.5	5.6	90.7	4.7	95.9	6.2						
		05/11/2005	86.1	7.5	93.9	5.9	99.4	1.6	93.0	7.9	87.8	7.1	98.8	2.2
			88.3	3.1	92.3	2.3	97.7	2.5	93.0	7.9	87.8	7.1	98.8	2.2
			**DJ1080**											
w	UAS-grim;DJ1080	04/20/2005	96.5	2.6	90.7	4.9	95.2	5.6						
		05/11/2005	90.5	9.5	84.3	4.9	97.5	3.9	93.5	4.8	91.4	4.1	98.3	2.3
			93.5	4.2	87.5	4.5	96.4	1.6	93.5	4.8	91.4	4.1	98.3	2.3
			**DJ1081**											
w	UAS-grim;DJ1081	04/20/2005	95.5	5.4	78.4	5.8	99.4	1.7						
		05/11/2005	97.0	2.8	91.3	3.3	96.1	3.8	94.5	3.7	93.1	3.4	97.1	3.4
			96.3	1.1	84.9	9.1	97.7	2.4	94.5	3.7	93.1	3.4	97.1	3.4
			**DJ1083**											
w	UAS-grim;DJ1083	04/20/2005	97.0	2.8	91.3	7.1	97.3	2.3						
		05/11/2005	84.0	6.0	91.6	5.2	95.2	6.1	87.5	5.4	93.3	7.0	97.0	3.5
			90.5	9.2	91.5	0.3	96.2	1.5	87.5	5.4	93.3	7.0	97.0	3.5

Parents		Date	%L1	SD	%P	SD	%A	SD	%L1	SD	%P	SD	%A	SD
Male	Female		AMP						AMP/TET					
			**DJ694 + DJ1077**											
DJ694	UAS-grim;DJ1077	04/27/2005	35.5	14.9	40.8	34.7	0.0	0.0	75.5	5.0	0.0	0.0	0.0	0.0
		04/11/2006	66.0	6.0	61.3	7.5	0.0	0.0	69.5	2.8	0.0	0.0	0.0	0.0
		05/18/2005	59.3	9.9	64.5	10.6	0.0	0.0	52.0	8.6	0.8	2.2	12.5	35.4
DJ694;DJ1077	UAS-grim	01/26/2006	79.5	3.8	46.5	3.4	1.1	1.6	39.5	4.5	0.0	0.0	0.0	0.0
		05/25/2005	89.0	6.0	86.8	7.8	8.8	6.5	93.5	3.7	3.3	3.1	0.0	0.0
			65.9	20.5	60.0	18.0	2.0	3.8	66.0	21.0	0.8	1.4	2.5	5.6
			**DJ694 + DJ1078**											
DJ694	UAS-grim;DJ1078	04/27/2005	37.5	6.7	18.7	17.0	0.0	0.0	64.5	9.7	5.3	6.8	0.0	0.0
		05/18/2005	69.5	9.5	43.1	4.6	0.0	0.0	78.7	7.3	7.1	7.8	44.8	43.4
DJ694;DJ1078	UAS-grim	05/25/2005	82.5	6.0	70.4	14.3	5.0	8.4	90.0	6.8	2.8	2.4	0.0	0.0
			63.2	23.2	44.0	25.8	1.7	2.9	77.7	12.8	5.1	2.1	14.9	25.9
			**DJ694 + DJ1079**											
DJ694	UAS-grim;DJ1079	04/27/2005	51.5	9.7	2.5	7.1	0.0	0.0	77.5	10.5	0.0	0.0	0.0	0.0
		05/18/2005	70.5	4.2	6.6	6.2	0.0	0.0	69.0	6.7	0.0	0.0	0.0	0.0
DJ694;DJ1079	UAS-grim	05/25/2005	91.5	6.2	85.4	8.0	0.0	0.0	95.0	4.7	5.7	4.4	0.0	0.0
			71.2	20.0	31.5	46.7	0.0	0.0	80.5	13.3	1.9	3.3	0.0	0.0
			**DJ694 + DJ1080**											
DJ694	UAS-grim;DJ1080	05/04/2005	73.5	7.7	17.0	11.5	0.0	0.0	74.0	11.7	0.0	0.0	0.0	0.0
		05/18/2005	71.5	10.8	46.0	14.7	0.0	0.0	65.0	10.2	0.0	0.0	0.0	0.0
DJ694;DJ1080	UAS-grim	05/25/2005	89.5	6.4	70.2	10.4	0.0	0.0	87.5	4.5	0.6	1.6	0.0	0.0
			78.2	9.9	44.4	26.6	0.0	0.0	75.5	11.3	0.2	0.3	0.0	0.0
			**DJ694 + DJ1081**											
DJ694	UAS-grim;DJ1081	05/04/2005	75.5	8.9	20.1	6.9	72.5	25.5	74.0	6.0	12.3	4.1	100.0	0.0
		05/18/2005	45.5	9.1	31.5	18.1	35.8	26.1	47.5	11.0	7.3	8.0	62.5	51.8
			60.5	21.2	25.8	8.1	54.2	25.9	60.8	18.7	9.8	3.5	81.3	26.5
			**DJ694 + DJ1083**											
DJ694	UAS-grim;DJ1083	05/04/2005	76.0	6.8	15.7	12.5	0.0	0.0	71.5	11.4	0.0	0.0	0.0	0.0
		05/18/2005	76.5	7.2	69.2	11.1	0.0	0.0	80.5	9.4	0.0	0.0	0.0	0.0
			76.3	0.4	42.5	37.8	0.0	0.0	76.0	6.4	0.0	0.0	0.0	0.0
			**DJ694 + 2 copies DJ1077**											
DJ694;DJ1077	UAS-grim;DJ1077	06/01/2005	90.5	7.1	74.7	6.2	0.0	0.0	83.5	8.1	75.7	8.4	0.0	0.0
		04/11/2006	78.5	4.5	79.4	6.4	78.8	5.4	84.0	4.0	66.7	3.7	0.0	0.0
		01/26/2006	85.0	4.2	58.3	6.2	49.9	10.7	13.0	3.5	0.0	0.0	0.0	0.0
			84.7	6.0	70.8	11.1	42.9	39.9	60.2	40.8	47.4	41.3	0.0	0.0
			**DJ694 + DJ1077 + DJ1078**											
DJ694;DJ1078	UAS-grim;DJ1077	06/01/2005	98.5	2.1	69.1	9.9	0.9	2.5	97.0	3.5	54.5	16.0	0.0	0.0
DJ694;DJ1077	UAS-grim;DJ1078	06/01/2005	93.5	6.4	75.7	16.7	0.0	0.0	88.0	5.7	58.4	11.2	0.0	0.0
			96.0	3.5	72.4	4.6	0.5	0.6	92.5	6.4	56.5	2.8	0.0	0.0
			**DJ694 + 2 copies DJ1078**											
DJ694;DJ1078	UAS-grim;DJ1078	06/01/2005	97.5	4.8	67.9	11.0	0.0	0.0	89.0	6.3	12.1	9.9	0.0	0.0
			97.5	4.8	67.9	11.0	0.0	0.0	89.0	6.3	12.1	9.9	0.0	0.0
			**DJ694 + DJ1077 + DJ1079**											
DJ694;DJ1077	UAS-grim;DJ1079	06/08/2005	95.5	2.6	46.3	26.3	0.0	0.0	89.0	6.0	4.2	6.8	0.0	0.0
DJ694;DJ1079	UAS-grim;DJ1077	06/15/2005	98.5	3.0	77.2	5.5	0.0	0.0	98.0	3.0	67.3	11.9	0.0	0.0
		04/11/2006	75.5	4.9	80.1	8.4	77.6	5.9	81.5	3.5	76.4	6.6	0.0	0.0
			89.8	12.5	67.9	18.7	25.9	44.8	89.5	8.3	49.3	39.3	0.0	0.0
			**DJ694 + DJ1078 + DJ1079**											
DJ694;DJ1078	UAS-grim;DJ1079	06/08/2005	89.0	4.1	26.0	22.0	0.0	0.0	84.5	5.0	0.6	1.7	0.0	0.0
DJ694;DJ1079	UAS-grim;DJ1078	06/15/2005	97.5	3.7	69.0	13.1	0.0	0.0	98.0	2.1	43.0	15.2	0.0	0.0
			93.3	6.0	47.5	30.4	0.0	0.0	91.3	9.5	21.8	30.0	0.0	0.0
			**DJ694 + DJ1077 + DJ1080**											
DJ694;DJ1077	UAS-grim;DJ1080	06/08/2005	97.5	3.0	75.0	7.1	0.0	0.0	90.0	8.0	25.0	8.6	0.0	0.0
DJ694;DJ1080	UAS-grim;DJ1077	06/15/2005	97.0	3.5	71.9	8.7	0.0	0.0	91.5	4.0	58.5	11.4	0.0	0.0
		04/11/2006	92.0	1.9	68.5	3.0	52.6	6.3	91.5	3.6	22.8	2.8	0.0	0.0
UAS-grim;DJ1077	DJ694;DJ1080	01/26/2006	93.5	2.4	73.6	6.7	25.2	9.5	89.0	2.3	57.8	3.3	0.0	0.0
UAS-grim;DJ1080	DJ694;DJ1077	01/26/2006	40.0	2.6	32.8	4.2	27.5	18.8	65.5	5.3	49.5	3.0	0.0	0.0
DJ694	UAS-grim; DJ1077+DJ1080	05/30/2006	69.0	2.6	55.4	6.2	0.0	0.0	69.0	5.8	0.0	0.0	0.0	0.0
			81.5	22.9	62.9	16.3	17.6	21.5	82.8	12.1	35.6	23.5	0.0	0.0

Parents		Date	%L1	SD	%P	SD	%A	SD	%L1	SD	%P	SD	%A	SD
Male	Female		AMP						AMP/TET					
			**DJ694 + DJ1078 + DJ1080**											
DJ694;DJ1078	UAS-grim;DJ1080	06/08/2005	98.5	2.1	75.3	14.0	0.0	0.0	88.5	9.2	3.9	4.7	0.0	0.0
DJ694;DJ1080	UAS-grim;DJ1078	06/15/2005	95.5	5.4	58.8	13.8	0.0	0.0	93.0	5.6	28.0	11.5	0.0	0.0
			97.0	2.1	67.0	11.6	0.0	0.0	90.8	3.2	16.0	17.0	0.0	0.0
			**DJ694 + DJ1077 + DJ1081**											
DJ694;DJ1077	UAS-grim;DJ1081	06/15/2005	87.5	5.4	42.7	17.8	79.1	15.5	89.5	5.2	31.9	8.6	98.4	4.4
			87.5	5.4	42.7	17.8	79.1	15.5	89.5	5.2	31.9	8.6	98.4	4.4
			**DJ694 + DJ1078 + DJ1081**											
DJ694;DJ1078	UAS-grim;DJ1081	06/15/2005	94.0	3.0	44.2	15.1	49.1	10.7	92.5	6.2	13.6	4.8	83.4	25.2
			94.0	3.0	44.2	15.1	49.1	10.7	92.5	6.2	13.6	4.8	83.4	25.2
			**DJ694 + DJ1077 + DJ1083**											
DJ694;DJ1077	UAS-grim;DJ1083	06/15/2005	94.0	3.7	72.5	9.4	0.0	0.0	92.0	4.3	1.1	2.0	0.0	0.0
UAS-grim;DJ1083	DJ694;DJ1077	01/26/2006	57.0	6.7	71.2	9.3	6.2	4.4	55.5	3.1	1.1	1.6	0.0	0.0
DJ694	UAS-grim;DJ1077+DJ1083	04/25/2006	28.0	10.4	21.6	7.1	4.2	5.9	28.0	4.8	0.0	0.0	0.0	0.0
	UAS-grim;DJ1077+DJ1083	05/30/2006	45.0	7.8	17.0	6.1	37.5	25.9	74.5	8.7	5.1	2.2	0.0	0.0
			56.0	28.0	45.6	30.4	12.0	17.2	62.5	27.4	1.8	2.2	0.0	0.0
			**DJ694 + DJ1078 + DJ1083**											
DJ694;DJ1078	UAS-grim;DJ1083	06/15/2005	87.5	5.8	47.5	23.5	0.0	0.0	91.0	6.3	0.0	0.0	0.0	0.0
			87.5	5.8	47.5	23.5	0.0	0.0	91.0	6.3	0.0	0.0	0.0	0.0
			**DJ694 + 2 copies DJ1079**											
DJ694;DJ1079	UAS-grim;DJ1079	06/01/2005	87.0	6.0	78.8	10.0	0.0	0.0	92.0	5.7	21.4	13.2	0.0	0.0
			87.0	6.0	78.8	10.0	0.0	0.0	92.0	5.7	21.4	13.2	0.0	0.0
			**DJ694 + DJ1079 + DJ1080**											
DJ694;DJ1080	UAS-grim;DJ1079	06/01/2005	93.5	4.2	84.4	7.6	0.0	0.0	85.0	6.0	61.5	8.8	0.0	0.0
DJ694;DJ1079	UAS-grim;DJ1080	06/01/2005	94.0	6.8	60.6	7.5	0.0	0.0	91.0	5.6	3.3	3.8	0.0	0.0
DJ694	UAS-grim;DJ1079+DJ1080	05/02/2006	88.0	3.9	64.9	10.6	41.8	6.8	72.0	2.6	27.1	5.8	100.0	0.0
			91.8	3.3	70.0	12.7	13.9	24.1	82.7	9.7	30.6	29.2	33.3	57.7
			**DJ694 + 2 copies DJ1080**											
DJ694;DJ1080	UAS-grim;DJ1080	06/01/2005	91.0	4.1	81.3	3.4	0.0	0.0	86.0	9.8	23.0	14.5	0.0	0.0
UAS-grim;DJ1080	DJ694;DJ1080	01/26/2006	83.0	4.9	74.9	8.7	6.6	3.1	85.0	2.6	25.8	6.0	0.0	0.0
			87.0	5.7	78.1	4.6	3.3	4.6	85.5	0.7	24.4	2.0	0.0	0.0
			**DJ694 + DJ1079 + DJ1081**											
DJ694;DJ1079	UAS-grim;DJ1081	06/08/2005	93.0	6.0	69.9	11.7	43.6	20.2	95.0	2.8	29.0	9.4	85.6	20.9
			93.0	6.0	69.9	11.7	43.6	20.2	95.0	2.8	29.0	9.4	85.6	20.9
			**DJ694 + DJ1080 + DJ1081**											
DJ694;DJ1080	UAS-grim;DJ1081	06/08/2005	85.0	11.5	32.5	16.7	60.3	29.0	95.0	4.7	31.9	11.3	100.0	0.0
			85.0	11.5	32.5	16.7	60.3	29.0	95.0	4.7	31.9	11.3	100.0	0.0
			**DJ694 + DJ1079 + DJ1083**											
DJ694;DJ1079	UAS-grim;DJ1083	06/08/2005	93.0	5.1	69.8	10.4	0.0	0.0	92.0	6.4	3.6	7.3	0.0	0.0
			93.0	5.1	69.8	10.4	0.0	0.0	92.0	6.4	3.6	7.3	0.0	0.0
			**DJ694 + DJ1080 + DJ1083**											
DJ694;DJ1080	UAS-grim;DJ1083	06/08/2005	82.5	4.2	50.8	20.2	0.0	0.0	93.5	5.2	0.0	0.0	0.0	0.0
UAS-grim;DJ1083	DJ694;DJ1080	01/26/2006	12.0	2.8	0.0	0.0	0.0	0.0	36.0	8.8	0.0	0.0	0.0	0.0
DJ694	UAS-grim;DJ1080+DJ1083	05/02/2006	56.5	7.8	60.9	10.0	0.0	0.0	54.0	6.9	0.0	0.0	0.0	0.0
		06/06/2006	62.0	8.4	65.2	6.1	7.5	3.5	77.5	7.6	0.0	0.0	0.0	0.0
			53.3	29.7	44.2	30.1	1.9	3.7	65.3	25.4	0.0	0.0	0.0	0.0
			**DJ1077 + DJ1080**											
UAS-grim; DJ1077+DJ1080	w	04/19/2006	87.0	1.8	90.2	4.1	100.0	0.0	82.5	5.0	94.9	0.1	100.0	0.0
		05/24/2006	99.0	0.9	74.1	7.2	99.4	0.9	86.0	5.3	79.8	0.6	99.3	2.1
DJ694; DJ1077+DJ1080	w	04/19/2006	87.0	2.6	82.4	5.2	99.4	0.9	84.0	4.0	84.4	3.6	98.7	1.3
		05/24/2006	93.0	2.8	90.3	2.3	98.2	1.3	93.0	2.3	96.8	1.0	99.4	0.8
			91.5	5.7	84.3	7.7	99.2	0.8	86.4	4.6	89.0	8.2	99.3	0.6
			**DJ1077 + DJ1083**											
UAS-grim;DJ1077+DJ1083	w	04/19/2006	89.0	3.5	90.7	4.1	98.9	1.6	89.0	3.7	94.5	2.7	98.9	1.0
		05/24/2006	96.5	1.7	76.1	4.8	99.4	0.8	92.0	3.4	86.2	4.8	98.2	1.3
DJ694;DJ1077+DJ1083	w	04/19/2006	92.5	2.0	90.9	1.3	100.0	0.0	90.5	3.4	95.4	2.2	99.5	0.7
		05/24/2006	94.0	1.9	96.3	1.8	97.3	1.1	95.5	1.7	95.3	2.1	97.3	1.6
			93.0	3.1	88.5	8.6	98.9	1.2	91.8	2.8	92.9	4.5	98.5	0.9

Parents: Male	Parents: Female	Date	AMP %L1	AMP SD	AMP %P	AMP SD	AMP %A	AMP SD	AMP/TET %L1	AMP/TET SD	AMP/TET %P	AMP/TET SD	AMP/TET %A	AMP/TET SD
**DJ1080 + DJ1083**														
UAS-grim;DJ1080+DJ1083	w	05/24/2006	94.0	3.4	53.0	6.5	100.0	0.0	88.5	2.3	59.5	3.8	97.0	3.0
		04/19/2006	74.5	5.3	88.2	3.9	100.0	0.0	65.0	5.2	91.4	2.0	99.2	1.2
DJ694;DJ1080+DJ1083	w	04/19/2006	90.0	2.6	88.1	4.5	99.3	1.0	87.0	3.0	83.9	3.5	98.0	1.4
			86.2	10.3	76.4	20.3	99.8	0.4	80.2	13.2	78.2	16.7	98.1	1.1
**DJ1079 + DJ1080**														
UAS-grim;DJ1079+DJ1080	w	04/19/2006	88.5	4.6	92.9	1.9	99.3	1.0	92.5	2.9	88.7	2.5	98.2	1.3
		05/24/2006	99.5	0.7	79.9	3.5	99.4	0.9	97.5	1.0	80.6	4.9	99.5	0.7
DJ694;DJ1079+DJ1080	w	04/19/2006	86.0	3.4	84.2	4.5	100.0	0.0	87.0	3.8	90.3	4.8	98.1	1.9
		05/24/2006	91.5	2.9	93.8	3.8	96.7	3.3	96.0	1.1	94.3	3.1	98.9	1.0
			91.4	5.9	87.7	6.8	98.8	1.5	93.3	4.7	88.5	5.8	98.7	0.7
**DJ694 + 2 copies DJ1077 + DJ1080**														
DJ694;DJ1077	UAS-grim; DJ1077+DJ1080	04/25/2006	93.5	1.5	82.9	2.5	62.9	5.2	90.0	3.5	79.9	5.7	0.0	0.0
		05/30/2006	79.5	4.9	74.1	5.4	20.4	8.0	92.0	1.5	56.7	9.0	0.0	0.0
			86.5	9.9	78.5	6.2	41.6	30.1	91.0	1.4	68.3	16.4	0.0	0.0
**DJ694 + DJ1077 + DJ1079 + DJ1080**														
DJ694;DJ1079	UAS-grim; DJ1077+DJ1080	04/25/2006	76.0	2.1	77.2	6.1	77.4	5.4	79.5	3.6	58.3	7.4	0.0	0.0
		05/30/2006	82.0	2.4	69.7	8.8	31.4	8.5	89.0	4.0	67.5	6.2	0.0	0.0
DJ694;DJ1077	UAS-grim;DJ1079+DJ1080	05/02/2006	72.5	3.3	67.4	4.7	84.0	5.3	78.5	3.0	77.1	4.4	38.1	6.5
			76.8	4.8	71.4	5.1	64.3	28.6	82.3	5.8	67.6	9.4	12.7	22.0
**DJ694 + DJ1077 + 2 copies DJ1080**														
DJ694;DJ1080	UAS-grim; DJ1077+DJ1080	04/25/2006	90.5	3.2	89.8	3.5	68.4	3.8	87.0	3.2	79.6	5.2	0.0	0.0
		05/30/2006	91.0	2.6	80.0	5.8	29.0	7.0	88.5	3.1	44.6	7.2	0.0	0.0
			90.8	0.4	84.9	6.9	48.7	27.9	87.8	1.1	62.1	24.8	0.0	0.0
**DJ694 + 2 copies DJ1077 + DJ1083**														
DJ694;DJ1077	UAS-grim;DJ1077+DJ1083	04/25/2006	45.5	5.3	43.7	6.5	73.8	9.1	42.0	8.6	10.8	4.6	0.0	0.0
		05/30/2006	54.0	5.5	23.2	6.3	88.0	8.8	64.0	12.1	3.1	1.8	0.0	0.0
			49.8	6.0	33.4	14.5	80.9	10.1	53.0	15.6	7.0	5.4	0.0	0.0
**DJ694 + DJ1077 + DJ1080 + DJ1083**														
DJ694;DJ1080	UAS-grim;DJ1077+DJ1083	04/25/2006	43.5	6.0	57.5	8.2	74.5	6.1	48.5	5.2	19.5	7.7	0.0	0.0
DJ694;DJ1077	UAS-grim;DJ1080+DJ1083	05/02/2006	89.0	2.6	86.1	4.9	59.8	7.6	78.0	4.3	80.4	6.6	0.0	0.0
DJ694;DJ1080	UAS-grim;DJ1077+DJ1083	05/30/2006	42.5	22.8	12.8	7.4	14.1	11.5	42.0	10.7	1.9	2.7	6.3	8.8
			58.3	26.6	52.1	36.9	49.5	31.5	56.2	19.2	33.9	41.2	2.1	3.6
**DJ694 + DJ1077 + 2 copies DJ1083**														
DJ694;DJ1083	UAS-grim;DJ1077+DJ1083	04/25/2006	26.5	11.1	25.5	16.5	41.7	20.9	21.0	5.3	4.3	4.0	0.0	0.0
			26.5	11.1	25.5	16.5	41.7	20.9	21.0	5.3	4.3	4.0	0.0	0.0
**DJ694 + DJ1079 + DJ1080 + DJ1083**														
DJ694;DJ1079	UAS-grim;DJ1080+DJ1083	05/02/2006	82.0	4.1	90.9	3.9	60.5	5.0	84.0	1.5	83.7	6.8	0.0	0.0
		06/06/2006	79.0	12.2	79.3	3.7	78.4	4.0	93.0	2.1	64.2	5.2	0.0	0.0
			80.5	2.1	85.1	8.2	69.4	12.6	88.5	6.4	73.9	13.8	0.0	0.0
**DJ694 + 2 copies DJ1080 + DJ1083**														
DJ694;DJ1080	UAS-grim;DJ1080+DJ1083	05/02/2006	80.0	4.7	78.4	4.2	43.2	6.0	80.0	3.7	71.6	3.6	0.0	0.0
		06/06/2006	85.5	2.6	75.7	5.1	47.4	8.5	95.0	1.8	33.8	4.1	0.0	0.0
			82.8	3.9	77.0	1.9	45.3	3.0	87.5	10.6	52.7	26.7	0.0	0.0
**DJ694 + 2 copies DJ1079 + DJ1080**														
DJ694;DJ1079	UAS-grim;DJ1079+DJ1080	05/02/2006	49.0	20.5	56.4	23.5	56.9	23.6	34.0	19.0	29.8	16.8	23.3	13.9
			49.0	20.5	56.4	23.5	56.9	23.6	34.0	19.0	29.8	16.8	23.3	13.9
**DJ694 + DJ1079 + 2 copies DJ1080**														
DJ694;DJ1080	UAS-grim;DJ1079+DJ1080	05/02/2006	87.0	4.2	80.9	3.9	78.8	4.3	90.5	3.7	78.8	4.5	42.9	6.1
			87.0	4.2	80.9	3.9	78.8	4.3	90.5	3.7	78.8	4.5	42.9	6.1
**DJ694 + DJ1077 + DJ1079 + DJ1083**														
DJ694;DJ1079	UAS-grim;DJ1077+DJ1083	05/30/2006	82.5	4.9	38.9	5.4	75.5	5.6	79.0	5.0	9.5	3.2	6.3	8.8
			82.5	4.9	38.9	5.4	75.5	5.6	79.0	5.0	9.5	3.2	6.3	8.8
**DJ694 + DJ1077 + DJ1079 + 2 copies DJ1080**														
DJ694;DJ1079+DJ1080	UAS-grim; DJ1077+DJ1080	05/09/2006	71.0	2.6	64.7	9.4	44.5	8.4	72.5	4.7	57.8	6.1	0.0	0.0
		06/13/2006	77.0	11.8	74.3	6.0	59.6	8.4	82.0	1.9	72.8	6.0	0.0	0.0
DJ694; DJ1077+DJ1080	UAS-grim;DJ1079+DJ1080	05/09/2006	88.5	3.6	80.7	2.5	72.6	3.6	89.0	2.8	83.9	5.6	34.9	5.3
			78.8	8.9	73.2	8.1	58.9	14.1	81.2	8.3	71.5	13.1	11.6	20.2
**DJ694 + 2 copies DJ1077 + 2 copies DJ1080**														
DJ694; DJ1077+DJ1080	UAS-grim; DJ1077+DJ1080	05/09/2006	89.5	1.8	84.7	4.9	53.9	7.6	91.0	3.7	63.9	4.0	0.0	0.0
		06/13/2006	85.5	3.5	78.1	5.8	57.4	6.9	94.0	2.4	63.5	6.5	0.0	0.0
			87.5	2.8	81.4	4.7	55.7	2.5	92.5	0.9	63.7	0.3	0.0	0.0

Parents: Male	Parents: Female	Date	%L1	SD	%P	SD	%A	SD	%L1	SD	%P	SD	%A	SD
			AMP						AMP/TET					
**DJ694 + 2 copies DJ1077 + DJ1080 + DJ1083**														
DJ694;DJ1077+DJ1083	UAS-grim; DJ1077+DJ1080	05/09/2006	91.0	1.4	78.6	3.5	55.7	6.8	87.5	3.3	55.4	6.2	36.6	8.5
		06/13/2006	93.5	2.4	82.9	4.7	80.4	4.6	94.0	2.6	68.2	6.4	78.9	5.3
DJ694; DJ1077+DJ1080	UAS-grim;DJ1077+DJ1083	05/16/2006	56.5	7.4	24.8	8.0	15.3	6.5	26.0	13.0	2.8	3.0	0.0	0.0
		06/06/2006	98.0	1.1	59.8	4.8	76.8	4.8	81.5	6.0	8.3	1.0	0.0	0.0
			77.3	29.3	42.3	24.7	46.0	43.5	53.8	39.2	5.6	3.9	0.0	0.0
**DJ694 + DJ1077 + 2 copies DJ1080 + DJ1083**														
DJ694;DJ1080+DJ1083	UAS-grim; DJ1077+DJ1080	05/09/2006	91.0	2.6	77.1	9.2	25.3	7.4	92.0	2.8	9.8	3.8	0.0	0.0
		06/13/2006	88.0	2.6	65.4	5.6	34.3	5.2	89.0	3.0	11.5	3.5	0.0	0.0
DJ694; DJ1077+DJ1080	UAS-grim;DJ1080+DJ1083	05/16/2006	94.5	1.8	86.8	3.1	73.7	4.7	95.0	2.1	57.1	6.0	0.0	0.0
			91.2	3.3	76.4	10.7	44.4	25.8	92.0	3.0	26.1	26.8	0.0	0.0
**DJ694 + 2 copies DJ1079 + 2 copies DJ1080**														
DJ694;DJ1079+DJ1080	UAS-grim;DJ1079+DJ1080	05/09/2006	83.5	3.6	83.1	5.3	84.7	5.3	78.0	2.1	92.5	1.4	38.2	10.3
			83.5	3.6	83.1	5.3	84.7	5.3	78.0	2.1	92.5	1.4	38.2	10.3
**DJ694 + DJ1077 + DJ1079 + DJ1080 + DJ1083**														
DJ694;DJ1077+DJ1083	UAS-grim;DJ1079+DJ1080	05/09/2006	89.5	3.4	88.9	3.4	81.7	4.0	92.0	2.6	77.8	3.9	81.0	6.7
DJ694;DJ1079+DJ1080	UAS-grim;DJ1077+DJ1083	05/16/2006	63.5	9.8	24.7	8.1	15.9	11.8	75.5	9.6	2.8	2.0	0.0	0.0
		06/06/2006	66.0	9.4	24.3	5.1	43.5	14.1	53.5	6.3	0.0	0.0	0.0	0.0
			64.8	1.8	24.5	0.2	29.7	19.5	64.5	15.6	1.4	2.0	0.0	0.0
**DJ694 + DJ1079 + 2 copies DJ1080 + DJ1083**														
DJ694;DJ1080+DJ1083	UAS-grim;DJ1079+DJ1080	05/09/2006	95.5	1.7	82.2	5.6	51.6	3.0	92.0	3.4	56.6	5.7	54.0	6.7
DJ694;DJ1079+DJ1080	UAS-grim;DJ1080+DJ1083	05/16/2006	92.0	2.4	79.3	7.4	67.7	7.3	86.5	3.8	66.6	7.7	0.0	0.0
		06/20/2006	75.0	11.6	59.3	10.4	20.1	5.5	92.5	1.3	53.7	9.2	0.0	0.0
			83.5	12.0	69.3	14.1	43.9	33.6	89.5	4.2	60.2	9.1	0.0	0.0
**DJ694 + DJ1077 + DJ1080 + 2 copies DJ1083**														
DJ694;DJ1077+DJ1083	UAS-grim;DJ1080+DJ1083	05/16/2006	93.0	1.8	80.5	4.6	75.1	4.6	88.5	3.6	58.5	7.6	45.5	13.0
		06/20/2006	93.5	2.4	76.0	4.4	59.9	5.9	96.0	2.1	48.9	4.3	65.6	8.2
DJ694;DJ1080+DJ1083	UAS-grim;DJ1077+DJ1083	05/16/2006	44.0	5.8	21.7	6.8	22.9	13.3	33.5	4.8	7.0	9.8	2.5	3.5
		06/06/2006	82.0	4.5	39.8	8.1	24.6	11.8	83.5	3.9	0.0	0.0	0.0	0.0
			78.1	23.4	54.5	28.4	45.7	26.0	75.4	28.4	28.6	29.4	28.4	32.4
**DJ694 + 2 copies DJ1080 + 2 copies DJ1083**														
DJ694;DJ1080+DJ1083	UAS-grim;DJ1080+DJ1083	05/16/2006	94.0	2.4	69.7	4.8	44.6	6.4	86.0	4.0	22.4	3.4	0.0	0.0
		06/20/2006	61.5	12.9	62.8	6.5	27.3	7.6	89.0	2.6	17.5	3.0	0.0	0.0
			77.8	23.0	66.3	4.9	35.9	12.2	87.5	2.1	19.9	3.5	0.0	0.0

Parents: Male	Parents: Female	Date	AMP %L1	SD	%P	SD	%A	SD	AMP/TET %L1	SD	%P	SD	%A	SD
			**0 GAL80**											
w	UAS-grim	04/13/2005	95.0	3.5	88.4	8.1	97.3	4.1						
w	UAS-grim	04/20/2005	95.5	3.3	81.2	4.4	96.1	3.6						
w	UAS-grim	04/27/2005	98.5	2.1	79.8	7.6	93.1	4.5	97.5	3.0	83.2	9.0	96.8	2.7
w	UAS-grim	05/04/2005	97.0	1.9	83.5	5.4	96.9	2.6	93.0	5.6	86.3	7.4	97.0	2.5
w	UAS-grim	05/11/2005	93.0	4.7	83.3	5.3	98.0	2.7	93.5	5.6	89.9	6.8	96.3	4.3
w	UAS-grim	05/11/2005	92.0	4.6	85.9	4.3	96.1	2.6	90.0	5.2	89.0	5.8	92.6	2.5
w	UAS-grim	05/18/2005	99.0	2.0	89.9	2.2	97.8	2.5	94.0	50.3	96.9	4.0	99.0	2.1
w	UAS-grim	04/11/2006	88.0	10.0	85.0	8.9	97.2	3.0	89.0	6.0	85.5	5.8	99.4	1.8
UAS-grim	w	01/26/2006	74.5	4.9	88.0	6.5	99.4	0.9	72.0	3.0	70.3	4.3	97.5	2.4
w	UAS-grim	04/19/2006	90.5	5.6	87.8	5.3	98.7	2.4	93.5	8.3	82.8	10.8	98.3	3.4
w	UAS-grim	04/25/2006	96.0	4.3	86.5	6.9	98.9	2.0	94.5	3.0	91.5	5.1	98.3	2.3
w	UAS-grim	05/02/2006	99.0	1.9	90.4	5.2	99.5	1.5	97.0	4.1	90.8	5.1	98.3	2.4
w	UAS-grim	05/09/2006	95.0	5.1	89.1	6.9	98.1	2.6	96.5	4.5	91.0	7.0	100.0	0.0
w	UAS-grim	05/16/2006	92.5	10.4	84.5	11.3	99.3	2.1	86.0	12.1	70.8	12.0	95.8	4.9
w	UAS-grim	05/24/2006	83.5	9.9	76.0	5.8	92.7	9.7	80.5	10.4	78.7	7.2	97.8	3.1
w	UAS-grim	05/30/2006	98.5	3.0	78.7	6.2	98.7	2.5	100.0	0.0	83.0	6.0	100.0	0.0
w	UAS-grim	06/06/2006	99.0	1.9	76.2	12.1	98.8	2.2	99.0	1.9	88.9	6.7	99.4	1.7
w	UAS-grim	09/26/2006	98.5	2.1	92.4	2.7	98.9	2.0	98.5	2.1	91.9	3.0	98.4	2.2
w	UAS-grim	10/05/2006	91.0	3.8	100.0	0.0	99.0	2.1	95.0	5.0	99.0	2.0	100.0	0.0
w	UAS-grim	10/12/2006	97.0	6.0	93.0	8.2	100.0	0.0	98.0	2.3	88.8	6.1	100.0	0.0
w	UAS-grim	10/19/2006	99.0	2.0	88.8	7.2	95.2	4.3	98.0	2.3	90.0	7.6	98.9	2.3
w	UAS-grim	10/23/2006	94.0	7.7	89.4	7.8	98.8	2.5	98.0	2.3	87.8	6.8	100.0	0.0
w	UAS-grim	11/02/2006	89.0	3.8	81.2	11.7	98.5	3.1	95.0	3.8	84.3	8.8	100.0	0.0
w	UAS-grim	11/16/2006	94.0	3.5	90.6	1.8	97.6	1.4	96.0	2.3	89.5	4.2	97.5	1.5
w	UAS-grim	12/07/2006	98.0	1.2	90.9	3.5	99.0	1.0	99.0	1.0	86.8	5.7	98.8	1.3
w	UAS-grim	01/24/2007	99.0	1.0	91.9	2.8	100.0	0.0	96.0	2.3	89.7	2.6	97.7	1.3
w	UAS-grim	05/18/2005	97.0	4.1	86.9	5.7	94.9	6.0	96.0	3.0	90.7	9.3	97.8	2.4
w	UAS-grim	11/27/2007	96.0	3.3	87.6	9.0	100.0	0.0	100.0	0.0	81.0	6.0	97.4	5.3
w	UAS-grim	12/18/2007	93.0	3.8	87.1	10.4	97.8	4.5	91.0	5.0	92.5	6.5	98.9	2.2
w	UAS-grim	01/18/2007	88.0	1.6	92.1	3.4	98.9	1.1	87.0	2.5	95.4	0.1	97.5	1.4
			94.0	5.4	86.9	5.3	97.8	1.9	93.7	6.3	87.4	6.6	98.2	1.6
			**DJ694 + 2.1**											
UAS-grim;2.1	DJ694	09/26/2006	77.0	4.8	51.1	7.9	0.0	0.0	72.0	7.6	13.2	3.2	0.0	0.0
		09/26/2006	88.5	2.3	19.0	7.7	0.0	0.0	85.0	2.1	25.3	4.7	0.0	0.0
DJ694;2.1	UAS-grim	10/12/2006	21.0	3.0	0.0	0.0	0.0	0.0	40.0	5.2	0.0	0.0	0.0	0.0
		10/19/2006	22.0	5.3	0.0	0.0	0.0	0.0	39.0	2.5	0.0	0.0	0.0	0.0
			52.1	35.7	17.5	24.1	0.0	0.0	59.0	23.1	9.6	12.1	0.0	0.0
			**DJ694 + 3.2**											
3.2;DJ694	UAS-grim	09/26/2006	37.5	6.3	14.9	7.6	0.0	0.0	47.0	4.8	3.6	2.5	0.0	0.0
UAS-grim	3.2;DJ694	11/16/2006	85.0	3.8	17.3	3.2	0.0	0.0	81.0	3.4	0.0	0.0	0.0	0.0
		11/23/2006	85.0	4.1	7.4	3.3	0.0	0.0	72.0	2.8	0.0	0.0	0.0	0.0
			69.2	27.4	13.2	5.2	0.0	0.0	66.7	17.6	1.2	2.1	0.0	0.0
			**DJ694 + 3.3**											
UAS-grim;3.3	DJ694	09/26/2006	33.0	3.8	8.0	4.5	0.0	0.0	15.5	3.1	0.0	0.0	0.0	0.0
		09/26/2006	66.0	5.0	3.1	1.7	0.0	0.0	50.5	3.2	0.0	0.0	0.0	0.0
DJ694;3.3	UAS-grim	10/12/2006	32.0	2.3	0.0	0.0	0.0	0.0	43.0	7.5	0.0	0.0	0.0	0.0
		10/19/2006	33.0	3.4	0.0	0.0	0.0	0.0	51.0	3.4	0.0	0.0	0.0	0.0
DJ694	UAS-grim;3.3	11/16/2006	60.0	4.9	23.7	8.4	0.0	0.0	53.0	4.4	31.4	15.9	0.0	0.0
		12/07/2006	85.0	3.4	45.1	6.7	10.8	4.2	78.0	4.8	10.1	1.8	0.0	0.0
			51.5	22.2	13.3	17.9	1.8	4.4	48.5	20.1	6.9	12.6	0.0	0.0
			**DJ694 + 4**											
UAS-grim	4;DJ694	11/16/2006	91.0	3.0	10.3	5.1	0.0	0.0	87.0	4.1	6.9	2.9	0.0	0.0
		11/23/2006	77.0	4.1	3.8	2.3	0.0	0.0	70.0	4.8	0.0	0.0	0.0	0.0
			84.0	9.9	7.0	4.6	0.0	0.0	78.5	12.0	3.5	4.9	0.0	0.0
			**3.3**											
		06/27/2006	76.5	6.3	83.5	4.5	88.7	4.4	82.0	4.0	100.0	0.0	90.2	3.6
UAS-grim;3.3	w	10/23/2006	93.0	3.0	78.5	1.7	91.2	7.0	95.0	1.0	90.6	4.3	100.0	0.0
		38986.00	76.5	6.6	83.5	5.6	88.7	2.1	82.0	6.3	89.6	6.2	91.0	2.1
DJ694;3.3	w	06/27/2006	79.0	12.1	88.2	4.8	100.0	0.0	82.0	3.4	87.4	3.9	99.4	0.9
		10/23/2006	82.0	4.8	63.2	5.7	100.0	0.0	92.0	0.0	56.6	1.8	100.0	0.0
			81.4	6.9	79.4	9.7	93.7	5.8	86.6	6.4	84.8	16.5	96.1	5.1

Parents		Date	%L1	SD	%P	SD	%A	SD	%L1	SD	%P	SD	%A	SD
Male	Female		AMP						AMP/TET					
					**2.1**									
UAS-grim;2.1	w	10/23/2006	99.0	1.0	88.9	4.5	100.0	0.0	100.0	0.0	81.0	6.0	100.0	0.0
		38986.00	73.0	6.3	81.3	12.8	100.0	0.0	79.5	7.4	83.2	11.4	99.3	0.0
DJ694;2.1	w	06/27/2006	73.0	3.2	81.3	6.4	100.0	0.0	79.5	3.3	83.2	2.8	99.3	1.0
		10/23/2006	79.0	1.0	88.7	2.4	95.7	2.8	73.0	1.9	86.2	1.9	100.0	0.0
			81.0	2.5	85.0	4.5	98.9	1.4	83.0	3.1	83.4	4.3	99.6	0.5
					**3.2**									
3.2;DJ694	w	38986.00	78.5	6.2	72.6	11.0	100.0	0.0	72.0	4.2	76.6	10.3	97.2	0.0
		06/27/2006	78.5	1.5	72.6	3.3	100.0	0.0	72.0	3.9	76.6	4.9	97.2	2.0
			78.5	0.0	72.6	0.0	100.0	0.0	72.0	0.0	76.6	0.0	97.2	0.0
					**DJ694 + 2 copies 2.1**									
UAS-grim;2.1	DJ694;2.1	09/26/2006	69.0	5.8	81.6	4.6	19.1	2.6	76.0	3.9	67.3	8.6	0.0	0.0
		10/23/2006	93.0	1.9	85.1	2.6	51.4	12.7	94.0	3.5	69.4	3.0	0.0	0.0
DJ694;2.1	UAS-grim;2.1	11/02/2006	89.0	1.0	85.4	2.3	67.6	9.6	88.0	2.8	81.9	2.4	4.1	1.4
		11/16/2006	94.0	1.2	88.5	3.5	27.8	2.2	94.0	3.5	81.9	2.0	0.0	0.0
			86.3	11.7	85.1	2.8	41.5	22.1	88.0	8.5	75.1	7.9	1.0	2.1
					**DJ694 + 2.1 + 3.3**									
UAS-grim;2.1	DJ694;3.3	09/26/2006	88.5	2.3	65.6	3.9	36.2	5.1	75.5	6.0	40.8	8.1	0.0	0.0
UAS-grim;3.3	DJ694;2.1	09/26/2006	81.5	4.4	46.8	6.4	16.7	7.4	74.0	2.8	26.9	3.6	0.0	0.0
		10/12/2006	47.0	3.0	78.5	9.7	42.1	7.8	52.0	3.7	76.9	2.9	0.0	0.0
DJ694;3.3	UAS-grim;2.1	10/23/2006	90.0	3.5	70.6	5.3	22.4	12.0	91.0	2.5	65.9	2.6	0.0	0.0
		11/02/2006	79.0	2.5	73.4	4.3	77.7	9.2	74.0	2.6	52.5	2.4	0.0	0.0
		11/16/2006	84.0	4.3	61.4	8.0	38.4	6.2	76.0	1.6	58.0	4.0	0.0	0.0
DJ694;2.1	UAS-grim;3.3	11/16/2006	81.0	6.2	65.2	3.2	3.4	2.0	73.0	4.4	61.9	2.3	0.0	0.0
		12/07/2006	90.0	1.2	71.2	2.1	53.0	2.8	86.0	1.2	28.0	4.4	0.0	0.0
			80.1	14.0	66.6	9.6	36.2	23.0	75.2	11.4	51.3	18.0	0.0	0.0
					**DJ694 + 2.1 + 3.2**									
UAS-grim;2.1	3.2;DJ694	09/26/2006	64.5	6.1	45.9	14.2	6.8	5.0	54.5	7.0	36.4	9.4	0.0	0.0
		11/16/2006	89.0	4.4	54.0	8.3	0.0	0.0	86.0	3.5	51.1	2.7	0.0	0.0
		11/23/2006	87.0	5.0	69.6	4.8	0.0	0.0	84.0	2.3	48.7	3.1	0.0	0.0
			80.2	13.6	56.5	12.1	2.3	3.9	74.8	17.6	45.4	7.9	0.0	0.0
					**DJ694 + 2 copies 3.3**									
UAS-grim;3.3	DJ694;3.3	09/26/2006	42.0	3.4	42.3	6.2	40.1	5.7	61.0	5.7	20.9	6.8	0.0	0.0
		10/12/2006	44.0	9.4	60.1	7.0	9.8	6.1	66.0	5.0	8.3	3.2	0.0	0.0
DJ694;3.3	UAS-grim;3.3	11/16/2006	67.0	3.0	52.5	6.1	0.0	0.0	68.0	7.8	64.2	7.3	0.0	0.0
		12/07/2006	67.0	5.3	16.3	1.9	52.1	16.8	71.0	1.0	24.0	3.5	0.0	0.0
			55.0	13.9	42.8	19.1	25.5	24.6	66.5	4.2	29.4	24.2	0.0	0.0
					**DJ694 + 3.2 + 3.3**									
		09/26/2006	58.0	7.0	51.9	6.6	6.7	3.9	52.0	4.8	9.9	3.7	0.0	0.0
UAS-grim;3.3	3.2;DJ694	11/16/2006	78.0	1.2	47.3	6.3	0.0	0.0	90.0	2.6	12.6	4.7	0.0	0.0
		11/23/2006	82.0	2.6	39.5	5.4	0.0	0.0	76.0	3.7	11.1	3.6	0.0	0.0
			72.7	12.9	46.2	6.3	2.2	3.9	72.7	19.2	11.2	1.3	0.0	0.0
					**DJ694 + 2.1 + 4**									
UAS-grim;2.1	4;DJ694	11/16/2006	86.0	4.2	29.0	8.0	0.0	0.0	77.0	3.0	25.7	3.6	0.0	0.0
		11/23/2006	77.0	4.1	44.2	11.0	0.0	0.0	87.0	3.4	25.2	2.2	0.0	0.0
			81.5	6.4	36.6	10.7	0.0	0.0	82.0	7.1	25.4	0.4	0.0	0.0
					**DJ694 + 3.3 + 4**									
UAS-grim;3.3	4;DJ694	11/16/2006	95.0	3.0	15.9	1.4	0.0	0.0	94.0	2.6	9.8	3.3	0.0	0.0
		11/23/2006	90.0	2.0	23.1	5.8	0.0	0.0	88.0	3.7	4.8	2.1	0.0	0.0
			92.5	3.5	19.5	5.1	0.0	0.0	91.0	4.2	7.3	3.6	0.0	0.0
					**DJ694 + DJ1077 + 3.3**									
DJ694;DJ1077	UAS-grim;3.3	12/07/2006	92.0	3.7	63.0	7.6	78.3	2.9	77.0	4.4	19.7	4.3	0.0	0.0
		11/30/2006	91.0	4.1	64.1	6.4	86.5	3.2	96.0	1.6	22.9	2.4	0.0	0.0
DJ694;3.3	UAS-grim;DJ1077	12/14/2006	84.0	4.9	64.3	4.7	95.7	2.5	77.0	2.5	18.2	6.6	0.0	0.0
		12/14/2006	50.0	5.8	38.5	6.0	100.0	0.0	42.0	2.0	14.6	3.1	0.0	0.0
			79.3	19.8	57.4	12.7	90.1	9.7	73.0	22.5	18.8	3.4	0.0	0.0
					**DJ694 + DJ1080 + 3.3**									
DJ694;DJ1080	UAS-grim;3.3	12/07/2006	97.0	1.9	49.6	1.0	77.1	8.6	95.0	1.9	11.6	3.6	0.0	0.0
		11/30/2006	92.0	2.8	53.7	4.5	38.6	2.2	99.0	1.0	22.3	4.0	0.0	0.0
DJ694;3.3	UAS-grim;DJ1080	12/14/2006	72.0	1.6	54.2	6.9	23.9	7.7	80.0	2.8	14.7	6.1	0.0	0.0
		12/14/2006	41.0	5.7	32.7	10.6	57.9	16.5	38.0	6.2	0.0	0.0	0.0	0.0
			75.5	25.4	47.6	10.1	49.4	23.2	78.0	27.9	12.1	9.3	0.0	0.0

Parents		Date	%L1	SD	%P	SD	%A	SD	%L1	SD	%P	SD	%A	SD
Male	Female		AMP						AMP/TET					
			**DJ694 + DJ1083 + 3.3**											
DJ694;DJ1083	UAS-grim;3.3	12/07/2006	97.0	1.9	64.9	2.8	53.9	2.4	97.0	4.4	30.2	2.3	92.9	4.1
		11/30/2006	93.0	3.0	50.4	3.6	82.6	7.7	82.0	6.0	45.9	4.3	94.4	3.3
DJ694;3.3	UAS-grim;DJ1083	12/07/2006	84.0	2.8	69.1	2.0	34.2	6.2	60.0	2.8	28.0	4.0	0.0	0.0
		12/14/2006	84.0	5.4	55.8	4.1	27.0	5.8	83.0	4.4	28.2	3.7	0.0	0.0
			84.0	0.0	62.5	9.4	30.6	5.1	71.5	16.3	28.1	0.1	0.0	0.0
			**DJ694 + DJ1079 + 3.3**											
DJ694;3.3	UAS-grim;DJ1079	12/07/2006	86.0	3.8	44.5	5.9	16.5	9.7	78.0	2.6	11.7	1.4	0.0	0.0
		12/14/2006	72.0	4.9	64.7	8.2	66.0	15.3	82.0	4.8	34.4	4.9	0.0	0.0
		12/14/2006	99.0	1.0	71.9	8.7	88.9	4.8	92.0	1.6	44.4	4.7	0.0	0.0
			85.7	13.5	60.4	14.2	57.1	37.0	84.0	7.2	30.2	16.8	0.0	0.0
			**DJ694 + DJ1079 + 4**											
4;DJ694	UAS-grim;DJ1079	12/07/2006	84.0	2.8	28.0	7.7	0.0	0.0	84.0	2.3	8.3	2.2	0.0	0.0
		12/14/2006	80.0	2.8	11.6	6.7	0.0	0.0	75.0	3.4	0.0	0.0	0.0	0.0
			82.0	2.8	19.8	11.6	0.0	0.0	79.5	6.4	4.2	5.9	0.0	0.0
			**DJ694 + DJ1083 + 4**											
4;DJ694	UAS-grim;DJ1083	12/07/2006	85.0	4.1	32.8	11.2	0.0	0.0	90.0	2.0	2.2	1.3	0.0	0.0
		12/14/2006	86.0	2.0	62.0	8.2	0.0	0.0	74.0	2.6	9.1	6.2	0.0	0.0
			85.5	0.7	47.4	20.7	0.0	0.0	82.0	11.3	5.7	4.9	0.0	0.0
			**DJ694 + DJ1077 + 2.1**											
DJ694;DJ1077	UAS-grim;2.1	12/07/2006	94.0	1.2	71.2	3.9	82.5	2.4	89.0	1.9	57.4	4.3	0.0	0.0
		02/01/2007	97.0	1.0	66.1	4.3	91.2	3.5	90.0	4.2	46.8	1.4	0.0	0.0
		02/08/2007	97.0	1.0	72.1	4.1	88.8	1.9	92.0	1.6	44.8	5.9	0.0	0.0
DJ694;2.1	UAS-grim;DJ1077	01/18/2007	90.0	3.5	62.6	5.7	89.9	5.3	76.0	4.9	48.3	3.1	0.0	0.0
		01/24/2007	94.0	2.6	54.5	2.4	84.5	4.3	87.0	1.9	34.5	3.8	0.0	0.0
			94.4	2.9	69.8	3.2	87.5	4.5	90.3	1.5	49.6	6.8	0.0	0.0
			**DJ694 + DJ1080 + 2.1**											
DJ694;DJ1080	UAS-grim;2.1	12/07/2006	88.0	2.8	57.7	3.1	20.0	7.4	94.0	1.2	37.3	2.2	0.0	0.0
		02/01/2007	97.0	1.0	58.8	5.5	37.5	12.9	95.0	1.9	41.0	3.7	0.0	0.0
		02/08/2007	97.0	1.0	61.9	2.7	31.7	0.6	92.0	2.8	48.9	4.6	0.0	0.0
DJ694;2.1	UAS-grim;DJ1080	01/18/2007	93.0	3.4	67.0	5.0	39.5	5.5	84.0	4.3	38.1	7.7	0.0	0.0
		01/24/2007	82.0	4.8	63.6	2.9	57.6	4.7	85.0	1.9	37.2	13.5	1.8	1.8
			91.4	6.4	61.8	3.7	37.3	13.7	90.0	5.1	40.5	5.0	0.4	0.8
			**DJ694 + DJ1083 + 2.1**											
DJ694;DJ1083	UAS-grim;2.1	12/07/2006	89.0	1.9	59.4	4.0	26.5	6.9	85.0	3.4	41.9	5.8	40.8	6.8
		02/01/2007	97.0	1.9	63.1	7.1	42.8	6.4	92.0	3.3	45.5	2.2	0.0	0.0
		02/08/2007	98.0	1.2	67.6	6.1	49.0	11.3	97.0	1.0	59.8	4.3	0.0	0.0
			97.5	0.7	65.3	3.2	45.9	4.3	94.5	3.5	52.7	10.1	0.0	0.0
			**DJ694 + DJ1077 + 4**											
4;DJ694	UAS-grim;DJ1077	12/14/2006	84.0	5.2	67.3	4.8	6.0	4.0	85.0	4.1	7.3	2.6	0.0	0.0
		12/14/2006	84.0	2.3	59.0	8.8	0.0	0.0	82.0	6.2	0.0	0.0	0.0	0.0
			84.0	0.0	63.1	5.9	3.0	4.2	83.5	2.1	3.6	5.1	0.0	0.0
			**DJ694 + DJ1080 + 4**											
4;DJ694	UAS-grim;DJ1080	12/14/2006	69.0	10.0	11.4	6.9	0.0	0.0	56.0	5.2	9.6	2.9	0.0	0.0
		12/14/2006	63.0	5.7	17.4	6.6	0.0	0.0	56.0	6.3	8.1	3.9	0.0	0.0
			66.0	4.2	14.4	4.2	0.0	0.0	56.0	0.0	8.9	1.1	0.0	0.0
			**DJ694 + DJ1079 + 2.1**											
DJ694;2.1	UAS-grim;DJ1079	01/18/2007	94.0	2.0	88.2	4.1	89.8	5.4	88.0	2.8	44.4	1.9	0.0	0.0
		01/24/2007	94.0	2.6	73.8	4.0	91.3	3.7	91.0	2.5	54.9	6.8	0.0	0.0
DJ694;DJ1079	UAS-grim;2.1	02/01/2007	97.0	1.0	57.8	5.1	31.8	4.7	93.0	1.9	29.2	3.8	3.6	3.6
		02/08/2007	94.0	3.5	57.7	8.1	3.8	2.4	93.0	3.0	47.5	4.0	0.0	0.0
			94.8	1.5	69.4	14.7	54.2	43.5	91.3	2.4	44.0	10.8	0.9	1.8
			**DJ1077 + 2.1**											
w	UAS-grim;2.1+DJ1077	11/20/2007	100.0	0.0	74.0	7.7	100.0	0.0	97.0	3.8	70.1	6.3	100.0	0.0
		12/11/2007	86.0	8.3	78.6	10.9	97.4	5.3	91.0	8.9	75.5	8.8	95.0	6.4
			93.0	9.9	76.3	3.2	98.7	1.9	94.0	4.2	72.8	3.9	97.5	3.5
			**DJ1079 + 2.1**											
w	UAS-grim;2.1+DJ1079(13)	11/27/2007	75.0	13.2	52.9	11.8	100.0	0.0	93.0	5.0	57.1	3.8	100.0	0.0
		12/18/2007	85.0	6.0	60.0	15.1	100.0	0.0	80.3	4.9	69.2	13.8	100.0	0.0
w	UAS-grim;2.1+DJ1079(14)	11/13/2007	94.0	5.2	85.3	4.9	98.7	2.6	94.0	2.3	84.3	11.3	98.8	2.4
		12/04/2007	87.0	3.8	80.7	11.7	100.0	0.0	79.0	5.0	74.6	4.4	98.5	3.1
			85.3	7.8	69.7	15.7	99.7	0.6	86.6	8.0	71.3	11.4	99.3	0.8

Parents		Date	%L1	SD	%P	SD	%A	SD	%L1	SD	%P	SD	%A	SD
Male	Female		AMP						AMP/TET					
			**DJ1077 + 3.3**											
w	UAS-grim;3.3+DJ1077	11/20/2007	46.0	12.4	88.5	13.3	100.0	0.0	72.0	3.3	90.3	12.3	100.0	0.0
		12/11/2007	90.0	9.5	54.4	14.9	100.0	0.0	86.0	8.3	60.1	14.7	100.0	0.0
			68.0	31.1	71.4	24.1	100.0	0.0	79.0	9.9	75.2	21.3	100.0	0.0
			**DJ1079 + 3.3**											
w	UAS-grim;3.3+DJ1079	11/13/2007	89.0	3.8	54.1	9.1	96.2	4.4	84.0	5.7	47.9	9.9	100.0	0.0
		12/04/2007	74.0	16.5	92.2	4.0	98.1	3.8	72.0	11.8	96.1	4.6	98.6	2.9
			81.5	10.6	73.1	26.9	97.2	1.3	78.0	8.5	72.0	34.1	99.3	1.0
			**DJ694 + DJ1077 + DJ1080 + 2.1**											
DJ694;2.1	UAS-grim; DJ1077+DJ1080	10/05/2006	90.0	1.2	66.6	4.7	15.2	5.2	96.0	1.6	70.7	8.4	0.0	0.0
		10/12/2006	69.0	6.6	80.5	5.2	69.6	7.1	69.0	1.9	59.6	4.5	0.0	0.0
		11/16/2006	99.0	1.0	84.9	0.9	83.4	5.7	96.0	1.6	77.0	3.0	0.0	0.0
DJ694; DJ1077+DJ1080	UAS-grim;2.1	10/19/2006	95.0	1.0	87.5	1.6	85.4	5.5	94.0	4.8	53.4	12.5	0.0	0.0
		11/02/2006	95.0	3.0	91.9	4.3	81.8	4.8	91.0	2.5	74.7	2.4	0.0	0.0
		12/07/2006	83.0	3.8	87.5	3.8	67.9	5.1	88.0	2.3	56.1	6.8	0.0	0.0
			88.5	11.0	83.1	8.9	67.2	26.5	89.0	10.3	65.2	10.1	0.0	0.0
			**DJ694 + DJ1077 + DJ1083 + 2.1**											
DJ694;2.1	UAS-grim;DJ1077+DJ1083	10/05/2006	57.0	6.6	53.2	13.1	11.8	6.8	69.0	8.4	37.9	7.4	0.0	0.0
		10/12/2006	28.0	3.3	58.9	7.0	80.4	7.1	24.0	4.9	53.3	10.5	0.0	0.0
		11/16/2006	69.0	5.3	67.4	5.6	88.4	4.4						
DJ694;DJ1077+DJ1083	UAS-grim;2.1	10/19/2006	95.0	3.0	86.3	4.5	75.2	3.0	99.0	1.0	69.9	6.9	83.8	1.8
			51.3	21.1	59.8	7.1	60.2	42.1	46.5	31.8	45.6	10.9	0.0	0.0
			**DJ694 + DJ1080 + DJ1083 + 2.1**											
DJ694;2.1	UAS-grim;DJ1080+DJ1083	10/05/2006	85.0	3.4	61.4	2.5	19.3	7.4	76.0	3.7	42.4	5.2	0.0	0.0
		10/12/2006	67.0	6.2	86.3	7.8	54.7	10.9	75.0	6.6	59.5	7.6	0.0	0.0
		11/16/2006	96.0	0.0	87.5	0.0	91.7	2.3	95.0	1.0	68.5	7.1	0.0	0.0
DJ694;DJ1080+DJ1083	UAS-grim;2.1	10/19/2006	99.0	1.0	74.8	4.1	11.6	4.7	96.0	1.6	27.2	2.1	0.0	0.0
		11/02/2006	97.0	1.0	85.7	3.5	23.4	7.7	98.0	2.0	53.3	4.7	0.0	0.0
		12/07/2006	84.0	4.3	58.0	7.4	10.8	4.6	88.0	3.7	25.4	3.3	0.0	0.0
			88.0	12.1	75.6	13.2	35.2	32.0	88.0	10.3	46.1	17.5	0.0	0.0
			**DJ694 + DJ1077 + DJ1080 + 3.3**											
DJ694;3.3	UAS-grim; DJ1077+DJ1080	10/05/2006	44.0	5.7	60.7	8.3	41.9	16.9	82.0	4.8	41.6	3.5	0.0	0.0
		10/12/2006	66.0	4.8	83.3	2.7	90.9	1.8	71.0	4.1	69.4	14.2	0.0	0.0
		11/16/2006	97.0	1.0	75.3	3.0	95.8	2.6	95.0	1.9	60.9	9.6	0.0	0.0
DJ694; DJ1077+DJ1080	UAS-grim;3.3	10/19/2006	94.0	2.6	69.6	5.1	92.5	3.0	87.0	1.0	40.4	5.1	0.0	0.0
		11/02/2006	87.0	1.9	94.3	1.1	92.5	3.2	93.0	1.9	44.4	7.9	0.0	0.0
			77.6	22.3	76.6	12.9	82.7	22.9	85.6	9.6	51.3	13.0	0.0	0.0
			**DJ694 + DJ1077 + DJ1083 + 3.3**											
DJ694;3.3	UAS-grim;DJ1077+DJ1083	10/05/2006	56.0	2.3	53.4	2.8	59.8	8.5	41.0	4.4	46.9	3.5	0.0	0.0
		10/12/2006	34.0	2.6	49.4	3.9	77.1	10.4	32.0	7.5	46.8	7.0	0.0	0.0
		11/16/2006	86.0	4.8	30.3	2.7	91.9	4.9	29.0	2.5	67.7	10.1	0.0	0.0
DJ694;DJ1077+DJ1083	UAS-grim;3.3	10/19/2006	93.0	1.0	76.6	4.8	88.1	7.0	86.0	4.8	66.3	5.9	79.4	8.2
			58.7	26.1	44.3	12.3	76.3	16.1	34.0	6.2	53.8	12.0	0.0	0.0
			**DJ694 + DJ1080 + DJ1083 + 3.3**											
DJ694;3.3	UAS-grim;DJ1080+DJ1083	10/05/2006	67.0	1.9	67.6	5.0	48.3	9.8	67.0	5.0	30.0	3.8	0.0	0.0
		10/12/2006	57.0	3.4	96.4	2.1	100.0	0.0	70.0	3.8	56.5	5.9	0.0	0.0
		11/16/2006	98.0	1.2	63.1	4.8	100.0	0.0	85.0	3.4	66.3	3.7	0.0	0.0
DJ694;DJ1080+DJ1083	UAS-grim;3.3	10/19/2006	88.0	3.3	38.4	6.1	54.5	11.1	89.0	3.0	12.3	1.9	0.0	0.0
		11/02/2006	91.0	1.9	66.1	7.6	30.5	6.9	88.0	3.7	18.1	2.8	0.0	0.0
			80.2	17.4	66.3	20.6	66.6	31.7	79.8	10.5	36.6	23.7	0.0	0.0
			**DJ694 + DJ1079 + DJ1080 + 2.1**											
DJ694;DJ1079+DJ1080	UAS-grim;2.1	10/19/2006	93.0	1.9	94.7	3.2	80.7	7.3	89.0	4.4	58.3	3.5	0.0	0.0
		11/02/2006	65.0	6.6	86.8	4.5	59.7	6.1	57.0	8.2	53.7	3.5	0.0	0.0
		12/07/2006	79.0	6.8	67.4	4.0	62.1	1.4	79.0	3.8	51.6	8.0	0.0	0.0
			79.0	14.0	83.0	14.0	67.5	11.5	75.0	16.4	54.5	3.5	0.0	0.0
			**DJ694 + DJ1079 + DJ1080 + 3.3**											
DJ694;DJ1079+DJ1080	UAS-grim;3.3	10/19/2006	92.0	5.4	70.7	6.1	94.8	3.7	85.0	5.0	65.6	13.1	0.0	0.0
		11/02/2006	77.0	3.4	92.6	3.1	91.6	1.7	82.0	2.0	54.1	4.9	0.0	0.0
			84.5	10.6	81.7	15.5	93.2	2.3	83.5	2.1	59.9	8.2	0.0	0.0
			**DJ694 + 2 copies DJ1077 + 2 copies 2.1**											
DJ694;2.1+DJ1077	UAS-grim;2.1+DJ1077	11/20/2007	82.0	12.0	69.3	12.0	93.5	4.4	81.0	2.0	75.4	10.6	0.0	0.0
		12/11/2007	84.0	5.7	88.8	11.7	93.5	10.2	78.0	4.0	88.6	7.4	0.0	0.0
			83.0	1.4	79.0	13.8	93.5	0.0	79.5	2.1	82.0	9.4	0.0	0.0

Parents: Male	Parents: Female	Date	AMP %L1	AMP SD	AMP %P	AMP SD	AMP %A	AMP SD	AMP/TET %L1	AMP/TET SD	AMP/TET %P	AMP/TET SD	AMP/TET %A	AMP/TET SD
**DJ694 + DJ1077 + DJ1079 + 2 copies 2.1**														
DJ694;2.1+DJ1079(13)	UAS-grim;2.1+DJ1077	11/20/2007	67.0	13.2	50.4	10.9	86.7	10.8	56.0	3.3	50.4	10.1	0.0	0.0
		12/11/2007	80.0	3.3	50.0	6.8	88.5	8.6	62.0	23.2	81.0	14.1	0.0	0.0
DJ694;2.1+DJ1077	UAS-grim;2.1+DJ1079(13)	11/27/2007	72.0	8.6	49.1	9.8	72.5	22.2	69.0	7.6	55.6	9.6	0.0	0.0
		12/18/2007	70.0	6.9	46.6	9.1	90.8	6.7	69.0	8.2	53.4	7.9	0.0	0.0
DJ694;2.1+DJ1079(14)	UAS-grim;2.1+DJ1077	11/20/2007	67.0	3.8	55.6	14.3	70.8	12.8	61.0	15.1	87.4	16.5	0.0	0.0
		12/11/2007	63.0	10.0	76.2	12.7	97.2	5.6	57.0	13.6	90.2	13.9	0.0	0.0
DJ694;2.1+DJ1077	UAS-grim;2.1+DJ1079(14)	11/13/2007	62.0	5.2	93.1	10.1	97.1	5.9	64.0	4.6	98.4	3.3	15.5	6.9
		12/04/2007	85.0	8.9	84.7	8.7	97.6	4.8	70.0	10.6	96.7	8.5	8.7	7.5
			70.8	8.1	63.2	18.5	87.7	10.7	63.5	5.5	76.6	20.2	3.0	5.9
**DJ694 + 2 copies DJ1077 + 2.1 + 3.3**														
DJ694;3.3+DJ1077	UAS-grim;2.1+DJ1077	11/20/2007	63.0	14.7	96.3	7.5	96.2	4.5	64.0	7.3	85.7	17.4	0.0	0.0
		12/11/2007	82.0	6.9	74.5	10.7	100.0	0.0	71.0	13.2	78.7	11.0	0.0	0.0
DJ694;2.1+DJ1077	UAS-grim;3.3+DJ1077	11/20/2007	51.0	7.6	91.2	10.4	100.0	0.0	34.0	6.9	95.0	10.0	12.9	2.6
		12/11/2007	87.0	3.8	72.3	16.3	100.0	0.0	55.0	15.1	85.4	10.7	33.2	4.9
			70.8	16.7	83.6	12.0	99.1	1.9	56.0	16.1	86.2	6.7	11.5	15.7
**DJ694 + 2 copies DJ1079 + 2 copies 2.1**														
DJ694;2.1+DJ1079(13)	UAS-grim;2.1+DJ1079(13)	11/27/2007	59.0	8.9	55.2	15.2	94.7	6.1	66.0	10.1	76.9	25.2	0.0	0.0
		12/18/2007	78.0	6.9	32.9	14.2	91.7	10.6	58.0	4.0	61.6	27.0	0.0	0.0
DJ694;2.1+DJ1079(14)	UAS-grim;2.1+DJ1079(14)	11/13/2007	76.0	11.3	78.6	15.9	100.0	0.0	80.0	8.6	80.9	7.2	11.2	9.5
		12/04/2007	81.0	13.2	61.1	14.8	95.9	8.3	71.0	8.9	83.5	7.5	15.8	9.4
DJ694;2.1+DJ1079(14)	UAS-grim;2.1+DJ1079(13)	11/27/2007	64.0	16.3	63.9	12.1	69.0	22.2	65.0	11.0	68.1	15.8	0.0	0.0
		12/18/2007	77.0	3.8	35.0	10.1	75.8	21.4	76.0	5.7	59.4	9.3	0.0	0.0
DJ694;2.1+DJ1079(13)	UAS-grim;2.1+DJ1079(14)	11/13/2007	91.0	3.8	76.1	6.7	95.4	5.9	80.0	8.0	89.1	6.9	23.2	10.4
		12/04/2007	80.0	8.6	73.6	6.5	100.0	0.0	63.0	8.2	80.2	13.0	22.9	12.6
			75.8	10.0	59.5	17.7	90.3	11.5	69.9	8.2	74.9	10.8	9.1	10.5
**DJ694 + DJ1077 + DJ1079 + 2.1 + 3.3**														
DJ694;3.3+DJ1077	UAS-grim;2.1+DJ1079(13)	11/27/2007	76.0	10.8	80.9	12.7	100.0	0.0	69.0	15.1	71.0	7.9	0.0	0.0
		12/18/2007	76.0	8.6	50.8	8.9	100.0	0.0	64.0	8.6	54.0	10.5	0.0	0.0
DJ694;2.1+DJ1079(13)	UAS-grim;3.3+DJ1077	11/20/2007	49.0	6.8	47.6	9.4	81.7	13.7	31.0	10.0	89.8	13.5	53.5	21.8
		12/11/2007	78.0	6.9	55.4	5.6	100.0	0.0	59.0	13.2	60.8	5.6	7.8	9.0
DJ694;3.3+DJ1077	UAS-grim;2.1+DJ1079(14)	11/13/2007	79.0	6.0	90.2	5.1	91.5	5.7	78.0	7.7	86.1	16.4	1.4	2.8
		12/04/2007	92.0	7.3	77.4	11.0	98.7	2.6	77.0	8.9	77.0	10.8	20.3	4.1
DJ694;2.1+DJ1079(14)	UAS-grim;3.3+DJ1077	11/20/2007	54.0	12.4	63.5	18.1	97.9	4.2	48.0	5.7	79.7	23.7	29.1	5.4
		12/11/2007	81.0	6.8	49.4	5.7	100.0	0.0	42.0	10.1	67.9	25.1	22.9	20.8
DJ694;2.1+DJ1077	UAS-grim;3.3+DJ1079	11/13/2007	63.0	11.5	81.0	8.4	87.3	14.9	64.0	8.6	83.7	13.7	22.8	13.4
		12/04/2007	74.0	6.9	88.4	8.6	96.7	6.7	64.0	7.3	92.6	7.4	32.1	7.1
			72.2	13.1	68.5	16.9	95.4	6.4	59.6	15.1	76.3	12.7	19.0	17.2
**DJ694 + 2 copies DJ1077 + 2 copies 3.3**														
DJ694;3.3+DJ1077	UAS-grim;3.3+DJ1077	11/20/2007	52.0	7.3	82.9	20.3	100.0	0.0	56.0	9.8	73.7	17.3	56.6	25.5
		12/11/2007	81.0	7.6	58.7	17.3	98.4	3.3	60.0	7.3	75.3	3.5	22.5	11.6
			66.5	20.5	70.8	17.1	99.2	1.2	58.0	2.8	74.5	1.1	39.5	24.1
**DJ694 + 2 copies DJ1079 + 2.1 + 3.3**														
DJ694;2.1+DJ1079(13)	UAS-grim;3.3+DJ1079	11/13/2007	78.0	12.0	41.3	5.8	81.8	10.2	83.0	5.0	51.4	22.0	33.6	38.5
		12/04/2007	69.0	10.5	78.5	5.9	97.8	4.5	69.0	2.0	88.5	12.6	7.2	8.4
DJ694;2.1+DJ1079(14)	UAS-grim;3.3+DJ1079	11/13/2007	59.0	8.2	91.3	11.0	86.6	11.7	56.0	17.0	74.6	17.4	27.3	27.4
		12/04/2007	59.0	8.2	59.4	11.2	90.2	6.6	53.0	11.0	65.7	5.9	20.8	12.0
			66.3	9.1	67.6	21.9	89.1	6.7	65.3	13.7	70.0	15.6	22.2	11.3
**DJ694 + DJ1077 + DJ1079 + 2 copies 3.3**														
DJ694;3.3+DJ1077	UAS-grim;3.3+DJ1079	11/13/2007	58.0	10.1	98.4	3.3	93.5	5.5	59.0	10.5	74.1	12.6	31.9	15.8
		12/04/2007	62.0	5.2	80.0	13.0	100.0	0.0	61.0	7.6	84.0	7.2	13.9	4.4
			60.0	2.8	89.2	13.0	96.7	4.6	60.0	1.4	79.0	7.0	22.9	12.7

Supplemental Table 2: UAS-grim lethality assay statistical analysis (t-tests).

		hatching		pupation		eclosion	
		-tet	+tet	-tet	+tet	-tet	+tet
**1 copy DJ146**							
**w1118;UAS-grim/+ compared to:**							
w1118;UAS-grim/+	;DJ1077 /+	8.41E-01	UD	2.29E-01	UD	7.88E-01	UD
""	;DJ1078 /+	5.84E-01	UD	6.33E-01	UD	1.66E-01	UD
""	;DJ1079 /+	1.40E-01	UD	1.51E-01	UD	9.33E-01	UD
""	;DJ1080 /+	8.59E-01	UD	8.33E-01	UD	3.09E-01	UD
""	;DJ1081 /+	6.04E-01	UD	6.51E-01	UD	9.68E-01	UD
""	;DJ1083 /+	3.72E-01	UD	2.20E-01	UD	2.65E-01	UD
**w1118;UAS-grim/+ compared to:**							
DJ694/ UAS-grim	;DJ1077 /+	1.85E-07	1.28E-06	9.53E-08	5.76E-24	8.59E-40	3.27E-37
""	;DJ1078 /+	4.08E-07	6.83E-04	2.43E-09	3.34E-19	1.72E-36	2.61E-18
""	;DJ1079 /+	1.45E-05	4.13E-03	1.03E-07	1.37E-19	1.11E-37	8.78E-39
""	;DJ1080 /+	8.37E-05	1.18E-04	4.31E-09	6.22E-20	1.11E-37	8.78E-39
""	;DJ1081 /+	1.17E-07	7.36E-07	1.69E-15	8.27E-16	2.28E-12	1.43E-04
""	;DJ1083 /+	7.35E-05	6.17E-04	1.30E-07	3.43E-17	3.99E-34	3.11E-35
**DJ694/ UAS-grim compared to:**							
DJ694/ UAS-grim	;DJ1077 /+	9.62E-02	2.44E-01	1.92E-23	8.40E-04	1.62E-03	7.29E-03
""	;DJ1078 /+	2.82E-01	2.31E-02	1.59E-14	4.11E-18	1.97E-04	3.03E-04
""	;DJ1079 /+	6.48E-02	1.07E-02	2.42E-05	3.03E-04	~ 1	~ 1
""	;DJ1080 /+	1.09E-02	4.06E-02	3.14E-14	3.03E-04	~ 1	~ 1
""	;DJ1081 /+	4.90E-01	7.52E-01	4.71E-25	6.72E-22	5.43E-19	3.23E-23
""	;DJ1083 /+	4.80E-02	7.90E-02	4.52E-11	~ 1	~ 1	~ 1
**DJ694/ UAS-grim; DJ1077/+ compared to:**							
DJ694/ UAS-grim	;DJ1078 /+	8.70E-01	4.22E-01	3.38E-01	1.38E-02	9.03E-01	3.17E-01
""	;DJ1079 /+	7.33E-01	3.30E-01	2.52E-01	5.32E-01	4.19E-01	4.82E-01
""	;DJ1080 /+	3.78E-01	5.04E-01	3.54E-01	4.90E-01	4.19E-01	4.82E-01
""	;DJ1081 /+	7.70E-01	7.72E-01	5.61E-02	3.18E-03	3.59E-03	7.49E-04
""	;DJ1083 /+	5.29E-01	5.56E-01	4.11E-01	4.79E-01	5.20E-01	5.76E-01
**DJ694/ UAS-grim; DJ1078/+ compared to:**							
DJ694/ UAS-grim	;DJ1079 /+	6.74E-01	8.07E-01	7.05E-01	2.31E-01	3.74E-01	3.74E-01
""	;DJ1080 /+	3.60E-01	8.32E-01	9.88E-01	1.71E-02	3.74E-01	3.74E-01
""	;DJ1081 /+	9.05E-01	3.04E-01	4.23E-01	1.48E-01	3.21E-02	6.87E-02
""	;DJ1083 /+	5.04E-01	8.75E-01	9.58E-01	4.96E-02	4.95E-01	4.95E-01
**DJ694/ UAS-grim; DJ1079/+ compared to:**							
DJ694/ UAS-grim	;DJ1080 /+	6.16E-01	6.45E-01	7.00E-01	4.20E-01	~ 1	~ 1
""	;DJ1081 /+	6.07E-01	2.52E-01	8.81E-01	8.18E-02	2.87E-02	1.01E-02
""	;DJ1083 /+	7.56E-01	6.95E-01	8.03E-01	4.95E-01	~ 1	~ 1
**DJ694/ UAS-grim; DJ1080/+ compared to:**							
DJ694/ UAS-grim	;DJ1081 /+	2.78E-01	3.39E-01	4.27E-01	1.42E-02	2.87E-02	1.01E-02
""	;DJ1083 /+	8.11E-01	9.60E-01	9.50E-01	4.95E-01	~ 1	~ 1

		hatching		pupation		eclosion	
		-tet	+tet	-tet	+tet	-tet	+tet
**DJ694/ UAS-grim; DJ1081/+ compared to:**							
DJ694/ UAS-grim	;DJ1083 /+	4.04E-01	3.90E-01	6.04E-01	5.87E-02	9.80E-02	4.93E-02
**1 copy DJ147**							
**w1118;UAS-grim/+ compared to:**							
w1118; UAS-grim/+ **OR** w1118; DJ694/+	;2.1 /+	5.93E-04	7.73E-03	5.10E-01	2.48E-01	2.90E-01	8.79E-02
""	;3.2 /+	4.16E-04	4.57E-05	7.39E-04	3.13E-02	1.20E-01	3.83E-01
""	;3.3 /+	5.44E-05	2.67E-02	1.43E-02	5.46E-01	3.33E-03	8.07E-02
**w1118;UAS-grim/+ compared to:**							
DJ694/ UAS-grim	;2.1 /+	2.52E-07	1.21E-07	1.10E-15	8.75E-19	7.76E-42	9.75E-42
""	;3.2 /+	1.92E-06	5.67E-07	4.61E-23	5.20E-22	7.76E-42	9.75E-42
""	;3.3 /+	3.23E-11	1.26E-11	4.76E-20	2.47E-21	9.31E-42	8.04E-47
**DJ694/ UAS-grim compared to:**							
DJ694/ UAS-grim	;2.1 /+	9.94E-01	8.45E-01	1.78E-05	8.46E-06	~ 1	~ 1
""	;3.2 /+	1.08E-01	2.96E-01	5.05E-20	3.03E-04	~ 1	~ 1
""	;3.3 /+	9.42E-01	1.93E-01	2.52E-05	1.58E-03	1.24E-02	~ 1
""	;4 /+	1.08E-02	4.93E-02	1.67E-14	2.34E-06	~ 1	~ 1
**DJ694/ UAS-grim; 2.1/+ compared to:**							
DJ694/ UAS-grim	;3.2 /+	5.24E-01	6.54E-01	7.75E-01	2.99E-01	~ 1	~ 1
""	;3.3 /+	9.73E-01	4.67E-01	7.57E-01	7.46E-01	4.47E-01	~ 1
""	;4 /+	3.05E-01	3.42E-01	5.95E-01	5.47E-01	~ 1	~ 1
**DJ694/ UAS-grim; 3.2/+ compared to:**							
DJ694/ UAS-grim	;3.3 /+	3.29E-01	2.27E-01	9.90E-01	4.76E-01	5.16E-01	~ 1
""	;4 /+	5.33E-01	4.76E-01	2.72E-01	5.07E-01	~ 1	~ 1
**DJ694/ UAS-grim; 3.3/+ compared to:**							
DJ694/ UAS-grim	;4 /+	1.03E-01	1.01E-01	6.57E-01	7.30E-01	6.04E-01	~ 1
**2 copies DJ146**							
**w1118;UAS-grim/+ compared to:**							
w1118; UAS-grim/+ **OR** w1118; DJ694/+	;DJ1077/DJ1080	3.97E-01	3.29E-02	3.83E-01	6.55E-01	1.59E-01	1.72E-01
"'	;DJ1077/DJ1083	7.24E-01	5.49E-01	6.00E-01	1.19E-01	2.84E-01	7.34E-01
"'	;DJ1080/DJ1083	3.53E-02	3.33E-03	2.41E-02	6.18E-02	9.15E-02	9.02E-01
"'	;DJ1079/DJ180	3.75E-01	8.92E-01	7.76E-01	7.50E-01	3.16E-01	5.62E-01
**w1118;UAS-grim/+ compared to:**							
DJ694/ UAS-grim	;DJ1077/DJ1077	8.43E-03	1.07E-04	7.78E-05	1.36E-05	5.93E-10	8.78E-39
"'	;DJ1077/DJ1078	6.15E-01	7.96E-01	7.29E-04	4.87E-07	4.06E-35	3.11E-35
"'	;DJ1078/DJ1078	UD	UD	UD	UD	UD	UD
"'	;DJ1077/DJ1079	2.71E-01	2.91E-01	9.16E-05	1.60E-05	1.51E-11	8.78E-39
"'	;DJ1078/DJ1079	8.52E-01	6.06E-01	6.83E-08	3.96E-11	3.33E-35	3.11E-35
"'	;DJ1077/DJ1080	9.24E-03	2.70E-03	8.95E-08	8.97E-12	2.99E-21	8.04E-47

		hatching		pupation		eclosion	
		-tet	+tet	-tet	+tet	-tet	+tet
'''	;DJ1078/DJ1080	4.50E-01	5.20E-01	3.71E-05	8.99E-14	3.33E-35	3.11E-35
'''	;DJ1077/DJ1081	UD	UD	UD	UD	UD	UD
'''	;DJ1078/DJ1081	UD	UD	UD	UD	UD	UD
'''	;DJ1077/DJ1083	4.32E-08	4.89E-06	2.49E-08	7.54E-22	1.57E-24	9.75E-42
'''	;DJ1078/DJ1083	UD	UD	UD	UD	UD	UD
'''	;DJ1079/DJ1079	UD	UD	UD	UD	UD	UD
'''	;DJ1079/DJ1080	5.07E-01	9.75E-03	6.37E-05	2.84E-10	2.78E-20	1.06E-07
'''	;DJ1080/DJ1080	8.86E-02	7.95E-02	2.96E-02	1.35E-13	1.57E-33	3.11E-35
'''	;DJ1079/DJ1081	UD	UD	UD	UD	UD	UD
'''	;DJ1080/DJ1081	UD	UD	UD	UD	UD	UD
'''	;DJ1079/DJ1083	UD	UD	UD	UD	UD	UD
'''	;DJ1080/DJ1083	2.55E-08	9.16E-06	1.06E-08	3.43E-22	3.33E-39	9.75E-42
**DJ694/ UAS-grim compared to:**							
DJ694/ UAS-grim	;DJ1077/DJ1077	1.77E-03	7.89E-01	3.82E-34	2.22E-09	3.38E-09	~ 1
'''	;DJ1077/DJ1078	6.85E-04	1.65E-03	1.79E-49	8.74E-51	1.14E-06	~ 1
'''	;DJ1078/DJ1078	UD	UD	UD	UD	UD	UD
'''	;DJ1077/DJ1079	4.46E-04	5.47E-04	2.51E-25	2.75E-10	1.97E-04	~ 1
'''	;DJ1078/DJ1079	1.33E-03	2.38E-03	4.45E-15	1.44E-06	~ 1	~ 1
'''	;DJ1077/DJ1080	4.42E-04	2.20E-04	8.01E-26	1.63E-11	5.50E-06	~ 1
'''	;DJ1078/DJ1080	5.35E-04	2.57E-03	4.10E-34	9.88E-09	~ 1	~ 1
'''	;DJ1077/DJ1081	UD	UD	UD	UD	UD	UD
'''	;DJ1078/DJ1081	UD	UD	UD	UD	UD	UD
'''	;DJ1077/DJ1083	6.74E-01	5.46E-01	2.51E-12	5.10E-06	3.41E-05	~ 1
'''	;DJ1078/DJ1083	UD	UD	UD	UD	UD	UD
'''	;DJ1079/DJ1079	UD	UD	UD	UD	UD	UD
'''	;DJ1079/DJ1080	2.04E-04	5.17E-03	7.62E-32	1.73E-08	1.97E-04	3.03E-04
'''	;DJ1080/DJ1080	5.53E-03	9.61E-03	8.42E-51	2.58E-43	1.14E-06	~ 1
'''	;DJ1079/DJ1081	UD	UD	UD	UD	UD	UD
'''	;DJ1080/DJ1081	UD	UD	UD	UD	UD	UD
'''	;DJ1079/DJ1083	UD	UD	UD	UD	UD	UD
'''	;DJ1080/DJ1083	9.00E-01	3.45E-01	4.29E-12	~ 1	1.71E-03	~ 1
**DJ694/ UAS-grim; DJ1077/DJ1077 compared to:**							
DJ694/ UAS-grim	;DJ1077/DJ1078	1.02E-01	3.69E-01	8.64E-01	7.89E-01	2.49E-01	~ 1
'''	;DJ1078/DJ1078	UD	UD	UD	UD	UD	UD
'''	;DJ1077/DJ1079	5.54E-01	2.90E-01	8.28E-01	9.57E-01	6.48E-01	~ 1
'''	;DJ1078/DJ1079	2.16E-01	3.88E-01	2.86E-01	5.13E-01	2.45E-01	~ 1
'''	;DJ1077/DJ1080	8.26E-01	2.27E-01	4.79E-01	5.91E-01	2.41E-01	~ 1
'''	;DJ1078/DJ1080	2.16E-01	3.90E-01	2.86E-01	3.99E-01	2.45E-01	~ 1
'''	;DJ1077/DJ1081	UD	UD	UD	UD	UD	UD
'''	;DJ1078/DJ1081	UD	UD	UD	UD	UD	UD

		hatching		pupation		eclosion	
		-tet	+tet	-tet	+tet	-tet	+tet
'''	;DJ1077/DJ1083	1.49E-01	9.31E-01	2.37E-01	7.15E-02	2.15E-01	~ 1
'''	;DJ1078/DJ1083	UD	UD	UD	UD	UD	UD
'''	;DJ1079/DJ1079	UD	UD	UD	UD	UD	UD
'''	;DJ1079/DJ1080	1.45E-01	4.06E-01	9.36E-01	5.95E-01	3.42E-01	3.74E-01
'''	;DJ1080/DJ1080	6.94E-01	4.66E-01	4.59E-01	5.09E-01	2.76E-01	~ 1
'''	;DJ1079/DJ1081	UD	UD	UD	UD	UD	UD
'''	;DJ1080/DJ1081	UD	UD	UD	UD	UD	UD
'''	;DJ1079/DJ1083	UD	UD	UD	UD	UD	UD
'''	;DJ1080/DJ1083	1.38E-01	8.46E-01	2.12E-01	6.35E-02	8.80E-02	~ 1
**DJ694/ UAS-grim; DJ1077/DJ1078 compared to:**							
DJ694/ UAS-grim	;DJ1078/DJ1078	UD	UD	UD	UD	UD	UD
'''	;DJ1077/DJ1079	5.63E-01	6.98E-01	7.71E-01	8.23E-01	5.02E-01	~ 1
'''	;DJ1078/DJ1079	6.33E-01	8.92E-01	3.70E-01	2.45E-01	4.23E-01	~ 1
'''	;DJ1077/DJ1080	4.30E-01	3.33E-01	4.66E-01	2.79E-01	3.27E-01	~ 1
'''	;DJ1078/DJ1080	7.64E-01	7.61E-01	6.06E-01	7.99E-02	4.23E-01	~ 1
'''	;DJ1077/DJ1081	UD	UD	UD	UD	UD	UD
'''	;DJ1078/DJ1081	UD	UD	UD	UD	UD	UD
'''	;DJ1077/DJ1083	1.30E-01	2.22E-01	3.07E-01	1.20E-05	4.23E-01	~ 1
'''	;DJ1078/DJ1083	UD	UD	UD	UD	UD	UD
'''	;DJ1079/DJ1079	UD	UD	UD	UD	UD	UD
'''	;DJ1079/DJ1080	2.72E-01	3.06E-01	8.19E-01	3.22E-01	5.08E-01	4.95E-01
'''	;DJ1080/DJ1080	1.97E-01	2.62E-01	3.41E-01	5.61E-03	4.83E-01	~ 1
'''	;DJ1079/DJ1081	UD	UD	UD	UD	UD	UD
'''	;DJ1080/DJ1081	UD	UD	UD	UD	UD	UD
'''	;DJ1079/DJ1083	UD	UD	UD	UD	UD	UD
'''	;DJ1080/DJ1083	1.28E-01	2.29E-01	2.81E-01	1.23E-06	6.41E-01	~ 1
**DJ694/ UAS-grim; DJ1078/DJ1078 compared to:**							
DJ694/ UAS-grim	;DJ1077/DJ1079	UD	UD	UD	UD	UD	UD
'''	;DJ1078/DJ1079	UD	UD	UD	UD	UD	UD
'''	;DJ1077/DJ1080	UD	UD	UD	UD	UD	UD
'''	;DJ1078/DJ1080	UD	UD	UD	UD	UD	UD
'''	;DJ1077/DJ1081	UD	UD	UD	UD	UD	UD
'''	;DJ1078/DJ1081	UD	UD	UD	UD	UD	UD
'''	;DJ1077/DJ1083	UD	UD	UD	UD	UD	UD
'''	;DJ1078/DJ1083	UD	UD	UD	UD	UD	UD
'''	;DJ1079/DJ1079	UD	UD	UD	UD	UD	UD
'''	;DJ1079/DJ1080	UD	UD	UD	UD	UD	UD
'''	;DJ1080/DJ1080	UD	UD	UD	UD	UD	UD
'''	;DJ1079/DJ1081	UD	UD	UD	UD	UD	UD
'''	;DJ1080/DJ1081	UD	UD	UD	UD	UD	UD

		hatching		pupation		eclosion	
		-tet	+tet	-tet	+tet	-tet	+tet
'''	;DJ1079/DJ1083	UD	UD	UD	UD	UD	UD
'''	;DJ1080/DJ1083	UD	UD	UD	UD	UD	UD
**DJ694/ UAS-grim; DJ1077/DJ1079 compared to:**							
DJ694/ UAS-grim	;DJ1078/DJ1079	7.51E-01	8.40E-01	4.08E-01	4.69E-01	4.95E-01	~ 1
'''	;DJ1077/DJ1080	5.83E-01	4.20E-01	6.90E-01	5.24E-01	7.08E-01	~ 1
'''	;DJ1078/DJ1080	5.01E-01	8.57E-01	9.60E-01	3.56E-01	4.95E-01	~ 1
'''	;DJ1077/DJ1081	UD	UD	UD	UD	UD	UD
'''	;DJ1078/DJ1081	UD	UD	UD	UD	UD	UD
'''	;DJ1077/DJ1083	1.13E-01	1.67E-01	3.19E-01	5.48E-02	5.87E-01	~ 1
'''	;DJ1078/DJ1083	UD	UD	UD	UD	UD	UD
'''	;DJ1079/DJ1079	UD	UD	UD	UD	UD	UD
'''	;DJ1079/DJ1080	8.02E-01	4.06E-01	8.80E-01	5.45E-01	7.06E-01	3.74E-01
'''	;DJ1080/DJ1080	7.91E-01	5.63E-01	5.23E-01	4.58E-01	5.48E-01	~ 1
'''	;DJ1079/DJ1081	UD	UD	UD	UD	UD	UD
'''	;DJ1080/DJ1081	UD	UD	UD	UD	UD	UD
'''	;DJ1079/DJ1083	UD	UD	UD	UD	UD	UD
'''	;DJ1080/DJ1083	1.06E-01	1.79E-01	2.89E-01	4.84E-02	3.20E-01	~ 1
**DJ694/ UAS-grim; DJ1078/DJ1079 compared to:**							
DJ694/ UAS-grim	;DJ1077/DJ1080	5.20E-01	4.08E-01	3.69E-01	5.19E-01	3.15E-01	~ 1
'''	;DJ1078/DJ1080	4.93E-01	9.50E-01	4.84E-01	8.33E-01	~ 1	~ 1
'''	;DJ1077/DJ1081	UD	UD	UD	UD	UD	UD
'''	;DJ1078/DJ1081	UD	UD	UD	UD	UD	UD
'''	;DJ1077/DJ1083	1.53E-01	2.42E-01	9.46E-01	2.02E-01	4.06E-01	~ 1
'''	;DJ1078/DJ1083	UD	UD	UD	UD	UD	UD
'''	;DJ1079/DJ1079	UD	UD	UD	UD	UD	UD
'''	;DJ1079/DJ1080	7.48E-01	4.02E-01	3.13E-01	7.66E-01	4.95E-01	4.95E-01
'''	;DJ1080/DJ1080	3.96E-01	4.85E-01	2.94E-01	9.15E-01	4.23E-01	~ 1
'''	;DJ1079/DJ1081	UD	UD	UD	UD	UD	UD
'''	;DJ1080/DJ1081	UD	UD	UD	UD	UD	UD
'''	;DJ1079/DJ1083	UD	UD	UD	UD	UD	UD
'''	;DJ1080/DJ1083	2.07E-01	2.73E-01	7.47E-01	2.62E-01	5.41E-01	~ 1
**DJ694/ UAS-grim; DJ1077/DJ1080 compared to:**							
DJ694/ UAS-grim	;DJ1078/DJ1080	4.00E-01	4.12E-01	7.54E-01	3.27E-01	3.15E-01	~ 1
'''	;DJ1077/DJ1081	UD	UD	UD	UD	UD	UD
'''	;DJ1078/DJ1081	UD	UD	UD	UD	UD	UD
'''	;DJ1077/DJ1083	1.52E-01	1.43E-01	2.71E-01	2.27E-02	6.77E-01	~ 1
'''	;DJ1078/DJ1083	UD	UD	UD	UD	UD	UD
'''	;DJ1079/DJ1079	UD	UD	UD	UD	UD	UD
'''	;DJ1079/DJ1080	4.77E-01	9.92E-01	5.34E-01	7.87E-01	8.25E-01	1.70E-01
'''	;DJ1080/DJ1080	7.60E-01	7.71E-01	2.61E-01	5.45E-01	4.09E-01	~ 1

		hatching		pupation		eclosion	
		-tet	+tet	-tet	+tet	-tet	+tet
'''	;DJ1079/DJ1081	UD	UD	UD	UD	UD	UD
'''	;DJ1080/DJ1081	UD	UD	UD	UD	UD	UD
'''	;DJ1079/DJ1083	UD	UD	UD	UD	UD	UD
'''	;DJ1080/DJ1083	1.27E-01	1.76E-01	2.35E-01	1.78E-02	1.94E-01	~ 1
**DJ694/ UAS-grim; DJ1078/DJ1080 compared to:**							
DJ694/ UAS-grim	;DJ1077/DJ1081	UD	UD	UD	UD	UD	UD
'''	;DJ1078/DJ1081	UD	UD	UD	UD	UD	UD
'''	;DJ1077/DJ1083	1.23E-01	2.42E-01	4.10E-01	1.34E-01	4.06E-01	~ 1
'''	;DJ1078/DJ1083	UD	UD	UD	UD	UD	UD
'''	;DJ1079/DJ1079	UD	UD	UD	UD	UD	UD
'''	;DJ1079/DJ1080	1.54E-01	3.56E-01	8.12E-01	5.79E-01	4.95E-01	4.95E-01
'''	;DJ1080/DJ1080	1.44E-01	1.50E-01	3.37E-01	5.60E-01	4.23E-01	~ 1
'''	;DJ1079/DJ1081	UD	UD	UD	UD	UD	UD
'''	;DJ1080/DJ1081	UD	UD	UD	UD	UD	UD
'''	;DJ1079/DJ1083	UD	UD	UD	UD	UD	UD
'''	;DJ1080/DJ1083	1.21E-01	2.52E-01	3.79E-01	9.61E-02	5.41E-01	~ 1
**DJ694/ UAS-grim; DJ1077/DJ1081 compared to:**							
DJ694/ UAS-grim	;DJ1078/DJ1081	UD	UD	UD	UD	UD	UD
'''	;DJ1077/DJ1083	UD	UD	UD	UD	UD	UD
'''	;DJ1078/DJ1083	UD	UD	UD	UD	UD	UD
'''	;DJ1079/DJ1079	UD	UD	UD	UD	UD	UD
'''	;DJ1079/DJ1080	UD	UD	UD	UD	UD	UD
'''	;DJ1080/DJ1080	UD	UD	UD	UD	UD	UD
'''	;DJ1079/DJ1081	UD	UD	UD	UD	UD	UD
'''	;DJ1080/DJ1081	UD	UD	UD	UD	UD	UD
'''	;DJ1079/DJ1083	UD	UD	UD	UD	UD	UD
'''	;DJ1080/DJ1083	UD	UD	UD	UD	UD	UD
**DJ694/ UAS-grim; DJ1078/DJ1081 compared to:**							
DJ694/ UAS-grim	;DJ1077/DJ1083	UD	UD	UD	UD	UD	UD
'''	;DJ1078/DJ1083	UD	UD	UD	UD	UD	UD
'''	;DJ1079/DJ1079	UD	UD	UD	UD	UD	UD
'''	;DJ1079/DJ1080	UD	UD	UD	UD	UD	UD
'''	;DJ1080/DJ1080	UD	UD	UD	UD	UD	UD
'''	;DJ1079/DJ1081	UD	UD	UD	UD	UD	UD
'''	;DJ1080/DJ1081	UD	UD	UD	UD	UD	UD
'''	;DJ1079/DJ1083	UD	UD	UD	UD	UD	UD
'''	;DJ1080/DJ1083	UD	UD	UD	UD	UD	UD
**DJ694/ UAS-grim; DJ1077/DJ1083 compared to:**							
DJ694/ UAS-grim	;DJ1078/DJ1083	UD	UD	UD	UD	UD	UD
'''	;DJ1079/DJ1079	UD	UD	UD	UD	UD	UD

		hatching		pupation		eclosion	
		-tet	+tet	-tet	+tet	-tet	+tet
'''	;DJ1079/DJ1080	8.38E-02	2.86E-01	2.56E-01	9.83E-02	9.04E-01	2.86E-01
'''	;DJ1080/DJ1080	2.16E-01	3.26E-01	2.29E-01	2.78E-04	5.43E-01	~ 1
'''	;DJ1079/DJ1081	UD	UD	UD	UD	UD	UD
'''	;DJ1080/DJ1081	UD	UD	UD	UD	UD	UD
'''	;DJ1079/DJ1083	UD	UD	UD	UD	UD	UD
'''	;DJ1080/DJ1083	8.97E-01	8.88E-01	9.51E-01	1.53E-01	2.95E-01	~ 1
**DJ694/ UAS-grim; DJ1078/DJ1083 compared to:**							
DJ694/ UAS-grim	;DJ1079/DJ1079	UD	UD	UD	UD	UD	UD
'''	;DJ1079/DJ1080	UD	UD	UD	UD	UD	UD
'''	;DJ1080/DJ1080	UD	UD	UD	UD	UD	UD
'''	;DJ1079/DJ1081	UD	UD	UD	UD	UD	UD
'''	;DJ1080/DJ1081	UD	UD	UD	UD	UD	UD
'''	;DJ1079/DJ1083	UD	UD	UD	UD	UD	UD
'''	;DJ1080/DJ1083	UD	UD	UD	UD	UD	UD
**DJ694/ UAS-grim; DJ1079/DJ1079 compared to:**							
DJ694/ UAS-grim	;DJ1079/DJ1080	UD	UD	UD	UD	UD	UD
'''	;DJ1080/DJ1080	UD	UD	UD	UD	UD	UD
'''	;DJ1079/DJ1081	UD	UD	UD	UD	UD	UD
'''	;DJ1080/DJ1081	UD	UD	UD	UD	UD	UD
'''	;DJ1079/DJ1083	UD	UD	UD	UD	UD	UD
'''	;DJ1080/DJ1083	UD	UD	UD	UD	UD	UD
**DJ694/ UAS-grim; DJ1079/DJ1080 compared to:**							
DJ694/ UAS-grim	;DJ1080/DJ1080	3.01E-01	7.22E-01	4.66E-01	7.94E-01	5.99E-01	4.95E-01
'''	;DJ1079/DJ1081	UD	UD	UD	UD	UD	UD
'''	;DJ1080/DJ1081	UD	UD	UD	UD	UD	UD
'''	;DJ1079/DJ1083	UD	UD	UD	UD	UD	UD
'''	;DJ1080/DJ1083	8.03E-02	3.18E-01	2.29E-01	8.26E-02	3.56E-01	2.86E-01
**DJ694/ UAS-grim; DJ1080/DJ1080 compared to:**							
DJ694/ UAS-grim	;DJ1079/DJ1081	UD	UD	UD	UD	UD	UD
'''	;DJ1080/DJ1081	UD	UD	UD	UD	UD	UD
'''	;DJ1079/DJ1083	UD	UD	UD	UD	UD	UD
'''	;DJ1080/DJ1083	2.06E-01	3.47E-01	2.09E-01	9.28E-06	7.03E-01	~ 1
**DJ694/ UAS-grim; DJ1079/DJ1081 compared to:**							
DJ694/ UAS-grim	;DJ1080/DJ1081	UD	UD	UD	UD	UD	UD
'''	;DJ1079/DJ1083	UD	UD	UD	UD	UD	UD
'''	;DJ1080/DJ1083	UD	UD	UD	UD	UD	UD
**DJ694/ UAS-grim; DJ1080/DJ1081 compared to:**							
DJ694/ UAS-grim	;DJ1079/DJ1083	UD	UD	UD	UD	UD	UD
'''	;DJ1080/DJ1083	UD	UD	UD	UD	UD	UD
**DJ694/ UAS-grim; DJ1079/DJ1083 compared to:**							

		hatching		pupation		eclosion	
		-tet	+tet	-tet	+tet	-tet	+tet
DJ694/ UAS-grim	;DJ1080/DJ1083	UD	UD	UD	UD	UD	UD
**2 copies DJ147**							
**w1118;UAS-grim/+ compared to:**							
DJ694/ UAS-grim	;2.1/2.1	2.74E-02	1.13E-01	5.25E-01	1.93E-03	4.08E-16	1.59E-40
'''	;2.1/3.3	8.67E-05	7.19E-07	1.58E-09	2.03E-10	3.91E-17	2.32E-51
'''	;2.1/3.2	4.33E-03	2.10E-04	1.84E-11	6.92E-13	8.71E-13	2.40E-10
'''	;3.3/3.3	2.42E-12	2.53E-09	3.99E-12	5.01E-12	5.66E-18	9.75E-42
'''	;3.2/3.3	2.98E-06	1.36E-04	1.15E-13	2.54E-18	7.71E-37	8.78E-39
'''	;2.1/4	3.90E-03	1.70E-02	2.46E-13	1.94E-13	3.33E-35	3.11E-35
'''	;3.3/4	7.06E-01	5.57E-01	2.84E-17	3.72E-16	3.33E-35	3.11E-35
**DJ694/UAS-grim compared to:**							
DJ694/ UAS-grim	;2.1/2.1	2.73E-04	1.85E-04	3.41E-59	2.68E-38	3.36E-15	2.33E-03
'''	;2.1/3.3	6.09E-05	2.20E-03	1.70E-36	3.45E-20	1.55E-12	~ 1
'''	;2.1/3.2	7.65E-04	1.56E-02	6.33E-31	1.00E-33	5.98E-04	2.33E-03
'''	;3.3/3.3	7.34E-01	2.18E-01	1.24E-17	1.97E-09	2.46E-08	~ 1
'''	;3.2/3.3	4.25E-02	9.17E-02	2.31E-36	2.99E-36	1.97E-04	~ 1
'''	;2.1/4	1.77E-02	2.23E-02	4.22E-26	1.08E-68	~ 1	~ 1
'''	;3.3/4	1.57E-03	2.42E-03	8.19E-28	1.29E-17	~ 1	~ 1
**DJ694/UAS-grim; 2.1/2.1 compared to:**							
DJ694/ UAS-grim	;2.1/3.3	4.72E-01	7.73E-02	4.10E-03	3.29E-02	7.15E-01	1.67E-01
'''	;2.1/3.2	7.52E-01	2.32E-01	1.34E-03	1.13E-03	4.37E-01	3.76E-01
'''	;3.3/3.3	1.38E-02	3.93E-03	4.67E-03	1.14E-02	3.72E-01	3.56E-01
'''	;3.2/3.3	2.04E-01	2.06E-01	9.76E-05	3.88E-05	3.12E-02	4.37E-01
'''	;2.1/4	6.33E-01	4.43E-01	6.79E-04	1.10E-03	6.69E-02	5.41E-01
'''	;3.3/4	5.22E-01	6.74E-01	2.74E-05	3.76E-04	6.69E-02	5.41E-01
**DJ694/UAS-grim; 2.1/3.3 compared to:**							
DJ694/ UAS-grim	;2.1/3.2	7.06E-01	8.55E-01	8.29E-02	5.53E-01	4.55E-01	1.67E-01
'''	;3.3/3.3	1.50E-02	1.79E-01	1.48E-02	1.04E-01	4.73E-01	~ 1
'''	;3.2/3.3	4.45E-01	7.90E-01	8.63E-03	4.70E-03	3.55E-02	~ 1
'''	;2.1/4	8.99E-01	4.55E-01	4.64E-03	8.78E-02	6.53E-02	~ 1
'''	;3.3/4	2.70E-01	1.01E-01	1.90E-04	1.10E-02	6.53E-02	~ 1
**DJ694/UAS-grim; 2.1/3.2 compared to:**							
DJ694/ UAS-grim	;3.3/3.3	2.39E-02	2.37E-01	3.06E-01	2.44E-01	8.97E-01	3.56E-01
'''	;3.2/3.3	3.25E-01	7.69E-01	2.57E-01	2.96E-04	4.39E-01	4.37E-01
'''	;2.1/4	8.61E-01	6.66E-01	1.12E-01	1.40E-02	5.01E-01	5.41E-01
'''	;3.3/4	4.02E-01	2.72E-01	1.16E-02	1.65E-03	5.01E-01	5.41E-01
**DJ694/UAS-grim; 3.3/3.3 compared to:**							
DJ694/ UAS-grim	;3.2/3.3	1.47E-01	5.49E-01	7.80E-01	2.60E-01	1.74E-01	~ 1
'''	;2.1/4	6.96E-02	2.43E-02	7.02E-01	8.39E-01	2.39E-01	~ 1
'''	;3.3/4	2.35E-02	2.56E-03	1.84E-01	2.93E-01	2.39E-01	~ 1

		hatching		pupation		eclosion	
		-tet	+tet	-tet	+tet	-tet	+tet
**DJ694/UAS-grim; 3.2/3.3 compared to:**							
DJ694/ UAS-grim	;2.1/4	4.48E-01	5.73E-01	2.80E-01	7.99E-04	4.95E-01	~ 1
'''	;3.3/4	1.35E-01	2.95E-01	1.57E-02	1.69E-01	4.95E-01	~ 1
**DJ694/UAS-grim; 2.1/4 compared to:**							
DJ694/ UAS-grim	;3.3/4	1.66E-01	2.63E-01	1.78E-01	1.92E-02	~ 1	~ 1
**1 copy DJ146 + 1 copy DJ147**							
**w1118;UAS-grim/+ compared to:**							
DJ694/ UAS-grim	;3.3/1077	1.50E-03	2.37E-04	6.03E-10	5.17E-19	2.11E-04	9.75E-42
'''	;3.3/1080	7.66E-04	9.74E-03	7.39E-14	4.03E-19	8.35E-14	9.75E-42
'''	;3.3/1083	1.57E-02	1.39E-04	1.04E-06	5.59E-13	6.32E-29	3.11E-35
'''	;3.3/1079	3.61E-02	1.75E-02	7.64E-08	6.48E-13	6.93E-08	8.78E-39
'''	;4/1079	4.65E-03	4.45E-03	1.71E-16	1.76E-16	3.33E-35	3.11E-35
'''	;4/1083	3.76E-02	2.06E-02	2.16E-09	2.49E-16	3.33E-35	3.11E-35
'''	;2.1/1077	8.74E-01	3.06E-02	2.74E-09	1.53E-13	1.92E-11	2.24E-44
'''	;2.1/1080	3.41E-01	2.24E-01	1.16E-11	8.20E-16	7.63E-23	4.37E-44
'''	;2.1/1083	3.77E-01	8.60E-01	3.80E-06	1.23E-07	5.56E-26	3.11E-35
'''	;4/1077	1.57E-02	3.18E-02	9.92E-07	1.37E-16	9.06E-34	3.11E-35
'''	;4/1080	6.86E-08	4.18E-09	3.33E-18	5.44E-16	3.33E-35	3.11E-35
'''	;2.1/1079	7.88E-01	4.51E-01	2.84E-05	2.03E-12	8.12E-07	8.54E-41
**DJ694/UAS-grim compared to:**							
DJ694/ UAS-grim	;3.3/1077	3.90E-03	5.92E-02	2.16E-28	8.67E-30	5.39E-40	~ 1
'''	;3.3/1080	1.45E-02	1.90E-02	5.77E-29	3.05E-10	5.75E-17	~ 1
'''	;3.3/1083	1.04E-02	1.86E-01	3.43E-36	2.05E-84	9.98E-35	~ 1
'''	;3.3/1079	1.54E-03	3.25E-03	1.00E-27	1.90E-14	2.76E-13	~ 1
'''	;4/1079	1.58E-02	3.86E-02	3.15E-16	2.34E-06	~ 1	~ 1
'''	;4/1083	7.57E-03	2.31E-02	2.82E-20	7.62E-11	~ 1	~ 1
'''	;2.1/1077	1.50E-06	6.58E-05	6.66E-40	2.23E-30	4.15E-56	~ 1
'''	;2.1/1080	6.06E-06	1.38E-05	6.02E-50	3.73E-36	2.44E-20	7.29E-03
'''	;2.1/1083	4.73E-04	9.46E-04	1.31E-53	9.67E-31	1.53E-43	~ 1
'''	;4/1077	1.04E-02	1.55E-02	1.01E-43	2.34E-06	1.14E-06	~ 1
'''	;4/1080	2.46E-01	8.91E-01	5.97E-26	1.90E-37	~ 1	~ 1
'''	;2.1/1079	9.29E-06	4.01E-05	4.76E-29	2.68E-25	3.40E-10	2.33E-03
**DJ694/UAS-grim; 3.3/DJ1077 compared to:**							
DJ694/ UAS-grim	;3.3/1080	8.24E-01	7.90E-01	2.68E-01	2.25E-01	1.76E-02	~ 1
'''	;3.3/1083	7.65E-01	9.39E-01	6.53E-01	2.29E-02	1.43E-03	~ 1
'''	;3.3/1079	6.53E-01	4.61E-01	7.85E-01	2.33E-01	1.39E-01	~ 1
'''	;4/1079	8.63E-01	7.23E-01	2.48E-02	1.55E-02	2.44E-04	~ 1
'''	;4/1083	6.96E-01	6.36E-01	4.84E-01	1.68E-02	2.44E-04	~ 1
'''	;2.1/1077	1.30E-01	2.26E-01	2.76E-01	4.32E-04	5.72E-01	~ 1
'''	;2.1/1080	2.32E-01	1.41E-01	4.82E-01	1.51E-04	3.36E-04	4.07E-01

		hatching		pupation		eclosion	
		-tet	+tet	-tet	+tet	-tet	+tet
'''	;2.1/1083	2.87E-01	2.74E-01	4.58E-01	2.67E-03	4.15E-03	~ 1
'''	;4/1077	7.65E-01	5.68E-01	5.93E-01	1.12E-02	3.13E-04	~ 1
'''	;4/1080	4.26E-01	3.71E-01	1.12E-02	1.91E-02	2.44E-04	~ 1
'''	;2.1/1079	1.70E-01	1.58E-01	2.65E-01	4.40E-03	1.58E-01	3.56E-01
**DJ694/UAS-grim; 3.3/DJ1080 compared to:**							
DJ694/ UAS-grim	;3.3/1083	6.79E-01	7.83E-01	1.59E-01	8.34E-02	3.43E-01	~ 1
'''	;3.3/1079	5.62E-01	7.37E-01	2.19E-01	1.25E-01	7.44E-01	~ 1
'''	;4/1079	7.51E-01	9.47E-01	3.79E-02	3.42E-01	4.68E-02	~ 1
'''	;4/1083	6.28E-01	8.61E-01	9.89E-01	4.22E-01	4.68E-02	~ 1
'''	;2.1/1077	1.37E-01	5.09E-01	1.75E-02	6.09E-04	7.92E-03	~ 1
'''	;2.1/1080	2.14E-01	3.70E-01	2.13E-02	5.82E-04	3.58E-01	4.07E-01
'''	;2.1/1083	3.13E-01	4.75E-01	8.23E-02	7.87E-03	8.52E-01	~ 1
'''	;4/1077	6.79E-01	8.06E-01	1.23E-01	3.09E-01	5.67E-02	~ 1
'''	;4/1080	6.46E-01	3.52E-01	1.31E-02	6.62E-01	4.68E-02	~ 1
'''	;2.1/1079	1.81E-01	3.80E-01	4.99E-02	4.23E-03	8.52E-01	3.56E-01
**DJ694/UAS-grim; 3.3/DJ1083 compared to:**							
DJ694/ UAS-grim	;3.3/1079	8.79E-01	3.05E-01	8.70E-01	8.79E-01	4.08E-01	~ 1
'''	;4/1079	4.23E-01	5.84E-01	5.63E-02	2.90E-02	1.36E-02	~ 1
'''	;4/1083	9.55E-02	5.32E-01	4.47E-01	2.29E-02	1.36E-02	~ 1
'''	;2.1/1077	4.78E-03	1.04E-01	6.77E-01	3.08E-02	1.29E-05	~ 1
'''	;2.1/1080	1.84E-01	5.01E-02	8.89E-01	2.04E-02	5.47E-01	5.76E-01
'''	;2.1/1083	1.37E-03	1.90E-01	7.24E-01	7.57E-02	8.33E-02	~ 1
'''	;4/1077	~ 1	4.10E-01	9.38E-01	2.15E-02	2.76E-02	~ 1
'''	;4/1080	2.67E-02	3.10E-01	2.23E-02	1.60E-03	1.36E-02	~ 1
'''	;2.1/1079	6.70E-04	5.30E-02	5.88E-01	1.22E-01	5.10E-01	5.41E-01
**DJ694/UAS-grim; 3.3/DJ1079 compared to:**							
DJ694/ UAS-grim	;4/1079	7.42E-01	5.29E-01	4.54E-02	1.37E-01	1.30E-01	~ 1
'''	;4/1083	9.88E-01	8.19E-01	4.56E-01	1.51E-01	1.30E-01	~ 1
'''	;2.1/1077	1.92E-01	5.83E-01	5.28E-01	1.09E-01	1.03E-01	~ 1
'''	;2.1/1080	4.35E-01	2.14E-01	8.30E-01	2.26E-01	3.02E-01	4.82E-01
'''	;2.1/1083	3.25E-01	1.62E-01	6.76E-01	1.97E-01	7.12E-01	~ 1
'''	;4/1077	8.79E-01	9.33E-01	8.18E-01	1.30E-01	1.45E-01	~ 1
'''	;4/1080	1.52E-01	1.37E-02	2.40E-02	1.88E-01	1.30E-01	~ 1
'''	;2.1/1079	2.26E-01	1.11E-01	4.53E-01	2.39E-01	9.28E-01	4.37E-01
**DJ694/UAS-grim; 4/DJ1079 compared to:**							
DJ694/ UAS-grim	;4/1083	2.32E-01	8.11E-01	2.42E-01	8.09E-01	~ 1	~ 1
'''	;2.1/1077	3.58E-03	2.26E-01	1.20E-03	1.31E-03	5.78E-07	~ 1
'''	;2.1/1080	1.15E-01	6.82E-02	4.52E-04	3.88E-04	1.49E-02	5.76E-01
'''	;2.1/1083	1.72E-02	1.00E-01	3.31E-02	2.80E-02	4.42E-03	~ 1
'''	;4/1077	4.23E-01	4.88E-01	4.20E-02	9.33E-01	4.23E-01	~ 1

		hatching		pupation		eclosion	
		-tet	+tet	-tet	+tet	-tet	+tet
'''	;4/1080	4.72E-02	3.48E-02	5.96E-01	3.82E-01	~ 1	~ 1
'''	;2.1/1079	1.56E-03	2.30E-02	1.49E-02	9.42E-03	1.72E-01	5.41E-01
**DJ694/UAS-grim; 4/DJ1083 compared to:**							
DJ694/ UAS-grim	;2.1/1077	9.38E-03	4.83E-01	1.15E-01	1.42E-03	5.78E-07	~ 1
'''	;2.1/1080	2.75E-01	2.21E-01	1.40E-01	3.86E-04	1.49E-02	5.76E-01
'''	;2.1/1083	3.45E-03	2.74E-01	3.49E-01	2.75E-02	4.42E-03	~ 1
'''	;4/1077	9.55E-02	8.71E-01	4.09E-01	7.27E-01	4.23E-01	~ 1
'''	;4/1080	2.35E-02	8.31E-02	1.58E-01	4.59E-01	~ 1	~ 1
'''	;2.1/1079	1.37E-03	1.50E-01	1.96E-01	1.02E-02	1.72E-01	5.41E-01
**DJ694/UAS-grim; 2.1/DJ1077 compared to:**							
DJ694/ UAS-grim	;2.1/1080	3.69E-01	4.05E-01	3.61E-01	2.09E-01	4.75E-05	3.47E-01
'''	;2.1/1083	2.13E-01	1.77E-01	9.97E-01	4.21E-01	4.80E-05	~ 1
'''	;4/1077	4.78E-03	5.21E-01	7.25E-01	1.16E-03	1.40E-06	~ 1
'''	;4/1080	1.29E-04	1.26E-03	2.67E-04	1.71E-03	5.78E-07	~ 1
'''	;2.1/1079	8.33E-01	2.27E-01	5.99E-01	7.17E-01	1.28E-01	2.92E-01
**DJ694/UAS-grim; 2.1/DJ1080 compared to:**							
DJ694/ UAS-grim	;2.1/1083	2.61E-01	3.20E-01	2.97E-01	7.05E-02	4.43E-01	5.76E-01
'''	;4/1077	1.84E-01	1.59E-01	7.19E-01	3.12E-04	2.13E-02	5.76E-01
'''	;4/1080	4.05E-03	3.10E-04	2.53E-05	3.76E-04	1.49E-02	5.76E-01
'''	;2.1/1079	3.47E-01	6.70E-01	2.97E-01	5.39E-01	4.33E-01	5.65E-01
**DJ694/UAS-grim; 2.1/DJ1083 compared to:**							
DJ694/ UAS-grim	;4/1077	1.37E-03	6.36E-02	6.89E-01	2.59E-02	9.80E-03	~ 1
'''	;4/1080	9.19E-03	4.19E-03	5.30E-03	2.61E-02	4.42E-03	~ 1
'''	;2.1/1079	7.78E-02	2.38E-01	7.34E-01	3.99E-01	8.13E-01	5.41E-01
**DJ694/UAS-grim; 4/DJ1077 compared to:**							
DJ694/ UAS-grim	;4/1080	2.67E-02	2.96E-03	1.07E-02	2.95E-01	4.23E-01	~ 1
'''	;2.1/1079	6.70E-04	1.78E-02	6.11E-01	8.67E-03	1.93E-01	5.41E-01
**DJ694/UAS-grim; 4/DJ1080 compared to:**							
DJ694/ UAS-grim	;2.1/1079	1.82E-04	3.77E-05	7.86E-03	1.24E-02	1.72E-01	5.41E-01
**3 copies DJ146**							
**w1118;UAS-grim/+ compared to:**							
DJ694/ UAS-grim	;DJ1077/DJ1077,DJ1080	7.89E-02	5.55E-01	4.00E-02	1.14E-03	4.37E-14	3.11E-35
'''	;DJ1077/DJ1079,DJ1080	1.05E-05	5.50E-03	3.46E-05	4.98E-05	2.50E-08	1.94E-20
'''	;DJ1080/DJ1077,DJ1080	4.12E-01	1.98E-01	6.20E-01	1.78E-04	2.35E-13	3.11E-35
'''	;DJ1077/DJ1077,DJ1083	3.82E-12	7.09E-09	1.87E-13	4.00E-16	6.51E-10	3.11E-35
'''	;DJ1077/DJ1080,DJ1083	1.02E-07	1.17E-08	7.49E-06	1.04E-07	6.11E-11	1.50E-36
'''	:DJ1083/DJ1077,DJ1083	UD	UD	UD	UD	UD	UD
'''	;DJ1079/DJ1080,DJ1083	1.70E-03	2.67E-01	6.56E-01	1.37E-02	5.51E-14	3.11E-35
'''	;DJ1080/DJ1080,DJ1083	7.73E-03	2.01E-01	1.48E-02	3.39E-06	1.17E-26	3.11E-35
'''	;DJ1079/DJ1079,DJ1080	UD	UD	UD	UD	UD	UD

		hatching		pupation		eclosion	
		-tet	+tet	-tet	+tet	-tet	+tet
'''	;DJ1080/DJ1079,DJ1080	UD	UD	UD	UD	UD	UD
'''	;DJ1079/DJ1077,DJ1083	UD	UD	UD	UD	UD	UD
	**DJ694/UAS-grim compared to:**						
DJ694/ UAS-grim	;DJ1077/DJ1077,DJ1080	6.33E-03	2.39E-03	3.94E-46	1.50E-27	1.56E-13	~ 1
'''	;DJ1077/DJ1079,DJ1080	1.46E-02	5.35E-03	1.32E-46	3.90E-34	2.89E-18	3.03E-04
'''	;DJ1080/DJ1077,DJ1080	2.35E-03	5.51E-03	8.99E-46	1.99E-20	1.52E-16	~ 1
'''	;DJ1077/DJ1077,DJ1083	8.47E-01	6.75E-01	2.48E-20	5.71E-12	2.97E-39	~ 1
'''	;DJ1077/DJ1080,DJ1083	5.47E-01	8.88E-01	3.48E-12	1.77E-06	1.75E-13	3.03E-04
'''	:DJ1083/DJ1077,DJ1083	UD	UD	UD	UD	UD	UD
'''	;DJ1079/DJ1080,DJ1083	2.14E-02	4.67E-03	4.46E-43	3.32E-31	2.18E-33	~ 1
'''	;DJ1080/DJ1080,DJ1083	1.36E-02	6.24E-03	2.37E-63	2.98E-17	4.96E-49	~ 1
'''	;DJ1079/DJ1079,DJ1080	UD	UD	UD	UD	UD	UD
'''	;DJ1080/DJ1079,DJ1080	UD	UD	UD	UD	UD	UD
'''	;DJ1079/DJ1077,DJ1083	UD	UD	UD	UD	UD	UD
	**DJ694/UAS-grim;DJ1077/DJ1077,DJ1080 compared to:**						
DJ694/ UAS-grim	;DJ1077/DJ1079,DJ1080	2.24E-01	1.42E-01	2.53E-01	9.56E-01	4.58E-01	4.95E-01
'''	;DJ1080/DJ1077,DJ1080	6.06E-01	1.22E-01	4.34E-01	7.96E-01	8.30E-01	~ 1
'''	;DJ1077/DJ1077,DJ1083	4.62E-02	7.51E-02	5.64E-02	3.75E-02	2.23E-01	~ 1
'''	;DJ1077/DJ1080,DJ1083	2.63E-01	9.31E-02	4.12E-01	3.60E-01	8.01E-01	4.95E-01
'''	:DJ1083/DJ1077,DJ1083	UD	UD	UD	UD	UD	UD
'''	;DJ1079/DJ1080,DJ1083	4.90E-01	6.42E-01	4.64E-01	7.47E-01	3.52E-01	~ 1
'''	;DJ1080/DJ1080,DJ1083	6.67E-01	6.89E-01	7.77E-01	5.54E-01	8.80E-01	~ 1
'''	;DJ1079/DJ1079,DJ1080	UD	UD	UD	UD	UD	UD
'''	;DJ1080/DJ1079,DJ1080	UD	UD	UD	UD	UD	UD
'''	;DJ1079/DJ1077,DJ1083	UD	UD	UD	UD	UD	UD
	**DJ694/UAS-grim;DJ1077/DJ1079,DJ1080 compared to:**						
DJ694/ UAS-grim	;DJ1080/DJ1077,DJ1080	3.03E-02	3.02E-01	8.31E-02	7.34E-01	5.91E-01	4.95E-01
'''	;DJ1077/DJ1077,DJ1083	1.09E-02	5.07E-02	2.12E-02	4.05E-03	5.05E-01	4.95E-01
'''	;DJ1077/DJ1080,DJ1083	3.01E-01	8.65E-02	4.21E-01	2.39E-01	5.80E-01	4.56E-01
'''	:DJ1083/DJ1077,DJ1083	UD	UD	UD	UD	UD	UD
'''	;DJ1079/DJ1080,DJ1083	4.00E-01	3.42E-01	9.87E-02	5.78E-01	8.32E-01	4.95E-01
'''	;DJ1080/DJ1080,DJ1083	2.47E-01	5.18E-01	2.49E-01	4.12E-01	4.41E-01	4.95E-01
'''	;DJ1079/DJ1079,DJ1080	UD	UD	UD	UD	UD	UD
'''	;DJ1080/DJ1079,DJ1080	UD	UD	UD	UD	UD	UD
'''	;DJ1079/DJ1077,DJ1083	UD	UD	UD	UD	UD	UD
	**DJ694/UAS-grim;DJ1080/DJ1077,DJ1080 compared to:**						
DJ694/ UAS-grim	;DJ1077/DJ1077,DJ1083	1.06E-02	8.76E-02	4.55E-02	9.16E-02	2.64E-01	~ 1
'''	;DJ1077/DJ1080,DJ1083	2.00E-01	1.14E-01	3.23E-01	4.61E-01	9.80E-01	4.95E-01
'''	:DJ1083/DJ1077,DJ1083	UD	UD	UD	UD	UD	UD
'''	;DJ1079/DJ1080,DJ1083	2.13E-02	8.85E-01	9.83E-01	6.15E-01	4.39E-01	~ 1

		hatching		pupation		eclosion	
		-tet	+tet	-tet	+tet	-tet	+tet
'''	;DJ1080/DJ1080,DJ1083	1.01E-01	9.77E-01	2.61E-01	7.50E-01	8.79E-01	~ 1
'''	;DJ1079/DJ1079,DJ1080	UD	UD	UD	UD	UD	UD
'''	;DJ1080/DJ1079,DJ1080	UD	UD	UD	UD	UD	UD
'''	;DJ1079/DJ1077,DJ1083	UD	UD	UD	UD	UD	UD
**DJ694/UAS-grim;DJ1077/DJ1077,DJ1083 compared to:**							
DJ694/ UAS-grim	;DJ1077/DJ1080,DJ1083	6.97E-01	8.60E-01	5.59E-01	4.46E-01	2.83E-01	4.95E-01
'''	:DJ1083/DJ1077,DJ1083	UD	UD	UD	UD	UD	UD
'''	;DJ1079/DJ1080,DJ1083	2.08E-02	9.62E-02	4.85E-02	2.37E-02	4.21E-01	~ 1
'''	;DJ1080/DJ1080,DJ1083	2.27E-02	1.22E-01	5.22E-02	1.41E-01	4.09E-02	~ 1
'''	;DJ1079/DJ1079,DJ1080	UD	UD	UD	UD	UD	UD
'''	;DJ1080/DJ1079,DJ1080	UD	UD	UD	UD	UD	UD
'''	;DJ1079/DJ1077,DJ1083	UD	UD	UD	UD	UD	UD
**DJ694/UAS-grim;DJ1077/DJ1080,DJ1083 compared to:**							
DJ694/ UAS-grim	:DJ1083/DJ1077,DJ1083	UD	UD	UD	UD	UD	UD
'''	;DJ1079/DJ1080,DJ1083	3.45E-01	1.15E-01	3.22E-01	2.94E-01	4.73E-01	4.95E-01
'''	;DJ1080/DJ1080,DJ1083	3.07E-01	1.34E-01	4.33E-01	6.17E-01	8.71E-01	4.95E-01
'''	;DJ1079/DJ1079,DJ1080	UD	UD	UD	UD	UD	UD
'''	;DJ1080/DJ1079,DJ1080	UD	UD	UD	UD	UD	UD
'''	;DJ1079/DJ1077,DJ1083	UD	UD	UD	UD	UD	UD
**DJ694/UAS-grim;DJ1083/DJ1077,DJ1083 compared to:**							
DJ694/ UAS-grim	;DJ1079/DJ1080,DJ1083	UD	UD	UD	UD	UD	UD
'''	;DJ1080/DJ1080,DJ1083	UD	UD	UD	UD	UD	UD
'''	;DJ1079/DJ1079,DJ1080	UD	UD	UD	UD	UD	UD
'''	;DJ1080/DJ1079,DJ1080	UD	UD	UD	UD	UD	UD
'''	;DJ1079/DJ1077,DJ1083	UD	UD	UD	UD	UD	UD
**DJ694/UAS-grim;DJ1079/DJ1080,DJ1083 compared to:**							
DJ694/ UAS-grim	;DJ1080/DJ1080,DJ1083	5.47E-01	9.19E-01	3.11E-01	4.23E-01	1.19E-01	~ 1
'''	;DJ1079/DJ1079,DJ1080	UD	UD	UD	UD	UD	UD
'''	;DJ1080/DJ1079,DJ1080	UD	UD	UD	UD	UD	UD
'''	;DJ1079/DJ1077,DJ1083	UD	UD	UD	UD	UD	UD
**DJ694/UAS-grim;DJ1080/DJ1080,DJ1083 compared to:**							
DJ694/ UAS-grim	;DJ1079/DJ1079,DJ1080	UD	UD	UD	UD	UD	UD
'''	;DJ1080/DJ1079,DJ1080	UD	UD	UD	UD	UD	UD
'''	;DJ1079/DJ1077,DJ1083	UD	UD	UD	UD	UD	UD
**DJ694/UAS-grim;DJ1079/DJ1079,DJ1080 compared to:**							
DJ694/ UAS-grim	;DJ1080/DJ1079,DJ1080	UD	UD	UD	UD	UD	UD
'''	;DJ1079/DJ1077,DJ1083	UD	UD	UD	UD	UD	UD
**DJ694/UAS-grim;DJ1080/DJ1079,DJ1080 compared to:**							
DJ694/ UAS-grim	;DJ1079/DJ1077,DJ1083	UD	UD	UD	UD	UD	UD
**2 copies DJ146 + 1 copy DJ147**							

		hatching		pupation		eclosion	
		-tet	+tet	-tet	+tet	-tet	+tet
**w1118;UAS-grim/+ compared to:**							
DJ694/ UAS-grim	2.1/DJ1077,DJ1080	6.97E-02	1.48E-01	1.70E-01	1.19E-07	1.31E-07	8.04E-47
'''	2.1/DJ1077,DJ1083	1.42E-10	2.79E-10	2.61E-09	1.16E-10	2.64E-06	8.78E-39
'''	2.1/DJ1080,DJ1083	5.80E-02	8.15E-02	1.05E-03	2.42E-11	4.80E-13	8.04E-47
'''	3.3/DJ1077,DJ1080	8.98E-04	1.99E-02	3.27E-03	8.41E-11	5.34E-04	2.24E-44
'''	3.3/DJ1077,DJ1083	9.92E-08	1.05E-15	6.48E-13	1.44E-08	5.29E-09	8.78E-39
'''	3.3/DJ1080,DJ1083	9.98E-04	2.64E-04	2.57E-05	3.77E-11	1.86E-06	2.24E-44
'''	2.1/DJ1079,DJ1080	4.81E-04	2.67E-04	3.07E-01	2.88E-09	2.00E-15	8.78E-39
'''	3.3/DJ1079,DJ1080	2.37E-03	3.70E-03	1.22E-02	3.03E-08	1.92E-05	8.78E-39
**DJ694/UAS-grim compared to:**							
DJ694/ UAS-grim	2.1/DJ1077,DJ1080	6.40E-06	7.97E-06	1.00E-40	1.00E-32	3.28E-19	~ 1
'''	2.1/DJ1077,DJ1083	9.43E-01	1.04E-02	2.11E-38	4.92E-09	2.40E-12	~ 1
'''	2.1/DJ1080,DJ1083	8.89E-06	1.33E-05	1.88E-32	8.61E-19	1.53E-08	~ 1
'''	3.3/DJ1077,DJ1080	3.46E-03	1.39E-04	8.01E-33	8.58E-25	1.01E-24	~ 1
'''	3.3/DJ1077,DJ1083	5.25E-01	8.52E-03	3.31E-25	4.68E-27	1.64E-29	~ 1
'''	3.3/DJ1080,DJ1083	1.01E-03	1.82E-03	6.40E-23	8.03E-12	1.53E-16	~ 1
'''	2.1/DJ1079,DJ1080	9.44E-03	5.03E-02	6.33E-33	5.99E-46	7.02E-33	~ 1
'''	3.3/DJ1079,DJ1080	3.20E-03	5.77E-03	3.87E-32	5.67E-35	5.07E-29	~ 1
**DJ694/UAS-grim;2.1/DJ1077,DJ1080 compared to:**							
DJ694/ UAS-grim	2.1/DJ1077,DJ1083	8.80E-03	5.36E-03	5.85E-03	2.29E-02	7.64E-01	~ 1
'''	2.1/DJ1080,DJ1083	9.42E-01	8.69E-01	2.74E-01	4.27E-02	8.92E-02	~ 1
'''	3.3/DJ1077,DJ1080	3.18E-01	5.88E-01	3.49E-01	7.67E-02	3.32E-01	~ 1
'''	3.3/DJ1077,DJ1083	4.01E-02	6.88E-05	9.28E-04	1.75E-01	6.10E-01	~ 1
'''	3.3/DJ1080,DJ1083	3.60E-01	1.77E-01	1.02E-01	2.46E-02	9.75E-01	~ 1
'''	2.1/DJ1079,DJ1080	2.98E-01	1.52E-01	9.82E-01	1.27E-01	9.86E-01	~ 1
'''	3.3/DJ1079,DJ1080	4.49E-01	2.99E-01	4.29E-01	2.61E-01	3.96E-01	~ 1
**DJ694/UAS-grim;2.1/DJ1077,DJ1083 compared to:**							
DJ694/ UAS-grim	2.1/DJ1080,DJ1083	1.13E-02	1.92E-02	9.92E-02	2.18E-03	3.49E-01	~ 1
'''	3.3/DJ1077,DJ1080	1.52E-01	3.76E-02	8.71E-02	1.75E-03	3.54E-01	~ 1
'''	3.3/DJ1077,DJ1083	7.24E-01	5.25E-01	1.34E-01	4.97E-01	5.70E-01	~ 1
'''	3.3/DJ1080,DJ1083	7.88E-02	6.67E-02	6.24E-01	7.55E-03	8.12E-01	~ 1
'''	2.1/DJ1079,DJ1080	1.31E-01	2.63E-01	6.34E-02	2.34E-03	7.87E-01	~ 1
'''	3.3/DJ1079,DJ1080	7.42E-02	1.27E-01	1.27E-01	5.34E-03	4.40E-01	~ 1
**DJ694/UAS-grim;2.1/DJ1080,DJ1083 compared to:**							
DJ694/ UAS-grim	3.3/DJ1077,DJ1080	3.50E-01	7.01E-01	9.00E-01	5.93E-01	2.19E-02	~ 1
'''	3.3/DJ1077,DJ1083	4.76E-02	7.65E-05	1.12E-02	5.19E-01	8.03E-02	~ 1
'''	3.3/DJ1080,DJ1083	4.03E-01	2.23E-01	3.86E-01	4.68E-01	1.38E-01	~ 1
'''	2.1/DJ1079,DJ1080	3.49E-01	1.79E-01	4.65E-01	4.49E-01	1.44E-01	~ 1
'''	3.3/DJ1079,DJ1080	5.19E-01	3.68E-01	8.95E-01	3.44E-01	5.16E-02	~ 1
**DJ694/UAS-grim;3.3/DJ1077,DJ1080 compared to:**							

		hatching		pupation		eclosion	
		-tet	+tet	-tet	+tet	-tet	+tet
DJ694/ UAS-grim	3.3/DJ1077,DJ1083	3.15E-01	1.82E-04	1.31E-02	7.97E-01	6.87E-01	~ 1
'''	3.3/DJ1080,DJ1083	8.42E-01	3.89E-01	3.69E-01	2.60E-01	3.85E-01	~ 1
'''	2.1/DJ1079,DJ1080	9.27E-01	2.83E-01	5.39E-01	7.00E-01	3.34E-01	~ 1
'''	3.3/DJ1079,DJ1080	7.25E-01	5.63E-01	9.78E-01	5.18E-01	9.94E-01	~ 1
**DJ694/UAS-grim;3.3/DJ1077,DJ1083 compared to:**							
DJ694/ UAS-grim	3.3/DJ1080,DJ1083	2.04E-01	5.12E-04	1.50E-01	2.97E-01	6.49E-01	~ 1
'''	2.1/DJ1079,DJ1080	3.00E-01	1.54E-02	2.32E-02	9.28E-01	4.84E-01	~ 1
'''	3.3/DJ1079,DJ1080	2.03E-01	2.76E-04	3.75E-02	7.10E-01	6.62E-01	~ 1
**DJ694/UAS-grim;3.3/DJ1080,DJ1083 compared to:**							
DJ694/ UAS-grim	2.1/DJ1079,DJ1080	9.23E-01	6.25E-01	2.68E-01	2.56E-01	9.67E-01	~ 1
'''	3.3/DJ1079,DJ1080	8.29E-01	7.42E-01	4.64E-01	2.08E-01	4.57E-01	~ 1
**DJ694/UAS-grim;2.1/DJ1079,DJ1080 compared to:**							
DJ694/ UAS-grim	3.3/DJ1079,DJ1080	7.15E-01	5.07E-01	6.21E-01	6.19E-01	2.81E-01	~ 1

4 copies DJ146

**w1118;UAS-grim/+ compared to:**							
DJ694/ UAS-grim	DJ1077,DJ1079/DJ1080,DJ1080	1.28E-04	3.24E-03	2.86E-04	1.13E-03	1.51E-16	1.48E-21
'''	DJ1077,DJ1077/DJ1080,DJ1080	1.08E-01	7.93E-01	1.63E-01	2.82E-05	5.05E-24	3.11E-35
'''	DJ1077,DJ1077/DJ1080,DJ1083	5.00E-03	4.54E-06	7.14E-10	2.21E-16	1.04E-09	3.11E-35
'''	DJ1077,DJ1080/DJ1080,DJ1083	3.86E-01	6.50E-01	5.61E-03	1.68E-11	4.55E-14	8.78E-39
'''	DJ1079,DJ1079/DJ1080,DJ1080	UD	UD	UD	UD	UD	UD
'''	DJ1077,DJ1079/DJ1080,DJ1083	2.48E-08	2.75E-06	1.53E-16	5.44E-17	1.00E-20	3.11E-35
'''	DJ1079,DJ1080/DJ1080,DJ1083	1.87E-02	3.63E-01	2.53E-04	6.30E-06	1.53E-12	3.11E-35
'''	DJ1077,DJ1080/DJ1083,DJ1083	1.94E-03	3.38E-03	9.48E-07	7.04E-11	2.15E-13	1.63E-13
'''	DJ1080,DJ1080/DJ1083,DJ1083	2.68E-03	1.81E-01	8.66E-06	2.79E-14	1.36E-23	3.11E-35
'''	DJ1077,DJ1077/DJ1083,DJ1083	4.99E-06	1.56E-05	8.78E-15	9.11E-13	4.18E-19	1.76E-06
**DJ694/UAS-grim compared to:**							
DJ694/ UAS-grim	DJ1077,DJ1079/DJ1080,DJ1080	9.07E-03	7.93E-03	1.09E-39	5.43E-30	1.54E-27	3.03E-04
'''	DJ1077,DJ1077/DJ1080,DJ1080	4.91E-03	1.61E-03	4.02E-51	5.98E-85	2.74E-55	~ 1
'''	DJ1077,DJ1077/DJ1080,DJ1083	4.86E-02	7.49E-01	3.07E-16	2.89E-13	2.00E-10	~ 1
'''	DJ1077,DJ1080/DJ1080,DJ1083	2.51E-04	2.10E-04	6.20E-36	7.79E-08	1.11E-14	~ 1
'''	DJ1079,DJ1079/DJ1080,DJ1080	UD	UD	UD	UD	UD	UD
'''	DJ1077,DJ1079/DJ1080,DJ1083	2.90E-01	5.01E-01	3.33E-62	2.34E-06	1.05E-14	~ 1
'''	DJ1079,DJ1080/DJ1080,DJ1083	1.22E-02	3.57E-03	1.25E-31	3.02E-34	8.01E-13	~ 1
'''	DJ1077,DJ1080/DJ1083,DJ1083	6.40E-03	3.99E-02	1.72E-15	2.21E-07	2.47E-14	1.54E-06
'''	DJ1080,DJ1080/DJ1083,DJ1083	4.13E-02	5.88E-03	3.09E-47	4.61E-32	6.55E-24	~ 1
'''	DJ1077,DJ1077/DJ1083,DJ1083	5.18E-01	5.59E-01	7.19E-52	2.74E-30	9.61E-49	9.02E-15
**DJ694/UAS-grim;DJ1077,DJ1079/DJ1080,DJ1080 compared to:**							
DJ694/ UAS-grim	DJ1077,DJ1077/DJ1080,DJ1080	2.92E-01	1.69E-01	3.00E-01	4.83E-01	7.82E-01	4.95E-01
'''	DJ1077,DJ1077/DJ1080,DJ1083	9.31E-01	2.94E-01	1.20E-01	7.08E-03	6.45E-01	4.95E-01
'''	DJ1077,DJ1080/DJ1080,DJ1083	8.71E-02	1.00E-01	7.00E-01	5.82E-02	4.42E-01	3.74E-01

		hatching		pupation		eclosion	
		-tet	+tet	-tet	+tet	-tet	+tet
'''	DJ1079,DJ1079/DJ1080,DJ1080	UD	UD	UD	UD	UD	UD
'''	DJ1077,DJ1079/DJ1080,DJ1083	1.26E-01	2.03E-01	3.94E-03	5.68E-03	1.41E-01	4.95E-01
'''	DJ1079,DJ1080/DJ1080,DJ1083	6.46E-01	2.94E-01	7.08E-01	3.75E-01	5.19E-01	4.95E-01
'''	DJ1077,DJ1080/DJ1083,DJ1083	9.63E-01	7.51E-01	3.28E-01	6.82E-02	4.67E-01	4.71E-01
'''	DJ1080,DJ1080/DJ1083,DJ1083	9.42E-01	3.87E-01	3.65E-01	1.40E-02	1.59E-01	4.95E-01
'''	DJ1077,DJ1077/DJ1083,DJ1083	4.13E-01	3.28E-01	7.15E-03	2.45E-02	4.77E-01	1.33E-01
**DJ694/UAS-grim;DJ1077,DJ1077/DJ1080,DJ1080 compared to:**							
DJ694/ UAS-grim	DJ1077,DJ1077/DJ1080,DJ1083	6.72E-01	2.98E-01	1.60E-01	2.24E-03	7.83E-01	~ 1
'''	DJ1077,DJ1080/DJ1080,DJ1083	2.88E-01	8.54E-01	5.95E-01	1.57E-01	6.00E-01	~ 1
'''	DJ1079,DJ1079/DJ1080,DJ1080	UD	UD	UD	UD	UD	UD
'''	DJ1077,DJ1079/DJ1080,DJ1083	1.06E-02	1.28E-01	3.37E-03	5.17E-04	2.03E-01	~ 1
'''	DJ1079,DJ1080/DJ1080,DJ1083	6.92E-01	4.65E-01	3.69E-01	6.41E-01	6.69E-01	~ 1
'''	DJ1077,DJ1080/DJ1083,DJ1083	6.22E-01	4.67E-01	2.78E-01	1.86E-01	6.35E-01	3.08E-01
'''	DJ1080,DJ1080/DJ1083,DJ1083	6.12E-01	1.43E-01	8.73E-02	3.23E-03	1.54E-01	~ 1
'''	DJ1077,DJ1077/DJ1083,DJ1083	3.87E-01	2.57E-01	5.30E-03	1.28E-02	8.33E-02	1.48E-01
**DJ694/UAS-grim;DJ1077,DJ1077/DJ1080,DJ1083 compared to:**							
DJ694/ UAS-grim	DJ1077,DJ1080/DJ1080,DJ1083	4.40E-01	1.63E-01	1.12E-01	3.82E-01	9.61E-01	~ 1
'''	DJ1079,DJ1079/DJ1080,DJ1080	UD	UD	UD	UD	UD	UD
'''	DJ1077,DJ1079/DJ1080,DJ1083	6.09E-01	7.53E-01	4.16E-01	3.09E-01	6.76E-01	~ 1
'''	DJ1079,DJ1080/DJ1080,DJ1083	8.07E-01	3.29E-01	3.12E-01	1.61E-02	9.61E-01	~ 1
'''	DJ1077,DJ1080/DJ1083,DJ1083	9.70E-01	4.72E-01	6.37E-01	3.57E-01	9.90E-01	3.08E-01
'''	DJ1080,DJ1080/DJ1083,DJ1083	9.87E-01	3.49E-01	3.12E-01	6.03E-02	7.82E-01	~ 1
'''	DJ1077,DJ1077/DJ1083,DJ1083	6.51E-01	7.92E-01	6.55E-01	4.21E-02	5.57E-01	1.48E-01
**DJ694/UAS-grim;DJ1077,DJ1080/DJ1080,DJ1083 compared to:**							
DJ694/ UAS-grim	DJ1079,DJ1079/DJ1080,DJ1080	UD	UD	UD	UD	UD	UD
'''	DJ1077,DJ1079/DJ1080,DJ1083	2.03E-03	4.80E-02	7.33E-03	3.05E-01	5.47E-01	~ 1
'''	DJ1079,DJ1080/DJ1080,DJ1083	3.41E-01	4.87E-01	5.59E-01	1.97E-01	9.85E-01	~ 1
'''	DJ1077,DJ1080/DJ1083,DJ1083	3.91E-01	3.69E-01	2.68E-01	9.14E-01	9.53E-01	1.99E-01
'''	DJ1080,DJ1080/DJ1083,DJ1083	3.57E-01	1.70E-01	3.11E-01	7.78E-01	7.03E-01	~ 1
'''	DJ1077,DJ1077/DJ1083,DJ1083	1.95E-01	1.33E-01	1.25E-02	9.14E-01	3.17E-01	5.40E-02
**DJ694/UAS-grim;DJ1079,DJ1079/DJ1080,DJ1080 compared to:**							
DJ694/ UAS-grim	DJ1077,DJ1079/DJ1080,DJ1083	UD	UD	UD	UD	UD	UD
'''	DJ1079,DJ1080/DJ1080,DJ1083	UD	UD	UD	UD	UD	UD
'''	DJ1077,DJ1080/DJ1083,DJ1083	UD	UD	UD	UD	UD	UD
'''	DJ1080,DJ1080/DJ1083,DJ1083	UD	UD	UD	UD	UD	UD
'''	DJ1077,DJ1077/DJ1083,DJ1083	UD	UD	UD	UD	UD	UD
**DJ694/UAS-grim;DJ1077,DJ1079/DJ1080,DJ1083 compared to:**							
DJ694/ UAS-grim	DJ1079,DJ1080/DJ1080,DJ1083	1.61E-01	1.60E-01	4.62E-02	1.24E-02	6.57E-01	~ 1
'''	DJ1077,DJ1080/DJ1083,DJ1083	4.88E-01	6.52E-01	2.32E-01	2.85E-01	4.94E-01	3.08E-01
'''	DJ1080,DJ1080/DJ1083,DJ1083	5.09E-01	1.74E-01	6.79E-03	2.28E-02	7.38E-01	~ 1

		hatching		pupation		eclosion	
		-tet	+tet	-tet	+tet	-tet	+tet
'''	DJ1077,DJ1077/DJ1083,DJ1083	8.73E-01	9.75E-01	1.93E-02	2.37E-02	1.16E-01	1.48E-01
**DJ694/UAS-grim;DJ1079,DJ1080/DJ1080,DJ1083 compared to:**							
DJ694/ UAS-grim	DJ1077,DJ1080/DJ1083,DJ1083	7.83E-01	5.45E-01	5.41E-01	2.31E-01	9.45E-01	3.08E-01
'''	DJ1080,DJ1080/DJ1083,DJ1083	7.84E-01	6.11E-01	8.01E-01	2.82E-02	7.83E-01	~ 1
'''	DJ1077,DJ1077/DJ1083,DJ1083	4.68E-01	2.98E-01	6.95E-02	5.29E-02	4.27E-01	1.48E-01
**DJ694/UAS-grim;DJ1077,DJ1080/DJ1083,DJ1083 compared to:**							
DJ694/ UAS-grim	DJ1080,DJ1080/DJ1083,DJ1083	9.86E-01	6.00E-01	6.12E-01	7.16E-01	6.56E-01	3.08E-01
'''	DJ1077,DJ1077/DJ1083,DJ1083	4.83E-01	6.54E-01	3.75E-01	9.97E-01	3.26E-01	2.92E-01
**DJ694/UAS-grim;DJ1080,DJ1080/DJ1083,DJ1083 compared to:**							
DJ694/ UAS-grim	DJ1077,DJ1077/DJ1083,DJ1083	6.14E-01	3.24E-01	1.20E-02	2.11E-01	7.54E-02	1.48E-01
**2 copies DJ146 + 2 copies DJ147**							
**w1118;UAS-grim/+ compared to:**							
DJ694/ UAS-grim	2.1,DJ1077/2.1,DJ1077	8.57E-03	3.91E-03	7.19E-02	2.85E-01	3.27E-03	3.11E-35
'''	2.1,DJ1077/2.1,DJ1079	1.50E-11	6.99E-10	2.71E-07	2.36E-07	1.14E-05	9.75E-42
'''	2.1,DJ1077/3.3,DJ1077	1.10E-06	4.78E-10	3.24E-01	7.39E-01	2.34E-01	1.94E-24
'''	2.1,DJ1079/2.1,DJ1079	3.66E-08	2.39E-10	6.75E-09	2.75E-04	1.17E-03	8.82E-32
'''	2.1,DJ1077/3.3,DJ1079	4.89E-09	6.97E-12	4.43E-06	1.14E-03	6.57E-02	3.62E-24
'''	3.3,DJ1077/3.3,DJ1077	2.67E-06	1.34E-08	1.04E-03	1.13E-02	3.32E-01	4.97E-16
'''	3.3,DJ1077/3.3,DJ1079	1.15E-09	3.85E-08	5.87E-01	9.66E-02	4.64E-01	7.23E-25
'''	2.1,DJ1079/3.3,DJ1079	4.05E-10	4.76E-08	1.47E-04	3.19E-04	1.06E-06	1.93E-26
**DJ694/UAS-grim compared to:**							
DJ694/ UAS-grim	2.1,DJ1077/2.1,DJ1077	1.29E-02	3.81E-02	5.40E-34	2.27E-38	#######	~ 1
'''	2.1,DJ1077/2.1,DJ1079	3.43E-03	2.46E-01	3.30E-24	1.07E-24	1.77E-39	3.30E-03
'''	2.1,DJ1077/3.3,DJ1077	3.82E-02	8.54E-01	2.44E-35	6.49E-43	2.25E-68	2.62E-05
'''	2.1,DJ1079/2.1,DJ1079	3.58E-04	2.30E-02	5.53E-24	7.23E-35	1.08E-38	4.48E-06
'''	2.1,DJ1077/3.3,DJ1079	9.20E-04	6.76E-01	1.11E-27	2.86E-33	6.75E-52	4.80E-08
'''	3.3,DJ1077/3.3,DJ1077	2.39E-01	9.55E-01	4.73E-29	4.04E-68	4.62E-76	7.19E-15
'''	3.3,DJ1077/3.3,DJ1079	5.06E-01	8.02E-01	8.91E-37	4.49E-42	4.00E-54	4.88E-16
'''	2.1,DJ1079/3.3,DJ1079	1.01E-01	3.04E-01	2.14E-22	9.21E-27	8.14E-46	4.57E-15
**DJ694/UAS-grim;2.1,DJ1077/2.1,DJ1077 compared to:**							
DJ694/ UAS-grim	2.1,DJ1077/2.1,DJ1079	7.46E-02	4.49E-03	2.98E-01	7.32E-01	4.82E-01	5.07E-01
'''	2.1,DJ1077/3.3,DJ1077	3.85E-01	1.24E-01	6.94E-01	5.54E-01	1.76E-02	3.82E-01
'''	2.1,DJ1079/2.1,DJ1079	3.57E-01	1.53E-01	1.91E-01	4.23E-01	7.18E-01	2.72E-01
'''	2.1,DJ1077/3.3,DJ1079	2.87E-01	1.03E-01	4.31E-01	5.62E-01	6.99E-01	1.63E-01
'''	3.3,DJ1077/3.3,DJ1077	3.74E-01	1.33E-02	6.50E-01	3.78E-01	2.05E-02	1.46E-01
'''	3.3,DJ1077/3.3,DJ1079	9.32E-03	8.44E-03	5.27E-01	7.55E-01	4.29E-01	1.26E-01
'''	2.1,DJ1079/3.3,DJ1079	7.17E-02	2.40E-01	5.51E-01	3.89E-01	4.31E-01	5.85E-02
**DJ694/UAS-grim;2.1,DJ1077/2.1,DJ1079 compared to:**							
DJ694/ UAS-grim	2.1,DJ1077/3.3,DJ1077	1.00E+00	2.45E-01	7.60E-02	3.88E-01	6.64E-02	1.91E-01
'''	2.1,DJ1079/2.1,DJ1079	2.90E-01	8.89E-02	6.93E-01	8.40E-01	6.42E-01	1.72E-01

		hatching		pupation		eclosion	
		-tet	+tet	-tet	+tet	-tet	+tet
'''	2.1,DJ1077/3.3,DJ1079	7.88E-01	4.99E-01	5.38E-01	9.64E-01	7.56E-02	2.36E-02
'''	3.3,DJ1077/3.3,DJ1077	6.21E-01	2.19E-01	6.14E-01	8.92E-01	1.85E-01	1.88E-03
'''	3.3,DJ1077/3.3,DJ1079	1.12E-01	4.15E-01	1.04E-01	8.76E-01	2.92E-01	7.64E-03
'''	2.1,DJ1079/3.3,DJ1079	4.02E-01	7.52E-01	7.18E-01	5.84E-01	8.13E-01	2.65E-03
**DJ694/UAS-grim;2.1,DJ1077/3.3,DJ1077 compared to:**							
DJ694/ UAS-grim	2.1,DJ1079/2.1,DJ1079	5.26E-01	6.97E-02	3.58E-02	8.84E-02	1.72E-01	7.58E-01
'''	2.1,DJ1077/3.3,DJ1079	8.65E-01	6.99E-01	1.32E-01	1.70E-01	2.94E-01	4.68E-01
'''	3.3,DJ1077/3.3,DJ1077	7.96E-01	8.77E-01	3.34E-01	8.25E-02	9.38E-01	1.50E-01
'''	3.3,DJ1077/3.3,DJ1079	4.42E-01	7.57E-01	6.24E-01	2.92E-01	3.98E-01	4.30E-01
'''	2.1,DJ1079/3.3,DJ1079	6.54E-01	4.15E-01	2.50E-01	1.06E-01	2.91E-02	3.10E-01
**DJ694/UAS-grim;2.1,DJ1079/2.1,DJ1079 compared to:**							
DJ694/ UAS-grim	2.1,DJ1077/3.3,DJ1079	5.36E-01	1.04E-01	2.92E-01	8.18E-01	2.53E-01	1.75E-01
'''	3.3,DJ1077/3.3,DJ1077	3.53E-01	8.81E-02	4.42E-01	9.58E-01	3.29E-01	1.83E-02
'''	3.3,DJ1077/3.3,DJ1079	6.76E-02	1.43E-01	6.05E-02	6.30E-01	4.78E-01	1.45E-01
'''	2.1,DJ1079/3.3,DJ1079	1.43E-01	4.75E-01	5.03E-01	5.34E-01	8.53E-01	7.45E-02
**DJ694/UAS-grim;2.1,DJ1077/3.3,DJ1079 compared to:**							
DJ694/ UAS-grim	3.3,DJ1077/3.3,DJ1077	6.10E-01	8.88E-01	8.61E-01	8.55E-01	4.42E-01	1.72E-01
'''	3.3,DJ1077/3.3,DJ1079	2.34E-01	9.72E-01	1.37E-01	7.74E-01	7.88E-01	7.69E-01
'''	2.1,DJ1079/3.3,DJ1079	4.26E-01	5.30E-01	9.42E-01	4.51E-01	1.28E-01	7.37E-01
**DJ694/UAS-grim;3.3,DJ1077/3.3,DJ1077 compared to:**							
DJ694/ UAS-grim	3.3,DJ1077/3.3,DJ1079	7.00E-01	4.65E-01	3.50E-01	4.62E-01	5.44E-01	4.80E-01
'''	2.1,DJ1079/3.3,DJ1079	9.83E-01	5.23E-01	8.69E-01	7.23E-01	1.17E-01	2.68E-01
**DJ694/UAS-grim;3.3,DJ1077/3.3,DJ1079 compared to:**							
DJ694/ UAS-grim	2.1,DJ1079/3.3,DJ1079	4.20E-01	6.37E-01	2.83E-01	4.98E-01	2.32E-01	9.49E-01

Supplemental Table 3: UAS-grim lethality assay statistical analysis (t-tests).

		hatching		pupation		eclosion	
		-tet	+tet	-tet	+tet	-tet	+tet
		**w1118;UAS-grim/+ compared to:**					
DJ694/UAS-grim		4.08E-20	2.54E-18	1.20E-71	8.17E-62	1.58E-103	2.66E-101
""	+ 1 copy 146	5.83E-09	1.85E-07	8.00E-10	5.11E-39	3.19E-61	1.56E-36
""	+ 2 copy 146	2.73E-03	1.13E-03	1.39E-09	2.31E-17	2.46E-29	3.39E-37
""	+ 3 copy 146	4.44E-07	2.55E-05	7.40E-06	2.91E-08	4.75E-13	5.47E-36
""	+ 4 copy 146	2.69E-05	7.40E-04	5.32E-07	1.47E-10	1.96E-16	1.88E-28
""	+ 1 copy 147	2.00E-08	2.70E-10	9.14E-26	1.28E-30	1.42E-58	1.17E-65
""	+ 2 copy 147	1.22E-06	1.41E-07	2.81E-10	8.17E-13	1.38E-23	3.80E-84
""	+ 2 copy 146+147	2.75E-03	2.37E-04	9.65E-14	1.43E-24	5.12E-11	4.22E-104
""	+ 3 copy 146+147	6.30E-05	1.53E-04	3.20E-05	8.94E-16	1.28E-07	1.26E-97
""	+ 4 copy 146+147	6.09E-15	7.78E-19	1.05E-06	2.14E-04	2.31E-03	2.12E-39
		**DJ694/UAS-grim compared to:**					
""	+ 1 copy 146	7.60E-04	4.25E-04	7.89E-14	3.46E-04	2.46E-02	7.05E-02
""	+ 2 copy 146	2.46E-10	1.99E-07	1.53E-32	4.20E-07	1.91E-03	3.21E-01
""	+ 3 copy 146	2.53E-04	3.39E-03	9.06E-23	1.94E-12	4.20E-22	1.12E-02
""	+ 4 copy 146	2.68E-08	8.42E-06	1.06E-23	2.18E-09	4.89E-18	9.01E-03
""	+ 1 copy 147	2.28E-01	7.67E-01	4.36E-06	8.94E-04	1.22E-01	~ 1
""	+ 2 copy 147	8.17E-08	6.76E-07	1.50E-23	2.14E-13	8.53E-07	2.56E-01
""	+ 2 copy 146+147	1.09E-14	6.28E-10	1.33E-29	3.88E-13	2.72E-11	1.98E-01
""	+ 3 copy 146+147	6.27E-08	1.92E-05	1.30E-36	2.37E-27	2.02E-20	~ 1
""	+ 4 copy 146+147	5.80E-08	4.54E-02	1.99E-35	4.74E-46	1.37E-66	1.65E-06
		**1 copy 146 compared to:**					
""	+ 2 copy 146	1.99E-02	1.96E-01	1.13E-02	7.78E-04	5.43E-02	8.93E-01
""	+ 3 copy 146	8.01E-01	8.59E-01	4.28E-02	8.55E-07	5.23E-12	5.89E-01
""	+ 4 copy 146	5.40E-02	3.23E-01	5.55E-02	4.25E-05	1.14E-09	3.35E-01
""	+ 1 copy 147	2.01E-01	2.62E-02	3.74E-04	9.69E-02	8.26E-01	2.35E-01
""	+ 2 copy 147	1.53E-01	4.49E-01	2.01E-01	2.57E-07	1.27E-03	1.33E-01
""	+ 2 copy 146+147	4.75E-04	6.83E-02	1.54E-01	5.40E-07	4.70E-06	7.07E-02
""	+ 3 copy 146+147	1.46E-01	5.54E-01	1.76E-04	5.93E-16	2.74E-11	7.96E-02
""	+ 4 copy 146+147	6.88E-01	7.59E-03	7.88E-04	3.69E-29	1.23E-42	2.05E-02
		**2 copies of 146 compared to:**					
""	+ 3 copy 146	3.67E-02	1.58E-01	8.44E-01	7.98E-03	1.90E-09	5.17E-01
""	+ 4 copy 146	5.71E-01	8.18E-01	8.99E-01	1.24E-01	7.35E-07	2.38E-01
""	+ 1 copy 147	8.25E-04	6.35E-04	1.73E-12	8.20E-03	5.80E-02	5.12E-01
""	+ 2 copy 147	2.13E-01	3.90E-01	1.57E-01	3.03E-02	1.15E-01	4.13E-01
""	+ 2 copy 146+147	4.42E-01	7.69E-01	6.50E-02	7.05E-01	8.27E-06	3.25E-01
""	+ 3 copy 146+147	3.05E-01	4.62E-01	3.37E-02	1.68E-05	1.00E-11	3.36E-01
""	+ 4 copy 146+147	1.55E-03	7.35E-06	2.02E-01	2.11E-16	5.04E-32	5.71E-03
		**3 copy 146 compared to:**					
""	+ 4 copy 146	1.17E-01	2.93E-01	8.04E-01	3.25E-01	1.33E-01	5.58E-01

""	+ 1 copy 147	1.38E-01	7.17E-02	2.95E-08	1.53E-05	1.60E-11	9.34E-02
""	+ 2 copy 147	2.72E-01	4.01E-01	2.20E-01	4.11E-01	4.13E-06	3.14E-02
""	+ 2 copy 146+147	1.84E-03	6.34E-02	1.13E-01	3.15E-03	1.91E-01	9.94E-03
""	+ 3 copy 146+147	2.30E-01	4.52E-01	1.58E-01	5.81E-01	3.55E-01	1.39E-02
""	+ 4 copy 146+147	9.95E-01	4.15E-02	4.45E-01	3.24E-06	4.64E-12	5.44E-02
			**4 copy 146 compared to:**				
""	+ 1 copy 147	5.06E-03	3.83E-03	1.68E-08	5.03E-04	2.79E-09	8.35E-02
""	+ 2 copy 147	5.13E-01	5.95E-01	3.14E-01	7.38E-01	5.36E-04	2.50E-02
""	+ 2 copy 146+147	1.40E-01	5.96E-01	1.84E-01	1.16E-01	9.17E-01	7.34E-03
""	+ 3 copy 146+147	6.80E-01	6.75E-01	7.24E-02	4.80E-02	2.22E-02	1.13E-02
""	+ 4 copy 146+147	1.21E-02	1.38E-04	2.58E-01	5.59E-09	2.84E-16	2.92E-01
			**1 copy 147 compared to:**				
""	+ 2 copy 147	9.79E-03	1.14E-03	3.69E-08	9.00E-06	1.63E-03	4.55E-01
""	+ 2 copy 146+147	1.48E-05	3.23E-05	1.47E-10	6.22E-05	8.04E-06	3.94E-01
""	+ 3 copy 146+147	8.21E-03	6.24E-03	4.94E-15	6.34E-13	7.67E-11	~ 1
""	+ 4 copy 146+147	2.24E-02	3.00E-01	1.74E-14	4.09E-25	2.49E-41	1.10E-03
			**2 copy 147 compared to:**				
""	+ 2 copy 146+147	2.14E-02	1.74E-01	8.90E-01	1.98E-02	3.08E-03	9.42E-01
""	+ 3 copy 146+147	8.48E-01	9.75E-01	1.73E-03	6.14E-02	1.12E-07	2.71E-01
""	+ 4 copy 146+147	8.18E-02	4.49E-05	1.25E-02	9.49E-11	1.94E-25	4.09E-05
			**2 copy 146+147 compared to:**				
""	+ 3 copy 146+147	5.34E-02	2.67E-01	1.17E-04	3.77E-07	2.01E-02	2.11E-01
""	+ 4 copy 146+147	1.91E-06	3.59E-08	1.81E-03	5.96E-22	1.52E-11	7.90E-07
			**3 copy 146+147 compared to:**				
""	+ 4 copy 146+147	6.65E-02	6.10E-04	3.64E-01	2.99E-10	4.59E-07	3.19E-06

Supplemental Table 4: UAS-lacZ assay L1 data set.

% DJ694 control: specific activity normalized to the positive control, bolded values are significantly different ($p<0.05$) from the positive control.

L1 larvae

Parents		Date	Specific activity ΔmOD/min/µg protein	% DJ694 control
Male	Female			
			**DJ694 + DJ1077**	
DJ694;1077	Bg2	15-Jun-07	1.95	**55.27**
DJ694;1077	Bg2	15-Jun-07	1.81	**51.36**
DJ694;1077	Bg2	15-Jun-07	2.07	**58.45**
DJ694;1077	Bg2	15-Jun-07	2.40	**67.95**
DJ694;1077	Bg2	04-Jul-07	0.22	**47.33**
DJ694;1077	Bg2	04-Jul-07	0.31	**68.97**
DJ694;1077	Bg2	04-Jul-07	0.27	**59.03**
DJ694;1077	Bg2	31-May-07	0.64	54.91
DJ694;1077	Bg2	31-May-07	0.96	82.43
DJ694;1077	Bg2	31-May-07	0.66	56.22
DJ694;1077	Bg2	31-May-07	0.93	79.78
			**DJ694 + DJ1080**	
DJ694;1080	Bg2	15-Jun-07	2.37	67.05
DJ694;1080	Bg2	15-Jun-07	3.40	96.35
DJ694;1080	Bg2	15-Jun-07	2.93	82.87
DJ694;1080	Bg2	15-Jun-07	4.49	127.17
DJ694;1080	Bg2	31-May-07	0.85	73.03
DJ694;1080	Bg2	31-May-07	0.97	82.87
DJ694;1080	Bg2	31-May-07	1.40	119.79
DJ694;1080	Bg2	31-May-07	1.49	127.36
DJ694;1080	Bg2	04-Jul-07	0.24	**53.14**
DJ694;1080	Bg2	04-Jul-07	0.31	**68.25**
DJ694;1080	Bg2	04-Jul-07	0.37	**82.01**
			**DJ694 + DJ1077 + DJ1080**	
DJ694	Bg2;1077,1080	03-Aug-07	2.43	173.06
DJ694	Bg2;1077,1080	03-Aug-07	0.36	25.82
DJ694	Bg2;1077,1080	03-Aug-07	0.70	50.09
DJ694	Bg2;1077,1080	15-Aug-07	0.07	14.90
DJ694	Bg2;1077,1080	15-Aug-07	0.09	18.72
DJ694;1077,1080	Bg2	15-Jun-07	1.53	**43.42**
DJ694;1077,1080	Bg2	15-Jun-07	2.58	**72.99**
DJ694;1077,1080	Bg2	15-Jun-07	2.28	**64.45**
DJ694;1077,1080	Bg2	15-Jun-07	1.51	**42.60**
DJ694;1077,1080	Bg2	04-Jul-07	0.05	**10.06**
DJ694;1077,1080	Bg2	04-Jul-07	0.06	**13.16**
DJ694;1077,1080	Bg2	04-Jul-07	0.17	**37.57**
			**DJ694 + DJ1080 + DJ1083**	
DJ694	Bg2;1080,1083	15-Aug-07	0.32	66.47

L1 larvae

Parents		Date	Specific activity ΔmOD/min/μg protein	% DJ694 control
**Male**	**Female**			
DJ694	Bg2;1080,1083	15-Aug-07	0.31	64.11
DJ694	Bg2;1080,1083	15-Aug-07	0.47	98.59
DJ694	Bg2;1080,1083	15-Jun-07	2.25	63.74
DJ694	Bg2;1080,1083	15-Jun-07	2.39	67.66
DJ694	Bg2;1080,1083	15-Jun-07	1.88	53.26
DJ694	Bg2;1080,1083	15-Jun-07	3.48	98.60
DJ694	Bg2;1080,1083	04-Jul-07	0.43	95.60
DJ694	Bg2;1080,1083	04-Jul-07	0.62	136.45
DJ694	Bg2;1080,1083	04-Jul-07	0.77	169.49
DJ694	Bg2;1080,1083	12-Jul-07	0.37	**44.00**
DJ694	Bg2;1080,1083	12-Jul-07	0.38	**44.40**
DJ694	Bg2;1080,1083	12-Jul-07	0.21	**24.43**
			**DJ694 + DJ1077 + DJ1079**	
DJ694;1077,1079	Bg2	15-Aug-07	0.18	38.30
DJ694;1077,1079	Bg2	15-Aug-07	0.15	32.17
DJ694;1077,1079	Bg2	15-Aug-07	0.24	49.62
DJ694;1077,1079	Bg2	15-Jun-07	1.10	**31.13**
DJ694;1077,1079	Bg2	15-Jun-07	1.17	**33.21**
DJ694;1077,1079	Bg2	15-Jun-07	1.43	**40.51**
DJ694;1077,1079	Bg2	15-Jun-07	1.26	**35.74**
DJ694;1077,1079	Bg2	04-Jul-07	0.38	**83.63**
DJ694;1077,1079	Bg2	04-Jul-07	0.24	**53.26**
DJ694;1077,1079	Bg2	04-Jul-07	0.24	**51.86**
			**DJ694 + 2 copies DJ1077 + DJ1080**	
DJ694;1077	Bg2;1077,1080	03-Aug-07	0.04	**3.07**
DJ694;1077	Bg2;1077,1080	03-Aug-07	0.07	**4.72**
DJ694;1077	Bg2;1077,1080	03-Aug-07	0.07	**5.23**
DJ694;1077	Bg2;1077,1080	15-Aug-07	0.01	2.28
DJ694;1077	Bg2;1077,1080	15-Aug-07	0.03	6.78
DJ694;1077	Bg2;1077,1080	12-Jul-07	0.00	**0.00**
DJ694;1077	Bg2;1077,1080	12-Jul-07	0.04	**5.12**
DJ694;1077	Bg2;1077,1080	12-Jul-07	0.04	**4.62**
DJ694;1077	Bg2;1080,1083	15-Aug-07	0.03	**5.21**
DJ694;1077	Bg2;1080,1083	15-Aug-07	0.01	**2.40**
DJ694;1077	Bg2;1080,1083	15-Aug-07	0.01	**2.94**
			**DJ694 + DJ1077 + 2 copies DJ1080**	
DJ694;1080	Bg2;1077,1080	03-Aug-07	0.10	**6.90**
DJ694;1080	Bg2;1077,1080	03-Aug-07	0.11	**8.08**
DJ694;1080	Bg2;1077,1080	03-Aug-07	0.07	**4.77**

L1 larvae

Parents		Date	Specific activity	% DJ694 control
Male	Female		ΔmOD/min/μg protein	
DJ694;1080	Bg2;1077,1080	15-Aug-07	0.03	**5.91**
DJ694;1080	Bg2;1077,1080	15-Aug-07	0.02	**4.12**
DJ694;1080	Bg2;1077,1080	15-Aug-07	0.04	**8.94**
DJ694;1080	Bg2;1077,1080	12-Jul-07	0.07	**7.74**
DJ694;1080	Bg2;1077,1080	12-Jul-07	0.06	**7.48**
DJ694;1080	Bg2;1077,1080	12-Jul-07	0.06	**6.79**
			**DJ694 + 2 copies DJ1080 + DJ1083**	
DJ694;1080	Bg2;1080,1083	03-Aug-07	0.18	**12.88**
DJ694;1080	Bg2;1080,1083	03-Aug-07	0.15	**11.03**
DJ694;1080	Bg2;1080,1083	03-Aug-07	0.08	**5.80**
DJ694;1080	Bg2;1080,1083	15-Aug-07	0.05	**10.52**
DJ694;1080	Bg2;1080,1083	15-Aug-07	0.04	**9.12**
DJ694;1080	Bg2;1080,1083	15-Aug-07	0.05	**10.58**
			**DJ694 + 2 copies DJ1077 + DJ1079 + DJ1080**	
DJ694;1077,1079	Bg2;1077,1080	15-Jun-07	0.17	**4.70**
DJ694;1077,1079	Bg2;1077,1080	15-Jun-07	0.07	**2.03**
DJ694;1077,1079	Bg2;1077,1080	15-Jun-07	0.22	**6.37**
DJ694;1077,1079	Bg2;1077,1080	15-Jun-07	0.20	**5.77**
DJ694;1077,1079	Bg2;1077,1080	04-Jul-07	0.10	**21.26**
DJ694;1077,1079	Bg2;1077,1080	04-Jul-07	0.00	**0.00**
DJ694;1077,1079	Bg2;1077,1080	04-Jul-07	0.05	**11.22**
			**DJ694 + DJ1077 + DJ1079 + DJ1080 + DJ1083**	
DJ694;1077,1079	Bg2;1080,1083	04-Jul-07	0.04	**8.51**
DJ694;1077,1079	Bg2;1080,1083	04-Jul-07	0.03	**6.97**
DJ694;1077,1079	Bg2;1080,1083	04-Jul-07	0.06	**12.38**
DJ694;1077,1079	Bg2;1080,1083	12-Jul-07	0.01	**1.02**
DJ694;1077,1079	Bg2;1080,1083	12-Jul-07	0.02	**2.71**
DJ694;1077,1079	Bg2;1080,1083	12-Jul-07	0.00	**0.00**
			**DJ694 + 2 copies DJ1077 + 2 copies DJ1080**	
DJ694;1077,1080	Bg2;1077,1080	15-Jun-07	0.19	**5.38**
DJ694;1077,1080	Bg2;1077,1080	15-Jun-07	0.20	**5.65**
DJ694;1077,1080	Bg2;1077,1080	15-Jun-07	0.10	**2.71**
DJ694;1077,1080	Bg2;1077,1080	15-Jun-07	0.14	**3.99**
DJ694;1077,1080	Bg2;1077,1080	31-May-07	0.02	**1.89**
DJ694;1077,1080	Bg2;1077,1080	31-May-07	0.00	**0.00**
DJ694;1077,1080	Bg2;1077,1080	31-May-07	0.00	**0.00**
DJ694;1077,1080	Bg2;1077,1080	04-Jul-07	0.05	**10.48**
DJ694;1077,1080	Bg2;1077,1080	04-Jul-07	0.07	**15.41**
DJ694;1077,1080	Bg2;1077,1080	04-Jul-07	0.04	**8.97**

L1 larvae

Parents		Date	Specific activity ΔmOD/min/μg protein	% DJ694 control
Male	Female			
			**DJ694 + 2.1**	
DJ694;2.1	Bg2	15-Jun-07	2.75	77.72
DJ694;2.1	Bg2	15-Jun-07	3.95	111.67
DJ694;2.1	Bg2	15-Jun-07	3.78	107.03
DJ694;2.1	Bg2	15-Jun-07	3.66	103.54
DJ694;2.1	Bg2	31-May-07	1.28	109.90
DJ694;2.1	Bg2	31-May-07	1.16	99.67
DJ694;2.1	Bg2	31-May-07	1.28	109.64
DJ694;2.1	Bg2	31-May-07	1.37	117.60
DJ694;2.1	Bg2	04-Jul-07	0.20	43.04
DJ694;2.1	Bg2	04-Jul-07	0.24	52.28
DJ694;2.1	Bg2	04-Jul-07	0.43	93.74
			**DJ694 + 3.3**	
DJ694;3.3	Bg2	15-Jun-07	2.38	67.24
DJ694;3.3	Bg2	15-Jun-07	4.59	129.94
DJ694;3.3	Bg2	15-Jun-07	2.42	68.50
DJ694;3.3	Bg2	15-Jun-07	2.69	76.07
DJ694;3.3	Bg2	31-May-07	0.87	74.91
DJ694;3.3	Bg2	31-May-07	0.78	66.96
DJ694;3.3	Bg2	31-May-07	0.13	11.18
DJ694;3.3	Bg2	31-May-07	0.66	56.57
DJ694;3.3	Bg2	04-Jul-07	0.35	76.42
DJ694;3.3	Bg2	04-Jul-07	0.41	89.64
DJ694;3.3	Bg2	04-Jul-07	0.40	88.06
			**DJ694 + 2.1 + 3.3**	
DJ694	Bg2;2.1,3.3	04-Jul-07	0.28	**60.60**
DJ694	Bg2;2.1,3.3	04-Jul-07	0.27	**58.85**
DJ694	Bg2;2.1,3.3	04-Jul-07	0.18	**40.06**
DJ694	Bg2;2.1,3.3	12-Jul-07	0.35	**41.23**
DJ694	Bg2;2.1,3.3	12-Jul-07	0.16	**18.52**
DJ694	Bg2;2.1,3.3	12-Jul-07	0.49	**57.27**
			**DJ694 + 2 copies 2.1 + 3.3**	
DJ694;2.1	Bg2;2.1,3.3	03-Aug-07	0.38	**27.02**
DJ694;2.1	Bg2;2.1,3.3	03-Aug-07	0.44	**31.59**
DJ694;2.1	Bg2;2.1,3.3	03-Aug-07	0.37	**26.21**
DJ694;2.1	Bg2;2.1,3.3	15-Aug-07	0.25	52.51
DJ694;2.1	Bg2;2.1,3.3	15-Aug-07	0.24	49.28
DJ694;2.1	Bg2;2.1,3.3	15-Aug-07	0.17	35.97
DJ694;2.1	Bg2;2.1,3.3	12-Jul-07	0.40	**47.26**

L1 larvae

Parents		Date	Specific activity	% DJ694 control
Male	Female		ΔmOD/min/µg protein	
DJ694;2.1	Bg2;2.1,3.3	12-Jul-07	0.32	**37.87**
DJ694;2.1	Bg2;2.1,3.3	12-Jul-07	0.17	**19.60**
			**DJ694 + 2.1 + 2 copies 3.3**	
DJ694;3.3	Bg2;2.1,3.3	03-Aug-07	0.60	**42.57**
DJ694;3.3	Bg2;2.1,3.3	03-Aug-07	0.55	**39.19**
DJ694;3.3	Bg2;2.1,3.3	03-Aug-07	0.48	**34.07**
DJ694;3.3	Bg2;2.1,3.3	15-Aug-07	0.30	61.81
DJ694;3.3	Bg2;2.1,3.3	15-Aug-07	0.33	67.92
DJ694;3.3	Bg2;2.1,3.3	12-Jul-07	0.33	**38.78**
DJ694;3.3	Bg2;2.1,3.3	12-Jul-07	0.16	**19.19**
DJ694;3.3	Bg2;2.1,3.3	12-Jul-07	0.23	**26.74**
			**DJ694 + 2.1 + DJ1077**	
DJ694	Bg2;2.1,1077	13-Nov-07	0.16	**16.94**
DJ694	Bg2;2.1,1077	13-Nov-07	0.24	**26.31**
DJ694	Bg2;2.1,1077	13-Nov-07	0.09	**9.73**
DJ694	Bg2;2.1,1077	30-Nov-07	0.18	**28.32**
DJ694	Bg2;2.1,1077	30-Nov-07	0.03	**4.18**
DJ694	Bg2;2.1,1077	30-Nov-07	0.10	**16.24**
DJ694;2.1,1077	Bg2	22-Feb-08	0.02	**3.49**
DJ694;2.1,1077	Bg2	22-Feb-08	0.05	**7.73**
DJ694;2.1,1077	Bg2	22-Feb-08	0.31	44.45
			**DJ694 + 2.1 + DJ1079**	
DJ694	Bg2;2.1,1079(13)	13-Nov-07	0.57	**62.15**
DJ694	Bg2;2.1,1079(13)	13-Nov-07	0.38	**41.20**
DJ694	Bg2;2.1,1079(13)	13-Nov-07	0.22	**23.29**
DJ694	Bg2;2.1,1079(13)	30-Nov-07	0.26	**42.04**
DJ694	Bg2;2.1,1079(13)	30-Nov-07	0.30	**47.68**
DJ694	Bg2;2.1,1079(13)	30-Nov-07	0.58	**93.49**
DJ694	Bg2;2.1,1079(14)	13-Nov-07	0.53	**57.14**
DJ694	Bg2;2.1,1079(14)	13-Nov-07	0.28	**30.50**
DJ694	Bg2;2.1,1079(14)	13-Nov-07	0.24	**26.14**
DJ694	Bg2;2.1,1079(14)	30-Nov-07	0.23	**37.82**
DJ694	Bg2;2.1,1079(14)	30-Nov-07	0.34	**54.26**
DJ694	Bg2;2.1,1079(14)	30-Nov-07	0.13	**20.21**
DJ694;2.1,1079(13)	Bg2	22-Feb-08	0.03	**3.70**
DJ694;2.1,1079(13)	Bg2	22-Feb-08	0.00	**0.00**
DJ694;2.1,1079(13)	Bg2	22-Feb-08	0.00	**0.00**
			**DJ694 + 3.3 + DJ1077**	
DJ694	Bg2;3.3,1077	13-Nov-07	0.04	**4.65**

L1 larvae

Parents		Date	Specific activity ΔmOD/min/µg protein	% DJ694 control
**Male**	**Female**			
DJ694	Bg2;3.3,1077	13-Nov-07	0.04	**4.68**
DJ694	Bg2;3.3,1077	13-Nov-07	0.09	**10.00**
DJ694	Bg2;3.3,1077	30-Nov-07	0.09	**13.88**
DJ694	Bg2;3.3,1077	30-Nov-07	0.07	**11.27**
DJ694	Bg2;3.3,1077	30-Nov-07	0.09	**14.67**
DJ694;3.3,1077	Bg2	22-Feb-08	0.08	**11.63**
DJ694;3.3,1077	Bg2	22-Feb-08	0.17	**23.85**
DJ694;3.3,1077	Bg2	22-Feb-08	0.14	**19.63**
			**DJ694 + 3.3 + DJ1079**	
DJ694	Bg2;3.3,1079	13-Nov-07	0.26	27.67
DJ694	Bg2;3.3,1079	13-Nov-07	0.30	32.37
DJ694	Bg2;3.3,1079	13-Nov-07	0.75	81.08
DJ694	Bg2;3.3,1079	30-Nov-07	0.72	116.18
DJ694	Bg2;3.3,1079	30-Nov-07	0.11	17.25
DJ694	Bg2;3.3,1079	30-Nov-07	0.19	30.47
			**DJ694 + 2.1 + DJ1077 + DJ1080**	
DJ694;2.1	Bg2;1077,1080	03-Aug-07	0.07	**4.80**
DJ694;2.1	Bg2;1077,1080	03-Aug-07	0.07	**4.72**
DJ694;2.1	Bg2;1077,1080	03-Aug-07	0.07	**4.67**
DJ694;2.1	Bg2;1077,1080	15-Aug-07	0.00	**0.00**
DJ694;2.1	Bg2;1077,1080	15-Aug-07	0.01	**2.28**
DJ694;2.1	Bg2;1077,1080	15-Aug-07	0.00	**0.00**
DJ694;2.1	Bg2;1077,1080	12-Jul-07	0.13	**15.05**
DJ694;2.1	Bg2;1077,1080	12-Jul-07	0.06	**6.79**
DJ694;2.1	Bg2;1077,1080	12-Jul-07	0.19	**22.33**
			**DJ694 + 2.1 + DJ1080 + DJ1083**	
DJ694;2.1	Bg2;1080,1083	03-Aug-07	0.19	**13.59**
DJ694;2.1	Bg2;1080,1083	03-Aug-07	0.14	**9.99**
DJ694;2.1	Bg2;1080,1083	03-Aug-07	0.19	**13.58**
DJ694;2.1	Bg2;1080,1083	15-Aug-07	0.02	**4.59**
DJ694;2.1	Bg2;1080,1083	15-Aug-07	0.02	**4.32**
DJ694;2.1	Bg2;1080,1083	15-Aug-07	0.05	**10.94**
DJ694;2.1	Bg2;1080,1083	12-Jul-07	0.00	**0.00**
DJ694;2.1	Bg2;1080,1083	12-Jul-07	0.00	**0.00**
DJ694;2.1	Bg2;1080,1083	12-Jul-07	0.02	**2.44**
			**DJ694 + 3.3 + DJ1077 + DJ1080**	
DJ694;3.3	Bg2;1077,1080	03-Aug-07	0.08	**5.73**
DJ694;3.3	Bg2;1077,1080	03-Aug-07	0.10	**6.87**
DJ694;3.3	Bg2;1077,1080	03-Aug-07	0.10	**7.04**

L1 larvae

Parents		Date	Specific activity ΔmOD/min/μg protein	% DJ694 control
**Male**	**Female**			
DJ694;3.3	Bg2;1077,1080	15-Aug-07	0.03	**6.23**
DJ694;3.3	Bg2;1077,1080	15-Aug-07	0.01	**1.47**
DJ694;3.3	Bg2;1077,1080	15-Aug-07	0.03	**6.60**
DJ694;3.3	Bg2;1077,1080	12-Jul-07	0.02	**2.04**
DJ694;3.3	Bg2;1077,1080	12-Jul-07	0.02	**2.37**
DJ694;3.3	Bg2;1077,1080	12-Jul-07	0.01	**1.04**
			**DJ694 + 3.3 + DJ1080 + DJ1083**	
DJ694;3.3	Bg2;1080,1083	03-Aug-07	0.11	**7.92**
DJ694;3.3	Bg2;1080,1083	03-Aug-07	0.21	**15.26**
DJ694;3.3	Bg2;1080,1083	03-Aug-07	0.16	**11.45**
DJ694;3.3	Bg2;1080,1083	15-Aug-07	0.07	**14.67**
DJ694;3.3	Bg2;1080,1083	15-Aug-07	0.05	**9.70**
DJ694;3.3	Bg2;1080,1083	15-Aug-07	0.05	**10.46**
DJ694;3.3	Bg2;1080,1083	12-Jul-07	0.15	**17.99**
DJ694;3.3	Bg2;1080,1083	12-Jul-07	0.11	**13.50**
DJ694;3.3	Bg2;1080,1083	12-Jul-07	0.13	**15.42**
			**DJ694 + 2.1 + 3.3 + DJ1077**	
DJ694;1077	Bg2;2.1,3.3	03-Aug-07	0.19	**13.49**
DJ694;1077	Bg2;2.1,3.3	03-Aug-07	0.30	**21.41**
DJ694;1077	Bg2;2.1,3.3	03-Aug-07	0.13	**9.56**
DJ694;1077	Bg2;2.1,3.3	15-Aug-07	0.07	**14.74**
DJ694;1077	Bg2;2.1,3.3	15-Aug-07	0.06	**13.43**
DJ694;1077	Bg2;2.1,3.3	15-Aug-07	0.07	**13.64**
DJ694;1077	Bg2;2.1,3.3	04-Jul-07	0.13	**29.57**
DJ694;1077	Bg2;2.1,3.3	04-Jul-07	0.16	**35.05**
DJ694;1077	Bg2;2.1,3.3	04-Jul-07	0.11	**24.72**
DJ694;1077	Bg2;2.1,3.3	12-Jul-07	0.05	**6.07**
DJ694;1077	Bg2;2.1,3.3	12-Jul-07	0.04	**5.11**
DJ694;1077	Bg2;2.1,3.3	12-Jul-07	0.03	**3.27**
			**DJ694 + 2.1 + 3.3 + DJ1080**	
DJ694;1080	Bg2;2.1,3.3	03-Aug-07	0.17	**12.01**
DJ694;1080	Bg2;2.1,3.3	03-Aug-07	0.30	**21.52**
DJ694;1080	Bg2;2.1,3.3	03-Aug-07	0.25	**18.07**
DJ694;1080	Bg2;2.1,3.3	15-Aug-07	0.15	32.19
DJ694;1080	Bg2;2.1,3.3	15-Aug-07	0.26	53.40
DJ694;1080	Bg2;2.1,3.3	15-Aug-07	0.29	59.33
DJ694;1080	Bg2;2.1,3.3	04-Jul-07	0.13	**28.35**
DJ694;1080	Bg2;2.1,3.3	04-Jul-07	0.14	**30.37**
DJ694;1080	Bg2;2.1,3.3	04-Jul-07	0.16	**34.63**

L1 larvae

Parents		Date	Specific activity ΔmOD/min/µg protein	% DJ694 control
Male	Female			
DJ694;1080	Bg2;2.1,3.3	12-Jul-07	0.46	**53.64**
DJ694;1080	Bg2;2.1,3.3	12-Jul-07	0.44	**51.52**
DJ694;1080	Bg2;2.1,3.3	12-Jul-07	0.28	**33.10**
			**DJ694 + 2 copies 2.1 + 2 copies DJ1077**	
DJ694;2.1,1077	Bg2;2.1,1077	13-Nov-07	0.08	**8.68**
DJ694;2.1,1077	Bg2;2.1,1077	13-Nov-07	0.03	**3.26**
DJ694;2.1,1077	Bg2;2.1,1077	13-Nov-07	0.06	**6.80**
DJ694;2.1,1077	Bg2;2.1,1077	30-Nov-07	0.00	**0.51**
DJ694;2.1,1077	Bg2;2.1,1077	30-Nov-07	0.02	**2.67**
DJ694;2.1,1077	Bg2;2.1,1077	30-Nov-07	0.01	**0.85**
DJ694;2.1,1077	Bg2;2.1,1077	19-Dec-07	0.01	**2.38**
DJ694;2.1,1077	Bg2;2.1,1077	19-Dec-07	0.01	**0.99**
DJ694;2.1,1077	Bg2;2.1,1077	19-Dec-07	0.01	**0.95**
DJ694;2.1,1077	Bg2;2.1,1077	19-Dec-07	0.00	**0.34**
DJ694;2.1,1077	Bg2;2.1,1077	19-Dec-07	0.02	**2.58**
DJ694;2.1,1077	Bg2;2.1,1077	10-Feb-08	0.01	**2.23**
DJ694;2.1,1077	Bg2;2.1,1077	10-Feb-08	0.02	**3.55**
DJ694;2.1,1077	Bg2;2.1,1077	10-Feb-08	0.00	**0.00**
DJ694;2.1,1077	Bg2;2.1,1077	22-Feb-08	0.00	**0.26**
DJ694;2.1,1077	Bg2;2.1,1077	22-Feb-08	0.01	**0.72**
DJ694;2.1,1077	Bg2;2.1,1077	22-Feb-08	0.00	**0.00**
DJ694;2.1,1077	Bg2;2.1,1077	19-Dec-07	0.43	72.77
			**DJ694 + 2 copies 2.1 + DJ1077 + DJ1079**	
DJ694;2.1,1077	Bg2;2.1,1079(13)	13-Nov-07	0.10	**10.71**
DJ694;2.1,1077	Bg2;2.1,1079(13)	13-Nov-07	0.16	**16.84**
DJ694;2.1,1077	Bg2;2.1,1079(13)	30-Nov-07	0.15	**23.59**
DJ694;2.1,1077	Bg2;2.1,1079(13)	30-Nov-07	0.16	**25.48**
DJ694;2.1,1077	Bg2;2.1,1079(13)	30-Nov-07	0.03	**5.41**
DJ694;2.1,1077	Bg2;2.1,1079(14)	13-Nov-07	0.02	**1.96**
DJ694;2.1,1077	Bg2;2.1,1079(14)	13-Nov-07	0.06	**6.97**
DJ694;2.1,1077	Bg2;2.1,1079(14)	13-Nov-07	0.02	**2.06**
DJ694;2.1,1077	Bg2;2.1,1079(14)	30-Nov-07	0.05	**8.59**
DJ694;2.1,1077	Bg2;2.1,1079(14)	30-Nov-07	0.13	**21.61**
DJ694;2.1,1077	Bg2;2.1,1079(14)	30-Nov-07	0.04	**5.85**
DJ694;2.1,1077	Bg2;2.1,1079(14)	19-Dec-07	0.09	**15.75**
DJ694;2.1,1077	Bg2;2.1,1079(14)	19-Dec-07	0.07	**12.61**
DJ694;2.1,1077	Bg2;2.1,1079(14)	19-Dec-07	0.00	**0.75**
DJ694;2.1,1077	Bg2;2.1,1079(14)	19-Dec-07	0.02	**3.38**
DJ694;2.1,1077	Bg2;2.1,1079(14)	19-Dec-07	0.02	**3.79**
DJ694;2.1,1079(13)	Bg2;2.1,1077	19-Dec-07	0.00	**0.59**

L1 larvae

Parents		Date	Specific activity ΔmOD/min/µg protein	% DJ694 control
**Male**	**Female**			
DJ694;2.1,1079(13)	Bg2;2.1,1077	19-Dec-07	0.01	**1.85**
DJ694;2.1,1079(13)	Bg2;2.1,1077	19-Dec-07	0.01	**2.43**
DJ694;2.1,1079(14)	Bg2;2.1,1077	19-Dec-07	0.07	**11.87**
DJ694;2.1,1079(14)	Bg2;2.1,1077	19-Dec-07	0.01	**1.00**
DJ694;2.1,1079(14)	Bg2;2.1,1077	19-Dec-07	0.01	**1.45**
DJ694;2.1,1079(14)	Bg2;2.1,1077	19-Dec-07	0.25	**43.09**
DJ694;2.1,1079(14)	Bg2;2.1,1077	19-Dec-07	0.02	**2.74**
DJ694;2.1,1077	Bg2;2.1,1079(13)	10-Feb-08	0.01	**2.53**
DJ694;2.1,1077	Bg2;2.1,1079(13)	10-Feb-08	0.03	**6.74**
DJ694;2.1,1077	Bg2;2.1,1079(13)	10-Feb-08	0.01	**1.24**
DJ694;2.1,1077	Bg2;2.1,1079(13)	22-Feb-08	0.00	**0.00**
DJ694;2.1,1077	Bg2;2.1,1079(13)	22-Feb-08	0.00	**0.17**
DJ694;2.1,1077	Bg2;2.1,1079(13)	22-Feb-08	0.02	**2.34**
DJ694;2.1,1077	Bg2;2.1,1079(14)	10-Feb-08	0.00	**0.00**
DJ694;2.1,1077	Bg2;2.1,1079(14)	10-Feb-08	0.00	**0.00**
DJ694;2.1,1077	Bg2;2.1,1079(14)	10-Feb-08	0.00	**0.00**
DJ694;2.1,1079(13)	Bg2;2.1,1077	10-Feb-08	0.00	**0.57**
DJ694;2.1,1079(13)	Bg2;2.1,1077	10-Feb-08	0.00	**0.15**
DJ694;2.1,1079(13)	Bg2;2.1,1077	10-Feb-08	0.02	**4.63**
DJ694;2.1,1079(14)	Bg2;2.1,1077	10-Feb-08	0.21	**47.42**
DJ694;2.1,1079(14)	Bg2;2.1,1077	10-Feb-08	0.02	**4.20**
DJ694;2.1,1079(14)	Bg2;2.1,1077	10-Feb-08	0.05	**10.98**
DJ694;2.1,1077	Bg2;2.1,1079(14)	19-Dec-07	0.43	73.00
DJ694;2.1,1079(14)	Bg2;2.1,1077	19-Dec-07	0.45	75.73

DJ694 + 2.1 + 3.3 + 2 copies DJ1077

DJ694;2.1,1077	Bg2;3.3,1077	13-Nov-07	0.05	**5.94**
DJ694;2.1,1077	Bg2;3.3,1077	13-Nov-07	0.03	**3.43**
DJ694;2.1,1077	Bg2;3.3,1077	13-Nov-07	0.08	**8.52**
DJ694;2.1,1077	Bg2;3.3,1077	30-Nov-07	0.02	**2.69**
DJ694;2.1,1077	Bg2;3.3,1077	30-Nov-07	0.09	**14.27**
DJ694;2.1,1077	Bg2;3.3,1077	30-Nov-07	0.02	**2.54**
DJ694;2.1,1077	Bg2;3.3,1077	19-Dec-07	0.11	**18.81**
DJ694;2.1,1077	Bg2;3.3,1077	19-Dec-07	0.01	**2.00**
DJ694;2.1,1077	Bg2;3.3,1077	19-Dec-07	0.01	**2.16**
DJ694;2.1,1077	Bg2;3.3,1077	19-Dec-07	0.15	**26.28**
DJ694;2.1,1077	Bg2;3.3,1077	19-Dec-07	0.12	**19.84**
DJ694;3.3,1077	Bg2;2.1,1077	13-Nov-07	0.12	**12.47**
DJ694;3.3,1077	Bg2;2.1,1077	13-Nov-07	0.02	**2.55**
DJ694;3.3,1077	Bg2;2.1,1077	13-Nov-07	0.03	**3.36**
DJ694;3.3,1077	Bg2;2.1,1077	30-Nov-07	0.08	**12.67**

L1 larvae

Parents		Date	Specific activity ΔmOD/min/µg protein	% DJ694 control
**Male**	**Female**			
DJ694;3.3,1077	Bg2;2.1,1077	30-Nov-07	0.03	**5.29**
DJ694;3.3,1077	Bg2;2.1,1077	30-Nov-07	0.01	**2.15**
DJ694;3.3,1077	Bg2;2.1,1077	19-Dec-07	0.01	**2.44**
DJ694;3.3,1077	Bg2;2.1,1077	19-Dec-07	0.01	**1.75**
DJ694;3.3,1077	Bg2;2.1,1077	19-Dec-07	0.02	**3.24**
DJ694;3.3,1077	Bg2;2.1,1077	19-Dec-07	0.09	**16.09**
DJ694;3.3,1077	Bg2;2.1,1077	19-Dec-07	0.08	**13.70**
DJ694;3.3,1077	Bg2;2.1,1077	19-Dec-07	0.07	**11.82**
DJ694;2.1,1077	Bg2;3.3,1077	10-Feb-08	0.01	**2.89**
DJ694;2.1,1077	Bg2;3.3,1077	10-Feb-08	0.00	**0.00**
DJ694;2.1,1077	Bg2;3.3,1077	10-Feb-08	0.00	**0.18**
DJ694;2.1,1077	Bg2;3.3,1077	22-Feb-08	0.04	**5.46**
DJ694;2.1,1077	Bg2;3.3,1077	22-Feb-08	0.00	**0.60**
DJ694;2.1,1077	Bg2;3.3,1077	22-Feb-08	0.01	**1.00**
DJ694;3.3,1077	Bg2;2.1,1077	10-Feb-08	0.01	**2.40**
DJ694;3.3,1077	Bg2;2.1,1077	10-Feb-08	0.01	**1.21**
DJ694;3.3,1077	Bg2;2.1,1077	10-Feb-08	0.00	**0.59**
DJ694;3.3,1077	Bg2;2.1,1077	22-Feb-08	0.02	**2.77**
DJ694;3.3,1077	Bg2;2.1,1077	22-Feb-08	0.01	**1.55**
DJ694;3.3,1077	Bg2;2.1,1077	22-Feb-08	0.01	**1.28**
DJ694;2.1,1077	Bg2;3.3,1077	19-Dec-07	0.02	3.54
			**DJ694 + 2.1 + 3.3 + DJ1077 + DJ1079**	
DJ694;2.1,1077	Bg2;3.3,1079	13-Nov-07	0.02	**1.73**
DJ694;2.1,1077	Bg2;3.3,1079	13-Nov-07	0.01	**1.02**
DJ694;2.1,1077	Bg2;3.3,1079	13-Nov-07	0.00	**0.00**
DJ694;2.1,1077	Bg2;3.3,1079	30-Nov-07	0.03	**5.13**
DJ694;2.1,1077	Bg2;3.3,1079	30-Nov-07	0.00	**0.00**
DJ694;2.1,1077	Bg2;3.3,1079	30-Nov-07	0.02	**3.78**
DJ694;2.1,1077	Bg2;3.3,1079	19-Dec-07	0.01	**2.42**
DJ694;2.1,1077	Bg2;3.3,1079	19-Dec-07	0.01	**2.08**
DJ694;2.1,1077	Bg2;3.3,1079	19-Dec-07	0.02	**3.79**
DJ694;2.1,1077	Bg2;3.3,1079	19-Dec-07	0.01	**2.49**
DJ694;2.1,1077	Bg2;3.3,1079	19-Dec-07	0.04	**6.19**
DJ694;2.1,1079(13)	Bg2;3.3,1077	19-Dec-07	0.04	**7.31**
DJ694;2.1,1079(13)	Bg2;3.3,1077	19-Dec-07	0.05	**8.29**
DJ694;2.1,1079(13)	Bg2;3.3,1077	19-Dec-07	0.03	**5.57**
DJ694;2.1,1079(14)	Bg2;3.3,1077	19-Dec-07	0.03	**4.67**
DJ694;2.1,1079(14)	Bg2;3.3,1077	19-Dec-07	0.02	**2.90**
DJ694;2.1,1079(14)	Bg2;3.3,1077	19-Dec-07	0.02	**3.03**
DJ694;2.1,1079(14)	Bg2;3.3,1077	19-Dec-07	0.12	**21.05**

L1 larvae

Parents		Date	Specific activity ΔmOD/min/µg protein	% DJ694 control
Male	Female			
DJ694;2.1,1079(14)	Bg2;3.3,1077	19-Dec-07	0.07	**12.52**
DJ694;3.3,1077	Bg2;2.1,1079(13)	13-Nov-07	0.05	**5.64**
DJ694;3.3,1077	Bg2;2.1,1079(13)	13-Nov-07	0.06	**6.82**
DJ694;3.3,1077	Bg2;2.1,1079(13)	13-Nov-07	0.04	**4.27**
DJ694;3.3,1077	Bg2;2.1,1079(13)	30-Nov-07	0.06	**9.95**
DJ694;3.3,1077	Bg2;2.1,1079(13)	30-Nov-07	0.04	**6.85**
DJ694;3.3,1077	Bg2;2.1,1079(13)	30-Nov-07	0.08	**12.12**
DJ694;3.3,1077	Bg2;2.1,1079(13)	19-Dec-07	0.04	**6.28**
DJ694;3.3,1077	Bg2;2.1,1079(13)	19-Dec-07	0.02	**3.00**
DJ694;3.3,1077	Bg2;2.1,1079(13)	19-Dec-07	0.02	**3.74**
DJ694;3.3,1077	Bg2;2.1,1079(14)	13-Nov-07	0.04	**4.58**
DJ694;3.3,1077	Bg2;2.1,1079(14)	13-Nov-07	0.04	**4.07**
DJ694;3.3,1077	Bg2;2.1,1079(14)	13-Nov-07	0.07	**7.19**
DJ694;3.3,1077	Bg2;2.1,1079(14)	30-Nov-07	0.04	**7.01**
DJ694;3.3,1077	Bg2;2.1,1079(14)	30-Nov-07	0.02	**3.26**
DJ694;3.3,1077	Bg2;2.1,1079(14)	30-Nov-07	0.02	**2.48**
DJ694;3.3,1077	Bg2;2.1,1079(14)	19-Dec-07	0.03	**4.78**
DJ694;3.3,1077	Bg2;2.1,1079(14)	19-Dec-07	0.01	**1.60**
DJ694;3.3,1077	Bg2;2.1,1079(14)	19-Dec-07	0.01	**1.56**
DJ694;3.3,1077	Bg2;2.1,1079(14)	19-Dec-07	0.05	**8.97**
DJ694;3.3,1077	Bg2;2.1,1079(14)	19-Dec-07	0.05	**8.25**
DJ694;3.3,1077	Bg2;2.1,1079(14)	19-Dec-07	0.03	**4.44**
DJ694;1077,1079	Bg2;2.1,3.3	15-Jun-07	0.12	**3.37**
DJ694;1077,1079	Bg2;2.1,3.3	15-Jun-07	0.24	**6.87**
DJ694;1077,1079	Bg2;2.1,3.3	15-Jun-07	0.11	**3.05**
DJ694;1077,1079	Bg2;2.1,3.3	15-Jun-07	0.18	**5.14**
DJ694;1077,1079	Bg2;2.1,3.3	04-Jul-07	0.09	**19.57**
DJ694;1077,1079	Bg2;2.1,3.3	04-Jul-07	0.18	**38.73**
DJ694;1077,1079	Bg2;2.1,3.3	04-Jul-07	0.09	**19.47**
DJ694;2.1,1077	Bg2;3.3,1079	10-Feb-08	0.00	**0.39**
DJ694;2.1,1077	Bg2;3.3,1079	10-Feb-08	0.00	**0.79**
DJ694;2.1,1077	Bg2;3.3,1079	10-Feb-08	0.00	**0.65**
DJ694;2.1,1079(13)	Bg2;3.3,1077	10-Feb-08	0.00	**0.00**
DJ694;2.1,1079(13)	Bg2;3.3,1077	10-Feb-08	0.00	**1.06**
DJ694;2.1,1079(13)	Bg2;3.3,1077	10-Feb-08	0.00	**-0.08**
DJ694;2.1,1079(13)	Bg2;3.3,1077	22-Feb-08	0.00	**0.51**
DJ694;2.1,1079(13)	Bg2;3.3,1077	22-Feb-08	0.01	**1.22**
DJ694;2.1,1079(13)	Bg2;3.3,1077	22-Feb-08	0.01	**1.07**
DJ694;2.1,1079(14)	Bg2;3.3,1077	10-Feb-08	0.02	**4.62**
DJ694;2.1,1079(14)	Bg2;3.3,1077	10-Feb-08	0.07	**15.03**

L1 larvae

Parents		Date	Specific activity ΔmOD/min/μg protein	% DJ694 control
Male	Female			
DJ694;2.1,1079(14)	Bg2;3.3,1077	10-Feb-08	0.07	**16.94**
DJ694;3.3,1077	Bg2;2.1,1079(13)	10-Feb-08	0.00	**0.88**
DJ694;3.3,1077	Bg2;2.1,1079(13)	10-Feb-08	0.00	**0.74**
DJ694;3.3,1077	Bg2;2.1,1079(13)	10-Feb-08	0.01	**2.63**
DJ694;3.3,1077	Bg2;2.1,1079(14)	10-Feb-08	0.00	**1.12**
DJ694;3.3,1077	Bg2;2.1,1079(14)	10-Feb-08	0.01	**1.67**
DJ694;3.3,1077	Bg2;2.1,1079(14)	10-Feb-08	0.01	**2.99**
DJ694;2.1,1077	Bg2;3.3,1079	19-Dec-07	0.10	17.53
DJ694;2.1,1079(14)	Bg2;3.3,1077	19-Dec-07	0.19	32.56

DJ694 + 2 copies 2.1 + 2 copies DJ1079

DJ694;2.1,1079(13)	Bg2;2.1,1079(14)	19-Dec-07	0.05	**8.32**
DJ694;2.1,1079(13)	Bg2;2.1,1079(14)	19-Dec-07	0.05	**8.62**
DJ694;2.1,1079(13)	Bg2;2.1,1079(14)	19-Dec-07	0.01	**2.25**
DJ694;2.1,1079(14)	Bg2;2.1,1079(13)	19-Dec-07	0.08	**14.28**
DJ694;2.1,1079(14)	Bg2;2.1,1079(13)	19-Dec-07	0.04	**6.32**
DJ694;2.1,1079(14)	Bg2;2.1,1079(14)	19-Dec-07	0.10	**16.78**
DJ694;2.1,1079(14)	Bg2;2.1,1079(14)	19-Dec-07	0.04	**7.27**
DJ694;2.1,1079(14)	Bg2;2.1,1079(14)	19-Dec-07	0.03	**4.78**
DJ694;2.1,1079(14)	Bg2;2.1,1079(14)	19-Dec-07	0.06	**10.07**
DJ694;2.1,1079(13)	Bg2;2.1,1079(13)	10-Feb-08	0.00	**0.05**
DJ694;2.1,1079(13)	Bg2;2.1,1079(13)	10-Feb-08	0.01	**2.70**
DJ694;2.1,1079(13)	Bg2;2.1,1079(13)	10-Feb-08	0.01	**1.38**
DJ694;2.1,1079(13)	Bg2;2.1,1079(14)	10-Feb-08	0.00	**0.00**
DJ694;2.1,1079(13)	Bg2;2.1,1079(14)	10-Feb-08	0.01	**1.25**
DJ694;2.1,1079(13)	Bg2;2.1,1079(14)	10-Feb-08	0.01	**2.15**
DJ694;2.1,1079(14)	Bg2;2.1,1079(13)	10-Feb-08	0.01	**3.09**
DJ694;2.1,1079(14)	Bg2;2.1,1079(13)	10-Feb-08	0.03	**7.32**
DJ694;2.1,1079(14)	Bg2;2.1,1079(13)	10-Feb-08	0.04	**9.16**
DJ694;2.1,1079(14)	Bg2;2.1,1079(14)	10-Feb-08	0.03	**6.16**
DJ694;2.1,1079(14)	Bg2;2.1,1079(14)	10-Feb-08	0.04	**9.07**
DJ694;2.1,1079(14)	Bg2;2.1,1079(14)	10-Feb-08	0.01	**2.82**
DJ694;2.1,1079(14)	Bg2;2.1,1079(13)	19-Dec-07	0.35	59.85
DJ694;2.1,1079(14)	Bg2;2.1,1079(14)	19-Dec-07	0.16	27.82
DJ694;2.1,1079(14)	Bg2;2.1,1079(14)	19-Dec-07	0.98	166.18

DJ694 + 2.1 + 3.3 + 2 copies DJ1079

DJ694;2.1,1079(13)	Bg2;3.3,1079	19-Dec-07	0.20	**33.24**
DJ694;2.1,1079(13)	Bg2;3.3,1079	19-Dec-07	0.08	**14.37**
DJ694;2.1,1079(13)	Bg2;3.3,1079	19-Dec-07	0.03	**5.05**
DJ694;2.1,1079(14)	Bg2;3.3,1079	19-Dec-07	0.02	**3.57**
DJ694;2.1,1079(14)	Bg2;3.3,1079	19-Dec-07	0.08	**12.95**

L1 larvae

Parents		Date	Specific activity ΔmOD/min/μg protein	% DJ694 control
Male	Female			
DJ694;2.1,1079(14)	Bg2;3.3,1079	19-Dec-07	0.04	**6.18**
DJ694;2.1,1079(14)	Bg2;3.3,1079	19-Dec-07	0.01	**1.30**
DJ694;2.1,1079(14)	Bg2;3.3,1079	19-Dec-07	0.01	**2.26**
DJ694;2.1,1079(14)	Bg2;3.3,1079	19-Dec-07	0.01	**1.17**
DJ694;2.1,1079(13)	Bg2;3.3,1079	10-Feb-08	0.01	**1.20**
DJ694;2.1,1079(13)	Bg2;3.3,1079	10-Feb-08	0.00	**0.41**
DJ694;2.1,1079(13)	Bg2;3.3,1079	10-Feb-08	0.00	**0.59**
DJ694;2.1,1079(14)	Bg2;3.3,1079	10-Feb-08	0.00	**0.00**
DJ694;2.1,1079(14)	Bg2;3.3,1079	10-Feb-08	0.03	**7.11**
DJ694;2.1,1079(14)	Bg2;3.3,1079	10-Feb-08	0.02	**4.05**
			**DJ694 + 2 copies 3.3 + 2 copies DJ1077**	
DJ694;3.3,1077	Bg2;3.3,1077	13-Nov-07	0.03	**3.58**
DJ694;3.3,1077	Bg2;3.3,1077	13-Nov-07	0.04	**4.17**
DJ694;3.3,1077	Bg2;3.3,1077	13-Nov-07	0.23	**24.58**
DJ694;3.3,1077	Bg2;3.3,1077	30-Nov-07	0.07	**11.80**
DJ694;3.3,1077	Bg2;3.3,1077	30-Nov-07	0.02	**2.92**
DJ694;3.3,1077	Bg2;3.3,1077	30-Nov-07	0.07	**11.80**
DJ694;3.3,1077	Bg2;3.3,1077	19-Dec-07	0.09	**15.24**
DJ694;3.3,1077	Bg2;3.3,1077	19-Dec-07	0.03	**4.29**
DJ694;3.3,1077	Bg2;3.3,1077	19-Dec-07	0.01	**2.23**
DJ694;3.3,1077	Bg2;3.3,1077	19-Dec-07	0.03	**4.89**
DJ694;3.3,1077	Bg2;3.3,1077	10-Feb-08	0.04	**8.49**
DJ694;3.3,1077	Bg2;3.3,1077	10-Feb-08	0.00	**0.66**
DJ694;3.3,1077	Bg2;3.3,1077	10-Feb-08	0.05	**10.95**
DJ694;3.3,1077	Bg2;3.3,1077	22-Feb-08	0.04	**5.61**
DJ694;3.3,1077	Bg2;3.3,1077	22-Feb-08	0.00	**0.38**
DJ694;3.3,1077	Bg2;3.3,1077	22-Feb-08	0.00	**0.71**
DJ694;3.3,1077	Bg2;3.3,1077	19-Dec-07	0.14	23.44
DJ694;3.3,1077	Bg2;3.3,1077	19-Dec-07	0.18	30.35
			**DJ694 + 2 copies 3.3 + DJ1077 + DJ1079**	
DJ694;3.3,1077	Bg2;3.3,1079	13-Nov-07	0.13	**14.07**
DJ694;3.3,1077	Bg2;3.3,1079	13-Nov-07	0.13	**14.33**
DJ694;3.3,1077	Bg2;3.3,1079	13-Nov-07	0.03	**3.12**
DJ694;3.3,1077	Bg2;3.3,1079	30-Nov-07	0.18	**28.25**
DJ694;3.3,1077	Bg2;3.3,1079	30-Nov-07	0.09	**14.83**
DJ694;3.3,1077	Bg2;3.3,1079	30-Nov-07	0.02	**3.58**
DJ694;3.3,1077	Bg2;3.3,1079	19-Dec-07	0.02	**4.21**
DJ694;3.3,1077	Bg2;3.3,1079	19-Dec-07	0.01	**1.59**
DJ694;3.3,1077	Bg2;3.3,1079	19-Dec-07	0.00	**0.57**
DJ694;3.3,1077	Bg2;3.3,1079	19-Dec-07	0.01	**2.31**

L1 larvae

Parents		Date	Specific activity ΔmOD/min/µg protein	% DJ694 control
Male	Female			
DJ694;3.3,1077	Bg2;3.3,1079	19-Dec-07	0.02	**3.61**
DJ694;3.3,1077	Bg2;3.3,1079	19-Dec-07	0.01	**2.17**
DJ694;3.3,1077	Bg2;3.3,1079	10-Feb-08	0.01	**1.18**
DJ694;3.3,1077	Bg2;3.3,1079	10-Feb-08	0.00	**0.42**
DJ694;3.3,1077	Bg2;3.3,1079	10-Feb-08	0.02	**4.07**
			**DJ694 + 2.1 + 3.3 + DJ1077 + DJ1080**	
DJ694;1077,1080	Bg2;2.1,3.3	15-Jun-07	0.39	**11.16**
DJ694;1077,1080	Bg2;2.1,3.3	15-Jun-07	0.36	**10.10**
DJ694;1077,1080	Bg2;2.1,3.3	15-Jun-07	0.46	**12.98**
DJ694;1077,1080	Bg2;2.1,3.3	15-Jun-07	0.33	**9.25**
DJ694;1077,1080	Bg2;2.1,3.3	04-Jul-07	0.21	**45.21**
DJ694;1077,1080	Bg2;2.1,3.3	04-Jul-07	0.05	**11.90**
DJ694;1077,1080	Bg2;2.1,3.3	04-Jul-07	0.33	**71.63**
DJ694;2.1,3.3	Bg2;1077,1080	31-May-07	0.02	**1.58**
DJ694;2.1,3.3	Bg2;1077,1080	31-May-07	0.00	**0.00**
DJ694;2.1,3.3	Bg2;1077,1080	31-May-07	0.07	**5.90**
DJ694;2.1,3.3	Bg2;1077,1080	31-May-07	0.01	**0.47**
			**DJ694 + 2.1 + 2 copies DJ1077 + DJ1079**	
DJ694;1077,1079	Bg2;2.1,1077	19-Dec-07	0.02	**4.21**
DJ694;1077,1079	Bg2;2.1,1077	19-Dec-07	0.05	**8.73**
DJ694;1077,1079	Bg2;2.1,1077	19-Dec-07	0.01	**1.21**
			**DJ694 + 2.1 + DJ1077 + 2 copies DJ1079**	
DJ694;1077,1079	Bg2;2.1,1079(14)	19-Dec-07	0.01	**2.41**
DJ694;1077,1079	Bg2;2.1,1079(14)	19-Dec-07	0.01	**1.03**
DJ694;1077,1079	Bg2;2.1,1079(14)	19-Dec-07	0.00	**0.72**
			**DJ694 + 3.3 + 2 copies DJ1077 + DJ1079**	
DJ694;1077,1079	Bg2;3.3,1077	19-Dec-07	0.01	**0.85**
DJ694;1077,1079	Bg2;3.3,1077	19-Dec-07	0.00	**0.47**
DJ694;1077,1079	Bg2;3.3,1077	19-Dec-07	0.00	**0.47**
			**DJ694 + 3.3 + DJ1077 + 2 copies DJ1079**	
DJ694;1077,1079	Bg2;3.3,1079	19-Dec-07	0.00	**0.00**
DJ694;1077,1079	Bg2;3.3,1079	19-Dec-07	0.01	**1.60**
DJ694;1077,1079	Bg2;3.3,1079	19-Dec-07	0.00	**0.32**
			**DJ694 + 2.1 + 2 copies DJ1077 + DJ1080**	
DJ694;1077,1080	Bg2;2.1,1077	10-Feb-08	0.01	**2.44**
DJ694;1077,1080	Bg2;2.1,1077	10-Feb-08	0.03	**7.19**
DJ694;1077,1080	Bg2;2.1,1077	10-Feb-08	0.02	**3.44**
DJ694;2.1,1077	Bg2;1077,1080	10-Feb-08	0.01	**1.78**
DJ694;2.1,1077	Bg2;1077,1080	10-Feb-08	0.00	**0.04**

L1 larvae

Parents		Date	Specific activity ΔmOD/min/µg protein	% DJ694 control
Male	Female			
DJ694;2.1,1077	Bg2;1077,1080	10-Feb-08	0.02	**3.57**
			**DJ694 + 2.1 + DJ1077 + DJ1079 + DJ1080**	
DJ694;1077,1080	Bg2;2.1,1079(13)	10-Feb-08	0.06	**14.46**
DJ694;1077,1080	Bg2;2.1,1079(13)	10-Feb-08	0.03	**6.18**
DJ694;1077,1080	Bg2;2.1,1079(13)	10-Feb-08	0.31	**69.83**
DJ694;1077,1080	Bg2;2.1,1079(14)	10-Feb-08	0.03	**6.59**
DJ694;1077,1080	Bg2;2.1,1079(14)	10-Feb-08	0.03	**7.35**
DJ694;1077,1080	Bg2;2.1,1079(14)	10-Feb-08	0.06	**14.54**
DJ694;2.1,1079(14)	Bg2;1077,1080	10-Feb-08	0.13	**29.15**
DJ694;2.1,1079(14)	Bg2;1077,1080	10-Feb-08	0.10	**22.92**
DJ694;2.1,1079(14)	Bg2;1077,1080	10-Feb-08	0.04	**8.86**
			**DJ694 + 3.3 + 2 copies DJ1077 + DJ1080**	
DJ694;1077,1080	Bg2;3.3,1077	10-Feb-08	0.01	**2.64**
DJ694;1077,1080	Bg2;3.3,1077	10-Feb-08	0.01	**2.15**
DJ694;1077,1080	Bg2;3.3,1077	10-Feb-08	0.00	**0.17**
DJ694;3.3,1077	Bg2;1077,1080	10-Feb-08	0.00	**-0.07**
DJ694;3.3,1077	Bg2;1077,1080	10-Feb-08	0.01	**1.27**
DJ694;3.3,1077	Bg2;1077,1080	10-Feb-08	0.00	**0.57**
			**DJ694 + 3.3 + DJ1077 + DJ1079 + DJ1080**	
DJ694;1077,1080	Bg2;3.3,1079	10-Feb-08	0.04	**9.73**
DJ694;1077,1080	Bg2;3.3,1079	10-Feb-08	0.04	**8.36**
DJ694;1077,1080	Bg2;3.3,1079	10-Feb-08	0.02	**5.03**
			**DJ694 + 2.1 + DJ1077 + DJ1080 + DJ1083**	
DJ694;2.1,1077	Bg2;1080,1083	10-Feb-08	0.00	**0.00**
DJ694;2.1,1077	Bg2;1080,1083	10-Feb-08	0.00	**0.90**
DJ694;2.1,1077	Bg2;1080,1083	10-Feb-08	0.01	**1.44**
			**DJ694 + 2.1 + DJ1077 + DJ1079 + DJ1080**	
DJ694;2.1,1079(13)	Bg2;1077,1080	10-Feb-08	0.01	**2.07**
DJ694;2.1,1079(13)	Bg2;1077,1080	10-Feb-08	0.00	**0.71**
DJ694;2.1,1079(13)	Bg2;1077,1080	10-Feb-08	0.00	**0.34**
			**DJ694 + 2.1 + DJ1079 + DJ1080 + DJ1083**	
DJ694;2.1,1079(13)	Bg2;1080,1083	10-Feb-08	0.00	**0.52**
DJ694;2.1,1079(13)	Bg2;1080,1083	10-Feb-08	0.00	**0.00**
DJ694;2.1,1079(13)	Bg2;1080,1083	10-Feb-08	0.00	**0.00**
DJ694;2.1,1079(14)	Bg2;1080,1083	10-Feb-08	0.17	**38.70**
DJ694;2.1,1079(14)	Bg2;1080,1083	10-Feb-08	0.04	**9.03**
			**DJ694 + 3.3 + DJ1077 + DJ1080 + DJ1083**	
DJ694;3.3,1077	Bg2;1080,1083	10-Feb-08	0.00	**0.97**
DJ694;3.3,1077	Bg2;1080,1083	10-Feb-08	0.02	**3.84**

L1 larvae

Parents		Date	Specific activity ΔmOD/min/µg protein	% DJ694 control
**Male**	**Female**			
DJ694;3.3,1077	Bg2;1080,1083	10-Feb-08	0.01	**1.82**

Supplemental Table 5: UAS-lacZ assay L3 data set.

% DJ694 control: specific activity normalized to the positive control, bolded values are significantly different ($p<0.05$) from the positive control.

L3 larvae

Parents		Date	Specific activity ΔmOD/min/μg protein	% DJ694 control
Male	Female			
			**DJ694 + DJ1077**	
DJ694;1077	Bg2	07-06-15	6.66	**48.45**
DJ694;1077	Bg2	07-06-15	6.72	**48.91**
DJ694;1077	Bg2	07-06-15	6.31	**45.87**
DJ694;1077	Bg2	07-07-04	2.91	79.29
DJ694;1077	Bg2	07-07-04	1.74	47.37
DJ694;1077	Bg2	07-07-04	3.27	88.99
DJ694;1077	Bg2	07-05-31	1.59	**48.94**
DJ694;1077	Bg2	07-05-31	2.34	**71.86**
DJ694;1077	Bg2	07-05-31	2.30	**70.58**
DJ694;1077	Bg2	07-05-31	2.26	**69.53**
DJ694;1077	Bg2	07-05-31	1.45	**44.47**
			**DJ694 + DJ1080**	
DJ694;1080	Bg2	07-06-15	6.22	**45.23**
DJ694;1080	Bg2	07-06-15	9.28	**67.52**
DJ694;1080	Bg2	07-06-15	9.00	**65.49**
DJ694;1080	Bg2	07-05-31	1.81	**55.72**
DJ694;1080	Bg2	07-05-31	1.74	**53.43**
DJ694;1080	Bg2	07-05-31	1.41	**43.18**
DJ694;1080	Bg2	07-05-31	1.68	**51.60**
DJ694;1080	Bg2	07-05-31	2.22	**68.14**
DJ694;1080	Bg2	07-07-04	2.22	60.38
DJ694;1080	Bg2	07-07-04	1.07	29.06
DJ694;1080	Bg2	07-07-04	4.68	127.42
			**DJ694 + DJ1077 + DJ1080**	
DJ694	Bg2;1077,1080	07-08-03	1.86	61.46
DJ694	Bg2;1077,1080	07-08-03	2.65	87.58
DJ694	Bg2;1077,1080	07-08-03	2.04	67.37
DJ694	Bg2;1077,1080	07-08-15	1.63	139.03
DJ694	Bg2;1077,1080	07-08-15	0.82	69.63
DJ694	Bg2;1077,1080	07-08-15	1.25	107.03
DJ694	Bg2;1077,1080	07-07-30	1.38	91.59
DJ694	Bg2;1077,1080	07-07-30	1.11	73.76
DJ694	Bg2;1077,1080	07-07-30	1.12	74.23
			**DJ694 + DJ1080 + DJ1083**	
DJ694	Bg2;1080,1083	07-06-15	11.19	**81.39**
DJ694	Bg2;1080,1083	07-06-15	5.20	**37.85**
DJ694	Bg2;1080,1083	07-06-15	7.24	**52.68**
DJ694	Bg2;1080,1083	07-07-04	3.06	**83.43**

L3 larvae

Parents		Date	Specific activity ΔmOD/min/µg protein	% DJ694 control
**Male**	**Female**			
DJ694	Bg2;1080,1083	07-07-04	2.88	**78.39**
DJ694	Bg2;1080,1083	07-07-04	2.84	**77.29**
DJ694	Bg2;1080,1083	07-07-12	1.15	74.09
DJ694	Bg2;1080,1083	07-07-12	1.10	70.90
DJ694	Bg2;1080,1083	07-07-12	1.60	102.61
			**DJ694 + DJ1077 + DJ1079**	
DJ694;1077,1079	Bg2	07-08-15	0.89	**76.33**
DJ694;1077,1079	Bg2	07-08-15	0.84	**71.27**
DJ694;1077,1079	Bg2	07-08-15	1.07	**91.17**
DJ694;1077,1079	Bg2	07-06-15	3.86	**28.07**
DJ694;1077,1079	Bg2	07-06-15	4.17	**30.33**
DJ694;1077,1079	Bg2	07-06-15	3.70	**26.93**
DJ694;1077,1079	Bg2	07-07-04	1.28	**34.79**
DJ694;1077,1079	Bg2	07-07-04	2.65	**72.29**
DJ694;1077,1079	Bg2	07-07-04	2.55	**69.33**
			**DJ694 + DJ1077 + DJ1080**	
DJ694;1077,1080	Bg2	07-06-15	5.72	**41.59**
DJ694;1077,1080	Bg2	07-06-15	7.24	**52.65**
DJ694;1077,1080	Bg2	07-06-15	7.38	**53.66**
DJ694;1077,1080	Bg2	07-07-04	2.89	**78.70**
DJ694;1077,1080	Bg2	07-07-04	2.24	**60.91**
DJ694;1077,1080	Bg2	07-07-04	2.27	**61.80**
			**DJ694 + 2 copies DJ1077 + DJ1080**	
DJ694;1077	Bg2;1077,1080	07-08-03	1.07	**35.23**
DJ694;1077	Bg2;1077,1080	07-08-03	0.49	**16.35**
DJ694;1077	Bg2;1077,1080	07-08-03	0.48	**15.81**
DJ694;1077	Bg2;1077,1080	07-08-15	0.24	**20.49**
DJ694;1077	Bg2;1077,1080	07-08-15	0.82	**70.27**
DJ694;1077	Bg2;1077,1080	07-08-15	0.60	**51.01**
DJ694;1077	Bg2;1077,1080	07-07-12	0.58	**37.30**
DJ694;1077	Bg2;1077,1080	07-07-12	0.38	**24.72**
DJ694;1077	Bg2;1077,1080	07-07-12	0.57	**36.87**
DJ694;1077	Bg2;1077,1080	07-07-30	0.97	64.11
DJ694;1077	Bg2;1077,1080	07-07-30	1.19	79.02
DJ694;1077	Bg2;1077,1080	07-07-30	0.92	61.25
			**DJ694 + DJ1077 + DJ1080 + DJ1083**	
DJ694;1077	Bg2;1080,1083	07-08-03	0.61	**20.01**
DJ694;1077	Bg2;1080,1083	07-08-03	0.58	**19.11**
DJ694;1077	Bg2;1080,1083	07-08-03	0.80	**26.59**

L3 larvae

Parents		Date	Specific activity ΔmOD/min/µg protein	% DJ694 control
**Male**	**Female**			
DJ694;1077	Bg2;1080,1083	07-08-15	0.51	**43.82**
DJ694;1077	Bg2;1080,1083	07-08-15	0.67	**56.90**
DJ694;1077	Bg2;1080,1083	07-08-15	0.56	**47.94**
DJ694;1077	Bg2;1080,1083	07-07-30	0.59	**39.36**
DJ694;1077	Bg2;1080,1083	07-07-30	0.54	**35.62**
DJ694;1077	Bg2;1080,1083	07-07-30	0.72	**47.54**
			**DJ694 + DJ1077 + 2 copies DJ1080**	
DJ694;1080	Bg2;1077,1080	07-08-03	0.95	**31.33**
DJ694;1080	Bg2;1077,1080	07-08-03	0.37	**12.34**
DJ694;1080	Bg2;1077,1080	07-08-03	0.72	**23.85**
DJ694;1080	Bg2;1077,1080	07-08-15	0.22	**18.64**
DJ694;1080	Bg2;1077,1080	07-08-15	0.26	**22.10**
DJ694;1080	Bg2;1077,1080	07-08-15	0.28	**23.54**
DJ694;1080	Bg2;1077,1080	07-07-12	0.66	**42.43**
DJ694;1080	Bg2;1077,1080	07-07-12	0.70	**45.14**
DJ694;1080	Bg2;1077,1080	07-07-12	0.67	**43.04**
DJ694;1080	Bg2;1077,1080	07-07-30	0.94	**62.32**
DJ694;1080	Bg2;1077,1080	07-07-30	0.73	**48.51**
DJ694;1080	Bg2;1077,1080	07-07-30	0.60	**39.58**
			**DJ694 + 2 copies DJ1080 + DJ1083**	
DJ694;1080	Bg2;1080,1083	07-08-03	0.98	**32.35**
DJ694;1080	Bg2;1080,1083	07-08-03	1.02	**33.84**
DJ694;1080	Bg2;1080,1083	07-08-03	0.95	**31.45**
DJ694;1080	Bg2;1080,1083	07-08-15	1.44	122.79
DJ694;1080	Bg2;1080,1083	07-08-15	0.93	79.54
DJ694;1080	Bg2;1080,1083	07-08-15	0.96	82.02
DJ694;1080	Bg2;1080,1083	07-07-30	0.82	**54.31**
DJ694;1080	Bg2;1080,1083	07-07-30	0.59	**38.93**
DJ694;1080	Bg2;1080,1083	07-07-30	0.83	**55.33**
			**DJ694 + 2 copies DJ1077 + DJ1079 + DJ1080**	
DJ694;1077,1079	Bg2;1077,1080	07-06-15	2.07	**15.04**
DJ694;1077,1079	Bg2;1077,1080	07-06-15	1.84	**13.38**
DJ694;1077,1079	Bg2;1077,1080	07-06-15	2.27	**16.53**
DJ694;1077,1079	Bg2;1077,1080	07-07-04	0.91	**24.68**
DJ694;1077,1079	Bg2;1077,1080	07-07-04	0.46	**12.49**
DJ694;1077,1079	Bg2;1077,1080	07-07-04	0.78	**21.24**
			**DJ694 + DJ1077 + DJ1079 + DJ1080 + DJ1083**	
DJ694;1077,1079	Bg2;1080,1083	07-07-04	1.33	**36.11**
DJ694;1077,1079	Bg2;1080,1083	07-07-04	0.90	**24.60**

L3 larvae

Parents		Date	Specific activity ΔmOD/min/µg protein	% DJ694 control
**Male**	**Female**			
DJ694;1077,1079	Bg2;1080,1083	07-07-04	0.68	**18.46**
DJ694;1077,1079	Bg2;1080,1083	07-07-12	0.66	**42.60**
DJ694;1077,1079	Bg2;1080,1083	07-07-12	0.55	**35.12**
DJ694;1077,1079	Bg2;1080,1083	07-07-12	0.44	**28.11**
			**DJ694 + 2 copies DJ1077 + 2 copies DJ1080**	
DJ694;1077,1080	Bg2;1077,1080	07-06-15	3.33	**24.20**
DJ694;1077,1080	Bg2;1077,1080	07-06-15	2.14	**15.59**
DJ694;1077,1080	Bg2;1077,1080	07-06-15	1.87	**13.57**
DJ694;1077,1080	Bg2;1077,1080	07-05-31	0.80	**24.55**
DJ694;1077,1080	Bg2;1077,1080	07-05-31	1.00	**30.58**
DJ694;1077,1080	Bg2;1077,1080	07-05-31	0.76	**23.45**
DJ694;1077,1080	Bg2;1077,1080	07-05-31	0.82	**25.25**
DJ694;1077,1080	Bg2;1077,1080	07-05-31	0.53	**16.42**
DJ694;1077,1080	Bg2;1077,1080	08-02-10	1.82	**34.88**
DJ694;1077,1080	Bg2;1077,1080	08-02-10	1.19	**22.82**
DJ694;1077,1080	Bg2;1077,1080	08-02-10	2.14	**40.93**
DJ694;1077,1080	Bg2;1077,1080	07-07-04	1.36	**36.92**
DJ694;1077,1080	Bg2;1077,1080	07-07-04	0.83	**22.67**
DJ694;1077,1080	Bg2;1077,1080	07-07-04	1.34	**36.61**
			**DJ694 + 2.1**	
DJ694;2.1	Bg2	07-06-15	14.15	102.93
DJ694;2.1	Bg2	07-06-15	10.12	73.57
DJ694;2.1	Bg2	07-06-15	10.19	74.13
DJ694;2.1	Bg2	07-05-31	2.09	**64.24**
DJ694;2.1	Bg2	07-05-31	1.93	**59.28**
DJ694;2.1	Bg2	07-05-31	1.22	**37.62**
DJ694;2.1	Bg2	07-05-31	2.84	**87.24**
DJ694;2.1	Bg2	07-05-31	2.75	**84.64**
DJ694;2.1	Bg2	07-07-04	4.13	112.38
DJ694;2.1	Bg2	07-07-04	2.96	80.61
DJ694;2.1	Bg2	07-07-04	3.33	90.68
			**DJ694 + 3.3**	
DJ694;3.3	Bg2	07-06-15	6.88	**50.01**
DJ694;3.3	Bg2	07-06-15	6.38	**46.40**
DJ694;3.3	Bg2	07-06-15	7.77	**56.50**
DJ694;3.3	Bg2	07-05-31	1.94	**59.51**
DJ694;3.3	Bg2	07-05-31	2.00	**61.48**
DJ694;3.3	Bg2	07-05-31	2.72	**83.64**
DJ694;3.3	Bg2	07-05-31	1.99	**61.27**

L3 larvae

Parents		Date	Specific activity ΔmOD/min/µg protein	% DJ694 control
**Male**	**Female**			
DJ694;3.3	Bg2	07-05-31	1.88	**57.92**
DJ694;3.3	Bg2	07-07-04	3.51	95.59
DJ694;3.3	Bg2	07-07-04	2.47	67.41
DJ694;3.3	Bg2	07-07-04	2.13	58.01
			**DJ694 + 2.1 + 3.3**	
DJ694	Bg2;2.1,3.3	07-07-04	3.58	97.63
DJ694	Bg2;2.1,3.3	07-07-04	2.32	63.11
DJ694	Bg2;2.1,3.3	07-07-12	1.10	70.43
DJ694	Bg2;2.1,3.3	07-07-12	1.42	91.17
DJ694	Bg2;2.1,3.3	07-07-04	4.27	116.40
DJ694	Bg2;2.1,3.3	07-07-12	2.18	140.36
			**DJ694 + 2 copies 2.1 + 3.3**	
DJ694;2.1	Bg2;2.1,3.3	07-08-03	2.25	74.34
DJ694;2.1	Bg2;2.1,3.3	07-08-03	1.42	46.85
DJ694;2.1	Bg2;2.1,3.3	07-08-03	2.44	80.51
DJ694;2.1	Bg2;2.1,3.3	07-08-15	1.35	115.17
DJ694;2.1	Bg2;2.1,3.3	07-08-15	1.26	107.81
DJ694;2.1	Bg2;2.1,3.3	07-08-15	0.70	59.88
DJ694;2.1	Bg2;2.1,3.3	07-07-12	1.71	110.19
DJ694;2.1	Bg2;2.1,3.3	07-07-12	1.31	83.96
DJ694;2.1	Bg2;2.1,3.3	07-07-12	1.44	92.84
DJ694;2.1	Bg2;2.1,3.3	07-07-30	2.55	**169.56**
DJ694;2.1	Bg2;2.1,3.3	07-07-30	3.27	**216.85**
			**DJ694 + 2.1 + 2 copies 3.3**	
DJ694;3.3	Bg2;2.1,3.3	07-08-15	0.78	66.65
DJ694;3.3	Bg2;2.1,3.3	07-08-15	1.19	101.27
DJ694;3.3	Bg2;2.1,3.3	07-08-15	0.62	52.70
DJ694;3.3	Bg2;2.1,3.3	07-07-12	1.15	73.77
DJ694;3.3	Bg2;2.1,3.3	07-07-12	1.36	87.19
DJ694;3.3	Bg2;2.1,3.3	07-07-12	1.18	76.10
DJ694;3.3	Bg2;2.1,3.3	07-07-30	0.90	59.59
DJ694;3.3	Bg2;2.1,3.3	07-07-30	0.91	60.50
DJ694;3.3	Bg2;2.1,3.3	07-07-30	0.92	60.80
			**DJ694 + 2.1 + DJ1077**	
DJ694	Bg2;2.1,1077	07-11-13	0.70	**24.65**
DJ694	Bg2;2.1,1077	07-11-13	0.80	**28.17**
DJ694	Bg2;2.1,1077	07-11-13	0.73	**25.65**
DJ694	Bg2;2.1,1077	07-11-30	0.70	**29.17**
DJ694	Bg2;2.1,1077	07-11-30	0.74	**30.87**

L3 larvae

Parents		Date	Specific activity ΔmOD/min/µg protein	% DJ694 control
**Male**	**Female**			
DJ694	Bg2;2.1,1077	07-11-30	0.90	**37.79**
DJ694;2.1,1077	Bg2	08-02-22	2.31	**26.70**
DJ694;2.1,1077	Bg2	08-02-22	1.41	**16.28**
DJ694;2.1,1077	Bg2	08-02-22	2.24	**25.86**
			**DJ694 + 2.1 + DJ1079**	
DJ694	Bg2;2.1,1079(13)	07-11-13	0.74	**26.04**
DJ694	Bg2;2.1,1079(13)	07-11-13	1.30	**45.55**
DJ694	Bg2;2.1,1079(13)	07-11-13	0.86	**30.29**
DJ694	Bg2;2.1,1079(13)	07-11-30	0.59	**24.82**
DJ694	Bg2;2.1,1079(13)	07-11-30	0.61	**25.43**
DJ694	Bg2;2.1,1079(13)	07-11-30	0.66	**27.66**
DJ694	Bg2;2.1,1079(14)	07-11-13	1.42	**49.73**
DJ694	Bg2;2.1,1079(14)	07-11-13	1.07	**37.73**
DJ694	Bg2;2.1,1079(14)	07-11-13	0.90	**31.45**
DJ694	Bg2;2.1,1079(14)	07-11-30	1.28	**53.50**
DJ694	Bg2;2.1,1079(14)	07-11-30	0.84	**35.30**
DJ694	Bg2;2.1,1079(14)	07-11-30	0.78	**32.90**
DJ694;2.1,1079(13)	Bg2	08-02-22	2.65	**30.58**
DJ694;2.1,1079(13)	Bg2	08-02-22	2.20	**25.46**
DJ694;2.1,1079(13)	Bg2	08-02-22	2.11	**24.40**
			**DJ694 + 3.3 + DJ1077**	
DJ694	Bg2;3.3,1077	07-11-13	0.52	**18.38**
DJ694	Bg2;3.3,1077	07-11-13	0.42	**14.93**
DJ694	Bg2;3.3,1077	07-11-13	0.86	**30.18**
DJ694	Bg2;3.3,1077	07-11-30	0.50	**21.11**
DJ694	Bg2;3.3,1077	07-11-30	0.59	**24.52**
DJ694	Bg2;3.3,1077	07-11-30	0.58	**24.45**
DJ694;3.3,1077	Bg2	08-02-22	1.83	**21.10**
DJ694;3.3,1077	Bg2	08-02-22	2.26	**26.15**
DJ694;3.3,1077	Bg2	08-02-22	3.93	**45.36**
			**DJ694 + 3.3 + DJ1079**	
DJ694	Bg2;3.3,1079	07-11-13	0.90	**31.54**
DJ694	Bg2;3.3,1079	07-11-13	1.56	**54.63**
DJ694	Bg2;3.3,1079	07-11-13	1.46	**51.43**
DJ694	Bg2;3.3,1079	07-11-30	0.93	**39.15**
DJ694	Bg2;3.3,1079	07-11-30	1.11	**46.44**
DJ694	Bg2;3.3,1079	07-11-30	0.72	**30.10**
			**DJ694 + 2.1 + DJ1077 + DJ1080**	
DJ694;2.1	Bg2;1077,1080	07-08-03	0.54	**17.89**

L3 larvae

Parents		Date	Specific activity ΔmOD/min/µg protein	% DJ694 control
**Male**	**Female**			
DJ694;2.1	Bg2;1077,1080	07-08-03	0.55	**18.09**
DJ694;2.1	Bg2;1077,1080	07-08-03	0.46	**15.11**
DJ694;2.1	Bg2;1077,1080	07-08-15	0.73	**62.06**
DJ694;2.1	Bg2;1077,1080	07-08-15	0.41	**34.80**
DJ694;2.1	Bg2;1077,1080	07-08-15	0.44	**37.17**
DJ694;2.1	Bg2;1077,1080	07-07-12	1.73	111.04
DJ694;2.1	Bg2;1077,1080	07-07-12	1.92	123.59
DJ694;2.1	Bg2;1077,1080	07-07-12	1.91	123.04
DJ694;2.1	Bg2;1077,1080	07-07-30	0.96	64.05
DJ694;2.1	Bg2;1077,1080	07-07-30	0.91	60.44
DJ694;2.1	Bg2;1077,1080	07-07-30	1.16	76.93
			**DJ694 + 2.1 + DJ1080 + DJ1083**	
DJ694;2.1	Bg2;1080,1083	07-08-03	0.68	**22.41**
DJ694;2.1	Bg2;1080,1083	07-08-03	0.73	**24.13**
DJ694;2.1	Bg2;1080,1083	07-08-03	0.92	**30.38**
DJ694;2.1	Bg2;1080,1083	07-08-15	0.55	**47.33**
DJ694;2.1	Bg2;1080,1083	07-08-15	0.39	**33.09**
DJ694;2.1	Bg2;1080,1083	07-08-15	0.51	**43.91**
DJ694;2.1	Bg2;1080,1083	07-07-12	0.75	**47.92**
DJ694;2.1	Bg2;1080,1083	07-07-12	0.92	**59.12**
DJ694;2.1	Bg2;1080,1083	07-07-12	0.94	**60.47**
DJ694;2.1	Bg2;1080,1083	07-07-30	0.79	**52.40**
DJ694;2.1	Bg2;1080,1083	07-07-30	0.86	**57.12**
DJ694;2.1	Bg2;1080,1083	07-07-30	0.67	**44.72**
			**DJ694 + 3.3 + DJ1077 + DJ1080**	
DJ694;3.3	Bg2;1077,1080	07-08-03	0.69	**22.89**
DJ694;3.3	Bg2;1077,1080	07-08-03	0.94	**30.98**
DJ694;3.3	Bg2;1077,1080	07-08-03	1.10	**36.47**
DJ694;3.3	Bg2;1077,1080	07-08-15	0.60	**51.48**
DJ694;3.3	Bg2;1077,1080	07-08-15	0.53	**45.46**
DJ694;3.3	Bg2;1077,1080	07-08-15	0.41	**35.01**
DJ694;3.3	Bg2;1077,1080	07-07-12	0.51	**32.54**
DJ694;3.3	Bg2;1077,1080	07-07-12	0.46	**29.42**
DJ694;3.3	Bg2;1077,1080	07-07-12	0.36	**23.28**
DJ694;3.3	Bg2;1077,1080	07-07-30	0.45	29.59
DJ694;3.3	Bg2;1077,1080	07-07-30	0.40	26.40
DJ694;3.3	Bg2;1077,1080	07-07-30	1.04	69.30
			**DJ694 + 3.3 + DJ1080 + DJ1083**	
DJ694;3.3	Bg2;1080,1083	07-08-03	1.16	**38.48**

L3 larvae

Parents		Date	Specific activity ΔmOD/min/µg protein	% DJ694 control
Male	Female			
DJ694;3.3	Bg2;1080,1083	07-08-03	1.09	**36.15**
DJ694;3.3	Bg2;1080,1083	07-08-03	0.58	**19.28**
DJ694;3.3	Bg2;1080,1083	07-08-15	0.42	**35.89**
DJ694;3.3	Bg2;1080,1083	07-08-15	0.59	**50.16**
DJ694;3.3	Bg2;1080,1083	07-08-15	0.55	**46.77**
DJ694;3.3	Bg2;1080,1083	07-07-12	0.17	**11.17**
DJ694;3.3	Bg2;1080,1083	07-07-12	0.51	**33.07**
DJ694;3.3	Bg2;1080,1083	07-07-12	0.49	**31.30**
DJ694;3.3	Bg2;1080,1083	07-07-30	0.65	**43.15**
DJ694;3.3	Bg2;1080,1083	07-07-30	0.71	**47.37**
DJ694;3.3	Bg2;1080,1083	07-07-30	0.76	**50.14**
			**DJ694 + 2.1 + 3.3 + DJ1077**	
DJ694;1077	Bg2;2.1,3.3	07-08-03	0.58	**19.19**
DJ694;1077	Bg2;2.1,3.3	07-08-03	0.43	**14.17**
DJ694;1077	Bg2;2.1,3.3	07-08-03	0.69	**22.78**
DJ694;1077	Bg2;2.1,3.3	07-08-15	0.30	**25.55**
DJ694;1077	Bg2;2.1,3.3	07-08-15	0.47	**39.78**
DJ694;1077	Bg2;2.1,3.3	07-08-15	0.32	**27.10**
DJ694;1077	Bg2;2.1,3.3	07-07-04	0.88	**24.08**
DJ694;1077	Bg2;2.1,3.3	07-07-04	1.32	**35.92**
DJ694;1077	Bg2;2.1,3.3	07-07-04	0.79	**21.58**
DJ694;1077	Bg2;2.1,3.3	07-07-12	0.33	**21.25**
DJ694;1077	Bg2;2.1,3.3	07-07-12	0.31	**19.83**
DJ694;1077	Bg2;2.1,3.3	07-07-12	0.29	**18.70**
DJ694;1077	Bg2;2.1,3.3	07-07-30	0.75	**50.01**
DJ694;1077	Bg2;2.1,3.3	07-07-30	0.75	**50.05**
DJ694;1077	Bg2;2.1,3.3	07-07-30	0.76	**50.16**
			**DJ694 + 2.1 + 3.3 + DJ1080**	
DJ694;1080	Bg2;2.1,3.3	07-08-03	1.14	**37.68**
DJ694;1080	Bg2;2.1,3.3	07-08-03	0.78	**25.86**
DJ694;1080	Bg2;2.1,3.3	07-08-03	0.67	**22.23**
DJ694;1080	Bg2;2.1,3.3	07-08-15	0.76	**65.10**
DJ694;1080	Bg2;2.1,3.3	07-08-15	0.65	**55.63**
DJ694;1080	Bg2;2.1,3.3	07-08-15	0.55	**46.54**
DJ694;1080	Bg2;2.1,3.3	07-07-04	2.06	**56.14**
DJ694;1080	Bg2;2.1,3.3	07-07-04	1.49	**40.62**
DJ694;1080	Bg2;2.1,3.3	07-07-04	1.62	**44.04**
DJ694;1080	Bg2;2.1,3.3	07-07-12	0.73	**46.94**
DJ694;1080	Bg2;2.1,3.3	07-07-12	0.62	**39.81**
DJ694;1080	Bg2;2.1,3.3	07-07-12	0.58	**37.20**

L3 larvae

Parents		Date	Specific activity ΔmOD/min/μg protein	% DJ694 control
**Male**	**Female**			
DJ694;1080	Bg2;2.1,3.3	07-07-30	1.81	120.29
DJ694;1080	Bg2;2.1,3.3	07-07-30	0.95	62.97
DJ694;1080	Bg2;2.1,3.3	07-07-30	0.61	40.39
			**DJ694 + 2 copies 2.1 + 2 copies DJ1077**	
DJ694;2.1,1077	Bg2;2.1,1077	07-11-13	0.28	**9.85**
DJ694;2.1,1077	Bg2;2.1,1077	07-11-13	0.29	**10.29**
DJ694;2.1,1077	Bg2;2.1,1077	07-11-13	0.41	**14.26**
DJ694;2.1,1077	Bg2;2.1,1077	07-11-30	0.45	**18.97**
DJ694;2.1,1077	Bg2;2.1,1077	07-11-30	0.44	**18.45**
DJ694;2.1,1077	Bg2;2.1,1077	07-11-30	0.65	**27.23**
DJ694;2.1,1077	Bg2;2.1,1077	07-12-19	1.39	**35.29**
DJ694;2.1,1077	Bg2;2.1,1077	07-12-19	1.30	**33.06**
DJ694;2.1,1077	Bg2;2.1,1077	07-12-19	1.04	**26.41**
DJ694;2.1,1077	Bg2;2.1,1077	07-12-19	1.72	**43.84**
DJ694;2.1,1077	Bg2;2.1,1077	07-12-19	1.09	**27.76**
DJ694;2.1,1077	Bg2;2.1,1077	07-12-19	1.64	**41.73**
DJ694;2.1,1077	Bg2;2.1,1077	08-02-10	0.66	**12.72**
DJ694;2.1,1077	Bg2;2.1,1077	08-02-10	0.89	**16.98**
DJ694;2.1,1077	Bg2;2.1,1077	08-02-10	1.07	**20.45**
DJ694;2.1,1077	Bg2;2.1,1077	08-02-22	1.26	**14.61**
DJ694;2.1,1077	Bg2;2.1,1077	08-02-22	1.63	**18.87**
DJ694;2.1,1077	Bg2;2.1,1077	08-02-22	2.00	**23.15**
			**DJ694 + 2 copies 2.1 + DJ1077 + DJ1079**	
DJ694;2.1,1077	Bg2;2.1,1079(13)	07-11-13	0.39	**13.72**
DJ694;2.1,1077	Bg2;2.1,1079(13)	07-11-13	0.60	**21.10**
DJ694;2.1,1077	Bg2;2.1,1079(13)	07-11-13	0.56	**19.58**
DJ694;2.1,1077	Bg2;2.1,1079(13)	07-11-30	0.53	**22.41**
DJ694;2.1,1077	Bg2;2.1,1079(13)	07-11-30	0.43	**17.87**
DJ694;2.1,1077	Bg2;2.1,1079(13)	07-11-30	0.45	**18.68**
DJ694;2.1,1077	Bg2;2.1,1079(13)	07-12-19	1.05	**26.61**
DJ694;2.1,1077	Bg2;2.1,1079(13)	07-12-19	1.02	**26.00**
DJ694;2.1,1077	Bg2;2.1,1079(13)	07-12-19	0.96	**24.30**
DJ694;2.1,1077	Bg2;2.1,1079(14)	07-12-19	1.65	**41.86**
DJ694;2.1,1077	Bg2;2.1,1079(14)	07-12-19	1.87	**47.66**
DJ694;2.1,1077	Bg2;2.1,1079(14)	07-12-19	2.12	**53.86**
DJ694;2.1,1077	Bg2;2.1,1079(14)	07-11-13	0.39	**13.82**
DJ694;2.1,1077	Bg2;2.1,1079(14)	07-11-13	0.29	**10.32**
DJ694;2.1,1077	Bg2;2.1,1079(14)	07-11-13	0.33	**11.62**
DJ694;2.1,1077	Bg2;2.1,1079(14)	07-11-30	0.67	**28.26**
DJ694;2.1,1077	Bg2;2.1,1079(14)	07-11-30	0.63	**26.22**

L3 larvae

Parents		Date	Specific activity ΔmOD/min/μg protein	% DJ694 control
Male	Female			
DJ694;2.1,1077	Bg2;2.1,1079(14)	07-11-30	0.45	**18.84**
DJ694;2.1,1077	Bg2;2.1,1079(14)	07-12-19	1.25	**31.72**
DJ694;2.1,1077	Bg2;2.1,1079(14)	07-12-19	1.35	**34.23**
DJ694;2.1,1077	Bg2;2.1,1079(14)	07-12-19	1.55	**39.49**
DJ694;2.1,1079(13)	Bg2;2.1,1077	07-12-19	0.54	**13.61**
DJ694;2.1,1079(13)	Bg2;2.1,1077	07-12-19	1.49	**37.77**
DJ694;2.1,1079(13)	Bg2;2.1,1077	07-12-19	1.01	**25.70**
DJ694;2.1,1079(14)	Bg2;2.1,1077	07-12-19	1.46	**37.18**
DJ694;2.1,1079(14)	Bg2;2.1,1077	07-12-19	1.17	**29.76**
DJ694;2.1,1079(14)	Bg2;2.1,1077	07-12-19	1.01	**25.55**
DJ694;2.1,1079(14)	Bg2;2.1,1077	07-12-19	0.63	**15.92**
DJ694;2.1,1079(14)	Bg2;2.1,1077	07-12-19	1.03	**26.06**
DJ694;2.1,1079(14)	Bg2;2.1,1077	07-12-19	1.25	**31.73**
DJ694;2.1,1077	Bg2;2.1,1079(13)	08-02-10	0.75	**14.26**
DJ694;2.1,1077	Bg2;2.1,1079(13)	08-02-10	0.54	**10.38**
DJ694;2.1,1077	Bg2;2.1,1079(13)	08-02-10	0.70	**13.49**
DJ694;2.1,1077	Bg2;2.1,1079(13)	08-02-22	1.65	**19.07**
DJ694;2.1,1077	Bg2;2.1,1079(13)	08-02-22	1.42	**16.44**
DJ694;2.1,1077	Bg2;2.1,1079(13)	08-02-22	1.38	**16.00**
DJ694;2.1,1077	Bg2;2.1,1079(14)	08-02-10	0.48	**9.10**
DJ694;2.1,1077	Bg2;2.1,1079(14)	08-02-10	0.78	**14.97**
DJ694;2.1,1077	Bg2;2.1,1079(14)	08-02-10	0.61	**11.58**
DJ694;2.1,1079(13)	Bg2;2.1,1077	08-02-10	0.46	**8.86**
DJ694;2.1,1079(13)	Bg2;2.1,1077	08-02-10	0.56	**10.67**
DJ694;2.1,1079(13)	Bg2;2.1,1077	08-02-10	0.66	**12.67**
DJ694;2.1,1079(13)	Bg2;2.1,1077	08-02-22	3.60	**41.65**
DJ694;2.1,1079(13)	Bg2;2.1,1077	08-02-22	2.29	**26.47**
DJ694;2.1,1079(13)	Bg2;2.1,1077	08-02-22	4.44	**51.31**
DJ694;2.1,1079(14)	Bg2;2.1,1077	08-02-10	0.86	**16.42**
DJ694;2.1,1079(14)	Bg2;2.1,1077	08-02-10	0.90	**17.23**
DJ694;2.1,1079(14)	Bg2;2.1,1077	08-02-10	0.83	**15.92**
			**DJ694 + 2.1 + 3.3 + 2 copies DJ1077**	
DJ694;2.1,1077	Bg2;3.3,1077	07-12-19	1.24	**31.58**
DJ694;2.1,1077	Bg2;3.3,1077	07-12-19	1.19	**30.35**
DJ694;2.1,1077	Bg2;3.3,1077	07-12-19	0.49	**12.38**
DJ694;2.1,1077	Bg2;3.3,1077	07-11-13	0.41	**14.46**
DJ694;2.1,1077	Bg2;3.3,1077	07-11-13	0.38	**13.42**
DJ694;2.1,1077	Bg2;3.3,1077	07-11-13	0.41	**14.30**
DJ694;2.1,1077	Bg2;3.3,1077	07-11-30	0.48	**20.23**
DJ694;2.1,1077	Bg2;3.3,1077	07-11-30	0.28	**11.61**

L3 larvae

Parents		Date	Specific activity ΔmOD/min/μg protein	% DJ694 control
**Male**	**Female**			
DJ694;2.1,1077	Bg2;3.3,1077	07-11-30	0.44	**18.56**
DJ694;2.1,1077	Bg2;3.3,1077	07-12-19	1.20	**30.52**
DJ694;2.1,1077	Bg2;3.3,1077	07-12-19	0.93	**23.62**
DJ694;2.1,1077	Bg2;3.3,1077	07-12-19	1.33	**33.89**
DJ694;3.3,1077	Bg2;2.1,1077	07-11-13	0.51	**17.95**
DJ694;3.3,1077	Bg2;2.1,1077	07-11-13	0.47	**16.34**
DJ694;3.3,1077	Bg2;2.1,1077	07-11-13	0.62	**21.88**
DJ694;3.3,1077	Bg2;2.1,1077	07-11-30	0.37	**15.67**
DJ694;3.3,1077	Bg2;2.1,1077	07-11-30	0.57	**23.85**
DJ694;3.3,1077	Bg2;2.1,1077	07-11-30	0.47	**19.62**
DJ694;3.3,1077	Bg2;2.1,1077	07-12-19	0.99	**25.17**
DJ694;3.3,1077	Bg2;2.1,1077	07-12-19	1.01	**25.66**
DJ694;3.3,1077	Bg2;2.1,1077	07-12-19	1.44	**36.52**
DJ694;3.3,1077	Bg2;2.1,1077	07-12-19	0.83	**21.00**
DJ694;3.3,1077	Bg2;2.1,1077	07-12-19	1.45	**36.96**
DJ694;3.3,1077	Bg2;2.1,1077	07-12-19	0.94	**23.99**
DJ694;2.1,1077	Bg2;3.3,1077	08-02-10	0.71	**13.65**
DJ694;2.1,1077	Bg2;3.3,1077	08-02-10	0.71	**13.56**
DJ694;2.1,1077	Bg2;3.3,1077	08-02-10	0.64	**12.22**
DJ694;2.1,1077	Bg2;3.3,1077	08-02-22	1.90	**21.98**
DJ694;2.1,1077	Bg2;3.3,1077	08-02-22	1.96	**22.66**
DJ694;2.1,1077	Bg2;3.3,1077	08-02-22	1.42	**16.36**
DJ694;3.3,1077	Bg2;2.1,1077	08-02-10	0.76	**14.60**
DJ694;3.3,1077	Bg2;2.1,1077	08-02-10	1.09	**20.88**
DJ694;3.3,1077	Bg2;2.1,1077	08-02-10	1.01	**19.28**
DJ694;3.3,1077	Bg2;2.1,1077	08-02-22	0.96	**11.06**
DJ694;3.3,1077	Bg2;2.1,1077	08-02-22	1.05	**12.14**
DJ694;3.3,1077	Bg2;2.1,1077	08-02-22	0.91	**10.51**
			**DJ694 + 2.1 + 3.3 + DJ1077 + DJ1079**	
DJ694;2.1,1077	Bg2;3.3,1079	07-11-13	0.37	**13.03**
DJ694;2.1,1077	Bg2;3.3,1079	07-11-13	0.39	**13.65**
DJ694;2.1,1077	Bg2;3.3,1079	07-11-13	0.65	**22.74**
DJ694;2.1,1077	Bg2;3.3,1079	07-11-30	0.44	**18.47**
DJ694;2.1,1077	Bg2;3.3,1079	07-11-30	0.41	**17.10**
DJ694;2.1,1077	Bg2;3.3,1079	07-11-30	0.62	**26.06**
DJ694;2.1,1077	Bg2;3.3,1079	07-12-19	1.20	**30.61**
DJ694;2.1,1077	Bg2;3.3,1079	07-12-19	1.55	**39.47**
DJ694;2.1,1077	Bg2;3.3,1079	07-12-19	1.25	**31.76**
DJ694;2.1,1077	Bg2;3.3,1079	07-12-19	1.10	**28.03**
DJ694;2.1,1077	Bg2;3.3,1079	07-12-19	1.72	**43.70**

L3 larvae

Parents		Date	Specific activity ΔmOD/min/μg protein	% DJ694 control
Male	Female			
DJ694;2.1,1077	Bg2;3.3,1079	07-12-19	1.85	**47.16**
DJ694;2.1,1079(13)	Bg2;3.3,1077	07-12-19	0.81	**20.60**
DJ694;2.1,1079(13)	Bg2;3.3,1077	07-12-19	0.72	**18.29**
DJ694;2.1,1079(13)	Bg2;3.3,1077	07-12-19	0.88	**22.44**
DJ694;2.1,1079(14)	Bg2;3.3,1077	07-12-19	0.88	**22.47**
DJ694;2.1,1079(14)	Bg2;3.3,1077	07-12-19	0.91	**23.21**
DJ694;2.1,1079(14)	Bg2;3.3,1077	07-12-19	0.73	**18.44**
DJ694;2.1,1079(14)	Bg2;3.3,1077	07-12-19	0.99	**25.28**
DJ694;2.1,1079(14)	Bg2;3.3,1077	07-12-19	0.99	**25.23**
DJ694;2.1,1079(14)	Bg2;3.3,1077	07-12-19	1.02	**25.88**
DJ694;3.3,1077	Bg2;2.1,1079(13)	07-11-13	0.36	**12.60**
DJ694;3.3,1077	Bg2;2.1,1079(13)	07-11-13	0.19	**6.84**
DJ694;3.3,1077	Bg2;2.1,1079(13)	07-11-13	0.59	**20.69**
DJ694;3.3,1077	Bg2;2.1,1079(13)	07-11-30	0.38	**15.91**
DJ694;3.3,1077	Bg2;2.1,1079(13)	07-11-30	0.42	**17.77**
DJ694;3.3,1077	Bg2;2.1,1079(13)	07-11-30	0.46	**19.37**
DJ694;3.3,1077	Bg2;2.1,1079(13)	07-12-19	0.87	**22.05**
DJ694;3.3,1077	Bg2;2.1,1079(13)	07-12-19	0.79	**19.96**
DJ694;3.3,1077	Bg2;2.1,1079(13)	07-12-19	0.91	**23.20**
DJ694;3.3,1077	Bg2;2.1,1079(14)	07-11-13	0.48	**16.78**
DJ694;3.3,1077	Bg2;2.1,1079(14)	07-11-13	0.35	**12.27**
DJ694;3.3,1077	Bg2;2.1,1079(14)	07-11-13	0.25	**8.81**
DJ694;3.3,1077	Bg2;2.1,1079(14)	07-11-30	0.38	**15.87**
DJ694;3.3,1077	Bg2;2.1,1079(14)	07-11-30	0.41	**17.08**
DJ694;3.3,1077	Bg2;2.1,1079(14)	07-11-30	0.39	**16.42**
DJ694;3.3,1077	Bg2;2.1,1079(14)	07-12-19	1.36	**34.63**
DJ694;3.3,1077	Bg2;2.1,1079(14)	07-12-19	0.72	**18.40**
DJ694;3.3,1077	Bg2;2.1,1079(14)	07-12-19	0.74	**18.73**
DJ694;3.3,1077	Bg2;2.1,1079(14)	07-12-19	1.10	**27.87**
DJ694;3.3,1077	Bg2;2.1,1079(14)	07-12-19	1.11	**28.20**
DJ694;3.3,1077	Bg2;2.1,1079(14)	07-12-19	1.11	**28.30**
DJ694;1077,1079	Bg2;2.1,3.3	07-06-15	1.44	**10.50**
DJ694;1077,1079	Bg2;2.1,3.3	07-06-15	1.45	**10.52**
DJ694;1077,1079	Bg2;2.1,3.3	07-06-15	1.34	**9.78**
DJ694;1077,1079	Bg2;2.1,3.3	07-07-04	0.89	**24.20**
DJ694;1077,1079	Bg2;2.1,3.3	07-07-04	0.88	**23.87**
DJ694;1077,1079	Bg2;2.1,3.3	07-07-04	0.80	**21.82**
DJ694;2.1,1077	Bg2;3.3,1079	08-02-10	0.96	**18.43**
DJ694;2.1,1077	Bg2;3.3,1079	08-02-10	1.25	**23.99**
DJ694;2.1,1077	Bg2;3.3,1079	08-02-10	1.00	**19.11**

L3 larvae

Parents		Date	Specific activity ΔmOD/min/µg protein	% DJ694 control
**Male**	**Female**			
DJ694;2.1,1079(13)	Bg2;3.3,1077	08-02-10	0.73	**13.92**
DJ694;2.1,1079(13)	Bg2;3.3,1077	08-02-10	0.82	**15.63**
DJ694;2.1,1079(13)	Bg2;3.3,1077	08-02-10	0.57	**10.83**
DJ694;2.1,1079(13)	Bg2;3.3,1077	08-02-22	2.17	**25.08**
DJ694;2.1,1079(13)	Bg2;3.3,1077	08-02-22	2.87	**33.18**
DJ694;2.1,1079(14)	Bg2;3.3,1077	08-02-10	1.32	**25.20**
DJ694;2.1,1079(14)	Bg2;3.3,1077	08-02-10	1.39	**26.54**
DJ694;2.1,1079(14)	Bg2;3.3,1077	08-02-10	1.62	**31.05**
DJ694;3.3,1077	Bg2;2.1,1079(13)	08-02-10	1.11	**21.19**
DJ694;3.3,1077	Bg2;2.1,1079(13)	08-02-10	0.97	**18.56**
DJ694;3.3,1077	Bg2;2.1,1079(13)	08-02-10	0.82	**15.70**
DJ694;3.3,1077	Bg2;2.1,1079(13)	08-02-22	1.98	**22.84**
DJ694;3.3,1077	Bg2;2.1,1079(13)	08-02-22	2.55	**29.47**
DJ694;3.3,1077	Bg2;2.1,1079(13)	08-02-22	2.95	**34.10**
DJ694;3.3,1077	Bg2;2.1,1079(14)	08-02-10	0.74	**14.13**
DJ694;3.3,1077	Bg2;2.1,1079(14)	08-02-10	0.69	**13.24**
DJ694;3.3,1077	Bg2;2.1,1079(14)	08-02-10	1.12	**21.36**
DJ694;2.1,1079(13)	Bg2;3.3,1077	08-02-22	7.98	92.26
			**DJ694 + 2 copies 2.1 + 2 copies DJ1079**	
DJ694;2.1,1079(13)	Bg2;2.1,1079(13)	07-12-19	1.13	**28.63**
DJ694;2.1,1079(13)	Bg2;2.1,1079(13)	07-12-19	0.81	**20.53**
DJ694;2.1,1079(13)	Bg2;2.1,1079(13)	07-12-19	0.89	**22.55**
DJ694;2.1,1079(13)	Bg2;2.1,1079(14)	07-12-19	0.72	**18.33**
DJ694;2.1,1079(13)	Bg2;2.1,1079(14)	07-12-19	0.88	**22.38**
DJ694;2.1,1079(13)	Bg2;2.1,1079(14)	07-12-19	0.79	**20.16**
DJ694;2.1,1079(14)	Bg2;2.1,1079(13)	07-12-19	1.23	**31.17**
DJ694;2.1,1079(14)	Bg2;2.1,1079(13)	07-12-19	1.39	**35.34**
DJ694;2.1,1079(14)	Bg2;2.1,1079(13)	07-12-19	1.09	**27.64**
DJ694;2.1,1079(14)	Bg2;2.1,1079(14)	07-12-19	1.37	**34.84**
DJ694;2.1,1079(14)	Bg2;2.1,1079(14)	07-12-19	0.98	**24.86**
DJ694;2.1,1079(14)	Bg2;2.1,1079(14)	07-12-19	1.61	**40.87**
DJ694;2.1,1079(14)	Bg2;2.1,1079(14)	07-12-19	1.03	**26.12**
DJ694;2.1,1079(14)	Bg2;2.1,1079(14)	07-12-19	1.62	**41.28**
DJ694;2.1,1079(14)	Bg2;2.1,1079(14)	07-12-19	1.60	**40.60**
DJ694;2.1,1079(13)	Bg2;2.1,1079(13)	08-02-10	0.73	**13.93**
DJ694;2.1,1079(13)	Bg2;2.1,1079(13)	08-02-10	0.96	**18.36**
DJ694;2.1,1079(13)	Bg2;2.1,1079(13)	08-02-10	0.79	**15.18**
DJ694;2.1,1079(13)	Bg2;2.1,1079(14)	08-02-10	0.62	**11.93**
DJ694;2.1,1079(13)	Bg2;2.1,1079(14)	08-02-10	0.77	**14.77**
DJ694;2.1,1079(13)	Bg2;2.1,1079(14)	08-02-10	0.83	**15.91**

L3 larvae

Parents		Date	Specific activity ΔmOD/min/µg protein	% DJ694 control
Male	Female			
DJ694;2.1,1079(14)	Bg2;2.1,1079(13)	08-02-10	0.69	**13.14**
DJ694;2.1,1079(14)	Bg2;2.1,1079(13)	08-02-10	0.62	**11.81**
DJ694;2.1,1079(14)	Bg2;2.1,1079(13)	08-02-10	0.63	**12.00**
DJ694;2.1,1079(14)	Bg2;2.1,1079(14)	08-02-10	0.87	**16.58**
DJ694;2.1,1079(14)	Bg2;2.1,1079(14)	08-02-10	0.91	**17.37**
DJ694;2.1,1079(14)	Bg2;2.1,1079(14)	08-02-10	1.09	**20.90**
			**DJ694 + 2.1 + 3.3 + 2 copies DJ1079**	
DJ694;2.1,1079(13)	Bg2;3.3,1079	07-12-19	1.11	**28.32**
DJ694;2.1,1079(13)	Bg2;3.3,1079	07-12-19	1.30	**33.03**
DJ694;2.1,1079(13)	Bg2;3.3,1079	07-12-19	1.10	**28.04**
DJ694;2.1,1079(14)	Bg2;3.3,1079	07-12-19	0.91	**23.16**
DJ694;2.1,1079(14)	Bg2;3.3,1079	07-12-19	0.91	**23.19**
DJ694;2.1,1079(14)	Bg2;3.3,1079	07-12-19	1.29	**32.84**
DJ694;2.1,1079(14)	Bg2;3.3,1079	07-12-19	1.03	**26.26**
DJ694;2.1,1079(14)	Bg2;3.3,1079	07-12-19	0.88	**22.25**
DJ694;2.1,1079(14)	Bg2;3.3,1079	07-12-19	1.55	**39.37**
DJ694;2.1,1079(13)	Bg2;3.3,1079	08-02-10	0.74	**14.13**
DJ694;2.1,1079(13)	Bg2;3.3,1079	08-02-10	0.80	**15.28**
DJ694;2.1,1079(14)	Bg2;3.3,1079	08-02-10	0.07	**1.41**
DJ694;2.1,1079(14)	Bg2;3.3,1079	08-02-10	1.05	**20.08**
DJ694;2.1,1079(14)	Bg2;3.3,1079	08-02-10	1.36	**26.06**
			**DJ694 + 2 copies 3.3 + 2 copies DJ1077**	
DJ694;3.3,1077	Bg2;3.3,1077	07-11-13	0.51	**17.84**
DJ694;3.3,1077	Bg2;3.3,1077	07-11-13	0.37	**12.89**
DJ694;3.3,1077	Bg2;3.3,1077	07-11-13	0.54	**18.92**
DJ694;3.3,1077	Bg2;3.3,1077	07-11-30	0.53	**22.31**
DJ694;3.3,1077	Bg2;3.3,1077	07-11-30	0.57	**23.82**
DJ694;3.3,1077	Bg2;3.3,1077	07-11-30	0.67	**28.21**
DJ694;3.3,1077	Bg2;3.3,1077	07-12-19	0.81	**20.71**
DJ694;3.3,1077	Bg2;3.3,1077	07-12-19	0.84	**21.42**
DJ694;3.3,1077	Bg2;3.3,1077	07-12-19	0.78	**19.93**
DJ694;3.3,1077	Bg2;3.3,1077	07-12-19	1.20	**30.47**
DJ694;3.3,1077	Bg2;3.3,1077	07-12-19	1.07	**27.16**
DJ694;3.3,1077	Bg2;3.3,1077	07-12-19	0.96	**24.32**
DJ694;3.3,1077	Bg2;3.3,1077	08-02-10	0.65	**12.45**
DJ694;3.3,1077	Bg2;3.3,1077	08-02-10	0.80	**15.36**
DJ694;3.3,1077	Bg2;3.3,1077	08-02-10	0.79	**15.03**
DJ694;3.3,1077	Bg2;3.3,1077	08-02-22	1.74	**20.10**
DJ694;3.3,1077	Bg2;3.3,1077	08-02-22	1.49	**17.17**
DJ694;3.3,1077	Bg2;3.3,1077	08-02-22	1.59	**18.33**

L3 larvae

Parents		Date	Specific activity ΔmOD/min/µg protein	% DJ694 control
Male	Female			
			**DJ694 + 2 copies 3.3 + DJ1077 + DJ1079**	
DJ694;3.3,1077	Bg2;3.3,1079	07-11-13	0.36	**12.76**
DJ694;3.3,1077	Bg2;3.3,1079	07-11-13	0.54	**19.09**
DJ694;3.3,1077	Bg2;3.3,1079	07-11-13	0.34	**12.00**
DJ694;3.3,1077	Bg2;3.3,1079	07-11-30	0.40	**16.81**
DJ694;3.3,1077	Bg2;3.3,1079	07-11-30	0.32	**13.55**
DJ694;3.3,1077	Bg2;3.3,1079	07-11-30	0.48	**20.25**
DJ694;3.3,1077	Bg2;3.3,1079	07-12-19	1.38	**35.00**
DJ694;3.3,1077	Bg2;3.3,1079	07-12-19	1.74	**44.29**
DJ694;3.3,1077	Bg2;3.3,1079	07-12-19	1.42	**36.09**
DJ694;3.3,1077	Bg2;3.3,1079	07-12-19	0.97	**24.67**
DJ694;3.3,1077	Bg2;3.3,1079	07-12-19	1.47	**37.32**
DJ694;3.3,1077	Bg2;3.3,1079	07-12-19	0.84	**21.30**
DJ694;3.3,1077	Bg2;3.3,1079	08-02-10	0.48	**9.14**
DJ694;3.3,1077	Bg2;3.3,1079	08-02-10	0.90	**17.12**
DJ694;3.3,1077	Bg2;3.3,1079	08-02-10	0.89	**17.06**
			**DJ694 + 2.1 + 3.3 + DJ1077 + DJ1080**	
DJ694;1077,1080	Bg2;2.1,3.3	07-06-15	2.08	**15.14**
DJ694;1077,1080	Bg2;2.1,3.3	07-06-15	1.48	**10.80**
DJ694;1077,1080	Bg2;2.1,3.3	07-06-15	1.57	**11.42**
DJ694;1077,1080	Bg2;2.1,3.3	07-07-04	0.89	**24.13**
DJ694;1077,1080	Bg2;2.1,3.3	07-07-04	1.31	**35.73**
DJ694;1077,1080	Bg2;2.1,3.3	07-07-04	1.16	**31.59**
DJ694;2.1,3.3	Bg2;1077,1080	07-05-31	0.30	**9.24**
DJ694;2.1,3.3	Bg2;1077,1080	07-05-31	0.41	**12.47**
DJ694;2.1,3.3	Bg2;1077,1080	07-05-31	0.12	**3.75**
DJ694;2.1,3.3	Bg2;1077,1080	07-05-31	0.29	**8.77**
DJ694;2.1,3.3	Bg2;1077,1080	07-05-31	0.10	**3.06**
			**DJ694 + 2.1 + 3.3 + DJ1080 + DJ1083**	
DJ694;1080,1083	Bg2;2.1,3.3	07-05-31	0.84	**25.90**
DJ694;1080,1083	Bg2;2.1,3.3	07-05-31	1.42	**43.59**
DJ694;1080,1083	Bg2;2.1,3.3	07-05-31	0.92	**28.19**
			**DJ694 + 2.1 + 2 copies DJ1077 + DJ1079**	
DJ694;1077,1079	Bg2;2.1,1077	07-12-19	1.22	**30.94**
DJ694;1077,1079	Bg2;2.1,1077	07-12-19	1.09	**27.71**
DJ694;1077,1079	Bg2;2.1,1077	07-12-19	1.09	**27.64**
			**DJ694 + 2.1 + DJ1077 + 2 copies DJ1079**	
DJ694;1077,1079	Bg2;2.1,1079(14)	07-12-19	1.00	**25.36**
DJ694;1077,1079	Bg2;2.1,1079(14)	07-12-19	0.82	**20.87**

L3 larvae

Parents		Date	Specific activity ΔmOD/min/µg protein	% DJ694 control
Male	Female			
DJ694;1077,1079	Bg2;2.1,1079(14)	07-12-19	0.83	**21.16**
			**DJ694 + 3.3 + 2 copies DJ1077 + DJ1079**	
DJ694;1077,1079	Bg2;3.3,1077	07-12-19	1.19	**30.19**
DJ694;1077,1079	Bg2;3.3,1077	07-12-19	0.92	**23.48**
DJ694;1077,1079	Bg2;3.3,1077	07-12-19	1.18	**29.99**
			**DJ694 + 3.3 + DJ1077 + 2 copies DJ1079**	
DJ694;1077,1079	Bg2;3.3,1079	07-12-19	1.20	**30.51**
DJ694;1077,1079	Bg2;3.3,1079	07-12-19	1.04	**26.55**
DJ694;1077,1079	Bg2;3.3,1079	07-12-19	1.32	**33.62**
			**DJ694 + 2.1 + 2 copies DJ1077 + DJ1080**	
DJ694;1077,1080	Bg2;2.1,1077	08-02-10	0.79	**15.19**
DJ694;1077,1080	Bg2;2.1,1077	08-02-10	0.74	**14.07**
DJ694;1077,1080	Bg2;2.1,1077	08-02-10	0.67	**12.80**
DJ694;2.1,1077	Bg2;1077,1080	08-02-10	1.12	**21.45**
DJ694;2.1,1077	Bg2;1077,1080	08-02-10	0.86	**16.49**
DJ694;2.1,1077	Bg2;1077,1080	08-02-10	0.72	**13.78**
			**DJ694 + 2.1 + DJ1077 + DJ1079 + DJ1080**	
DJ694;1077,1080	Bg2;2.1,1079(13)	08-02-10	0.51	**9.70**
DJ694;1077,1080	Bg2;2.1,1079(13)	08-02-10	1.22	**23.43**
DJ694;1077,1080	Bg2;2.1,1079(13)	08-02-10	0.68	**12.96**
DJ694;1077,1080	Bg2;2.1,1079(14)	08-02-10	0.88	**16.79**
DJ694;1077,1080	Bg2;2.1,1079(14)	08-02-10	0.68	**12.96**
DJ694;1077,1080	Bg2;2.1,1079(14)	08-02-10	0.74	**14.23**
DJ694;2.1,1079(13)	Bg2;1077,1080	08-02-10	0.32	**6.13**
DJ694;2.1,1079(13)	Bg2;1077,1080	08-02-10	0.18	**3.49**
DJ694;2.1,1079(14)	Bg2;1077,1080	08-02-10	0.99	**18.88**
DJ694;2.1,1079(14)	Bg2;1077,1080	08-02-10	0.86	**16.47**
DJ694;2.1,1079(14)	Bg2;1077,1080	08-02-10	1.09	**20.77**
			**DJ694 + 3.3 + 2 copies DJ1077 + DJ1080**	
DJ694;1077,1080	Bg2;3.3,1077	08-02-10	0.74	**14.21**
DJ694;1077,1080	Bg2;3.3,1077	08-02-10	0.77	**14.79**
DJ694;1077,1080	Bg2;3.3,1077	08-02-10	0.84	**15.98**
DJ694;3.3,1077	Bg2;1077,1080	08-02-10	0.65	**12.51**
DJ694;3.3,1077	Bg2;1077,1080	08-02-10	0.66	**12.69**
DJ694;3.3,1077	Bg2;1077,1080	08-02-10	0.69	**13.24**
			**DJ694 + 3.3 + DJ1077 + DJ1079 + DJ1080**	
DJ694;1077,1080	Bg2;3.3,1079	08-02-10	1.05	**20.06**
DJ694;1077,1080	Bg2;3.3,1079	08-02-10	1.52	**29.10**
DJ694;1077,1080	Bg2;3.3,1079	08-02-10	0.93	**17.73**

L3 larvae

Parents		Date	Specific activity ΔmOD/min/µg protein	% DJ694 control
**Male**	**Female**			
			**DJ694 + 2.1 + DJ1077 + DJ1080 + DJ1083**	
DJ694;2.1,1077	Bg2;1080,1083	08-02-10	0.69	**13.16**
DJ694;2.1,1077	Bg2;1080,1083	08-02-10	0.76	**14.56**
DJ694;2.1,1077	Bg2;1080,1083	08-02-10	0.76	**14.63**
			**DJ694 + 2.1 + DJ1079 + DJ1080 + DJ1083**	
DJ694;2.1,1079(13)	Bg2;1080,1083	08-02-10	0.54	**10.27**
DJ694;2.1,1079(13)	Bg2;1080,1083	08-02-10	0.62	**11.88**
DJ694;2.1,1079(13)	Bg2;1080,1083	08-02-10	0.57	**10.86**
DJ694;2.1,1079(14)	Bg2;1080,1083	08-02-10	0.85	**16.24**
DJ694;2.1,1079(14)	Bg2;1080,1083	08-02-10	1.24	**23.76**
DJ694;2.1,1079(14)	Bg2;1080,1083	08-02-10	1.33	**25.39**
			**DJ694 + 3.3 + DJ1077 + DJ1080 + DJ1083**	
DJ694;3.3,1077	Bg2;1080,1083	08-02-10	0.58	**11.06**
DJ694;3.3,1077	Bg2;1080,1083	08-02-10	0.63	**12.11**
DJ694;3.3,1077	Bg2;1080,1083	08-02-10	0.55	**10.50**

Supplemental Table 6: UAS-lacZ assay EP data set.

% DJ694 control: specific activity normalized to the positive control, bolded values are significantly different ($p<0.05$) from the positive control.

Early pupae

Parents		Date	Specific activity ΔmOD/min/µg protein	% DJ694 control
Male	Female			
			**DJ694 + DJ1077**	
DJ694;1077	Bg2	15-Jun-07	4.13	**49.47**
DJ694;1077	Bg2	15-Jun-07	3.99	**47.72**
DJ694;1077	Bg2	15-Jun-07	4.09	**48.93**
DJ694;1077	Bg2	04-Jul-07	1.47	**65.16**
DJ694;1077	Bg2	04-Jul-07	1.30	**57.32**
DJ694;1077	Bg2	04-Jul-07	1.32	**58.44**
DJ694;1077	Bg2	31-May-07	3.09	**62.66**
DJ694;1077	Bg2	31-May-07	2.68	**54.49**
DJ694;1077	Bg2	31-May-07	3.52	**71.39**
DJ694;1077	Bg2	31-May-07	3.75	**76.06**
DJ694;1077	Bg2	31-May-07	2.36	**47.85**
			**DJ694 + DJ1080**	
DJ694;1080	Bg2	15-Jun-07	6.68	79.95
DJ694;1080	Bg2	15-Jun-07	8.13	97.34
DJ694;1080	Bg2	15-Jun-07	6.74	80.65
DJ694;1080	Bg2	31-May-07	4.31	87.39
DJ694;1080	Bg2	31-May-07	4.80	97.39
DJ694;1080	Bg2	31-May-07	3.56	72.30
DJ694;1080	Bg2	31-May-07	4.18	84.94
DJ694;1080	Bg2	31-May-07	2.50	50.79
DJ694;1080	Bg2	04-Jul-07	1.97	87.11
DJ694;1080	Bg2	04-Jul-07	2.08	91.88
DJ694;1080	Bg2	04-Jul-07	2.13	94.06
			**DJ694 + DJ1077 + DJ1080**	
DJ694	Bg2;1077,1080	03-Aug-07	1.33	**46.34**
DJ694	Bg2;1077,1080	03-Aug-07	1.90	**66.34**
DJ694	Bg2;1077,1080	03-Aug-07	1.77	**61.95**
DJ694	Bg2;1077,1080	15-Aug-07	1.21	86.84
DJ694	Bg2;1077,1080	15-Aug-07	0.97	69.63
DJ694	Bg2;1077,1080	15-Aug-07	1.15	82.01
DJ694	Bg2;1077,1080	30-Jul-07	1.20	**67.26**
DJ694	Bg2;1077,1080	30-Jul-07	0.91	**51.23**
DJ694	Bg2;1077,1080	30-Jul-07	0.68	**38.22**
DJ694;1077,1080	Bg2	15-Jun-07	5.01	**59.93**
DJ694;1077,1080	Bg2	15-Jun-07	5.67	**67.82**
DJ694;1077,1080	Bg2	15-Jun-07	4.09	**48.94**
DJ694;1077,1080	Bg2	04-Jul-07	0.94	**41.50**
DJ694;1077,1080	Bg2	04-Jul-07	1.39	**61.35**

Early pupae

Parents		Date	Specific activity ΔmOD/min/µg protein	% DJ694 control
**Male**	**Female**			
DJ694;1077,1080	Bg2	04-Jul-07	0.85	**37.46**
			**DJ694 + DJ1080 + DJ1083**	
DJ694	Bg2;1080,1083	15-Aug-07	2.15	154.06
DJ694	Bg2;1080,1083	15-Aug-07	1.59	113.71
DJ694	Bg2;1080,1083	15-Aug-07	1.40	100.32
DJ694	Bg2;1080,1083	15-Jun-07	8.60	102.95
DJ694	Bg2;1080,1083	15-Jun-07	6.73	80.57
DJ694	Bg2;1080,1083	15-Jun-07	6.82	81.62
DJ694	Bg2;1080,1083	04-Jul-07	1.14	50.55
DJ694	Bg2;1080,1083	04-Jul-07	1.81	79.91
DJ694	Bg2;1080,1083	04-Jul-07	1.46	64.58
DJ694	Bg2;1080,1083	12-Jul-07	1.28	47.88
DJ694	Bg2;1080,1083	12-Jul-07	1.85	69.16
DJ694	Bg2;1080,1083	12-Jul-07	2.37	88.66
			**DJ694 + DJ1077 + DJ1079**	
DJ694;1077,1079	Bg2	15-Aug-07	0.63	44.98
DJ694;1077,1079	Bg2	15-Aug-07	0.69	49.51
DJ694;1077,1079	Bg2	15-Aug-07	0.90	64.28
DJ694;1077,1079	Bg2	15-Jun-07	3.24	**38.83**
DJ694;1077,1079	Bg2	15-Jun-07	3.24	**38.78**
DJ694;1077,1079	Bg2	15-Jun-07	3.87	**46.34**
DJ694;1077,1079	Bg2	04-Jul-07	1.08	**47.55**
DJ694;1077,1079	Bg2	04-Jul-07	1.18	**52.31**
DJ694;1077,1079	Bg2	04-Jul-07	1.05	**46.56**
			**DJ694 + 2 copies DJ1077 + DJ1080**	
DJ694;1077	Bg2;1077,1080	03-Aug-07	0.41	**14.46**
DJ694;1077	Bg2;1077,1080	03-Aug-07	0.79	**27.61**
DJ694;1077	Bg2;1077,1080	03-Aug-07	0.60	**20.95**
DJ694;1077	Bg2;1077,1080	12-Jul-07	0.34	**12.87**
DJ694;1077	Bg2;1077,1080	12-Jul-07	0.28	**10.42**
DJ694;1077	Bg2;1077,1080	12-Jul-07	0.33	**12.30**
DJ694;1077	Bg2;1077,1080	30-Jul-07	0.33	**18.33**
DJ694;1077	Bg2;1077,1080	30-Jul-07	0.64	**35.89**
DJ694;1077	Bg2;1077,1080	30-Jul-07	0.30	**16.54**
			**DJ694 + DJ1077 + DJ1080 + DJ1083**	
DJ694;1077	Bg2;1080,1083	03-Aug-07	0.74	**25.82**
DJ694;1077	Bg2;1080,1083	03-Aug-07	0.57	**20.00**
DJ694;1077	Bg2;1080,1083	03-Aug-07	0.78	**27.25**
DJ694;1077	Bg2;1080,1083	15-Aug-07	0.42	29.99

Early pupae

Parents		Date	Specific activity ΔmOD/min/μg protein	% DJ694 control
**Male**	**Female**			
DJ694;1077	Bg2;1080,1083	15-Aug-07	0.67	47.98
DJ694;1077	Bg2;1080,1083	15-Aug-07	0.52	37.53
DJ694;1077	Bg2;1080,1083	30-Jul-07	0.41	**22.70**
DJ694;1077	Bg2;1080,1083	30-Jul-07	0.63	**35.48**
DJ694;1077	Bg2;1080,1083	30-Jul-07	0.61	**34.23**
			**DJ694 + DJ1077 + 2 copies DJ1080**	
DJ694;1080	Bg2;1077,1080	03-Aug-07	0.75	**26.27**
DJ694;1080	Bg2;1077,1080	03-Aug-07	0.77	**26.83**
DJ694;1080	Bg2;1077,1080	03-Aug-07	0.88	**30.58**
DJ694;1080	Bg2;1077,1080	15-Aug-07	0.42	29.97
DJ694;1080	Bg2;1077,1080	15-Aug-07	0.45	32.07
DJ694;1080	Bg2;1077,1080	15-Aug-07	0.40	28.95
DJ694;1080	Bg2;1077,1080	12-Jul-07	0.47	**17.67**
DJ694;1080	Bg2;1077,1080	12-Jul-07	0.35	**13.05**
DJ694;1080	Bg2;1077,1080	12-Jul-07	0.46	**17.37**
DJ694;1080	Bg2;1077,1080	30-Jul-07	0.36	**20.13**
DJ694;1080	Bg2;1077,1080	30-Jul-07	0.39	**21.74**
DJ694;1080	Bg2;1077,1080	30-Jul-07	0.32	**17.97**
			**DJ694 + 2 copies DJ1080 + DJ1083**	
DJ694;1080	Bg2;1080,1083	03-Aug-07	1.33	**46.40**
DJ694;1080	Bg2;1080,1083	03-Aug-07	0.95	**33.05**
DJ694;1080	Bg2;1080,1083	03-Aug-07	1.14	**39.80**
DJ694;1080	Bg2;1080,1083	15-Aug-07	0.64	45.65
DJ694;1080	Bg2;1080,1083	15-Aug-07	0.71	50.74
DJ694;1080	Bg2;1080,1083	15-Aug-07	0.72	51.30
DJ694;1080	Bg2;1080,1083	30-Jul-07	0.64	**36.02**
DJ694;1080	Bg2;1080,1083	30-Jul-07	0.74	**41.28**
DJ694;1080	Bg2;1080,1083	30-Jul-07	0.65	**36.21**
			**DJ694 + 2 copies DJ1077 + DJ1079 + DJ1080**	
DJ694;1077,1079	Bg2;1077,1080	15-Jun-07	0.70	**8.39**
DJ694;1077,1079	Bg2;1077,1080	15-Jun-07	0.91	**10.93**
DJ694;1077,1079	Bg2;1077,1080	15-Jun-07	0.72	**8.58**
DJ694;1077,1079	Bg2;1077,1080	04-Jul-07	0.39	**17.39**
DJ694;1077,1079	Bg2;1077,1080	04-Jul-07	0.45	**20.08**
DJ694;1077,1079	Bg2;1077,1080	04-Jul-07	0.47	**20.98**
			**DJ694 + DJ1077 + DJ1079 + DJ1080 + DJ1083**	
DJ694;1077,1079	Bg2;1080,1083	04-Jul-07	0.70	**30.71**
DJ694;1077,1079	Bg2;1080,1083	04-Jul-07	0.62	**27.28**
DJ694;1077,1079	Bg2;1080,1083	04-Jul-07	0.50	**22.17**

Early pupae

Parents		Date	Specific activity ΔmOD/min/µg protein	% DJ694 control
Male	Female			
DJ694;1077,1079	Bg2;1080,1083	12-Jul-07	0.53	**19.72**
DJ694;1077,1079	Bg2;1080,1083	12-Jul-07	0.45	**16.99**
DJ694;1077,1079	Bg2;1080,1083	12-Jul-07	0.50	**18.58**
			**DJ694 + 2 copies DJ1077 + 2 copies DJ1080**	
DJ694;1077,1080	Bg2;1077,1080	15-Jun-07	1.34	**16.02**
DJ694;1077,1080	Bg2;1077,1080	15-Jun-07	1.41	**16.89**
DJ694;1077,1080	Bg2;1077,1080	15-Jun-07	0.68	**8.15**
DJ694;1077,1080	Bg2;1077,1080	31-May-07	0.75	**15.22**
DJ694;1077,1080	Bg2;1077,1080	31-May-07	0.89	**18.06**
DJ694;1077,1080	Bg2;1077,1080	31-May-07	0.62	**12.65**
DJ694;1077,1080	Bg2;1077,1080	31-May-07	0.62	**12.63**
DJ694;1077,1080	Bg2;1077,1080	10-Feb-08	0.58	**14.38**
DJ694;1077,1080	Bg2;1077,1080	10-Feb-08	1.09	**26.99**
DJ694;1077,1080	Bg2;1077,1080	10-Feb-08	0.83	**20.71**
DJ694;1077,1080	Bg2;1077,1080	04-Jul-07	0.56	**24.85**
DJ694;1077,1080	Bg2;1077,1080	04-Jul-07	0.71	**31.38**
DJ694;1077,1080	Bg2;1077,1080	04-Jul-07	0.39	**17.20**
			**DJ694 + DJ1077 + 2 copies DJ1080 + DJ1083**	
DJ694;1077,1080	Bg2;1080,1083	10-Feb-08	1.84	45.65
DJ694;1077,1080	Bg2;1080,1083	10-Feb-08	1.79	44.32
DJ694;1077,1080	Bg2;1080,1083	10-Feb-08	3.10	77.06
			**DJ694 + 2.1**	
DJ694;2.1	Bg2	15-Jun-07	6.97	83.38
DJ694;2.1	Bg2	15-Jun-07	7.69	92.10
DJ694;2.1	Bg2	15-Jun-07	7.53	90.17
DJ694;2.1	Bg2	31-May-07	4.74	96.29
DJ694;2.1	Bg2	31-May-07	4.69	95.18
DJ694;2.1	Bg2	31-May-07	4.87	98.93
DJ694;2.1	Bg2	31-May-07	3.25	65.91
DJ694;2.1	Bg2	31-May-07	4.74	96.30
DJ694;2.1	Bg2	04-Jul-07	2.56	113.29
DJ694;2.1	Bg2	04-Jul-07	2.42	107.08
DJ694;2.1	Bg2	04-Jul-07	2.70	119.21
			**DJ694 + 3.3**	
DJ694;3.3	Bg2	15-Jun-07	6.92	82.87
DJ694;3.3	Bg2	15-Jun-07	8.71	104.26
DJ694;3.3	Bg2	15-Jun-07	7.65	91.62
DJ694;3.3	Bg2	31-May-07	4.23	85.85
DJ694;3.3	Bg2	31-May-07	4.24	86.06

Early pupae

Parents		Date	Specific activity ΔmOD/min/μg protein	% DJ694 control
**Male**	**Female**			
DJ694;3.3	Bg2	31-May-07	4.32	87.60
DJ694;3.3	Bg2	31-May-07	3.55	72.05
DJ694;3.3	Bg2	31-May-07	3.75	76.13
DJ694;3.3	Bg2	04-Jul-07	1.83	81.05
DJ694;3.3	Bg2	04-Jul-07	1.94	85.75
DJ694;3.3	Bg2	04-Jul-07	2.14	94.62
			**DJ694 + 2.1 + 3.3**	
DJ694	Bg2;2.1,3.3	04-Jul-07	1.36	60.30
DJ694	Bg2;2.1,3.3	04-Jul-07	1.61	70.97
DJ694	Bg2;2.1,3.3	04-Jul-07	2.44	107.96
DJ694	Bg2;2.1,3.3	12-Jul-07	1.68	**62.84**
DJ694	Bg2;2.1,3.3	12-Jul-07	1.41	**52.81**
DJ694	Bg2;2.1,3.3	12-Jul-07	1.33	**49.76**
			**DJ694 + 2 copies 2.1 + 3.3**	
DJ694;2.1	Bg2;2.1,3.3	03-Aug-07	1.81	**63.01**
DJ694;2.1	Bg2;2.1,3.3	03-Aug-07	1.80	**62.73**
DJ694;2.1	Bg2;2.1,3.3	03-Aug-07	1.80	**62.66**
DJ694;2.1	Bg2;2.1,3.3	15-Aug-07	1.02	73.14
DJ694;2.1	Bg2;2.1,3.3	15-Aug-07	1.75	125.27
DJ694;2.1	Bg2;2.1,3.3	15-Aug-07	1.53	109.88
DJ694;2.1	Bg2;2.1,3.3	12-Jul-07	1.75	**65.64**
DJ694;2.1	Bg2;2.1,3.3	12-Jul-07	1.72	**64.39**
DJ694;2.1	Bg2;2.1,3.3	12-Jul-07	1.71	**64.07**
DJ694;2.1	Bg2;2.1,3.3	30-Jul-07	1.69	94.50
DJ694;2.1	Bg2;2.1,3.3	30-Jul-07	1.99	111.71
DJ694;2.1	Bg2;2.1,3.3	30-Jul-07	0.72	40.41
			**DJ694 + 2.1 + 2 copies 3.3**	
DJ694;3.3	Bg2;2.1,3.3	15-Aug-07	1.52	108.56
DJ694;3.3	Bg2;2.1,3.3	15-Aug-07	1.09	78.21
DJ694;3.3	Bg2;2.1,3.3	15-Aug-07	0.82	58.45
DJ694;3.3	Bg2;2.1,3.3	12-Jul-07	1.24	**46.36**
DJ694;3.3	Bg2;2.1,3.3	12-Jul-07	1.88	**70.26**
DJ694;3.3	Bg2;2.1,3.3	12-Jul-07	1.42	**53.08**
DJ694;3.3	Bg2;2.1,3.3	30-Jul-07	0.74	**41.27**
DJ694;3.3	Bg2;2.1,3.3	30-Jul-07	0.54	**30.00**
DJ694;3.3	Bg2;2.1,3.3	30-Jul-07	0.74	**41.70**
			**DJ694 + 2.1 + DJ1077**	
DJ694	Bg2;2.1,1077	13-Nov-07	0.91	**35.82**
DJ694	Bg2;2.1,1077	13-Nov-07	0.42	**16.56**

Early pupae

Parents		Date	Specific activity ΔmOD/min/µg protein	% DJ694 control
**Male**	**Female**			
DJ694	Bg2;2.1,1077	13-Nov-07	0.69	**27.14**
DJ694	Bg2;2.1,1077	30-Nov-07	1.15	51.52
DJ694	Bg2;2.1,1077	30-Nov-07	0.49	21.82
DJ694	Bg2;2.1,1077	30-Nov-07	1.02	45.51
DJ694;2.1,1077	Bg2	22-Feb-08	4.32	44.47
DJ694;2.1,1077	Bg2	22-Feb-08	1.92	19.79
DJ694;2.1,1077	Bg2	22-Feb-08	7.19	74.00
			**DJ694 + 2.1 + DJ1079**	
DJ694	Bg2;2.1,1079(13)	13-Nov-07	0.29	**11.36**
DJ694	Bg2;2.1,1079(13)	13-Nov-07	0.60	**23.65**
DJ694	Bg2;2.1,1079(13)	13-Nov-07	0.39	**15.55**
DJ694	Bg2;2.1,1079(13)	30-Nov-07	0.15	**6.54**
DJ694	Bg2;2.1,1079(13)	30-Nov-07	0.39	**17.40**
DJ694	Bg2;2.1,1079(13)	30-Nov-07	0.27	**11.94**
DJ694	Bg2;2.1,1079(14)	13-Nov-07	1.62	**63.93**
DJ694	Bg2;2.1,1079(14)	13-Nov-07	1.78	**70.12**
DJ694	Bg2;2.1,1079(14)	13-Nov-07	1.29	**50.84**
DJ694	Bg2;2.1,1079(14)	30-Nov-07	0.78	**34.84**
DJ694	Bg2;2.1,1079(14)	30-Nov-07	0.77	**34.58**
DJ694	Bg2;2.1,1079(14)	30-Nov-07	1.48	**66.29**
DJ694;2.1,1079(13)	Bg2	22-Feb-08	2.27	**23.42**
DJ694;2.1,1079(13)	Bg2	22-Feb-08	2.43	**25.01**
DJ694;2.1,1079(13)	Bg2	22-Feb-08	2.00	**20.56**
			**DJ694 + 3.3 + DJ1077**	
DJ694	Bg;3.3,1077	13-Nov-07	0.28	**11.13**
DJ694	Bg;3.3,1077	13-Nov-07	0.33	**13.19**
DJ694	Bg;3.3,1077	13-Nov-07	0.65	**25.65**
DJ694	Bg;3.3,1077	30-Nov-07	0.59	26.50
DJ694	Bg;3.3,1077	30-Nov-07	0.60	26.81
DJ694	Bg;3.3,1077	30-Nov-07	0.30	13.51
DJ694;3.3,1077	Bg2	22-Feb-08	4.13	**42.57**
DJ694;3.3,1077	Bg2	22-Feb-08	2.20	**22.66**
DJ694;3.3,1077	Bg2	22-Feb-08	5.42	**55.84**
			**DJ694 + 3.3 + DJ1079**	
DJ694	Bg2;3.3,1079	13-Nov-07	1.45	**57.08**
DJ694	Bg2;3.3,1079	13-Nov-07	1.81	**71.46**
DJ694	Bg2;3.3,1079	13-Nov-07	1.50	**59.04**
DJ694	Bg2;3.3,1079	30-Nov-07	0.54	24.35
DJ694	Bg2;3.3,1079	30-Nov-07	1.47	65.65

Early pupae

Parents		Date	Specific activity ΔmOD/min/µg protein	% DJ694 control
**Male**	**Female**			
DJ694	Bg2;3.3,1079	30-Nov-07	1.04	46.58
			**DJ694 + 2.1 + DJ1077 + DJ1080**	
DJ694;2.1	Bg2;1077+1080	03-Aug-07	0.30	**10.49**
DJ694;2.1	Bg2;1077+1080	03-Aug-07	0.25	**8.75**
DJ694;2.1	Bg2;1077+1080	03-Aug-07	0.30	**10.47**
DJ694;2.1	Bg2;1077+1080	15-Aug-07	0.27	19.65
DJ694;2.1	Bg2;1077+1080	15-Aug-07	0.17	12.29
DJ694;2.1	Bg2;1077+1080	15-Aug-07	0.23	16.33
DJ694;2.1	Bg2;1077+1080	12-Jul-07	2.03	**75.90**
DJ694;2.1	Bg2;1077+1080	12-Jul-07	1.87	**69.80**
DJ694;2.1	Bg2;1077+1080	12-Jul-07	1.76	**65.97**
DJ694;2.1	Bg2;1077+1080	30-Jul-07	0.24	**13.61**
DJ694;2.1	Bg2;1077+1080	30-Jul-07	0.26	**14.33**
DJ694;2.1	Bg2;1077+1080	30-Jul-07	0.30	**16.73**
			**DJ694 + 2.1 + DJ1080 + DJ1083**	
DJ694;2.1	Bg2;1080,1083	03-Aug-07	0.76	**26.42**
DJ694;2.1	Bg2;1080,1083	03-Aug-07	0.99	**34.61**
DJ694;2.1	Bg2;1080,1083	03-Aug-07	0.73	**25.49**
DJ694;2.1	Bg2;1080,1083	15-Aug-07	0.29	20.55
DJ694;2.1	Bg2;1080,1083	15-Aug-07	0.52	37.25
DJ694;2.1	Bg2;1080,1083	15-Aug-07	0.37	26.37
DJ694;2.1	Bg2;1080,1083	12-Jul-07	0.68	**25.32**
DJ694;2.1	Bg2;1080,1083	12-Jul-07	0.77	**28.92**
DJ694;2.1	Bg2;1080,1083	12-Jul-07	0.65	**24.43**
DJ694;2.1	Bg2;1080,1083	30-Jul-07	0.77	**43.39**
DJ694;2.1	Bg2;1080,1083	30-Jul-07	0.57	**31.83**
DJ694;2.1	Bg2;1080,1083	30-Jul-07	0.72	**40.33**
			**DJ694 + 3.3 + DJ1077 + DJ1080**	
DJ694;3.3	Bg2;1077+1080	03-Aug-07	0.31	**10.81**
DJ694;3.3	Bg2;1077+1080	03-Aug-07	0.45	**15.64**
DJ694;3.3	Bg2;1077+1080	03-Aug-07	0.27	**9.28**
DJ694;3.3	Bg2;1077+1080	15-Aug-07	0.33	23.40
DJ694;3.3	Bg2;1077+1080	15-Aug-07	0.27	19.24
DJ694;3.3	Bg2;1077+1080	15-Aug-07	0.23	16.78
DJ694;3.3	Bg2;1077+1080	12-Jul-07	0.31	**11.57**
DJ694;3.3	Bg2;1077+1080	12-Jul-07	0.20	**7.50**
DJ694;3.3	Bg2;1077+1080	12-Jul-07	0.27	**10.17**
DJ694;3.3	Bg2;1077+1080	30-Jul-07	0.33	**18.54**
DJ694;3.3	Bg2;1077+1080	30-Jul-07	0.25	**14.02**

Early pupae

Parents		Date	Specific activity ΔmOD/min/µg protein	% DJ694 control
**Male**	**Female**			
DJ694;3.3	Bg2;1077+1080	30-Jul-07	0.25	**14.13**
			**DJ694 + 3.3 + DJ1080 + DJ1083**	
DJ694;3.3	Bg2;1080,1083	03-Aug-07	0.40	**14.11**
DJ694;3.3	Bg2;1080,1083	03-Aug-07	0.66	**23.09**
DJ694;3.3	Bg2;1080,1083	03-Aug-07	0.64	**22.28**
DJ694;3.3	Bg2;1080,1083	15-Aug-07	0.48	34.31
DJ694;3.3	Bg2;1080,1083	15-Aug-07	0.30	21.56
DJ694;3.3	Bg2;1080,1083	15-Aug-07	0.40	28.90
DJ694;3.3	Bg2;1080,1083	12-Jul-07	0.41	**15.28**
DJ694;3.3	Bg2;1080,1083	12-Jul-07	0.49	**18.52**
DJ694;3.3	Bg2;1080,1083	12-Jul-07	0.49	**18.43**
DJ694;3.3	Bg2;1080,1083	30-Jul-07	0.52	**28.91**
DJ694;3.3	Bg2;1080,1083	30-Jul-07	0.35	**19.67**
DJ694;3.3	Bg2;1080,1083	30-Jul-07	0.41	**23.03**
			**DJ694 + 2.1 + 3.3 + DJ1077**	
DJ694;1077	Bg2;2.1,3.3	03-Aug-07	0.60	**20.79**
DJ694;1077	Bg2;2.1,3.3	03-Aug-07	0.47	**16.38**
DJ694;1077	Bg2;2.1,3.3	03-Aug-07	0.75	**26.08**
DJ694;1077	Bg2;2.1,3.3	15-Aug-07	0.26	18.38
DJ694;1077	Bg2;2.1,3.3	15-Aug-07	0.14	9.99
DJ694;1077	Bg2;2.1,3.3	15-Aug-07	0.29	20.86
DJ694;1077	Bg2;2.1,3.3	04-Jul-07	0.54	**24.06**
DJ694;1077	Bg2;2.1,3.3	04-Jul-07	0.89	**39.28**
DJ694;1077	Bg2;2.1,3.3	04-Jul-07	0.85	**37.33**
DJ694;1077	Bg2;2.1,3.3	12-Jul-07	0.37	**13.69**
DJ694;1077	Bg2;2.1,3.3	12-Jul-07	0.34	**12.86**
DJ694;1077	Bg2;2.1,3.3	12-Jul-07	0.29	**10.69**
DJ694;1077	Bg2;2.1,3.3	30-Jul-07	0.40	**22.58**
DJ694;1077	Bg2;2.1,3.3	30-Jul-07	0.35	**19.62**
DJ694;1077	Bg2;2.1,3.3	30-Jul-07	0.46	**25.65**
			**DJ694 + 2.1 + 3.3 + DJ1080**	
DJ694;1080	Bg2;2.1,3.3	03-Aug-07	0.65	**22.81**
DJ694;1080	Bg2;2.1,3.3	03-Aug-07	0.88	**30.88**
DJ694;1080	Bg2;2.1,3.3	03-Aug-07	0.65	**22.86**
DJ694;1080	Bg2;2.1,3.3	15-Aug-07	0.78	56.00
DJ694;1080	Bg2;2.1,3.3	15-Aug-07	1.04	74.63
DJ694;1080	Bg2;2.1,3.3	15-Aug-07	0.44	31.26
DJ694;1080	Bg2;2.1,3.3	04-Jul-07	0.92	**40.63**
DJ694;1080	Bg2;2.1,3.3	04-Jul-07	0.69	**30.39**

Early pupae

Parents		Date	Specific activity ΔmOD/min/µg protein	% DJ694 control
**Male**	**Female**			
DJ694;1080	Bg2;2.1,3.3	04-Jul-07	0.67	**29.47**
DJ694;1080	Bg2;2.1,3.3	12-Jul-07	0.57	**21.45**
DJ694;1080	Bg2;2.1,3.3	12-Jul-07	0.75	**27.97**
DJ694;1080	Bg2;2.1,3.3	12-Jul-07	0.98	**36.62**
DJ694;1080	Bg2;2.1,3.3	30-Jul-07	0.73	**41.09**
DJ694;1080	Bg2;2.1,3.3	30-Jul-07	0.51	**28.63**
DJ694;1080	Bg2;2.1,3.3	30-Jul-07	0.58	**32.60**
			**DJ694 + 2 copies 2.1 + 2 copies DJ1077**	
DJ694;2.1,1077	Bg2;2.1,1077	13-Nov-07	0.16	**6.50**
DJ694;2.1,1077	Bg2;2.1,1077	13-Nov-07	0.13	**5.12**
DJ694;2.1,1077	Bg2;2.1,1077	13-Nov-07	0.16	**6.42**
DJ694;2.1,1077	Bg2;2.1,1077	30-Nov-07	0.17	**7.41**
DJ694;2.1,1077	Bg2;2.1,1077	30-Nov-07	0.17	**7.47**
DJ694;2.1,1077	Bg2;2.1,1077	30-Nov-07	0.35	**15.50**
DJ694;2.1,1077	Bg2;2.1,1077	19-Dec-07	0.35	**5.94**
DJ694;2.1,1077	Bg2;2.1,1077	19-Dec-07	0.29	**4.87**
DJ694;2.1,1077	Bg2;2.1,1077	19-Dec-07	0.39	**6.65**
DJ694;2.1,1077	Bg2;2.1,1077	19-Dec-07	0.72	**12.19**
DJ694;2.1,1077	Bg2;2.1,1077	19-Dec-07	0.90	**15.34**
DJ694;2.1,1077	Bg2;2.1,1077	19-Dec-07	0.95	**16.20**
DJ694;2.1,1077	Bg2;2.1,1077	10-Feb-08	0.17	**4.30**
DJ694;2.1,1077	Bg2;2.1,1077	10-Feb-08	0.11	**2.82**
DJ694;2.1,1077	Bg2;2.1,1077	10-Feb-08	0.13	**3.17**
DJ694;2.1,1077	Bg2;2.1,1077	22-Feb-08	0.53	**5.41**
DJ694;2.1,1077	Bg2;2.1,1077	22-Feb-08	0.39	**4.07**
DJ694;2.1,1077	Bg2;2.1,1077	22-Feb-08	0.34	**3.55**
			**DJ694 + 2 copies 2.1 + DJ1077 + DJ1079**	
DJ694;2.1,1077	Bg2;2.1,1079(13)	13-Nov-07	0.15	**5.91**
DJ694;2.1,1077	Bg2;2.1,1079(13)	13-Nov-07	0.16	**6.48**
DJ694;2.1,1077	Bg2;2.1,1079(13)	13-Nov-07	0.30	**11.66**
DJ694;2.1,1077	Bg2;2.1,1079(13)	30-Nov-07	0.32	**14.35**
DJ694;2.1,1077	Bg2;2.1,1079(13)	30-Nov-07	0.17	**7.77**
DJ694;2.1,1077	Bg2;2.1,1079(13)	30-Nov-07	0.24	**10.63**
DJ694;2.1,1077	Bg2;2.1,1079(13)	19-Dec-07	0.44	**7.56**
DJ694;2.1,1077	Bg2;2.1,1079(13)	19-Dec-07	0.50	**8.56**
DJ694;2.1,1077	Bg2;2.1,1079(13)	19-Dec-07	0.49	**8.42**
DJ694;2.1,1077	Bg2;2.1,1079(14)	13-Nov-07	0.16	**6.37**
DJ694;2.1,1077	Bg2;2.1,1079(14)	13-Nov-07	0.31	**12.42**
DJ694;2.1,1077	Bg2;2.1,1079(14)	13-Nov-07	0.34	**13.40**
DJ694;2.1,1077	Bg2;2.1,1079(14)	30-Nov-07	0.38	**17.12**

Early pupae

Parents		Date	Specific activity ΔmOD/min/µg protein	% DJ694 control
**Male**	**Female**			
DJ694;2.1,1077	Bg2;2.1,1079(14)	30-Nov-07	0.31	**13.85**
DJ694;2.1,1077	Bg2;2.1,1079(14)	30-Nov-07	0.32	**14.42**
DJ694;2.1,1077	Bg2;2.1,1079(14)	19-Dec-07	0.65	**11.09**
DJ694;2.1,1077	Bg2;2.1,1079(14)	19-Dec-07	0.43	**7.31**
DJ694;2.1,1077	Bg2;2.1,1079(14)	19-Dec-07	0.66	**11.28**
DJ694;2.1,1077	Bg2;2.1,1079(14)	19-Dec-07	3.71	**63.27**
DJ694;2.1,1077	Bg2;2.1,1079(14)	19-Dec-07	1.77	**30.17**
DJ694;2.1,1077	Bg2;2.1,1079(14)	19-Dec-07	2.18	**37.18**
DJ694;2.1,1079(13)	Bg2;2.1,1077	19-Dec-07	0.49	**8.35**
DJ694;2.1,1079(13)	Bg2;2.1,1077	19-Dec-07	0.25	**4.30**
DJ694;2.1,1079(13)	Bg2;2.1,1077	19-Dec-07	0.56	**9.57**
DJ694;2.1,1079(14)	Bg2;2.1,1077	19-Dec-07	0.32	**5.49**
DJ694;2.1,1079(14)	Bg2;2.1,1077	19-Dec-07	0.70	**11.89**
DJ694;2.1,1079(14)	Bg2;2.1,1077	19-Dec-07	0.23	**3.90**
DJ694;2.1,1079(14)	Bg2;2.1,1077	19-Dec-07	0.34	**5.76**
DJ694;2.1,1079(14)	Bg2;2.1,1077	19-Dec-07	0.34	**5.72**
DJ694;2.1,1079(14)	Bg2;2.1,1077	19-Dec-07	0.38	**6.51**
DJ694;2.1,1077	Bg2;2.1,1079(13)	10-Feb-08	0.13	**3.15**
DJ694;2.1,1077	Bg2;2.1,1079(13)	10-Feb-08	0.15	**3.68**
DJ694;2.1,1077	Bg2;2.1,1079(13)	10-Feb-08	0.10	**2.53**
DJ694;2.1,1077	Bg2;2.1,1079(13)	22-Feb-08	0.83	**8.59**
DJ694;2.1,1077	Bg2;2.1,1079(13)	22-Feb-08	0.55	**5.68**
DJ694;2.1,1077	Bg2;2.1,1079(13)	22-Feb-08	0.49	**5.05**
DJ694;2.1,1077	Bg2;2.1,1079(14)	10-Feb-08	0.17	**4.28**
DJ694;2.1,1077	Bg2;2.1,1079(14)	10-Feb-08	0.15	**3.79**
DJ694;2.1,1077	Bg2;2.1,1079(14)	10-Feb-08	0.21	**5.33**
DJ694;2.1,1079(13)	Bg2;2.1,1077	10-Feb-08	0.09	**2.22**
DJ694;2.1,1079(13)	Bg2;2.1,1077	10-Feb-08	0.13	**3.15**
DJ694;2.1,1079(13)	Bg2;2.1,1077	10-Feb-08	0.10	**2.56**
DJ694;2.1,1079(13)	Bg2;2.1,1077	22-Feb-08	0.55	**5.67**
DJ694;2.1,1079(13)	Bg2;2.1,1077	22-Feb-08	0.81	**8.38**
DJ694;2.1,1079(13)	Bg2;2.1,1077	22-Feb-08	0.57	**5.85**
DJ694;2.1,1079(14)	Bg2;2.1,1077	10-Feb-08	0.65	**16.10**
DJ694;2.1,1079(14)	Bg2;2.1,1077	10-Feb-08	1.68	**41.83**
DJ694;2.1,1079(14)	Bg2;2.1,1077	10-Feb-08	0.57	**14.25**
			**DJ694 + 2.1 + 3.3 + 2 copies DJ1077**	
DJ694;2.1,1077	Bg2;3.3,1077	13-Nov-07	0.15	**6.01**
DJ694;2.1,1077	Bg2;3.3,1077	13-Nov-07	0.15	**5.76**
DJ694;2.1,1077	Bg2;3.3,1077	13-Nov-07	0.18	**6.93**
DJ694;2.1,1077	Bg2;3.3,1077	30-Nov-07	0.17	**7.42**

Early pupae

Parents		Date	Specific activity ΔmOD/min/µg protein	% DJ694 control
**Male**	**Female**			
DJ694;2.1,1077	Bg2;3.3,1077	30-Nov-07	0.33	**14.65**
DJ694;2.1,1077	Bg2;3.3,1077	30-Nov-07	0.31	**13.79**
DJ694;2.1,1077	Bg2;3.3,1077	19-Dec-07	0.63	**10.73**
DJ694;2.1,1077	Bg2;3.3,1077	19-Dec-07	0.65	**11.15**
DJ694;2.1,1077	Bg2;3.3,1077	19-Dec-07	0.33	**5.57**
DJ694;2.1,1077	Bg2;3.3,1077	19-Dec-07	1.41	**24.04**
DJ694;2.1,1077	Bg2;3.3,1077	19-Dec-07	1.00	**17.08**
DJ694;2.1,1077	Bg2;3.3,1077	19-Dec-07	1.22	**20.77**
DJ694;3.3,1077	Bg2;2.1,1077	13-Nov-07	0.18	**6.93**
DJ694;3.3,1077	Bg2;2.1,1077	13-Nov-07	0.29	**11.43**
DJ694;3.3,1077	Bg2;2.1,1077	13-Nov-07	0.20	**8.04**
DJ694;3.3,1077	Bg2;2.1,1077	30-Nov-07	0.26	**11.74**
DJ694;3.3,1077	Bg2;2.1,1077	30-Nov-07	0.11	**4.82**
DJ694;3.3,1077	Bg2;2.1,1077	30-Nov-07	0.27	**12.12**
DJ694;3.3,1077	Bg2;2.1,1077	19-Dec-07	0.36	**6.22**
DJ694;3.3,1077	Bg2;2.1,1077	19-Dec-07	0.44	**7.56**
DJ694;3.3,1077	Bg2;2.1,1077	19-Dec-07	0.49	**8.38**
DJ694;3.3,1077	Bg2;2.1,1077	19-Dec-07	0.45	**7.75**
DJ694;3.3,1077	Bg2;2.1,1077	19-Dec-07	0.61	**10.36**
DJ694;3.3,1077	Bg2;2.1,1077	19-Dec-07	0.34	**5.73**
DJ694;2.1,1077	Bg2;3.3,1077	10-Feb-08	0.12	**3.01**
DJ694;2.1,1077	Bg2;3.3,1077	10-Feb-08	0.14	**3.45**
DJ694;2.1,1077	Bg2;3.3,1077	10-Feb-08	0.15	**3.78**
DJ694;2.1,1077	Bg2;3.3,1077	22-Feb-08	0.81	**8.32**
DJ694;2.1,1077	Bg2;3.3,1077	22-Feb-08	0.90	**9.29**
DJ694;2.1,1077	Bg2;3.3,1077	22-Feb-08	0.66	**6.81**
DJ694;3.3,1077	Bg2;2.1,1077	10-Feb-08	0.18	**4.47**
DJ694;3.3,1077	Bg2;2.1,1077	10-Feb-08	0.18	**4.51**
DJ694;3.3,1077	Bg2;2.1,1077	10-Feb-08	0.09	**2.30**
DJ694;3.3,1077	Bg2;2.1,1077	22-Feb-08	0.63	**6.49**
DJ694;3.3,1077	Bg2;2.1,1077	22-Feb-08	0.49	**5.08**
DJ694;3.3,1077	Bg2;2.1,1077	22-Feb-08	0.47	**4.87**
			**DJ694 + 2.1 + 3.3 + DJ1077 + DJ1079**	
DJ694;2.1,1077	Bg2;3.3,1079	13-Nov-07	0.32	**12.75**
DJ694;2.1,1077	Bg2;3.3,1079	13-Nov-07	0.30	**11.70**
DJ694;2.1,1077	Bg2;3.3,1079	13-Nov-07	0.25	**9.99**
DJ694;2.1,1077	Bg2;3.3,1079	30-Nov-07	0.21	**9.42**
DJ694;2.1,1077	Bg2;3.3,1079	30-Nov-07	0.22	**9.94**
DJ694;2.1,1077	Bg2;3.3,1079	30-Nov-07	0.14	**6.31**
DJ694;2.1,1077	Bg2;3.3,1079	19-Dec-07	1.38	**23.55**

Early pupae

Parents		Date	Specific activity ΔmOD/min/µg protein	% DJ694 control
Male	Female			
DJ694;2.1,1077	Bg2;3.3,1079	19-Dec-07	1.10	**18.73**
DJ694;2.1,1077	Bg2;3.3,1079	19-Dec-07	0.95	**16.16**
DJ694;2.1,1077	Bg2;3.3,1079	19-Dec-07	1.94	**32.99**
DJ694;2.1,1077	Bg2;3.3,1079	19-Dec-07	1.62	**27.59**
DJ694;2.1,1077	Bg2;3.3,1079	19-Dec-07	1.56	**26.65**
DJ694;2.1,1079(13)	Bg2;3.3,1077	19-Dec-07	0.58	**9.85**
DJ694;2.1,1079(13)	Bg2;3.3,1077	19-Dec-07	0.45	**7.72**
DJ694;2.1,1079(13)	Bg2;3.3,1077	19-Dec-07	0.25	**4.23**
DJ694;2.1,1079(14)	Bg2;3.3,1077	19-Dec-07	2.28	**38.93**
DJ694;2.1,1079(14)	Bg2;3.3,1077	19-Dec-07	1.15	**19.52**
DJ694;2.1,1079(14)	Bg2;3.3,1077	19-Dec-07	1.01	**17.25**
DJ694;2.1,1079(14)	Bg2;3.3,1077	19-Dec-07	0.83	**14.10**
DJ694;2.1,1079(14)	Bg2;3.3,1077	19-Dec-07	0.61	**10.44**
DJ694;2.1,1079(14)	Bg2;3.3,1077	19-Dec-07	0.60	**10.29**
DJ694;3.3,1077	Bg2;2.1,1079(13)	13-Nov-07	0.39	**15.35**
DJ694;3.3,1077	Bg2;2.1,1079(13)	13-Nov-07	0.28	**10.97**
DJ694;3.3,1077	Bg2;2.1,1079(13)	13-Nov-07	0.23	**8.93**
DJ694;3.3,1077	Bg2;2.1,1079(13)	30-Nov-07	0.39	**17.36**
DJ694;3.3,1077	Bg2;2.1,1079(13)	30-Nov-07	0.43	**19.05**
DJ694;3.3,1077	Bg2;2.1,1079(13)	30-Nov-07	0.22	**9.84**
DJ694;3.3,1077	Bg2;2.1,1079(13)	19-Dec-07	0.35	**6.03**
DJ694;3.3,1077	Bg2;2.1,1079(13)	19-Dec-07	0.35	**5.93**
DJ694;3.3,1077	Bg2;2.1,1079(13)	19-Dec-07	0.42	**7.13**
DJ694;3.3,1077	Bg2;2.1,1079(14)	13-Nov-07	0.23	**9.10**
DJ694;3.3,1077	Bg2;2.1,1079(14)	13-Nov-07	0.43	**17.09**
DJ694;3.3,1077	Bg2;2.1,1079(14)	13-Nov-07	0.17	**6.58**
DJ694;3.3,1077	Bg2;2.1,1079(14)	30-Nov-07	0.20	**9.00**
DJ694;3.3,1077	Bg2;2.1,1079(14)	30-Nov-07	0.26	**11.57**
DJ694;3.3,1077	Bg2;2.1,1079(14)	30-Nov-07	0.23	**10.48**
DJ694;3.3,1077	Bg2;2.1,1079(14)	19-Dec-07	0.43	**7.29**
DJ694;3.3,1077	Bg2;2.1,1079(14)	19-Dec-07	1.09	**18.56**
DJ694;3.3,1077	Bg2;2.1,1079(14)	19-Dec-07	1.48	**25.14**
DJ694;3.3,1077	Bg2;2.1,1079(14)	19-Dec-07	1.76	**29.98**
DJ694;3.3,1077	Bg2;2.1,1079(14)	19-Dec-07	1.74	**29.62**
DJ694;3.3,1077	Bg2;2.1,1079(14)	19-Dec-07	2.51	**42.72**
DJ694;1077,1079	Bg2;2.1,3.3	15-Jun-07	0.50	**6.02**
DJ694;1077,1079	Bg2;2.1,3.3	15-Jun-07	0.55	**6.56**
DJ694;1077,1079	Bg2;2.1,3.3	15-Jun-07	0.46	**5.56**
DJ694;1077,1079	Bg2;2.1,3.3	04-Jul-07	0.35	**15.41**
DJ694;1077,1079	Bg2;2.1,3.3	04-Jul-07	0.28	**12.40**

Early pupae

Parents		Date	Specific activity ΔmOD/min/µg protein	% DJ694 control
**Male**	**Female**			
DJ694;1077,1079	Bg2;2.1,3.3	04-Jul-07	0.32	**13.93**
DJ694;1077,1079	Bg2;2.1,3.3	15-Jun-07	0.95	**11.42**
DJ694;2.1,1077	Bg2;3.3,1079	10-Feb-08	0.12	**2.96**
DJ694;2.1,1077	Bg2;3.3,1079	10-Feb-08	0.17	**4.21**
DJ694;2.1,1077	Bg2;3.3,1079	10-Feb-08	0.21	**5.09**
DJ694;2.1,1079(14)	Bg2;3.3,1077	10-Feb-08	3.78	**93.94**
DJ694;2.1,1079(14)	Bg2;3.3,1077	10-Feb-08	0.94	**23.40**
DJ694;2.1,1079(14)	Bg2;3.3,1077	10-Feb-08	0.87	**21.63**
DJ694;2.1,1079(13)	Bg2;3.3,1077	10-Feb-08	0.13	**3.34**
DJ694;2.1,1079(13)	Bg2;3.3,1077	10-Feb-08	0.16	**3.86**
DJ694;2.1,1079(13)	Bg2;3.3,1077	10-Feb-08	0.11	**2.78**
DJ694;2.1,1079(13)	Bg2;3.3,1077	22-Feb-08	0.79	**8.16**
DJ694;2.1,1079(13)	Bg2;3.3,1077	22-Feb-08	1.11	**11.38**
DJ694;2.1,1079(13)	Bg2;3.3,1077	22-Feb-08	1.02	**10.45**
DJ694;3.3,1077	Bg2;2.1,1079(13)	10-Feb-08	0.28	**6.95**
DJ694;3.3,1077	Bg2;2.1,1079(13)	10-Feb-08	0.30	**7.54**
DJ694;3.3,1077	Bg2;2.1,1079(13)	10-Feb-08	0.28	**7.01**
DJ694;3.3,1077	Bg2;2.1,1079(13)	22-Feb-08	0.62	**6.34**
DJ694;3.3,1077	Bg2;2.1,1079(13)	22-Feb-08	0.71	**7.29**
DJ694;3.3,1077	Bg2;2.1,1079(13)	22-Feb-08	0.63	**6.49**
DJ694;3.3,1077	Bg2;2.1,1079(14)	10-Feb-08	0.17	**4.28**
DJ694;3.3,1077	Bg2;2.1,1079(14)	10-Feb-08	0.19	**4.79**
DJ694;3.3,1077	Bg2;2.1,1079(14)	10-Feb-08	0.28	**6.86**
			**DJ694 + 2 copies 2.1 + 2 copies DJ1079**	
DJ694;2.1,1079(13)	Bg;2.1,1079(13)	19-Dec-07	0.60	**10.17**
DJ694;2.1,1079(13)	Bg;2.1,1079(13)	19-Dec-07	0.32	**5.49**
DJ694;2.1,1079(13)	Bg;2.1,1079(13)	19-Dec-07	0.30	**5.20**
DJ694;2.1,1079(13)	Bg2;2.1,1079(14)	19-Dec-07	0.49	**8.31**
DJ694;2.1,1079(13)	Bg2;2.1,1079(14)	19-Dec-07	0.27	**4.64**
DJ694;2.1,1079(13)	Bg2;2.1,1079(14)	19-Dec-07	0.60	**10.30**
DJ694;2.1,1079(14)	Bg2;2.1,1079(13)	19-Dec-07	0.21	**3.59**
DJ694;2.1,1079(14)	Bg2;2.1,1079(13)	19-Dec-07	0.25	**4.20**
DJ694;2.1,1079(14)	Bg2;2.1,1079(13)	19-Dec-07	0.39	**6.62**
DJ694;2.1,1079(14)	Bg2;2.1,1079(14)	19-Dec-07	1.62	**27.56**
DJ694;2.1,1079(14)	Bg2;2.1,1079(14)	19-Dec-07	0.95	**16.11**
DJ694;2.1,1079(14)	Bg2;2.1,1079(14)	19-Dec-07	1.18	**20.09**
DJ694;2.1,1079(14)	Bg2;2.1,1079(14)	19-Dec-07	0.86	**14.64**
DJ694;2.1,1079(14)	Bg2;2.1,1079(14)	19-Dec-07	1.07	**18.15**
DJ694;2.1,1079(14)	Bg2;2.1,1079(14)	19-Dec-07	0.70	**11.94**
DJ694;2.1,1079(14)	Bg2;2.1,1079(13)	10-Feb-08	0.75	**18.69**

Early pupae

Parents		Date	Specific activity ΔmOD/min/μg protein	% DJ694 control
Male	Female			
DJ694;2.1,1079(14)	Bg2;2.1,1079(13)	10-Feb-08	1.19	**29.46**
DJ694;2.1,1079(14)	Bg2;2.1,1079(13)	10-Feb-08	0.58	**14.45**
DJ694;2.1,1079(14)	Bg2;2.1,1079(14)	10-Feb-08	1.19	**29.48**
DJ694;2.1,1079(14)	Bg2;2.1,1079(14)	10-Feb-08	0.51	**12.70**
DJ694;2.1,1079(14)	Bg2;2.1,1079(14)	10-Feb-08	0.71	**17.65**
DJ694;2.1,1079(13)	Bg2;2.1,1079(13)	10-Feb-08	0.22	**5.56**
DJ694;2.1,1079(13)	Bg2;2.1,1079(13)	10-Feb-08	0.24	**6.07**
DJ694;2.1,1079(13)	Bg2;2.1,1079(13)	10-Feb-08	0.26	**6.58**
DJ694;2.1,1079(13)	Bg2;2.1,1079(14)	10-Feb-08	0.10	**2.56**
DJ694;2.1,1079(13)	Bg2;2.1,1079(14)	10-Feb-08	0.13	**3.35**
DJ694;2.1,1079(13)	Bg2;2.1,1079(14)	10-Feb-08	0.18	**4.47**
			**DJ694 + 2.1 + 3.3 + 2 copies DJ1079**	
DJ694;2.1,1079(13)	Bg2;3.3,1079	19-Dec-07	0.49	**8.30**
DJ694;2.1,1079(13)	Bg2;3.3,1079	19-Dec-07	0.45	**7.65**
DJ694;2.1,1079(13)	Bg2;3.3,1079	19-Dec-07	0.58	**9.80**
DJ694;2.1,1079(14)	Bg2;3.3,1079	19-Dec-07	1.13	**19.30**
DJ694;2.1,1079(14)	Bg2;3.3,1079	19-Dec-07	1.79	**30.44**
DJ694;2.1,1079(14)	Bg2;3.3,1079	19-Dec-07	1.34	**22.77**
DJ694;2.1,1079(14)	Bg2;3.3,1079	19-Dec-07	0.86	**14.61**
DJ694;2.1,1079(14)	Bg2;3.3,1079	19-Dec-07	0.51	**8.62**
DJ694;2.1,1079(14)	Bg2;3.3,1079	19-Dec-07	1.52	**25.93**
DJ694;2.1,1079(13)	Bg2;3.3,1079	10-Feb-08	0.27	**6.82**
DJ694;2.1,1079(13)	Bg2;3.3,1079	10-Feb-08	0.35	**8.70**
DJ694;2.1,1079(13)	Bg2;3.3,1079	10-Feb-08	0.45	**11.26**
DJ694;2.1,1079(14)	Bg2;3.3,1079	10-Feb-08	2.04	**50.55**
DJ694;2.1,1079(14)	Bg2;3.3,1079	10-Feb-08	2.37	**58.79**
DJ694;2.1,1079(14)	Bg2;3.3,1079	10-Feb-08	2.48	**61.64**
			**DJ694 + 2 copies 3.3 + 2 copies DJ1077**	
DJ694;3.3,1077	Bg2;3.3,1077	13-Nov-07	0.20	**8.02**
DJ694;3.3,1077	Bg2;3.3,1077	13-Nov-07	0.15	**5.79**
DJ694;3.3,1077	Bg2;3.3,1077	13-Nov-07	0.17	**6.87**
DJ694;3.3,1077	Bg2;3.3,1077	30-Nov-07	0.40	**17.74**
DJ694;3.3,1077	Bg2;3.3,1077	30-Nov-07	0.28	**12.61**
DJ694;3.3,1077	Bg2;3.3,1077	30-Nov-07	0.27	**11.88**
DJ694;3.3,1077	Bg2;3.3,1077	19-Dec-07	0.58	**9.86**
DJ694;3.3,1077	Bg2;3.3,1077	19-Dec-07	0.62	**10.49**
DJ694;3.3,1077	Bg2;3.3,1077	19-Dec-07	0.50	**8.58**
DJ694;3.3,1077	Bg2;3.3,1077	19-Dec-07	0.93	**15.88**
DJ694;3.3,1077	Bg2;3.3,1077	19-Dec-07	0.80	**13.57**
DJ694;3.3,1077	Bg2;3.3,1077	19-Dec-07	0.42	**7.15**

Early pupae

Parents		Date	Specific activity ΔmOD/min/µg protein	% DJ694 control
**Male**	**Female**			
DJ694;3.3,1077	Bg2;3.3,1077	10-Feb-08	0.21	**5.24**
DJ694;3.3,1077	Bg2;3.3,1077	10-Feb-08	0.19	**4.79**
DJ694;3.3,1077	Bg2;3.3,1077	10-Feb-08	0.21	**5.10**
DJ694;3.3,1077	Bg2;3.3,1077	22-Feb-08	0.67	**6.91**
DJ694;3.3,1077	Bg2;3.3,1077	22-Feb-08	1.30	**13.37**
DJ694;3.3,1077	Bg2;3.3,1077	22-Feb-08	0.85	**8.73**
			**DJ694 + 2 copies 3.3 + DJ1077 + DJ1079**	
DJ694;3.3,1077	Bg2;3.3,1079	13-Nov-07	0.26	**10.31**
DJ694;3.3,1077	Bg2;3.3,1079	13-Nov-07	0.21	**8.29**
DJ694;3.3,1077	Bg2;3.3,1079	13-Nov-07	0.21	**8.33**
DJ694;3.3,1077	Bg2;3.3,1079	30-Nov-07	0.28	**12.44**
DJ694;3.3,1077	Bg2;3.3,1079	30-Nov-07	0.21	**9.23**
DJ694;3.3,1077	Bg2;3.3,1079	30-Nov-07	0.18	**8.21**
DJ694;3.3,1077	Bg2;3.3,1079	19-Dec-07	1.27	**21.62**
DJ694;3.3,1077	Bg2;3.3,1079	19-Dec-07	0.52	**8.91**
DJ694;3.3,1077	Bg2;3.3,1079	19-Dec-07	0.50	**8.58**
DJ694;3.3,1077	Bg2;3.3,1079	19-Dec-07	0.51	**8.66**
DJ694;3.3,1077	Bg2;3.3,1079	19-Dec-07	0.42	**7.14**
DJ694;3.3,1077	Bg2;3.3,1079	19-Dec-07	0.46	**7.83**
DJ694;3.3,1077	Bg2;3.3,1079	10-Feb-08	0.13	**3.22**
DJ694;3.3,1077	Bg2;3.3,1079	10-Feb-08	0.13	**3.29**
DJ694;3.3,1077	Bg2;3.3,1079	10-Feb-08	0.29	**7.23**
			**DJ694 + 2.1 + 3.3 + DJ1077 + DJ1080**	
DJ694;2.1,3.3	Bg2;1077,1080	31-May-07	0.07	**1.33**
DJ694;2.1,3.3	Bg2;1077,1080	31-May-07	0.06	**1.16**
DJ694;2.1,3.3	Bg2;1077,1080	31-May-07	0.08	**1.63**
DJ694;2.1,3.3	Bg2;1077,1080	31-May-07	0.04	**0.76**
DJ694;2.1,3.3	Bg2;1077,1080	31-May-07	0.41	**8.28**
DJ694;1077,1080	Bg2;2.1,3.3	15-Jun-07	1.73	**20.69**
DJ694;1077,1080	Bg2;2.1,3.3	15-Jun-07	0.86	**10.30**
DJ694;1077,1080	Bg2;2.1,3.3	04-Jul-07	0.15	**6.57**
DJ694;1077,1080	Bg2;2.1,3.3	04-Jul-07	0.67	**29.71**
DJ694;1077,1080	Bg2;2.1,3.3	04-Jul-07	0.52	**23.16**
			**DJ694 + 2.1 + 3.3 + DJ1080 + DJ1083**	
DJ694;1080,1083	Bg2;2.1,3.3	31-May-07	2.12	**43.05**
DJ694;1080,1083	Bg2;2.1,3.3	31-May-07	2.30	**46.71**
DJ694;1080,1083	Bg2;2.1,3.3	31-May-07	3.38	**68.52**
DJ694;1080,1083	Bg2;2.1,3.3	31-May-07	2.39	**48.61**
DJ694;1080,1083	Bg2;2.1,3.3	31-May-07	0.86	**17.51**

Early pupae

Parents		Date	Specific activity ΔmOD/min/μg protein	% DJ694 control
Male	Female			
			**DJ694 + 2.1 + 2 copies DJ1077 + DJ1079**	
DJ694;1077,1079	Bg2;2.1,1077	19-Dec-07	0.76	**12.99**
DJ694;1077,1079	Bg2;2.1,1077	19-Dec-07	0.43	**7.32**
DJ694;1077,1079	Bg2;2.1,1077	19-Dec-07	0.37	**6.32**
			**DJ694 + 2.1 + DJ1077 + 2 copies DJ1079**	
DJ694;1077,1079	Bg2;2.1,1079(14)	19-Dec-07	0.65	**11.03**
DJ694;1077,1079	Bg2;2.1,1079(14)	19-Dec-07	0.56	**9.47**
DJ694;1077,1079	Bg2;2.1,1079(14)	19-Dec-07	0.81	**13.80**
			**DJ694 + 3.3 + 2 copies DJ1077 + DJ1079**	
DJ694;1077,1079	Bg2;3.3,1077	19-Dec-07	0.87	**14.84**
DJ694;1077,1079	Bg2;3.3,1077	19-Dec-07	0.71	**12.13**
DJ694;1077,1079	Bg2;3.3,1077	19-Dec-07	0.68	**11.52**
			**DJ694 + 3.3 + DJ1077 + 2 copies DJ1079**	
DJ694;1077,1079	Bg2;3.3,1079	19-Dec-07	0.81	**13.88**
DJ694;1077,1079	Bg2;3.3,1079	19-Dec-07	0.87	**14.84**
DJ694;1077,1079	Bg2;3.3,1079	19-Dec-07	0.94	**15.95**
			**DJ694 + 2.1 + 2 copies DJ1077 + DJ1080**	
DJ694;1077,1080	Bg2;2.1,1077	10-Feb-08	1.56	**38.74**
DJ694;1077,1080	Bg2;2.1,1077	10-Feb-08	0.57	**14.20**
DJ694;1077,1080	Bg2;2.1,1077	10-Feb-08	1.34	**33.23**
DJ694;2.1,1077	Bg2;1077,1080	10-Feb-08	0.12	**3.04**
DJ694;2.1,1077	Bg2;1077,1080	10-Feb-08	0.10	**2.38**
DJ694;2.1,1077	Bg2;1077,1080	10-Feb-08	0.13	**3.17**
			**DJ694 + 2.1 + DJ1077 + DJ1079 + DJ1080**	
DJ694;2.1,1079(13)	Bg2;1077,1080	10-Feb-08	0.30	**7.34**
DJ694;2.1,1079(13)	Bg2;1077,1080	10-Feb-08	0.20	**5.05**
DJ694;1077,1080	Bg2;2.1,1079(13)	10-Feb-08	0.33	**8.27**
DJ694;1077,1080	Bg2;2.1,1079(13)	10-Feb-08	1.77	**44.03**
DJ694;1077,1080	Bg2;2.1,1079(13)	10-Feb-08	1.26	**31.17**
DJ694;1077,1080	Bg2;2.1,1079(14)	10-Feb-08	0.62	**15.49**
DJ694;1077,1080	Bg2;2.1,1079(14)	10-Feb-08	0.45	**11.18**
DJ694;1077,1080	Bg2;2.1,1079(14)	10-Feb-08	1.06	**26.20**
DJ694;2.1,1079(14)	Bg2;1077,1080	10-Feb-08	0.39	**9.76**
DJ694;2.1,1079(14)	Bg2;1077,1080	10-Feb-08	0.32	**7.87**
DJ694;2.1,1079(14)	Bg2;1077,1080	10-Feb-08	1.29	**32.03**
			**DJ694 + 3.3 + 2 copies DJ1077 + DJ1080**	
DJ694;1077,1080	Bg2;3.3,1077	10-Feb-08	0.29	**7.29**
DJ694;1077,1080	Bg2;3.3,1077	10-Feb-08	1.09	**27.10**
DJ694;1077,1080	Bg2;3.3,1077	10-Feb-08	0.73	**18.10**

Early pupae

Parents		Date	Specific activity ΔmOD/min/µg protein	% DJ694 control
**Male**	**Female**			
DJ694;3.3,1077	Bg2;1077,1080	10-Feb-08	0.20	**5.02**
DJ694;3.3,1077	Bg2;1077,1080	10-Feb-08	0.17	**4.10**
DJ694;3.3,1077	Bg2;1077,1080	10-Feb-08	0.24	**5.85**
			**DJ694 + 3.3 + DJ1077 + DJ1079 + DJ1080**	
DJ694;1077,1080	Bg2;3.3,1079	10-Feb-08	0.31	**7.66**
DJ694;1077,1080	Bg2;3.3,1079	10-Feb-08	0.70	**17.45**
DJ694;1077,1080	Bg2;3.3,1079	10-Feb-08	0.92	**22.92**
			**DJ694 + 2.1 + DJ1077 + DJ1080 + DJ1083**	
DJ694;2.1,1077	Bg2;1080,1083	10-Feb-08	0.23	**5.59**
DJ694;2.1,1077	Bg2;1080,1083	10-Feb-08	0.19	**4.79**
DJ694;2.1,1077	Bg2;1080,1083	10-Feb-08	0.18	**4.35**
			**DJ694 + 2.1 + DJ1079 + DJ1080 + DJ1083**	
DJ694;2.1,1079(13)	Bg2;1080,1083	10-Feb-08	0.18	**4.55**
DJ694;2.1,1079(13)	Bg2;1080,1083	10-Feb-08	0.26	**6.44**
DJ694;2.1,1079(13)	Bg2;1080,1083	10-Feb-08	0.11	**2.72**
DJ694;2.1,1079(14)	Bg2;1080,1083	10-Feb-08	0.46	**11.47**
DJ694;2.1,1079(14)	Bg2;1080,1083	10-Feb-08	0.24	**5.97**
DJ694;2.1,1079(14)	Bg2;1080,1083	10-Feb-08	0.71	**17.55**
			**DJ694 + 3.3 + DJ1077 + DJ1080 + DJ1083**	
DJ694;3.3,1077	Bg2;1080,1083	10-Feb-08	0.27	**6.63**
DJ694;3.3,1077	Bg2;1080,1083	10-Feb-08	0.22	**5.50**
DJ694;3.3,1077	Bg2;1080,1083	10-Feb-08	0.19	**4.72**

Supplemental Table 7: UAS-lacZ assay LP data set.

% DJ694 control: specific activity normalized to the positive control, bolded values are significantly different ($p<0.05$) from the positive control.

Late pupae

Parents		Date	Specific activity ΔmOD/min/µg protein	% DJ694 control
Male	Female			
			DJ694 + DJ1077	
DJ694;1077	Bg2	15-Jun-07	7.50	**35.56**
DJ694;1077	Bg2	15-Jun-07	7.29	**34.56**
DJ694;1077	Bg2	15-Jun-07	7.59	**35.97**
DJ694;1077	Bg2	04-Jul-07	1.60	**20.60**
DJ694;1077	Bg2	04-Jul-07	2.75	**35.48**
DJ694;1077	Bg2	04-Jul-07	2.76	**35.63**
DJ694;1077	Bg2	31-May-07	11.99	**27.84**
DJ694;1077	Bg2	31-May-07	12.64	**29.36**
DJ694;1077	Bg2	31-May-07	14.03	**32.57**
DJ694;1077	Bg2	31-May-07	13.68	**31.76**
			DJ694 + DJ1080	
DJ694;1080	Bg2	15-Jun-07	11.36	53.82
DJ694;1080	Bg2	15-Jun-07	14.25	67.53
DJ694;1080	Bg2	15-Jun-07	8.38	39.72
DJ694;1080	Bg2	31-May-07	13.44	**31.22**
DJ694;1080	Bg2	31-May-07	17.07	**39.65**
DJ694;1080	Bg2	31-May-07	11.27	**26.17**
DJ694;1080	Bg2	31-May-07	10.37	**24.08**
DJ694;1080	Bg2	04-Jul-07	3.41	**44.00**
DJ694;1080	Bg2	04-Jul-07	3.04	**39.22**
DJ694;1080	Bg2	04-Jul-07	4.80	**61.94**
			DJ694 + DJ1077 + DJ1080	
DJ694	Bg2;1077,1080	03-Aug-07	4.09	**17.97**
DJ694	Bg2;1077,1080	03-Aug-07	2.56	**11.26**
DJ694	Bg2;1077,1080	15-Aug-07	1.29	**35.36**
DJ694	Bg2;1077,1080	15-Aug-07	0.74	**20.20**
DJ694	Bg2;1077,1080	15-Aug-07	0.89	**24.37**
DJ694	Bg2;1077,1080	30-Jul-07	3.53	**28.96**
DJ694	Bg2;1077,1080	30-Jul-07	3.47	**28.46**
DJ694	Bg2;1077,1080	30-Jul-07	3.76	**30.82**
			DJ694 + DJ1080 + DJ1083	
DJ694	Bg2;1080,1083	15-Jun-07	12.38	58.69
DJ694	Bg2;1080,1083	15-Jun-07	13.00	61.59
DJ694	Bg2;1080,1083	15-Jun-07	13.40	63.52
DJ694	Bg2;1080,1083	04-Jul-07	3.98	**51.41**
DJ694	Bg2;1080,1083	04-Jul-07	4.22	**54.52**
DJ694	Bg2;1080,1083	04-Jul-07	4.05	**52.28**
DJ694	Bg2;1080,1083	12-Jul-07	7.42	**71.27**

Late pupae

Parents		Date	Specific activity ΔmOD/min/µg protein	% DJ694 control
**Male**	**Female**			
DJ694	Bg2;1080,1083	12-Jul-07	6.13	**58.88**
DJ694	Bg2;1080,1083	12-Jul-07	6.09	**58.49**
			**DJ694 + DJ1077 + DJ1079**	
DJ694;1077,1079	Bg2	15-Aug-07	1.60	**43.82**
DJ694;1077,1079	Bg2	15-Aug-07	1.75	**47.97**
DJ694;1077,1079	Bg2	15-Aug-07	1.37	**37.66**
DJ694;1077,1079	Bg2	15-Jun-07	8.18	**38.75**
DJ694;1077,1079	Bg2	15-Jun-07	11.98	**56.76**
DJ694;1077,1079	Bg2	15-Jun-07	7.07	**33.51**
DJ694;1077,1079	Bg2	04-Jul-07	3.37	**43.50**
DJ694;1077,1079	Bg2	04-Jul-07	3.74	**48.29**
DJ694;1077,1079	Bg2	04-Jul-07	3.33	**42.94**
			**DJ694 + DJ1077 + DJ1080**	
DJ694;1077,1080	Bg2	15-Jun-07	9.72	46.08
DJ694;1077,1080	Bg2	15-Jun-07	9.98	47.32
DJ694;1077,1080	Bg2	15-Jun-07	13.69	64.88
DJ694;1077,1080	Bg2	04-Jul-07	2.88	**37.23**
DJ694;1077,1080	Bg2	04-Jul-07	3.29	**42.47**
DJ694;1077,1080	Bg2	04-Jul-07	3.53	**45.54**
			**DJ694 + 2 copies DJ1077 + DJ1080**	
DJ694;1077	Bg2;1077,1080	03-Aug-07	4.83	**21.22**
DJ694;1077	Bg2;1077,1080	03-Aug-07	4.67	**20.53**
DJ694;1077	Bg2;1077,1080	03-Aug-07	4.59	**20.17**
DJ694;1077	Bg2;1077,1080	15-Aug-07	0.34	**9.25**
DJ694;1077	Bg2;1077,1080	15-Aug-07	0.87	**23.91**
DJ694;1077	Bg2;1077,1080	15-Aug-07	0.18	**5.02**
DJ694;1077	Bg2;1077,1080	12-Jul-07	3.15	**30.23**
DJ694;1077	Bg2;1077,1080	12-Jul-07	3.03	**29.10**
DJ694;1077	Bg2;1077,1080	12-Jul-07	3.48	**33.39**
DJ694;1077	Bg2;1077,1080	30-Jul-07	2.58	**21.12**
DJ694;1077	Bg2;1077,1080	30-Jul-07	2.32	**18.98**
DJ694;1077	Bg2;1077,1080	30-Jul-07	2.50	**20.46**
			**DJ694 + DJ1077 + DJ0180 + DJ1083**	
DJ694;1077	Bg2;1080,1083	03-Aug-07	4.55	**19.99**
DJ694;1077	Bg2;1080,1083	03-Aug-07	4.84	**21.25**
DJ694;1077	Bg2;1080,1083	03-Aug-07	3.38	**14.86**
DJ694;1077	Bg2;1080,1083	15-Aug-07	0.94	**25.73**
DJ694;1077	Bg2;1080,1083	15-Aug-07	1.23	**33.76**
DJ694;1077	Bg2;1080,1083	15-Aug-07	1.01	**27.67**

Late pupae

Parents		Date	Specific activity ΔmOD/min/µg protein	% DJ694 control
Male	Female			
DJ694;1077	Bg2;1080,1083	30-Jul-07	3.80	**31.13**
DJ694;1077	Bg2;1080,1083	30-Jul-07	3.30	**27.02**
DJ694;1077	Bg2;1080,1083	30-Jul-07	3.51	**28.76**
			**DJ694 + DJ1077 + 2 copies DJ1080**	
DJ694;1080	Bg2;1077,1080	03-Aug-07	5.37	**23.59**
DJ694;1080	Bg2;1077,1080	03-Aug-07	3.94	**17.30**
DJ694;1080	Bg2;1077,1080	03-Aug-07	4.10	**18.04**
DJ694;1080	Bg2;1077,1080	15-Aug-07	0.82	**22.44**
DJ694;1080	Bg2;1077,1080	15-Aug-07	0.88	**24.01**
DJ694;1080	Bg2;1077,1080	15-Aug-07	0.91	**24.95**
DJ694;1080	Bg2;1077,1080	12-Jul-07	3.40	**32.68**
DJ694;1080	Bg2;1077,1080	12-Jul-07	3.13	**30.11**
DJ694;1080	Bg2;1077,1080	12-Jul-07	3.28	**31.48**
DJ694;1080	Bg2;1077,1080	30-Jul-07	2.37	**19.44**
DJ694;1080	Bg2;1077,1080	30-Jul-07	2.65	**21.70**
DJ694;1080	Bg2;1077,1080	30-Jul-07	3.28	**26.87**
			**DJ694 + 2 copies DJ1080 + DJ1083**	
DJ694;1080	Bg2;1080,1083	03-Aug-07	2.86	**12.58**
DJ694;1080	Bg2;1080,1083	03-Aug-07	3.97	**17.43**
DJ694;1080	Bg2;1080,1083	03-Aug-07	3.37	**14.81**
DJ694;1080	Bg2;1080,1083	15-Aug-07	1.35	**36.93**
DJ694;1080	Bg2;1080,1083	15-Aug-07	0.99	**27.23**
DJ694;1080	Bg2;1080,1083	15-Aug-07	1.11	**30.42**
DJ694;1080	Bg2;1080,1083	30-Jul-07	3.77	**30.91**
DJ694;1080	Bg2;1080,1083	30-Jul-07	3.98	**32.64**
DJ694;1080	Bg2;1080,1083	30-Jul-07	4.19	**34.34**
			**DJ694 + 2 copies DJ1077 + DJ1079 + DJ1080**	
DJ694;1077,1079	Bg2;1077,1080	15-Jun-07	5.95	**28.19**
DJ694;1077,1079	Bg2;1077,1080	15-Jun-07	7.33	**34.72**
DJ694;1077,1079	Bg2;1077,1080	15-Jun-07	5.76	**27.29**
DJ694;1077,1079	Bg2;1077,1080	04-Jul-07	1.98	**25.53**
DJ694;1077,1079	Bg2;1077,1080	04-Jul-07	2.71	**34.98**
DJ694;1077,1079	Bg2;1077,1080	04-Jul-07	2.15	**27.76**
			**DJ694 + DJ1077 + DJ1079 + DJ1080 + DJ1083**	
DJ694;1077,1079	Bg2;1080,1083	04-Jul-07	2.47	**31.84**
DJ694;1077,1079	Bg2;1080,1083	04-Jul-07	2.52	**32.57**
DJ694;1077,1079	Bg2;1080,1083	04-Jul-07	1.94	**25.03**
DJ694;1077,1079	Bg2;1080,1083	12-Jul-07	3.96	**38.05**
DJ694;1077,1079	Bg2;1080,1083	12-Jul-07	3.68	**35.32**

Late pupae

Parents		Date	Specific activity	% DJ694 control
**Male**	**Female**		**ΔmOD/min/μg protein**	
DJ694;1077,1079	Bg2;1080,1083	12-Jul-07	3.89	**37.36**
			**DJ694 + 2 copies DJ1077 + 2 copies DJ1080**	
DJ694;1077,1080	Bg2;1077,1080	15-Jun-07	6.99	**33.12**
DJ694;1077,1080	Bg2;1077,1080	15-Jun-07	6.74	**31.96**
DJ694;1077,1080	Bg2;1077,1080	15-Jun-07	7.44	**35.27**
DJ694;1077,1080	Bg2;1077,1080	31-May-07	9.03	**20.96**
DJ694;1077,1080	Bg2;1077,1080	31-May-07	8.56	**19.87**
DJ694;1077,1080	Bg2;1077,1080	31-May-07	7.31	**16.98**
DJ694;1077,1080	Bg2;1077,1080	31-May-07	9.07	**21.07**
DJ694;1077,1080	Bg2;1077,1080	10-Feb-08	19.30	**66.52**
DJ694;1077,1080	Bg2;1077,1080	10-Feb-08	4.35	**15.01**
DJ694;1077,1080	Bg2;1077,1080	10-Feb-08	7.17	**24.70**
DJ694;1077,1080	Bg2;1077,1080	04-Jul-07	2.84	**36.68**
DJ694;1077,1080	Bg2;1077,1080	04-Jul-07	3.07	**39.67**
DJ694;1077,1080	Bg2;1077,1080	04-Jul-07	2.65	**34.16**
			**DJ694 + 2.1**	
DJ694;2.1	Bg2	15-Jun-07	10.46	**49.55**
DJ694;2.1	Bg2	15-Jun-07	10.10	**47.86**
DJ694;2.1	Bg2	15-Jun-07	10.96	**51.93**
DJ694;2.1	Bg2	31-May-07	12.47	**28.96**
DJ694;2.1	Bg2	31-May-07	17.19	**39.92**
DJ694;2.1	Bg2	31-May-07	16.86	**39.16**
DJ694;2.1	Bg2	31-May-07	14.92	**34.66**
DJ694;2.1	Bg2	04-Jul-07	4.22	**54.46**
DJ694;2.1	Bg2	04-Jul-07	3.85	**49.70**
DJ694;2.1	Bg2	04-Jul-07	4.85	**62.59**
			**DJ694 + 3.3**	
DJ694;3.3	Bg2	15-Jun-07	8.78	**41.60**
DJ694;3.3	Bg2	15-Jun-07	9.96	**47.19**
DJ694;3.3	Bg2	15-Jun-07	9.15	**43.36**
DJ694;3.3	Bg2	31-May-07	9.98	**23.18**
DJ694;3.3	Bg2	31-May-07	10.88	**25.27**
DJ694;3.3	Bg2	31-May-07	11.86	**27.54**
DJ694;3.3	Bg2	31-May-07	9.88	**22.95**
DJ694;3.3	Bg2	04-Jul-07	3.65	**47.12**
DJ694;3.3	Bg2	04-Jul-07	2.87	**37.01**
DJ694;3.3	Bg2	04-Jul-07	3.19	**41.15**
			**DJ694 + 2.1 + 3.3**	
DJ694	Bg2;2.1,3.3	04-Jul-07	3.74	**48.24**

Late pupae

Parents		Date	Specific activity ΔmOD/min/µg protein	% DJ694 control
**Male**	**Female**			
DJ694	Bg2;2.1,3.3	04-Jul-07	3.60	**46.44**
DJ694	Bg2;2.1,3.3	04-Jul-07	4.20	**54.26**
DJ694	Bg2;2.1,3.3	12-Jul-07	4.99	**47.93**
DJ694	Bg2;2.1,3.3	12-Jul-07	4.18	**40.12**
DJ694	Bg2;2.1,3.3	12-Jul-07	5.63	**54.12**
			**DJ694 + 2 copies 2.1 + 3.3**	
DJ694;2,1	Bg2;2.1,3.3	03-Aug-07	3.01	**13.22**
DJ694;2,1	Bg2;2.1,3.3	03-Aug-07	4.88	**21.46**
DJ694;2,1	Bg2;2.1,3.3	03-Aug-07	5.45	**23.98**
DJ694;2,1	Bg2;2.1,3.3	15-Aug-07	1.29	**35.34**
DJ694;2,1	Bg2;2.1,3.3	15-Aug-07	0.98	**26.75**
DJ694;2,1	Bg2;2.1,3.3	15-Aug-07	1.22	**33.39**
DJ694;2,1	Bg2;2.1,3.3	12-Jul-07	2.18	**20.90**
DJ694;2,1	Bg2;2.1,3.3	12-Jul-07	3.56	**34.23**
DJ694;2,1	Bg2;2.1,3.3	12-Jul-07	2.67	**25.62**
			**DJ694 + 2.1 + 2 copies 3.3**	
DJ694;3.3	Bg2;2.1,3.3	03-Aug-07	3.46	15.21
DJ694;3.3	Bg2;2.1,3.3	15-Aug-07	1.32	**36.24**
DJ694;3.3	Bg2;2.1,3.3	15-Aug-07	1.35	**37.07**
DJ694;3.3	Bg2;2.1,3.3	15-Aug-07	1.36	**37.21**
DJ694;3.3	Bg2;2.1,3.3	12-Jul-07	2.24	**21.54**
DJ694;3.3	Bg2;2.1,3.3	12-Jul-07	2.18	**20.97**
DJ694;3.3	Bg2;2.1,3.3	12-Jul-07	3.25	**31.21**
DJ694;3.3	Bg2;2.1,3.3	30-Jul-07	1.44	**11.81**
DJ694;3.3	Bg2;2.1,3.3	30-Jul-07	1.67	**13.70**
			**DJ694 + 2.1 + DJ1077**	
DJ694	Bg2;2.1,1077	13-Nov-07	1.79	**22.65**
DJ694	Bg2;2.1,1077	13-Nov-07	1.62	**20.48**
DJ694	Bg2;2.1,1077	13-Nov-07	1.62	**20.42**
DJ694	Bg2;2.1,1077	30-Nov-07	1.93	**19.10**
DJ694	Bg2;2.1,1077	30-Nov-07	2.64	**26.14**
DJ694	Bg2;2.1,1077	30-Nov-07	3.12	**30.90**
DJ694;2.1,1077	Bg2	22-Feb-08	1.79	**9.47**
DJ694;2.1,1077	Bg2	22-Feb-08	1.66	**8.78**
DJ694;2.1,1077	Bg2	22-Feb-08	2.02	**10.69**
			**DJ694 + 2.1 + DJ1079**	
DJ694	Bg2;2.1,1079(13)	13-Nov-07	0.90	**11.37**
DJ694	Bg2;2.1,1079(13)	13-Nov-07	0.77	**9.76**
DJ694	Bg2;2.1,1079(13)	13-Nov-07	0.94	**11.82**

Late pupae

Parents		Date	Specific activity ΔmOD/min/μg protein	% DJ694 control
**Male**	**Female**			
DJ694	Bg2;2.1,1079(13)	30-Nov-07	0.77	**7.64**
DJ694	Bg2;2.1,1079(13)	30-Nov-07	2.04	**20.19**
DJ694	Bg2;2.1,1079(13)	30-Nov-07	1.88	**18.58**
DJ694	Bg2;2.1,1079(14)	13-Nov-07	3.33	**42.06**
DJ694	Bg2;2.1,1079(14)	13-Nov-07	3.47	**43.76**
DJ694	Bg2;2.1,1079(14)	13-Nov-07	3.19	**40.32**
DJ694	Bg2;2.1,1079(14)	30-Nov-07	4.26	**42.12**
DJ694	Bg2;2.1,1079(14)	30-Nov-07	0.84	**8.35**
DJ694	Bg2;2.1,1079(14)	30-Nov-07	3.29	**32.57**
DJ694;2.1,1079(13)	Bg2	22-Feb-08	1.34	**7.06**
DJ694;2.1,1079(13)	Bg2	22-Feb-08	0.96	**5.06**
DJ694;2.1,1079(13)	Bg2	22-Feb-08	1.58	**8.35**
			**DJ694 + 3.3 + DJ1077**	
DJ694	Bg2;3.3,1077	13-Nov-07	2.21	**27.86**
DJ694	Bg2;3.3,1077	13-Nov-07	1.96	**24.78**
DJ694	Bg2;3.3,1077	13-Nov-07	1.79	**22.56**
DJ694	Bg2;3.3,1077	30-Nov-07	1.44	**14.25**
DJ694	Bg2;3.3,1077	30-Nov-07	1.94	**19.16**
DJ694	Bg2;3.3,1077	30-Nov-07	1.08	**10.64**
DJ694;3.3,1077	Bg2	22-Feb-08	1.84	**9.70**
DJ694;3.3,1077	Bg2	22-Feb-08	1.45	**7.68**
DJ694;3.3,1077	Bg2	22-Feb-08	4.17	22.04
			**DJ694 + 3.3 + DJ1079**	
DJ694	Bg2;3.3,1079	13-Nov-07	3.08	**38.84**
DJ694	Bg2;3.3,1079	13-Nov-07	3.93	**49.63**
DJ694	Bg2;3.3,1079	13-Nov-07	4.29	**54.12**
DJ694	Bg2;3.3,1079	30-Nov-07	3.89	**38.48**
DJ694	Bg2;3.3,1079	30-Nov-07	3.92	**38.75**
DJ694	Bg2;3.3,1079	30-Nov-07	4.40	**43.49**
			**DJ694 + 2.1 + DJ1077 + DJ1080**	
DJ694;2.1	Bg2;1077,1080	03-Aug-07	1.70	**7.49**
DJ694;2.1	Bg2;1077,1080	03-Aug-07	2.44	**10.74**
DJ694;2.1	Bg2;1077,1080	03-Aug-07	2.07	**9.11**
DJ694;2.1	Bg2;1077,1080	15-Aug-07	0.38	**10.30**
DJ694;2.1	Bg2;1077,1080	15-Aug-07	0.32	**8.81**
DJ694;2.1	Bg2;1077,1080	15-Aug-07	0.37	**10.26**
DJ694;2.1	Bg2;1077,1080	12-Jul-07	2.74	**26.35**
DJ694;2.1	Bg2;1077,1080	12-Jul-07	3.80	**36.53**
DJ694;2.1	Bg2;1077,1080	12-Jul-07	3.03	**29.14**

Late pupae

Parents		Date	Specific activity ΔmOD/min/μg protein	% DJ694 control
Male	Female			
DJ694;2.1	Bg2;1077,1080	30-Jul-07	0.85	**6.94**
DJ694;2.1	Bg2;1077,1080	30-Jul-07	1.73	**14.17**
DJ694;2.1	Bg2;1077,1080	30-Jul-07	0.99	**8.12**
			**DJ694 + 2.1 + DJ1080 + DJ1083**	
DJ694;2.1	Bg2;1080,1083	03-Aug-07	1.73	**7.62**
DJ694;2.1	Bg2;1080,1083	03-Aug-07	3.09	**13.58**
DJ694;2.1	Bg2;1080,1083	03-Aug-07	4.67	**20.53**
DJ694;2.1	Bg2;1080,1083	15-Aug-07	0.64	**17.53**
DJ694;2.1	Bg2;1080,1083	15-Aug-07	0.51	**13.84**
DJ694;2.1	Bg2;1080,1083	15-Aug-07	0.78	**21.38**
DJ694;2.1	Bg2;1080,1083	12-Jul-07	1.87	**17.93**
DJ694;2.1	Bg2;1080,1083	12-Jul-07	1.74	**16.75**
DJ694;2.1	Bg2;1080,1083	12-Jul-07	2.04	**19.58**
DJ694;2.1	Bg2;1080,1083	30-Jul-07	2.36	**19.31**
DJ694;2.1	Bg2;1080,1083	30-Jul-07	2.22	**18.18**
DJ694;2.1	Bg2;1080,1083	30-Jul-07	2.42	**19.84**
			**DJ694 + 3.3 + DJ1077 + DJ1080**	
DJ694;3.3	Bg2;1077,1080	03-Aug-07	1.29	**5.65**
DJ694;3.3	Bg2;1077,1080	03-Aug-07	1.10	**4.84**
DJ694;3.3	Bg2;1077,1080	03-Aug-07	0.87	**3.84**
DJ694;3.3	Bg2;1077,1080	15-Aug-07	0.18	**4.81**
DJ694;3.3	Bg2;1077,1080	15-Aug-07	0.25	**6.94**
DJ694;3.3	Bg2;1077,1080	15-Aug-07	0.22	**6.15**
DJ694;3.3	Bg2;1077,1080	12-Jul-07	0.84	**8.05**
DJ694;3.3	Bg2;1077,1080	12-Jul-07	0.87	**8.35**
DJ694;3.3	Bg2;1077,1080	12-Jul-07	1.16	**11.18**
DJ694;3.3	Bg2;1077,1080	30-Jul-07	0.56	**4.63**
DJ694;3.3	Bg2;1077,1080	30-Jul-07	0.69	**5.67**
DJ694;3.3	Bg2;1077,1080	30-Jul-07	0.58	**4.78**
			**DJ694 + 3.3 + DJ1080 + DJ1083**	
DJ694;3.3	Bg2;1080,1083	03-Aug-07	2.47	**10.87**
DJ694;3.3	Bg2;1080,1083	03-Aug-07	3.16	**13.88**
DJ694;3.3	Bg2;1080,1083	03-Aug-07	3.13	**13.76**
DJ694;3.3	Bg2;1080,1083	15-Aug-07	0.42	**11.47**
DJ694;3.3	Bg2;1080,1083	15-Aug-07	0.50	**13.74**
DJ694;3.3	Bg2;1080,1083	15-Aug-07	0.49	**13.38**
DJ694;3.3	Bg2;1080,1083	12-Jul-07	1.21	**11.65**
DJ694;3.3	Bg2;1080,1083	12-Jul-07	1.79	**17.18**
DJ694;3.3	Bg2;1080,1083	12-Jul-07	1.50	**14.44**

Late pupae

Parents		Date	Specific activity ΔmOD/min/µg protein	% DJ694 control
Male	Female			
DJ694;3.3	Bg2;1080,1083	30-Jul-07	1.02	**8.34**
DJ694;3.3	Bg2;1080,1083	30-Jul-07	1.14	**9.37**
DJ694;3.3	Bg2;1080,1083	30-Jul-07	1.25	**10.25**
			**DJ694 + 2.1 + 3.3 + DJ1077**	
DJ694;1077	Bg2;2.1,3.3	03-Aug-07	0.69	**3.05**
DJ694;1077	Bg2;2.1,3.3	03-Aug-07	0.79	**3.48**
DJ694;1077	Bg2;2.1,3.3	03-Aug-07	0.66	**2.89**
DJ694;1077	Bg2;2.1,3.3	15-Aug-07	0.13	**3.44**
DJ694;1077	Bg2;2.1,3.3	15-Aug-07	0.14	**3.93**
DJ694;1077	Bg2;2.1,3.3	15-Aug-07	0.11	**3.11**
DJ694;1077	Bg2;2.1,3.3	04-Jul-07	0.60	**7.78**
DJ694;1077	Bg2;2.1,3.3	04-Jul-07	0.82	**10.63**
DJ694;1077	Bg2;2.1,3.3	04-Jul-07	0.42	**5.44**
DJ694;1077	Bg2;2.1,3.3	12-Jul-07	1.37	**13.12**
DJ694;1077	Bg2;2.1,3.3	12-Jul-07	0.65	**6.22**
DJ694;1077	Bg2;2.1,3.3	12-Jul-07	0.43	**4.16**
DJ694;1077	Bg2;2.1,3.3	30-Jul-07	0.36	**2.95**
DJ694;1077	Bg2;2.1,3.3	30-Jul-07	0.26	**2.16**
			**DJ694 + 2.1 + 3.3 + DJ1080**	
DJ694;1080	Bg2;2.1,3.3	03-Aug-07	1.59	**7.01**
DJ694;1080	Bg2;2.1,3.3	03-Aug-07	1.06	**4.67**
DJ694;1080	Bg2;2.1,3.3	03-Aug-07	1.06	**4.65**
DJ694;1080	Bg2;2.1,3.3	15-Aug-07	0.31	**8.44**
DJ694;1080	Bg2;2.1,3.3	15-Aug-07	0.17	**4.75**
DJ694;1080	Bg2;2.1,3.3	15-Aug-07	0.28	**7.70**
DJ694;1080	Bg2;2.1,3.3	04-Jul-07	0.79	**10.15**
DJ694;1080	Bg2;2.1,3.3	04-Jul-07	1.48	**19.04**
DJ694;1080	Bg2;2.1,3.3	04-Jul-07	0.85	**10.92**
DJ694;1080	Bg2;2.1,3.3	12-Jul-07	1.05	**10.14**
DJ694;1080	Bg2;2.1,3.3	12-Jul-07	0.99	**9.48**
DJ694;1080	Bg2;2.1,3.3	12-Jul-07	0.56	**5.35**
DJ694;1080	Bg2;2.1,3.3	30-Jul-07	0.94	**7.70**
DJ694;1080	Bg2;2.1,3.3	30-Jul-07	0.57	**4.68**
DJ694;1080	Bg2;2.1,3.3	30-Jul-07	0.96	**7.87**
			**DJ694 + 2 copies 2.1 + 2 copies DJ1077**	
DJ694;2.1,1077	Bg2;2.1,1077	13-Nov-07	0.41	**5.14**
DJ694;2.1,1077	Bg2;2.1,1077	13-Nov-07	0.34	**4.34**
DJ694;2.1,1077	Bg2;2.1,1077	13-Nov-07	0.35	**4.43**
DJ694;2.1,1077	Bg2;2.1,1077	30-Nov-07	0.36	**3.61**

Late pupae

Parents		Date	Specific activity ΔmOD/min/μg protein	% DJ694 control
**Male**	**Female**			
DJ694;2.1,1077	Bg2;2.1,1077	30-Nov-07	0.36	**3.55**
DJ694;2.1,1077	Bg2;2.1,1077	30-Nov-07	0.33	**3.26**
DJ694;2.1,1077	Bg2;2.1,1077	19-Dec-07	0.66	**5.31**
DJ694;2.1,1077	Bg2;2.1,1077	19-Dec-07	0.94	**7.58**
DJ694;2.1,1077	Bg2;2.1,1077	19-Dec-07	1.19	**9.62**
DJ694;2.1,1077	Bg2;2.1,1077	19-Dec-07	0.96	**7.75**
DJ694;2.1,1077	Bg2;2.1,1077	19-Dec-07	0.88	**7.10**
DJ694;2.1,1077	Bg2;2.1,1077	19-Dec-07	1.42	**11.51**
DJ694;2.1,1077	Bg2;2.1,1077	10-Feb-08	0.87	**2.99**
DJ694;2.1,1077	Bg2;2.1,1077	10-Feb-08	0.58	**2.01**
DJ694;2.1,1077	Bg2;2.1,1077	10-Feb-08	0.55	**1.91**
DJ694;2.1,1077	Bg2;2.1,1077	22-Feb-08	0.69	**3.66**
DJ694;2.1,1077	Bg2;2.1,1077	22-Feb-08	0.55	**2.91**
DJ694;2.1,1077	Bg2;2.1,1077	22-Feb-08	0.58	**3.04**
			**DJ694 + 2 copies 2.1 + DJ1077 + DJ1079**	
DJ694;2.1,1077	Bg2;2.1,1079(13)	13-Nov-07	0.73	**9.20**
DJ694;2.1,1077	Bg2;2.1,1079(13)	13-Nov-07	0.29	**3.66**
DJ694;2.1,1077	Bg2;2.1,1079(13)	13-Nov-07	0.36	**4.52**
DJ694;2.1,1077	Bg2;2.1,1079(13)	30-Nov-07	0.40	**3.94**
DJ694;2.1,1077	Bg2;2.1,1079(13)	30-Nov-07	0.36	**3.60**
DJ694;2.1,1077	Bg2;2.1,1079(13)	30-Nov-07	0.46	**4.55**
DJ694;2.1,1077	Bg2;2.1,1079(13)	19-Dec-07	0.64	**5.18**
DJ694;2.1,1077	Bg2;2.1,1079(13)	19-Dec-07	0.45	**3.63**
DJ694;2.1,1077	Bg2;2.1,1079(13)	19-Dec-07	0.56	**4.50**
DJ694;2.1,1077	Bg2;2.1,1079(14)	13-Nov-07	0.43	**5.42**
DJ694;2.1,1077	Bg2;2.1,1079(14)	13-Nov-07	0.44	**5.58**
DJ694;2.1,1077	Bg2;2.1,1079(14)	13-Nov-07	0.35	**4.39**
DJ694;2.1,1077	Bg2;2.1,1079(14)	30-Nov-07	0.26	**2.52**
DJ694;2.1,1077	Bg2;2.1,1079(14)	30-Nov-07	0.46	**4.52**
DJ694;2.1,1077	Bg2;2.1,1079(14)	30-Nov-07	0.38	**3.75**
DJ694;2.1,1077	Bg2;2.1,1079(14)	19-Dec-07	2.64	**21.38**
DJ694;2.1,1077	Bg2;2.1,1079(14)	19-Dec-07	2.09	**16.88**
DJ694;2.1,1077	Bg2;2.1,1079(14)	19-Dec-07	1.55	**12.52**
DJ694;2.1,1077	Bg2;2.1,1079(14)	19-Dec-07	1.51	**12.18**
DJ694;2.1,1077	Bg2;2.1,1079(14)	19-Dec-07	3.17	**25.61**
DJ694;2.1,1077	Bg2;2.1,1079(14)	19-Dec-07	1.92	**15.52**
DJ694;2.1,1079(13)	Bg2;2.1,1077	19-Dec-07	0.78	**6.33**
DJ694;2.1,1079(13)	Bg2;2.1,1077	19-Dec-07	0.72	**5.85**
DJ694;2.1,1079(13)	Bg2;2.1,1077	19-Dec-07	0.93	**7.50**
DJ694;2.1,1079(14)	Bg2;2.1,1077	19-Dec-07	1.07	**8.64**

Late pupae

Parents		Date	Specific activity	% DJ694 control
**Male**	**Female**		**ΔmOD/min/μg protein**	
DJ694;2.1,1079(14)	Bg2;2.1,1077	19-Dec-07	2.21	**17.85**
DJ694;2.1,1079(14)	Bg2;2.1,1077	19-Dec-07	0.44	**3.53**
DJ694;2.1,1079(14)	Bg2;2.1,1077	19-Dec-07	0.35	**2.81**
DJ694;2.1,1079(14)	Bg2;2.1,1077	19-Dec-07	0.31	**2.55**
DJ694;2.1,1077	Bg2;2.1,1079(13)	10-Feb-08	0.92	**3.17**
DJ694;2.1,1077	Bg2;2.1,1079(13)	10-Feb-08	2.00	**6.91**
DJ694;2.1,1077	Bg2;2.1,1079(13)	10-Feb-08	1.96	**6.77**
DJ694;2.1,1077	Bg2;2.1,1079(13)	22-Feb-08	0.34	**1.82**
DJ694;2.1,1077	Bg2;2.1,1079(13)	22-Feb-08	0.74	**3.93**
DJ694;2.1,1077	Bg2;2.1,1079(13)	22-Feb-08	0.40	**2.14**
DJ694;2.1,1077	Bg2;2.1,1079(14)	10-Feb-08	0.65	**2.25**
DJ694;2.1,1077	Bg2;2.1,1079(14)	10-Feb-08	0.94	**3.24**
DJ694;2.1,1077	Bg2;2.1,1079(14)	10-Feb-08	1.88	**6.49**
DJ694;2.1,1079(13)	Bg2;2.1,1077	10-Feb-08	0.96	**3.30**
DJ694;2.1,1079(13)	Bg2;2.1,1077	10-Feb-08	1.01	**3.48**
DJ694;2.1,1079(13)	Bg2;2.1,1077	10-Feb-08	1.78	**6.14**
DJ694;2.1,1079(13)	Bg2;2.1,1077	22-Feb-08	2.95	**15.57**
DJ694;2.1,1079(13)	Bg2;2.1,1077	22-Feb-08	1.12	**5.91**
DJ694;2.1,1079(13)	Bg2;2.1,1077	22-Feb-08	0.57	**2.99**
DJ694;2.1,1079(14)	Bg2;2.1,1077	10-Feb-08	2.30	**7.94**
DJ694;2.1,1079(14)	Bg2;2.1,1077	10-Feb-08	2.75	**9.47**
DJ694;2.1,1079(14)	Bg2;2.1,1077	10-Feb-08	3.35	**11.55**
DJ694;2.1,1079(14)	Bg2;2.1,1077	19-Dec-07	3.41	27.56
			**DJ694 + 2.1 + 3.3 + 2 copies DJ1077**	
DJ694;2.1,1077	Bg2;3.3,1077	13-Nov-07	0.43	**5.40**
DJ694;2.1,1077	Bg2;3.3,1077	13-Nov-07	0.29	**3.66**
DJ694;2.1,1077	Bg2;3.3,1077	13-Nov-07	0.30	**3.79**
DJ694;2.1,1077	Bg2;3.3,1077	30-Nov-07	0.26	**2.61**
DJ694;2.1,1077	Bg2;3.3,1077	30-Nov-07	0.23	**2.28**
DJ694;2.1,1077	Bg2;3.3,1077	30-Nov-07	0.29	**2.91**
DJ694;2.1,1077	Bg2;3.3,1077	19-Dec-07	0.74	**6.02**
DJ694;2.1,1077	Bg2;3.3,1077	19-Dec-07	1.01	**8.21**
DJ694;2.1,1077	Bg2;3.3,1077	19-Dec-07	0.81	**6.58**
DJ694;2.1,1077	Bg2;3.3,1077	19-Dec-07	0.71	**5.72**
DJ694;2.1,1077	Bg2;3.3,1077	19-Dec-07	0.98	**7.91**
DJ694;3.3,1077	Bg2;2.1,1077	13-Nov-07	0.23	**2.88**
DJ694;3.3,1077	Bg2;2.1,1077	13-Nov-07	0.36	**4.50**
DJ694;3.3,1077	Bg2;2.1,1077	13-Nov-07	0.33	**4.10**
DJ694;3.3,1077	Bg2;2.1,1077	30-Nov-07	0.34	**3.36**
DJ694;3.3,1077	Bg2;2.1,1077	30-Nov-07	0.41	**4.10**

Late pupae

Parents		Date	Specific activity ΔmOD/min/µg protein	% DJ694 control
**Male**	**Female**			
DJ694;3.3,1077	Bg2;2.1,1077	30-Nov-07	0.48	**4.78**
DJ694;3.3,1077	Bg2;2.1,1077	19-Dec-07	0.89	**7.16**
DJ694;3.3,1077	Bg2;2.1,1077	19-Dec-07	0.81	**6.55**
DJ694;3.3,1077	Bg2;2.1,1077	19-Dec-07	0.78	**6.32**
DJ694;3.3,1077	Bg2;2.1,1077	19-Dec-07	1.05	**8.48**
DJ694;3.3,1077	Bg2;2.1,1077	19-Dec-07	0.45	**3.68**
DJ694;2.1,1077	Bg2;3.3,1077	10-Feb-08	0.55	**1.90**
DJ694;2.1,1077	Bg2;3.3,1077	10-Feb-08	0.61	**2.12**
DJ694;2.1,1077	Bg2;3.3,1077	10-Feb-08	0.54	**1.86**
DJ694;2.1,1077	Bg2;3.3,1077	22-Feb-08	1.26	**6.63**
DJ694;2.1,1077	Bg2;3.3,1077	22-Feb-08	0.40	**2.12**
DJ694;2.1,1077	Bg2;3.3,1077	22-Feb-08	0.37	**1.98**
DJ694;3.3,1077	Bg2;2.1,1077	10-Feb-08	0.56	**1.93**
DJ694;3.3,1077	Bg2;2.1,1077	10-Feb-08	0.57	**1.98**
DJ694;3.3,1077	Bg2;2.1,1077	10-Feb-08	0.63	**2.16**
DJ694;3.3,1077	Bg2;2.1,1077	22-Feb-08	0.61	**3.22**
DJ694;3.3,1077	Bg2;2.1,1077	22-Feb-08	0.61	**3.22**
DJ694;3.3,1077	Bg2;2.1,1077	22-Feb-08	0.69	**3.66**
DJ694;2.1,1077	Bg2;3.3,1077	19-Dec-07	1.70	13.72
DJ694;3.3,1077	Bg2;2.1,1077	19-Dec-07	2.13	17.25
			**DJ694 + 2.1 + 3.3 + DJ1077 + DJ1079**	
DJ694;2.1,1077	Bg2;3.3,1079	13-Nov-07	0.32	**4.06**
DJ694;2.1,1077	Bg2;3.3,1079	13-Nov-07	0.30	**3.82**
DJ694;2.1,1077	Bg2;3.3,1079	13-Nov-07	0.45	**5.71**
DJ694;2.1,1077	Bg2;3.3,1079	30-Nov-07	0.30	**2.99**
DJ694;2.1,1077	Bg2;3.3,1079	30-Nov-07	0.48	**4.78**
DJ694;2.1,1077	Bg2;3.3,1079	30-Nov-07	0.83	**8.21**
DJ694;2.1,1077	Bg2;3.3,1079	19-Dec-07	1.28	**10.36**
DJ694;2.1,1077	Bg2;3.3,1079	19-Dec-07	1.69	**13.70**
DJ694;2.1,1077	Bg2;3.3,1079	19-Dec-07	1.49	**12.02**
DJ694;2.1,1077	Bg2;3.3,1079	19-Dec-07	1.52	**12.32**
DJ694;2.1,1077	Bg2;3.3,1079	19-Dec-07	2.12	**17.14**
DJ694;2.1,1079(13)	Bg2;3.3,1077	19-Dec-07	0.56	**4.56**
DJ694;2.1,1079(13)	Bg2;3.3,1077	19-Dec-07	0.55	**4.43**
DJ694;2.1,1079(14)	Bg2;3.3,1077	19-Dec-07	1.94	**15.67**
DJ694;2.1,1079(14)	Bg2;3.3,1077	19-Dec-07	1.63	**13.17**
DJ694;2.1,1079(14)	Bg2;3.3,1077	19-Dec-07	2.36	**19.08**
DJ694;2.1,1079(14)	Bg2;3.3,1077	19-Dec-07	0.69	**5.58**
DJ694;2.1,1079(14)	Bg2;3.3,1077	19-Dec-07	0.43	**3.49**
DJ694;2.1,1079(14)	Bg2;3.3,1077	19-Dec-07	0.77	**6.25**

Late pupae

Parents		Date	Specific activity ΔmOD/min/µg protein	% DJ694 control
**Male**	**Female**			
DJ694;3.3,1077	Bg2;2.1,1079(13)	13-Nov-07	0.25	**3.14**
DJ694;3.3,1077	Bg2;2.1,1079(13)	13-Nov-07	0.23	**2.94**
DJ694;3.3,1077	Bg2;2.1,1079(13)	13-Nov-07	0.26	**3.26**
DJ694;3.3,1077	Bg2;2.1,1079(13)	30-Nov-07	0.31	**3.03**
DJ694;3.3,1077	Bg2;2.1,1079(13)	30-Nov-07	0.28	**2.81**
DJ694;3.3,1077	Bg2;2.1,1079(13)	30-Nov-07	0.34	**3.35**
DJ694;3.3,1077	Bg2;2.1,1079(13)	19-Dec-07	0.77	**6.20**
DJ694;3.3,1077	Bg2;2.1,1079(13)	19-Dec-07	0.71	**5.74**
DJ694;3.3,1077	Bg2;2.1,1079(13)	19-Dec-07	0.75	**6.08**
DJ694;3.3,1077	Bg2;2.1,1079(14)	13-Nov-07	0.30	**3.79**
DJ694;3.3,1077	Bg2;2.1,1079(14)	13-Nov-07	0.57	**7.19**
DJ694;3.3,1077	Bg2;2.1,1079(14)	13-Nov-07	0.52	**6.51**
DJ694;3.3,1077	Bg2;2.1,1079(14)	30-Nov-07	0.45	**4.46**
DJ694;3.3,1077	Bg2;2.1,1079(14)	30-Nov-07	0.47	**4.63**
DJ694;3.3,1077	Bg2;2.1,1079(14)	30-Nov-07	0.31	**3.06**
DJ694;3.3,1077	Bg2;2.1,1079(14)	19-Dec-07	1.37	**11.09**
DJ694;3.3,1077	Bg2;2.1,1079(14)	19-Dec-07	1.99	**16.12**
DJ694;3.3,1077	Bg2;2.1,1079(14)	19-Dec-07	1.76	**14.26**
DJ694;3.3,1077	Bg2;2.1,1079(14)	19-Dec-07	1.51	**12.24**
DJ694;3.3,1077	Bg2;2.1,1079(14)	19-Dec-07	1.42	**11.53**
DJ694;1077,1079	Bg2;2.1,3.3	15-Jun-07	0.68	**3.23**
DJ694;1077,1079	Bg2;2.1,3.3	15-Jun-07	2.05	**9.70**
DJ694;1077,1079	Bg2;2.1,3.3	15-Jun-07	0.85	**4.01**
DJ694;1077,1079	Bg2;2.1,3.3	04-Jul-07	0.72	**9.24**
DJ694;1077,1079	Bg2;2.1,3.3	04-Jul-07	0.48	**6.16**
DJ694;1077,1079	Bg2;2.1,3.3	04-Jul-07	0.28	**3.68**
DJ694;2.1,1077	Bg2;3.3,1079	10-Feb-08	0.68	**2.34**
DJ694;2.1,1077	Bg2;3.3,1079	10-Feb-08	1.54	**5.32**
DJ694;2.1,1077	Bg2;3.3,1079	10-Feb-08	0.71	**2.44**
DJ694;2.1,1079(13)	Bg2;3.3,1077	10-Feb-08	0.60	**2.08**
DJ694;2.1,1079(13)	Bg2;3.3,1077	10-Feb-08	0.60	**2.07**
DJ694;2.1,1079(13)	Bg2;3.3,1077	10-Feb-08	0.64	**2.22**
DJ694;2.1,1079(13)	Bg2;3.3,1077	10-Feb-08	0.67	**2.32**
DJ694;2.1,1079(13)	Bg2;3.3,1077	22-Feb-08	0.37	**1.97**
DJ694;2.1,1079(13)	Bg2;3.3,1077	22-Feb-08	0.55	**2.90**
DJ694;2.1,1079(13)	Bg2;3.3,1077	22-Feb-08	0.48	**2.53**
DJ694;2.1,1079(14)	Bg2;3.3,1077	10-Feb-08	1.64	**5.65**
DJ694;2.1,1079(14)	Bg2;3.3,1077	10-Feb-08	1.72	**5.92**
DJ694;2.1,1079(14)	Bg2;3.3,1077	10-Feb-08	1.33	**4.57**
DJ694;3.3,1077	Bg2;2.1,1079(13)	10-Feb-08	0.58	**2.00**

Late pupae

Parents		Date	Specific activity ΔmOD/min/µg protein	% DJ694 control
Male	Female			
DJ694;3.3,1077	Bg2;2.1,1079(13)	10-Feb-08	0.86	**2.98**
DJ694;3.3,1077	Bg2;2.1,1079(13)	10-Feb-08	0.57	**1.97**
DJ694;3.3,1077	Bg2;2.1,1079(13)	22-Feb-08	0.55	**2.90**
DJ694;3.3,1077	Bg2;2.1,1079(13)	22-Feb-08	0.46	**2.42**
DJ694;3.3,1077	Bg2;2.1,1079(13)	22-Feb-08	0.55	**2.89**
DJ694;3.3,1077	Bg2;2.1,1079(14)	10-Feb-08	0.58	**1.99**
DJ694;3.3,1077	Bg2;2.1,1079(14)	10-Feb-08	0.96	**3.29**
DJ694;3.3,1077	Bg2;2.1,1079(14)	10-Feb-08	0.73	**2.53**
DJ694;2.1,1077	Bg2;3.3,1079	19-Dec-07	2.70	21.84
DJ694;2.1,1079(13)	Bg2;3.3,1077	19-Dec-07	1.23	9.93
DJ694;3.3,1077	Bg2;2.1,1079(14)	19-Dec-07	0.36	2.90
			**DJ694 + 2 copies 2.1 + 2 copies DJ1079**	
DJ694;2.1,1079(13)	Bg2;2.1,1079(13)	10-Feb-08	0.73	**2.52**
DJ694;2.1,1079(13)	Bg2;2.1,1079(13)	10-Feb-08	0.50	**1.71**
DJ694;2.1,1079(13)	Bg2;2.1,1079(13)	10-Feb-08	0.63	**2.17**
DJ694;2.1,1079(13)	Bg2;2.1,1079(14)	10-Feb-08	0.70	**2.42**
DJ694;2.1,1079(13)	Bg2;2.1,1079(14)	10-Feb-08	0.90	**3.10**
DJ694;2.1,1079(13)	Bg2;2.1,1079(13)	19-Dec-07	0.39	**3.15**
DJ694;2.1,1079(13)	Bg2;2.1,1079(13)	19-Dec-07	0.49	**3.95**
DJ694;2.1,1079(13)	Bg2;2.1,1079(14)	19-Dec-07	0.60	**4.83**
DJ694;2.1,1079(13)	Bg2;2.1,1079(14)	19-Dec-07	0.73	**5.93**
DJ694;2.1,1079(14)	Bg2;2.1,1079(13)	19-Dec-07	1.38	**11.15**
DJ694;2.1,1079(14)	Bg2;2.1,1079(13)	19-Dec-07	1.35	**10.96**
DJ694;2.1,1079(14)	Bg2;2.1,1079(13)	19-Dec-07	1.35	**10.94**
DJ694;2.1,1079(14)	Bg2;2.1,1079(14)	19-Dec-07	2.46	**19.93**
DJ694;2.1,1079(14)	Bg2;2.1,1079(14)	19-Dec-07	2.58	**20.90**
DJ694;2.1,1079(14)	Bg2;2.1,1079(14)	19-Dec-07	2.52	**20.42**
DJ694;2.1,1079(14)	Bg2;2.1,1079(14)	19-Dec-07	0.78	**6.35**
DJ694;2.1,1079(14)	Bg2;2.1,1079(14)	19-Dec-07	0.91	**7.33**
DJ694;2.1,1079(14)	Bg2;2.1,1079(14)	19-Dec-07	0.53	**4.30**
DJ694;2.1,1079(14)	Bg2;2.1,1079(13)	10-Feb-08	3.23	**11.13**
DJ694;2.1,1079(14)	Bg2;2.1,1079(13)	10-Feb-08	3.07	**10.58**
DJ694;2.1,1079(14)	Bg2;2.1,1079(13)	10-Feb-08	3.89	**13.41**
DJ694;2.1,1079(14)	Bg2;2.1,1079(14)	10-Feb-08	1.60	**5.50**
DJ694;2.1,1079(14)	Bg2;2.1,1079(14)	10-Feb-08	3.50	**12.05**
DJ694;2.1,1079(14)	Bg2;2.1,1079(14)	10-Feb-08	1.60	**5.53**
DJ694;2.1,1079(13)	Bg2;2.1,1079(13)	19-Dec-07	1.03	8.33
DJ694;2.1,1079(13)	Bg2;2.1,1079(14)	19-Dec-07	3.16	25.56
			**DJ694 + 2.1 + 3.3 + 2 copies DJ1079**	
DJ694;2.1,1079(13)	Bg2;3.3,1079	19-Dec-07	1.25	**10.09**

Late pupae

Parents		Date	Specific activity ΔmOD/min/µg protein	% DJ694 control
**Male**	**Female**			
DJ694;2.1,1079(13)	Bg2;3.3,1079	19-Dec-07	1.02	**8.27**
DJ694;2.1,1079(14)	Bg2;3.3,1079	19-Dec-07	3.31	**26.77**
DJ694;2.1,1079(14)	Bg2;3.3,1079	19-Dec-07	3.52	**28.46**
DJ694;2.1,1079(14)	Bg2;3.3,1079	19-Dec-07	2.90	**23.49**
DJ694;2.1,1079(14)	Bg2;3.3,1079	19-Dec-07	0.67	**5.45**
DJ694;2.1,1079(14)	Bg2;3.3,1079	19-Dec-07	0.43	**3.45**
DJ694;2.1,1079(14)	Bg2;3.3,1079	19-Dec-07	0.57	**4.61**
DJ694;2.1,1079(13)	Bg2;3.3,1079	10-Feb-08	0.73	**2.51**
DJ694;2.1,1079(13)	Bg2;3.3,1079	10-Feb-08	1.25	**4.31**
DJ694;2.1,1079(13)	Bg2;3.3,1079	10-Feb-08	1.02	**3.51**
DJ694;2.1,1079(14)	Bg2;3.3,1079	10-Feb-08	2.87	**9.88**
DJ694;2.1,1079(14)	Bg2;3.3,1079	10-Feb-08	1.92	**6.62**
DJ694;2.1,1079(14)	Bg2;3.3,1079	10-Feb-08	1.76	**6.05**
DJ694;2.1,1079(13)	Bg2;3.3,1079	19-Dec-07	0.48	3.89
			**DJ694 + 2 copies 3.3 + 2 copies DJ1077**	
DJ694;3.3,1077	Bg2;3.3,1077	13-Nov-07	0.20	**2.49**
DJ694;3.3,1077	Bg2;3.3,1077	13-Nov-07	0.16	**1.96**
DJ694;3.3,1077	Bg2;3.3,1077	13-Nov-07	0.13	**1.60**
DJ694;3.3,1077	Bg2;3.3,1077	30-Nov-07	0.15	**1.46**
DJ694;3.3,1077	Bg2;3.3,1077	30-Nov-07	0.24	**2.33**
DJ694;3.3,1077	Bg2;3.3,1077	30-Nov-07	0.29	**2.91**
DJ694;3.3,1077	Bg2;3.3,1077	19-Dec-07	1.19	**9.62**
DJ694;3.3,1077	Bg2;3.3,1077	19-Dec-07	1.21	**9.79**
DJ694;3.3,1077	Bg2;3.3,1077	19-Dec-07	1.07	**8.63**
DJ694;3.3,1077	Bg2;3.3,1077	19-Dec-07	0.66	**5.33**
DJ694;3.3,1077	Bg2;3.3,1077	19-Dec-07	1.08	**8.71**
DJ694;3.3,1077	Bg2;3.3,1077	10-Feb-08	1.04	**3.57**
DJ694;3.3,1077	Bg2;3.3,1077	10-Feb-08	1.06	**3.66**
DJ694;3.3,1077	Bg2;3.3,1077	10-Feb-08	0.95	**3.29**
DJ694;3.3,1077	Bg2;3.3,1077	22-Feb-08	0.49	**2.57**
DJ694;3.3,1077	Bg2;3.3,1077	22-Feb-08	0.61	**3.20**
DJ694;3.3,1077	Bg2;3.3,1077	22-Feb-08	0.55	**2.91**
DJ694;3.3,1077	Bg2;3.3,1077	19-Dec-07	0.60	4.81
			**DJ694 + 2 copies 3.3 + DJ1077 + DJ1079**	
DJ694;3.3,1077	Bg2;3.3,1079	13-Nov-07	0.28	**3.53**
DJ694;3.3,1077	Bg2;3.3,1079	13-Nov-07	0.28	**3.57**
DJ694;3.3,1077	Bg2;3.3,1079	13-Nov-07	0.38	**4.77**
DJ694;3.3,1077	Bg2;3.3,1079	30-Nov-07	0.36	**3.56**
DJ694;3.3,1077	Bg2;3.3,1079	30-Nov-07	0.30	**3.00**
DJ694;3.3,1077	Bg2;3.3,1079	30-Nov-07	0.38	**3.78**

Late pupae

Parents		Date	Specific activity ΔmOD/min/µg protein	% DJ694 control
Male	Female			
DJ694;3.3,1077	Bg2;3.3,1079	19-Dec-07	0.96	**7.80**
DJ694;3.3,1077	Bg2;3.3,1079	19-Dec-07	1.71	**13.86**
DJ694;3.3,1077	Bg2;3.3,1079	19-Dec-07	1.23	**9.99**
DJ694;3.3,1077	Bg2;3.3,1079	19-Dec-07	1.72	**13.95**
DJ694;3.3,1077	Bg2;3.3,1079	19-Dec-07	1.16	**9.39**
DJ694;3.3,1077	Bg2;3.3,1079	19-Dec-07	1.13	**9.10**
DJ694;3.3,1077	Bg2;3.3,1079	10-Feb-08	0.55	**1.91**
DJ694;3.3,1077	Bg2;3.3,1079	10-Feb-08	0.67	**2.31**
DJ694;3.3,1077	Bg2;3.3,1079	10-Feb-08	0.74	**2.54**
			**DJ694 + 2.1 + 3.3 + DJ1077 + DJ1080**	
DJ694;1077,1080	Bg2;2.1,3.3	15-Jun-07	2.09	**9.89**
DJ694;1077,1080	Bg2;2.1,3.3	15-Jun-07	1.40	**6.61**
DJ694;1077,1080	Bg2;2.1,3.3	15-Jun-07	3.62	**17.15**
DJ694;1077,1080	Bg2;2.1,3.3	04-Jul-07	0.34	**4.35**
DJ694;1077,1080	Bg2;2.1,3.3	04-Jul-07	1.27	**16.35**
DJ694;1077,1080	Bg2;2.1,3.3	04-Jul-07	0.48	**6.16**
DJ694;2.1,3.3	Bg2;1077,1080	31-May-07	0.97	**2.25**
DJ694;2.1,3.3	Bg2;1077,1080	31-May-07	0.69	**1.61**
DJ694;2.1,3.3	Bg2;1077,1080	31-May-07	0.81	**1.88**
DJ694;2.1,3.3	Bg2;1077,1080	31-May-07	0.25	**0.57**
			**DJ694 + 2.1 + 3.3 + DJ1080 + DJ1083**	
DJ694;1080,1083	Bg2;2.1,3.3	31-May-07	1.67	**3.88**
DJ694;1080,1083	Bg2;2.1,3.3	31-May-07	4.55	**10.56**
DJ694;1080,1083	Bg2;2.1,3.3	31-May-07	3.61	**8.39**
DJ694;1080,1083	Bg2;2.1,3.3	31-May-07	2.02	**4.70**
			**DJ694 + 2.1 + 2 copies DJ1077 + DJ1079**	
DJ694;1077,1079	Bg2;2.1,1077	19-Dec-07	0.63	**5.11**
DJ694;1077,1079	Bg2;2.1,1077	19-Dec-07	0.56	**4.55**
DJ694;1077,1079	Bg2;2.1,1077	19-Dec-07	0.51	**4.11**
			**DJ694 + 2.1 + DJ1077 + 2 copies DJ1079**	
DJ694;1077,1079	Bg2;2.1,1079(14)	19-Dec-07	0.89	**7.20**
DJ694;1077,1079	Bg2;2.1,1079(14)	19-Dec-07	1.17	**9.43**
DJ694;1077,1079	Bg2;2.1,1079(14)	19-Dec-07	1.83	**14.80**
			**DJ694 + 3.3 + 2 copies DJ1077 + DJ1079**	
DJ694;1077,1079	Bg2;3.3,1077	19-Dec-07	0.87	**7.07**
DJ694;1077,1079	Bg2;3.3,1077	19-Dec-07	0.62	**4.98**
DJ694;1077,1079	Bg2;3.3,1077	19-Dec-07	0.68	**5.48**
			**DJ694 + 3.3 + DJ1077 + 2 copies DJ1079**	
DJ694;1077,1079	Bg2;3.3,1079	19-Dec-07	1.67	**13.55**

Late pupae

Parents		Date	Specific activity ΔmOD/min/μg protein	% DJ694 control
Male	Female			
DJ694;1077,1079	Bg2;3.3,1079	19-Dec-07	1.11	**8.96**
DJ694;1077,1079	Bg2;3.3,1079	19-Dec-07	1.40	**11.34**
			**DJ694 + 2.1 + 2 copies DJ1077 + DJ1080**	
DJ694;1077,1080	Bg2;2.1,1077	10-Feb-08	1.95	**6.73**
DJ694;1077,1080	Bg2;2.1,1077	10-Feb-08	2.90	**9.98**
DJ694;1077,1080	Bg2;2.1,1077	10-Feb-08	2.29	**7.91**
			**DJ694 + 2.1 + DJ1077 + DJ1079 + DJ1080**	
DJ694;1077,1080	Bg2;2.1,1079(13)	10-Feb-08	2.63	**9.06**
DJ694;1077,1080	Bg2;2.1,1079(13)	10-Feb-08	2.40	**8.26**
DJ694;1077,1080	Bg2;2.1,1079(13)	10-Feb-08	1.31	**4.51**
DJ694;1077,1080	Bg2;2.1,1079(14)	10-Feb-08	3.37	**11.61**
DJ694;1077,1080	Bg2;2.1,1079(14)	10-Feb-08	2.76	**9.53**
DJ694;1077,1080	Bg2;2.1,1079(14)	10-Feb-08	5.85	**20.15**
DJ694;2.1,1079(13)	Bg2;1077,1080	10-Feb-08	0.58	**2.00**
DJ694;2.1,1079(13)	Bg2;1077,1080	10-Feb-08	0.47	**1.62**
DJ694;2.1,1079(13)	Bg2;1077,1080	10-Feb-08	0.41	**1.40**
DJ694;2.1,1079(14)	Bg2;1077,1080	10-Feb-08	1.12	**3.85**
DJ694;2.1,1079(14)	Bg2;1077,1080	10-Feb-08	2.61	**8.99**
DJ694;2.1,1079(14)	Bg2;1077,1080	10-Feb-08	1.16	**4.01**
			**DJ694 + 3.3 + 2 copies DJ1077 + DJ1080**	
DJ694;1077,1080	Bg2;3.3,1077	10-Feb-08	3.05	**10.51**
DJ694;1077,1080	Bg2;3.3,1077	10-Feb-08	3.12	**10.75**
DJ694;1077,1080	Bg2;3.3,1077	10-Feb-08	3.75	**12.91**
DJ694;3.3,1077	Bg2;1077,1080	10-Feb-08	1.48	**5.09**
DJ694;3.3,1077	Bg2;1077,1080	10-Feb-08	1.20	**4.15**
DJ694;3.3,1077	Bg2;1077,1080	10-Feb-08	1.25	**4.31**
			**DJ694 + 3.3 + DJ1077 + DJ1079 + DJ1080**	
DJ694;1077,1080	Bg2;3.3,1079	10-Feb-08	1.23	**4.22**
DJ694;1077,1080	Bg2;3.3,1079	10-Feb-08	4.76	**16.40**
DJ694;1077,1080	Bg2;3.3,1079	10-Feb-08	3.49	**12.01**
			**DJ694 + 2.1 + 2 copies DJ1077 + DJ1080**	
DJ694;2.1,1077	Bg2;1077,1080	10-Feb-08	2.38	**8.20**
DJ694;2.1,1077	Bg2;1077,1080	10-Feb-08	0.84	**2.89**
DJ694;2.1,1077	Bg2;1077,1080	10-Feb-08	0.65	**2.24**
			**DJ694 + 2.1 + DJ1077 + DJ1080 + DJ1083**	
DJ694;2.1,1077	Bg2;1080,1083	10-Feb-08	1.97	**6.80**
DJ694;2.1,1077	Bg2;1080,1083	10-Feb-08	4.21	**14.51**
DJ694;2.1,1077	Bg2;1080,1083	10-Feb-08	3.60	**12.42**
			**DJ694 + 2.1 + DJ1079 + DJ1080 + DJ1083**	

Late pupae

Parents		Date	Specific activity ΔmOD/min/μg protein	% DJ694 control
Male	Female			
DJ694;2.1,1079(13)	Bg2;1080,1083	10-Feb-08	1.19	**4.10**
DJ694;2.1,1079(13)	Bg2;1080,1083	10-Feb-08	1.12	**3.87**
DJ694;2.1,1079(13)	Bg2;1080,1083	10-Feb-08	0.81	**2.79**
DJ694;2.1,1079(14)	Bg2;1080,1083	10-Feb-08	1.46	**5.04**
DJ694;2.1,1079(14)	Bg2;1080,1083	10-Feb-08	1.75	**6.03**
DJ694;2.1,1079(14)	Bg2;1080,1083	10-Feb-08	1.55	**5.35**
			**DJ694 + 3.3 + DJ1077 + DJ1080 + DJ1083**	
DJ694;3.3,1077	Bg2;1080,1083	10-Feb-08	1.45	**4.99**
DJ694;3.3,1077	Bg2;1080,1083	10-Feb-08	1.72	**5.94**
DJ694;3.3,1077	Bg2;1080,1083	10-Feb-08	1.34	**4.62**

Supplemental Table 8: UAS-lacZ assay statistical analysis (t-tests).

	L1	L3	EP	LP
**1 copy 146 compared to:**				
2 copy 146	0.07009452	0.10739306	0.34418726	0.09753996
3 copy 146	4.6455E-19	0.00299309	9.4825E-17	1.0518E-06
4 copy 146	2.9076E-17	1.6604E-11	1.4828E-14	0.0596847
1 copy 147	0.32952063	0.03752734	8.3178E-05	0.35191187
2 copy 147	0.00863281	0.00040376	0.67111125	0.03671816
3 copy 147	1.6585E-06	0.00280185	0.85102789	0.00149374
1 copy 146 + 1 copy 147	2.9931E-09	4.4585E-11	2.6056E-09	0.0002597
2 copy 146 + 1 copy 147	4.2565E-23	0.01315022	6.9832E-18	3.1082E-16
1 copy 146 + 2 copy 147	1.2546E-10	0.00056488	1.6271E-13	3.6108E-17
2 copy 146 + 2 copy 147	3.4105E-48	5.0396E-39	8.6409E-61	2.23E-63
3 copy 146 + 1 copy 147	7.2153E-26	6.7846E-23	4.6195E-28	1.2061E-24
**2 copy 146 compared to:**				
3 copy 146	7.1657E-09	4.6915E-06	3.2099E-12	6.6773E-11
4 copy 146	4.0231E-08	2.8557E-12	2.0745E-11	0.00040294
1 copy 147	0.01234006	0.73902451	3.2102E-05	0.41341207
2 copy 147	0.45744807	0.0167807	0.84181842	0.4531964
3 copy 147	0.05051782	0.0240573	0.52180499	1.3538E-05
1 copy 146 + 1 copy 147	0.00022545	3.5655E-13	1.7123E-07	4.8933E-08
2 copy 146 + 1 copy 147	7.6965E-11	2.8247E-05	6.5125E-15	4.85E-21
1 copy 146 + 2 copy 147	0.00030284	2.237E-06	5.0833E-10	2.0492E-19
2 copy 146 + 2 copy 147	9.9955E-33	1.6013E-57	3.2725E-61	4.2773E-87
3 copy 146 + 1 copy 147	1.2209E-13	1.1666E-23	6.6905E-23	3.2349E-28
**3 copy 146 compared to:**				
4 copy 146	0.81325587	0.00030482	0.04852396	0.00189258
1 copy 147	3.6801E-18	3.3303E-06	8.03E-28	1.5244E-09
2 copy 147	3.5016E-13	2.4906E-06	1.7638E-08	2.9546E-10
3 copy 147	1.4596E-14	1.9257E-07	9.642E-12	0.51507179
1 copy 146 + 1 copy 147	5.0394E-05	0.00475991	0.07252262	0.66472621
2 copy 146 + 1 copy 147	0.26930174	0.69334129	0.09854234	1.2274E-11
1 copy 146 + 2 copy 147	2.4569E-07	0.52554701	0.90303297	1.6366E-18
2 copy 146 + 2 copy 147	0.45176244	6.7247E-20	2.9975E-14	4.8574E-53
3 copy 146 + 1 copy 147	0.98055004	6.0319E-11	3.379E-10	1.4256E-24
**4 copy 146 compared to:**				
1 copy 147	1.6902E-16	2.3785E-14	8.5839E-23	0.00349934
2 copy 147	8.5856E-11	5.1036E-12	2.7773E-07	0.00032519
3 copy 147	1.2326E-12	7.9843E-10	1.7117E-10	0.07397915
1 copy 146 + 1 copy 147	0.00012147	0.01479651	0.00459995	0.01717501
2 copy 146 + 1 copy 147	0.26963059	0.00023312	0.6823459	9.8385E-14
1 copy 146 + 2 copy 147	1.4739E-06	0.00230957	0.10956928	1.3217E-16
2 copy 146 + 2 copy 147	0.42723063	0.13858641	4.94E-05	1.0122E-55

3 copy 146 + 1 copy 147	0.92776288	0.00027873	0.00072429	3.2476E-23
**1 copy 147 compared to:**				
2 copy 147	0.00473671	0.01568184	0.00172565	0.11259027
3 copy 147	5.9372E-07	0.06222065	0.00144373	4.2483E-05
1 copy 146 + 1 copy 147	1.9962E-10	2.429E-15	3.5761E-17	7.678E-06
2 copy 146 + 1 copy 147	3.0595E-22	3.0321E-05	4.8528E-28	1.5179E-19
1 copy 146 + 2 copy 147	5.3858E-11	8.6368E-07	1.6669E-22	5.4275E-20
2 copy 146 + 2 copy 147	2.1261E-53	6.42E-55	8.0643E-89	2.0556E-73
3 copy 146 + 1 copy 147	8.6345E-26	7.0678E-27	3.2865E-40	1.2688E-28
**2 copy 147 compared to:**				
3 copy 147	0.29119495	0.71474846	0.84426375	4.962E-06
1 copy 146 + 1 copy 147	0.11936543	6.8958E-14	0.00072283	7.4976E-05
2 copy 146 + 1 copy 147	1.0584E-13	1.4218E-05	3.6185E-08	3.4694E-17
1 copy 146 + 2 copy 147	0.01054415	1.8137E-06	1.1094E-06	5.7321E-22
2 copy 146 + 2 copy 147	3.0905E-08	1.6365E-42	1.9428E-23	5.6081E-52
3 copy 146 + 1 copy 147	7.5198E-10	1.3268E-22	1.284E-15	2.9619E-29
**3 copy 147 compared to:**				
1 copy 146 + 1 copy 147	0.13933676	1.2413E-11	4.298E-07	0.49275503
2 copy 146 + 1 copy 147	6.6598E-16	4.3376E-07	1.4387E-13	2.8494E-08
1 copy 146 + 2 copy 147	0.01109755	6.609E-07	1.5795E-09	3.0288E-13
2 copy 146 + 2 copy 147	4.7957E-13	1.8203E-55	9.428E-52	6.4547E-34
3 copy 146 + 1 copy 147	1.3336E-13	1.6916E-18	7.7168E-21	1.0222E-17
**1 copy 146 + 1 copy 147 compared to:**				
2 copy 146 + 1 copy 147	1.0799E-05	0.002266	0.0028108	1.7684E-05
1 copy 146 + 2 copy 147	0.63026461	0.04068154	0.10060908	5.5346E-08
2 copy 146 + 2 copy 147	1.972E-10	2.6921E-07	7.6157E-21	2.3567E-33
3 copy 146 + 1 copy 147	5.3612E-07	1.2909E-10	5.5185E-10	2.9372E-11
**2 copy 146 + 1 copy 147 compared to:**				
1 copy 146 + 2 copy 147	7.9679E-08	0.33360573	0.18568044	4.9253E-05
2 copy 146 + 2 copy 147	0.71984686	1.3848E-22	1.3371E-08	1.8162E-11
3 copy 146 + 1 copy 147	0.5020725	6.8156E-11	3.4079E-05	9.0616E-06
**1 copy 146 + 2 copy 147 compared to:**				
2 copy 146 + 2 copy 147	8.4526E-07	4.5666E-13	5.4463E-11	0.8735066
3 copy 146 + 1 copy 147	1.3347E-07	3.8672E-09	7.3415E-08	0.48343005
**2 copy 146 + 2 copy 147 compared to:**				
3 copy 146 + 1 copy 147	0.30992956	0.00709331	0.86283639	0.29785406

Supplemental Table 9. Repression ability of 2x146 + 2x147 in adults fed through life. Significant p-values (>0.05) from comparison between experimental and negative or positive controls are bolded. Complete repression and total absence of repression result in unsignificant difference with the negative control and positive control respectively.

Age (days)	Tetracycline (μg/ml)	vs negative control ♂	vs negative control ♀	vs positive control ♂	vs positive control ♀
2	0	**1.5E-06**	**1.1E-08**	**1.8E-07**	**3.7E-06**
	1	**3.8E-06**	**1.1E-07**	**1.9E-09**	**5.2E-09**
	10	**2.4E-05**	**3.4E-07**	**1.5E-08**	**1.4E-08**
	100	**4.2E-06**	**7.7E-06**	**3.7E-07**	**6.0E-06**
5	0	**9.0E-05**	**2.6E-04**	**7.7E-10**	**1.2E-05**
	1	**8.5E-05**	**7.3E-05**	**5.6E-09**	**2.7E-04**
	10	**3.5E-06**	**2.2E-07**	**9.1E-07**	**8.0E-07**
	100	**6.8E-05**	**8.7E-06**	**1.4E-05**	**2.0E-05**
10	0	**5.2E-06**	**2.4E-07**	**3.2E-12**	**8.0E-08**
	1	**5.4E-06**	**2.9E-07**	**5.9E-07**	**4.3E-03**
	10	**4.0E-04**	**1.5E-06**	**1.8E-05**	**4.3E-04**
	100	**6.8E-08**	**3.3E-09**	**1.5E-06**	**6.1E-04**
20	0	**3.0E-05**	**2.0E-04**	**9.6E-08**	**3.9E-05**
	1	**2.0E-06**	**2.3E-03**	**6.3E-04**	0.86
	10	**1.2E-04**	**4.1E-04**	0.11	0.53
	100	**1.7E-08**	**1.4E-04**	0.52	0.14
30	0	**0.02**	**6.8E-03**	**2.3E-04**	**4.3E-04**
	1	**1.2E-04**	**4.5E-05**	**7.8E-03**	**3.1E-04**
	10	**3.6E-04**	**5.3E-04**	**2.2E-03**	**0.02**
	100	**4.7E-04**	**3.2E-03**	0.70	0.82
40	0	**1.3E-05**	**1.8E-03**	**9.7E-04**	**7.6E-03**
	1	**7.4E-04**	**2.5E-05**	**6.2E-05**	**2.9E-04**
	10	**7.9E-04**	**2.5E-03**	**1.4E-03**	**1.8E-03**
	100	**2.5E-03**	**3.7E-03**	**4.2E-03**	0.07

Supplemental Table 10. Induction abilities of 2x146 + 2x147 in adults fed through life. Significant p-values (<0.05) are bolded. Comparisons and grouping as described in Poirier et al, 2008.

	Compared to	*Tetracycline (µg/ml)*	P values					
			Days of induction					
			2	5	10	20	30	40
**Males**	**0 µg/ml**	1	0.13	**2.2E-03**	**1.1E-05**	**3.4E-06**	0.06	0.18
		10	0.56	**1.9E-05**	**6.5E-04**	**1.8E-04**	0.07	0.15
		100	0.39	**4.2E-04**	**1.2E-07**	**2.6E-08**	**0.01**	0.97
	**1 µg/ml**	10	0.43	**2.1E-03**	0.45	0.76	0.92	0.93
		100	0.50	**0.04**	**0.02**	**0.02**	0.12	0.35
	**10 µg/ml**	100	0.86	0.25	0.41	0.17	0.16	0.32
**Females**	**0 µg/ml**	1	3.8E-04	**9.4E-03**	**3.0E-06**	**0.01**	**0.02**	0.66
		10	0.28	**4.4E-04**	**3.1E-05**	**2.2E-03**	**0.04**	0.70
		100	0.93	**8.7E-04**	**2.8E-07**	**3.7E-03**	0.06	0.94
	**1 µg/ml**	10	**8.2E-03**	0.74	0.04	0.77	0.87	0.96
		100	**0.01**	0.27	3.2E-03	0.21	0.61	0.76
	**10 µg/ml**	100	0.44	0.27	0.38	0.08	0.72	0.77
**Number of inducing concentrations**		**Different levels of induction**	**Grouping**					
**3**		3						
		2		♂	♂	♂		
		1		♀	♀	♀		
**2**		2						
		1					♀	
**1**		1					♂	
**None**		0	♂ ♀					♂ ♀

Supplemental Table 11. Repression ability of 2x146 + 2x147 in adults fed starting at 20 days old. Significant p-values (>0.05) from comparison between experimental and negative or positive controls are bolded. Complete repression and total absence of repression result in unsignificant difference with the negative control and positive control respectively.

**Induction length (days)**	**Tetracycline (μg/ml)**	**vs negative control**		**vs positive control**	
		♂	♀	♂	♀
2	0	**1.8E-07**	**9.7E-09**	**8.0E-07**	**1.6E-06**
	1	**1.0E-03**	**1.0E-05**	**6.7E-09**	**8.3E-05**
	100	**2.3E-06**	**2.9E-05**	**1.2E-05**	**1.1E-06**
5	0	**1.6E-07**	**0.01**	**1.6E-12**	**1.0E-03**
	1	**5.0E-04**	**3.7E-04**	**1.5E-05**	**6.4E-05**
	100	**1.6E-04**	**1.3E-04**	**6.1E-03**	**4.7E-04**
10	0	**5.3E-04**	**1.9E-03**	**3.6E-04**	**9.7E-03**
	1	**1.3E-03**	**3.0E-04**	**6.4E-05**	**1.9E-03**
	100	**3.8E-03**	**3.1E-05**	**1.8E-04**	**8.3E-04**
20	0	**4.5E-04**	**2.6E-06**	**1.4E-04**	**1.0E-07**
	1	**6.8E-05**	**5.0E-06**	**5.4E-03**	**0.03**
	100	**1.8E-03**	**1.9E-04**	**4.9E-06**	**0.02**

Supplemental Table 12. Induction abilities of 2x146 + 2x147 in adults fed starting at 20 days old. Significant p-values (<0.05) are bolded. Comparisons and grouping as described in Poirier et al, 2008.

	**Compared to**	***Tetracycline (µg/ml)***	**P values**			
			Days of induction			
			2	5	10	20
**Males**	**0 µg/ml**	1	0.76	**0.02**	0.06	**8.4E-03**
		100	0.28	**0.01**	0.15	0.71
	**1 µg/ml**	100	0.82	0.82	0.74	**6.6E-03**
**Females**	**0 µg/ml**	1	0.18	0.20	0.40	**2.0E-04**
		100	0.63	0.13	**0.04**	**0.01**
	**1 µg/ml**	100	0.50	0.82	0.14	0.16
**Number of inducing concentrations**		**Different levels of induction**	**Grouping**			
**2**		2				
		1		♂		♀
**1**		1			♀	♂
**None**		0	♂ ♀	♀	♂	

Supplemental Table 13: GAL4 driver survey data set.

%: percentage of individuals remaining from previous stage; Plate#. Exp#: plate number and experiment number; SD: standard deviation.

Parents		Plate #.	%L1	SD	%P	SD	%A	SD	%L1	SD	%P	SD	%A	SD
Male	Female	Exp #	AMP						AMP/TET					
			**da-GAL4 + 0 GAL80**											
da-GAL4	UAS-grim	1.1	0.0		0.0		0.0		0.0		0.0		0.0	
		2.1	0.0		0.0		0.0		0.0		0.0		0.0	
		3.1	0.0		0.0		0.0		0.0		0.0		0.0	
		4.1	0.0		0.0		0.0		0.0		0.0		0.0	
		1.2	0.0		0.0		0.0		0.0		0.0		0.0	
		2.2	0.0		0.0		0.0		0.0		0.0		0.0	
		3.2	0.0		0.0		0.0		0.0		0.0		0.0	
		4.2	0.0		0.0		0.0		0.0		0.0		0.0	
			0.0	0.0	0.0	0.0	0.0	0.0	0.0	0.0	0.0	0.0	0.0	0.0
			**0 GAL80**											
da-GAL4	w	1.1	96.0		66.7		93.8		88.0		54.6		91.7	
		2.1	96.0		70.9		100.0		84.0		71.5		93.4	
		3.1	92.0		69.6		100.0		64.0		100.0		100.0	
		4.1	64.0		68.8		91.0		60.0		46.7		85.8	
		1.2	96.0		79.2		94.8		96.0		75.0		100.0	
		2.2	88.0		77.3		88.3		100.0		92.0		100.0	
		3.2	100.0		84.0		100.0		100.0		96.0		100.0	
		4.2	100.0		92.0		100.0		92.0		87.0		100.0	
			91.5	11.8	76.1	8.8	96.0	4.7	85.5	15.6	77.9	19.5	96.4	5.5
			**da-GAL4 + DJ1077**											
da-GAL4	UAS-grim;DJ1077	1.1	0.0		0.0		0.0		0.0		0.0		0.0	
		2.1	0.0		0.0		0.0		0.0		0.0		0.0	
		3.1	0.0		0.0		0.0		0.0		0.0		0.0	
		4.1	0.0		0.0		0.0		0.0		0.0		0.0	
			0.0	0.0	0.0	0.0	0.0	0.0	0.0	0.0	0.0	0.0	0.0	0.0
			**da-GAL4 + 3.3**											
da-GAL4	UAS-grim;3.3	1.1	0.0		0.0		0.0		0.0		0.0		0.0	
		2.1	0.0		0.0		0.0		0.0		0.0		0.0	
		3.1	0.0		0.0		0.0		0.0		0.0		0.0	
		4.1	0.0		0.0		0.0		0.0		0.0		0.0	
			0.0	0.0	0.0	0.0	0.0	0.0	0.0	0.0	0.0	0.0	0.0	0.0
			**da-GAL4 + 1X146 + 1X147**											
da-GAL4	UAS-grim;2.1+DJ1077	1.1	0.0		0.0		0.0		0.0		0.0		0.0	
		2.1	0.0		0.0		0.0		0.0		0.0		0.0	
		3.1	0.0		0.0		0.0		0.0		0.0		0.0	
		4.1	0.0		0.0		0.0		0.0		0.0		0.0	
da-GAL4	UAS-grim;2.1+DJ1079	1.2	0.0		0.0		0.0		0.0		0.0		0.0	
		2.2	0.0		0.0		0.0		0.0		0.0		0.0	
		3.2	0.0		0.0		0.0		0.0		0.0		0.0	
		4.2	0.0		0.0		0.0		0.0		0.0		0.0	
			0.0	0.0	0.0	0.0	0.0	0.0	0.0	0.0	0.0	0.0	0.0	0.0
			**tubGAL4 + 0 GAL80**											
tubGAL4	UAS-grim	1.1	0.0		0.0		0.0		0.0		0.0		0.0	
		2.1	0.0		0.0		0.0		0.0		0.0		0.0	
		3.1	0.0		0.0		0.0		0.0		0.0		0.0	
		4.1	0.0		0.0		0.0		0.0		0.0		0.0	
			0.0	0.0	0.0	0.0	0.0	0.0	0.0	0.0	0.0	0.0	0.0	0.0
			**0 GAL80**											
tubGAL4	w	1.1	92.0		91.4		100.0		100.0		96.0		95.9	
		2.1	100.0		92.0		95.7		96.0		83.4		100.0	
		3.1	100.0		72.0		94.5		92.0		91.4		100.0	
		4.1	96.0		95.9		95.7		96.0		100.0		100.0	
		1.2	100.0		100.0		100.0		96.0		100.0		95.9	
		2.2	100.0		92.0		95.7		96.0		95.9		100.0	
		3.2	100.0		84.0		100.0		92.0		95.7		100.0	
		4.2	100.0		84.0		100.0		84.0		85.8		100.0	
			97.0	3.8	87.8	10.7	96.5	2.4	96.0	3.3	92.7	7.1	99.0	2.1
			**tubGAL4 + DJ1077**											
tubGAL4	UAS-grim;DJ1077	1.1	0.0		0.0		0.0		0.0		0.0		0.0	
		2.1	0.0		0.0		0.0		0.0		0.0		0.0	
		3.1	0.0		0.0		0.0		0.0		0.0		0.0	
		4.1	0.0		0.0		0.0		0.0		0.0		0.0	
			0.0	0.0	0.0	0.0	0.0	0.0	0.0	0.0	0.0	0.0	0.0	0.0

Parents		Plate #.	%L1	SD	%P	SD	%A	SD	%L1	SD	%P	SD	%A	SD
Male	Female	Exp #	AMP						AMP/TET					
			**tubGAL4 + 3.3**											
tubGAL4	UAS-grim;3.3	1.1	0.0		0.0		0.0		0.0		0.0		0.0	
		2.1	0.0		0.0		0.0		0.0		0.0		0.0	
		3.1	0.0		0.0		0.0		0.0		0.0		0.0	
		4.1	0.0		0.0		0.0		0.0		0.0		0.0	
			0.0	0.0	0.0	0.0	0.0	0.0	0.0	0.0	0.0	0.0	0.0	0.0
			**tubGAL4 + 1X146 + 1X147**											
tubGAL4	UAS-grim;2.1+DJ1077	1.1	20.0		0.0		0.0		16.0		0.0		0.0	
		2.1	8.0		0.0		0.0		4.0		0.0		0.0	
		3.1	8.0		0.0		0.0		0.0		0.0		0.0	
		4.1	0.0		0.0		0.0		8.0		0.0		0.0	
tubGAL4	UAS-grim;2.1+DJ1079	1.2	12.0		0.0		0.0		8.0		0.0		0.0	
		2.2	4.0		0.0		0.0		8.0		0.0		0.0	
		3.2	0.0		0.0		0.0		8.0		0.0		0.0	
		4.2	4.0		0.0		0.0		8.0		0.0		0.0	
			7.0	6.7	0.0	0.0	0.0	0.0	7.5	4.5	0.0	0.0	0.0	0.0
			**DJ634 + 0 GAL80**											
DJ634	UAS-grim	1.1	44.0		0.0		0.0		96.0		0.0		0.0	
		2.1	64.0		0.0		0.0		100.0		0.0		0.0	
		3.1	68.0		0.0		0.0		92.0		0.0		0.0	
		4.1	48.0		0.0		0.0		92.0		0.0		0.0	
		1.2	44.0		0.0		0.0		84.0		0.0		0.0	
		2.2	56.0		0.0		0.0		72.0		0.0		0.0	
		3.2	56.0		0.0		0.0		48.0		0.0		0.0	
		4.2	68.0		0.0		0.0		76.0		0.0		0.0	
			56.0	11.8	0.0	0.0	0.0	0.0	95.0	3.8	0.0	0.0	0.0	0.0
			**0 GAL80**											
DJ634	w	1.1	92.0		69.6		100.0		92.0		100.0		100.0	
		2.1	92.0		60.9		100.0		96.0		95.9		95.7	
		3.1	92.0		65.3		100.0		100.0		92.0		100.0	
		4.1	92.0		91.4		100.0		96.0		91.7		100.0	
		1.2	96.0		79.2		94.8		92.0		100.0		100.0	
		2.2	92.0		87.0		95.0		92.0		100.0		95.7	
		3.2	100.0		96.0		100.0		100.0		88.0		100.0	
		4.2	100.0		96.0		100.0		96.0		95.9		100.0	
			92.0	0.0	71.8	13.5	100.0	0.0	96.0	3.3	94.9	3.9	98.9	2.2
			**DJ634 + DJ1077**											
DJ634	UAS-grim;DJ1077	1.1	64.0		0.0		0.0		72.0		0.0		0.0	
		2.1	72.0		0.0		0.0		88.0		0.0		0.0	
		3.1	84.0		0.0		0.0		76.0		0.0		0.0	
		4.1	64.0		0.0		0.0		88.0		0.0		0.0	
			71.0	9.5	0.0	0.0	0.0	0.0	81.0	8.2	0.0	0.0	0.0	0.0
			**DJ634 + 3.3**											
DJ634	UAS-grim;3.3	1.1	92.0		0.0		0.0		84.0		0.0		0.0	
		2.1	92.0		0.0		0.0		88.0		0.0		0.0	
		3.1	96.0		0.0		0.0		88.0		0.0		0.0	
		4.1	88.0		0.0		0.0		84.0		0.0		0.0	
			92.0	3.3	0.0	0.0	0.0	0.0	86.0	2.3	0.0	0.0	0.0	0.0
			**DJ634 + 1X146 + 1X147**											
DJ634	UAS-grim;2.1+DJ1077	1.1	32.0		12.5		0.0		68.0		0.0		0.0	
		2.1	64.0		0.0		0.0		68.0		0.0		0.0	
		3.1	52.0		0.0		0.0		60.0		6.7		0.0	
		4.1	68.0		0.0		0.0		68.0		0.0		0.0	
DJ634	UAS-grim;2.1+DJ1079	1.2	52.0		7.7		0.0		80.0		0.0		0.0	
		2.2	56.0		14.3		0.0		56.0		0.0		0.0	
		3.2	68.0		0.0		0.0		72.0		0.0		0.0	
		4.2	52.0		23.1		0.0		80.0		0.0		0.0	
			55.5	11.8	7.2	8.8	0.0	0.0	69.0	8.5	0.8	2.4	0.0	0.0
			**D42 + 0 GAL80**											
D42	UAS-grim	1.1	0.0		0.0		0.0		0.0		0.0		0.0	
		2.1	0.0		0.0		0.0		0.0		0.0		0.0	
		3.1	0.0		0.0		0.0		0.0		0.0		0.0	
		4.1	0.0		0.0		0.0		0.0		0.0		0.0	
		1.2	8.0		0.0		0.0		0.0		0.0		0.0	
		2.2	8.0		0.0		0.0		0.0		0.0		0.0	
		3.2	12.0		0.0		0.0		0.0		0.0		0.0	
		4.2	8.0		0.0		0.0		0.0		0.0		0.0	
			4.5	5.0	0.0	0.0	0.0	0.0	0.0	0.0	0.0	0.0	0.0	0.0

Parents		Plate #.	%L1	SD	%P	SD	%A	SD	%L1	SD	%P	SD	%A	SD
Male	Female	Exp #	AMP						AMP/TET					
			**0 GAL80**											
D42	w	1.1	96.0		33.4		100.0		100.0		60.0		100.0	
		2.1	76.0		79.0		100.0		100.0		80.0		100.0	
		3.1	76.0		68.5		92.4		96.0		91.7		100.0	
		4.1	84.0		66.7		100.0		100.0		92.0		100.0	
		1.2	100.0		80.0		100.0		100.0		96.0		91.7	
		2.2	100.0		92.0		100.0		100.0		80.0		100.0	
		3.2	100.0		88.0		100.0		100.0		100.0		100.0	
		4.2	100.0		72.0		94.5		100.0		100.0		100.0	
			91.5	11.0	72.5	18.1	98.4	3.1	99.5	1.4	87.5	13.6	99.0	2.9
			**D42 + DJ1077**											
D42	UAS-grim;DJ1077	1.1	8.0		0.0		0.0		52.0		0.0		0.0	
		2.1	20.0		0.0		0.0		60.0		0.0		0.0	
		3.1	24.0		0.0		0.0		60.0		0.0		0.0	
		4.1	40.0		0.0		0.0		60.0		0.0		0.0	
			23.0	13.2	0.0	0.0	0.0	0.0	58.0	4.0	0.0	0.0	0.0	0.0
			**D42 + 3.3**											
D42	UAS-grim;3.3	1.1	0.0		0.0		0.0		0.0		0.0		0.0	
		2.1	0.0		0.0		0.0		0.0		0.0		0.0	
		3.1	0.0		0.0		0.0		0.0		0.0		0.0	
		4.1	0.0		0.0		0.0		0.0		0.0		0.0	
			0.0	0.0	0.0	0.0	0.0	0.0	0.0	0.0	0.0	0.0	0.0	0.0
			**D42 + 1X146 + 1X147**											
D42	UAS-grim;2.1+DJ1077	1.1	40.0		30.0		0.0		52.0		15.4		0.0	
		2.1	44.0		27.3		0.0		36.0		22.3		0.0	
		3.1	40.0		10.0		0.0		36.0		0.0		0.0	
		4.1	36.0		0.0		0.0		20.0		0.0		0.0	
D42	UAS-grim;2.1+DJ1079	1.2	40.0		40.0		0.0		52.0		7.7		0.0	
		2.2	56.0		42.9		0.0		84.0		9.6		0.0	
		3.2	56.0		21.5		0.0		64.0		6.3		0.0	
		4.2	48.0		16.7		0.0		48.0		8.4		0.0	
			45.0	7.6	23.6	14.6	0.0	0.0	49.0	19.4	8.7	7.5	0.0	0.0
			**24B + 0 GAL80**											
24B	UAS-grim	1.1	0.0		0.0		0.0		0.0		0.0		0.0	
		2.1	0.0		0.0		0.0		0.0		0.0		0.0	
		3.1	0.0		0.0		0.0		0.0		0.0		0.0	
		4.1	0.0		0.0		0.0		0.0		0.0		0.0	
		1.2	0.0		0.0		0.0		0.0		0.0		0.0	
		2.2	0.0		0.0		0.0		0.0		0.0		0.0	
		3.2	0.0		0.0		0.0		0.0		0.0		0.0	
		4.2	0.0		0.0		0.0		0.0		0.0		0.0	
			0.0	0.0	0.0	0.0	0.0	0.0	0.0	0.0	0.0	0.0	0.0	0.0
			**0 GAL80**											
24B	w	1.1	100.0		92.0		100.0		96.0		100.0		100.0	
		2.1	100.0		84.0		95.3		100.0		100.0		96.0	
		3.1	100.0		84.0		100.0		100.0		92.0		100.0	
		4.1	96.0		100.0		100.0		96.0		91.7		100.0	
		1.2	96.0		100.0		100.0		96.0		100.0		100.0	
		2.2	96.0		87.5		100.0		100.0		100.0		100.0	
		3.2	96.0		91.7		100.0		100.0		92.0		100.0	
		4.2	96.0		83.4		100.0		96.0		95.9		95.7	
			97.5	2.1	90.3	6.8	99.4	1.7	98.0	2.1	96.5	4.0	99.0	1.9
			**24B + DJ1077**											
24B	UAS-grim;DJ1077	1.1	0.0		0.0		0.0		0.0		0.0		0.0	
		2.1	0.0		0.0		0.0		0.0		0.0		0.0	
		3.1	0.0		0.0		0.0		0.0		0.0		0.0	
		4.1	0.0		0.0		0.0		0.0		0.0		0.0	
			0.0	0.0	0.0	0.0	0.0	0.0	0.0	0.0	0.0	0.0	0.0	0.0
			**24B + 3.3**											
24B	UAS-grim;3.3	1.1	0.0		0.0		0.0		0.0		0.0		0.0	
		2.1	0.0		0.0		0.0		0.0		0.0		0.0	
		3.1	0.0		0.0		0.0		0.0		0.0		0.0	
		4.1	0.0		0.0		0.0		0.0		0.0		0.0	
			0.0	0.0	0.0	0.0	0.0	0.0	0.0	0.0	0.0	0.0	0.0	0.0

Parents		Plate #.	%L1	SD	%P	SD	%A	SD	%L1	SD	%P	SD	%A	SD
Male	Female	Exp #	AMP						AMP/TET					
			**24B + 1X146 + 1X147**											
		1.1	32.0		0.0		0.0		8.0		0.0		0.0	
24B	UAS-grim;2.1+DJ1077	2.1	20.0		0.0		0.0		12.0		0.0		0.0	
		3.1	36.0		0.0		0.0		12.0		0.0		0.0	
		4.1	44.0		0.0		0.0		12.0		0.0		0.0	
		1.2	40.0		0.0		0.0		20.0		0.0		0.0	
24B	UAS-grim;2.1+DJ1079	2.2	36.0		0.0		0.0		20.0		0.0		0.0	
		3.2	24.0		0.0		0.0		32.0		0.0		0.0	
		4.2	16.0		0.0		0.0		32.0		0.0		0.0	
			31.0	10.0	0.0	0.0	0.0	0.0	18.5	9.3	0.0	0.0	0.0	0.0
			**ELAV + 0 GAL80**											
		1.1	0.0		0.0		0.0		0.0		0.0		0.0	
ELAV	UAS-grim	2.1	0.0		0.0		0.0		0.0		0.0		0.0	
		3.1	0.0		0.0		0.0		0.0		0.0		0.0	
		4.1	0.0		0.0		0.0		0.0		0.0		0.0	
			0.0	0.0	0.0	0.0	0.0	0.0	0.0	0.0	0.0	0.0	0.0	0.0
			**0 GAL80**											
		1.1	96.0		83.4		100.0		100.0		84.0		100.0	
ELAV	w	2.1	100.0		96.0		100.0		100.0		100.0		100.0	
		3.1	92.0		100.0		95.7		100.0		92.0		100.0	
		4.1	92.0		69.6		81.3		100.0		84.0		100.0	
		1.2	100.0		88.0		100.0		100.0		88.0		100.0	
		2.2	92.0		100.0		100.0		100.0		80.0		100.0	
		3.2	92.0		87.0		100.0		96.0		87.5		100.0	
		4.2	96.0		79.2		100.0		96.0		79.2		100.0	
			95.0	3.5	87.9	10.6	97.1	6.6	99.0	1.9	86.8	6.8	100.0	0.0
			**ELAV + DJ1077**											
		1.1	36.0		0.0		0.0		64.0		0.0		0.0	
ELAV	UAS-grim;DJ1077	2.1	44.0		0.0		0.0		40.0		0.0		0.0	
		3.1	68.0		0.0		0.0		56.0		0.0		0.0	
		4.1	60.0		0.0		0.0		52.0		0.0		0.0	
			52.0	14.6	0.0	0.0	0.0	0.0	53.0	10.0	0.0	0.0	0.0	0.0
			**ELAV + 3.3**											
		1.1	0.0		0.0		0.0		0.0		0.0		0.0	
ELAV	UAS-grim;3.3	2.1	0.0		0.0		0.0		0.0		0.0		0.0	
		3.1	0.0		0.0		0.0		0.0		0.0		0.0	
		4.1	0.0		0.0		0.0		0.0		0.0		0.0	
			0.0	0.0	0.0	0.0	0.0	0.0	0.0	0.0	0.0	0.0	0.0	0.0
			**ELAV + 1X146 + 1X147**											
		1.1	80.0		0.0		0.0		68.0		0.0		0.0	
ELAV	UAS-grim;2.1+DJ1077	2.1	68.0		0.0		0.0		72.0		0.0		0.0	
		3.1	64.0		0.0		0.0		52.0		0.0		0.0	
		4.1	72.0		0.0		0.0		84.0		0.0		0.0	
		1.2	16.0		0.0		0.0		56.0		0.0		0.0	
ELAV	UAS-grim;2.1+DJ1079	2.2	44.0		0.0		0.0		44.0		0.0		0.0	
		3.2	28.0		0.0		0.0		52.0		0.0		0.0	
		4.2	44.0		0.0		0.0		36.0		0.0		0.0	
			52.0	22.6	0.0	0.0	0.0	0.0	58.0	15.7	0.0	0.0	0.0	0.0
			**DJ646 + 0 GAL80**											
		1.1	88.0		18.2		25.0		76.0		57.9		0.0	
DJ646	UAS-grim	2.1	52.0		7.7		100.0		60.0		60.0		0.0	
		3.1	84.0		0.0		0.0		64.0		31.3		0.0	
		4.1	88.0		13.7		0.0		76.0		52.7		0.0	
		1.2	92.0		30.5		28.6		100.0		36.0		0.0	
		2.2	96.0		37.5		11.2		92.0		34.8		0.0	
		3.2	96.0		29.2		14.3		92.0		26.1		16.7	
		4.2	96.0		29.2		14.3		92.0		34.8		25.0	
			86.5	14.6	20.8	13.0	24.2	32.3	81.5	14.6	41.7	13.1	5.2	9.9
			**0 GAL80**											
		1.1	92.0		87.0		95.0		88.0		63.7		100.0	
DJ646	w	2.1	92.0		91.4		95.3		68.0		53.0		88.9	
		3.1	92.0		78.3		100.0		40.0		70.0		100.0	
		4.1	96.0		83.4		90.0		52.0		92.4		75.0	
		1.2	96.0		79.2		94.8		96.0		91.7		100.0	
		2.2	92.0		87.0		100.0		96.0		95.9		100.0	
		3.2	100.0		88.0		100.0		100.0		88.0		100.0	
		4.2	96.0		70.9		94.2		100.0		88.0		100.0	
			94.5	3.0	83.2	6.7	96.2	3.6	80.0	23.6	80.3	15.9	95.5	9.1

Parents		Plate #.	%L1	SD	%P	SD	%A	SD	%L1	SD	%P	SD	%A	SD
Male	Female	Exp #	AMP						AMP/TET					
DJ646 + DJ1077														
		1.1	56.0		85.8		100.0		92.0		87.0		80.0	
DJ646	UAS-grim;DJ1077	2.1	80.0		75.0		86.7		84.0		76.2		62.5	
		3.1	80.0		90.0		94.5		92.0		65.3		73.4	
		4.1	92.0		56.6		84.7		80.0		90.0		77.8	
			77.0	15.1	76.9	14.9	91.5	7.1	87.0	6.0	79.6	11.2	73.4	7.8
DJ646 + 3.3														
		1.1	92.0		52.2		66.7		80.0		75.0		13.4	
DJ646	UAS-grim;3.3	2.1	96.0		62.5		100.0		72.0		50.0		33.4	
		3.1	88.0		54.6		75.0		88.0		54.6		83.4	
		4.1	92.0		26.1		66.7		80.0		70.0		71.5	
			92.0	3.3	48.9	15.8	77.1	15.8	80.0	6.5	62.4	12.0	50.4	32.6
DJ646 + 1X146 + 1X147														
		1.1	84.0		47.7		90.0		92.0		69.6		93.8	
DJ646	UAS-grim;2.1+DJ1077	2.1	80.0		55.0		100.0		84.0		57.2		100.0	
		3.1	92.0		60.9		92.9		96.0		83.4		100.0	
		4.1	96.0		62.5		100.0		96.0		62.5		100.0	
		1.2	88.0		91.0		100.0		88.0		45.5		100.0	
DJ646	UAS-grim;2.1+DJ1079	2.2	76.0		79.0		93.4		80.0		85.0		100.0	
		3.2	72.0		72.3		100.0		76.0		63.2		100.0	
		4.2	68.0		64.8		100.0		72.0		55.6		100.0	
			82.0	9.8	66.7	13.8	97.0	4.2	85.5	9.1	65.3	13.6	99.2	2.2
YPI-GAL4 + 0 GAL80														
		1.1	96.0		58.4		100.0		100.0		80.0		100.0	
YPI-GAL4	UAS-grim	2.1	92.0		56.6		100.0		100.0		88.0		95.5	
		3.1	88.0		63.7		100.0		100.0		84.0		100.0	
		4.1	92.0		60.9		100.0		96.0		91.7		100.0	
		1.2	100.0		80.0		100.0		88.0		59.1		100.0	
		2.2	96.0		70.9		100.0		92.0		82.7		100.0	
		3.2	96.0		75.0		100.0		100.0		84.0		100.0	
		4.2	92.0		47.9		91.0		92.0		91.4		100.0	
			94.0	3.7	64.2	10.6	98.9	3.2	96.0	4.8	82.6	10.4	99.4	1.6
0 GAL80														
		1.1	64.0		56.3		77.8		32.0		62.5		100.0	
YPI-GAL4	w	2.1	40.0		60.0		83.4		44.0		63.7		85.8	
		3.1	44.0		81.9		100.0		44.0		72.8		87.5	
		4.1	28.0		85.8		100.0		36.0		66.7		100.0	
		1.2	100.0		76.0		100.0		96.0		87.5		100.0	
		2.2	92.0		78.3		100.0		92.0		87.0		100.0	
		3.2	96.0		79.2		100.0		92.0		87.0		100.0	
		4.2	100.0		76.0		100.0		100.0		96.0		100.0	
			70.5	30.1	74.2	10.4	95.2	9.1	67.0	30.3	77.9	13.0	96.7	6.2
YPI-GAL4 + DJ1077														
		1.1	72.0		66.7		100.0		80.0		85.0		100.0	
YPI-GAL4	UAS-grim;DJ1077	2.1	80.0		80.0		87.5		84.0		85.8		100.0	
		3.1	88.0		81.9		100.0		84.0		100.0		100.0	
		4.1	84.0		66.7		100.0		88.0		95.5		100.0	
			81.0	6.8	73.8	8.3	96.9	6.3	84.0	3.3	91.6	7.4	100.0	0.0
YPI-GAL4 + 3.3														
		1.1	96.0		83.4		95.0		96.0		66.7		100.0	
YPI-GAL4	UAS-grim;3.3	2.1	96.0		75.0		94.5		100.0		64.0		87.5	
		3.1	88.0		81.9		94.5		92.0		82.7		100.0	
		4.1	100.0		72.0		100.0		100.0		76.0		100.0	
			95.0	5.0	78.1	5.5	96.0	2.7	97.0	3.8	72.4	8.6	96.9	6.3
DJ628 + 0 GAL80														
		1.1	88.0		0.0		0.0		96.0		0.0		0.0	
DJ628	UAS-grim	2.1	100.0		0.0		0.0		96.0		0.0		0.0	
		3.1	100.0		0.0		0.0		100.0		0.0		0.0	
		4.1	96.0		0.0		0.0		96.0		0.0		0.0	
		1.2	88.0		0.0		0.0		76.0		0.0		0.0	
		2.2	64.0		0.0		0.0		76.0		0.0		0.0	
		3.2	68.0		0.0		0.0		84.0		0.0		0.0	
		4.2	68.0		0.0		0.0		80.0		0.0		0.0	
			84.0	15.1	0.0	0.0	0.0	0.0	88.0	10.0	0.0	0.0	0.0	0.0

Parents		Plate #.	%L1	SD	%P	SD	%A	SD	%L1	SD	%P	SD	%A	SD
Male	Female	Exp #	AMP						AMP/TET					
			**0 GAL80**											
		1.1	96.0		95.9		100.0		96.0		91.7		100.0	
DJ628	w	2.1	96.0		87.5		95.3		92.0		95.7		91.0	
		3.1	88.0		95.5		100.0		100.0		88.0		100.0	
		4.1	100.0		100.0		100.0		96.0		91.7		100.0	
		1.2	96.0		87.5		100.0		92.0		91.4		100.0	
		2.2	100.0		100.0		100.0		96.0		87.5		100.0	
		3.2	92.0		87.0		100.0		92.0		91.4		100.0	
		4.2	88.0		100.0		100.0		100.0		96.0		100.0	
			94.5	4.8	94.2	5.9	99.4	1.7	95.5	3.3	91.7	3.1	98.9	3.2
			**DJ628 + DJ1077**											
		1.1	84.0		33.4		0.0		100.0		0.0		0.0	
DJ628	UAS-grim;DJ1077	2.1	84.0		28.6		0.0		92.0		0.0		0.0	
		3.1	100.0		28.0		0.0		88.0		0.0		0.0	
		4.1	84.0		28.6		0.0		84.0		0.0		0.0	
		1.2	52.0		0.0		0.0		68.0		0.0		0.0	
		2.2	48.0		0.0		0.0		56.0		0.0		0.0	
		3.2	52.0		0.0		0.0		56.0		0.0		0.0	
		4.2	64.0		0.0		0.0		52.0		0.0		0.0	
			71.0	19.4	14.8	15.9	0.0	0.0	74.5	18.8	0.0	0.0	0.0	0.0
			**DJ628 + 3.3**											
		1.1	96.0		16.7		0.0		84.0		4.8		0.0	
DJ628	UAS-grim;3.3	2.1	92.0		0.0		0.0		76.0		0.0		0.0	
		3.1	84.0		9.6		0.0		80.0		0.0		0.0	
		4.1	84.0		19.1		0.0		76.0		0.0		0.0	
		1.2	56.0		35.8		0.0		40.0		30.0		0.0	
		2.2	56.0		0.0		0.0		36.0		11.2		0.0	
		3.2	64.0		6.3		0.0		56.0		7.2		0.0	
		4.2	68.0		0.0		0.0		28.0		28.6		0.0	
			75.0	16.0	10.9	12.5	0.0	0.0	59.5	22.4	10.2	12.4	0.0	0.0
			**DJ628 + 1X146 + 1X147**											
		1.1	76.0		42.2		0.0		84.0		14.3		0.0	
DJ628	UAS-grim;2.1+DJ1077	2.1	76.0		73.7		0.0		80.0		35.0		0.0	
		3.1	80.0		15.0		0.0		84.0		4.8		0.0	
		4.1	84.0		71.5		0.0		84.0		14.3		0.0	
		1.2	40.0		30.0		0.0		56.0		0.0		0.0	
DJ628	UAS-grim;2.1+DJ1079	2.2	32.0		25.0		0.0		48.0		0.0		0.0	
		3.2	36.0		0.0		0.0		76.0		0.0		0.0	
		4.2	48.0		16.7		0.0		52.0		0.0		0.0	
			59.0	22.0	34.3	26.6	0.0	0.0	70.5	15.7	8.6	12.4	0.0	0.0
			**DJ695 + 0 GAL80**											
		1.1	68.0		0.0		0.0		88.0		0.0		0.0	
DJ695	UAS-grim	2.1	84.0		0.0		0.0		84.0		0.0		0.0	
		3.1	76.0		0.0		0.0		88.0		0.0		0.0	
		4.1	84.0		0.0		0.0		96.0		0.0		0.0	
			78.0	7.7	0.0	0.0	0.0	0.0	89.0	5.0	0.0	0.0	0.0	0.0
			**0 GAL80**											
		1.1	96.0		91.7		100.0		88.0		95.5		100.0	
DJ695	w	2.1	92.0		78.3		100.0		80.0		95.0		100.0	
		3.1	92.0		91.4		100.0		96.0		95.9		95.7	
		4.1	92.0		91.4		95.3		88.0		86.4		100.0	
			93.0	2.0	88.2	6.6	98.8	2.4	88.0	6.5	93.2	4.5	98.9	2.2
			**DJ695 + DJ1077**											
		1.1	100.0		0.0		0.0		92.0		0.0		0.0	
DJ695	UAS-grim;DJ1077	2.1	92.0		0.0		0.0		84.0		0.0		0.0	
		3.1	96.0		0.0		0.0		84.0		0.0		0.0	
		4.1	96.0		0.0		0.0		96.0		0.0		0.0	
			96.0	3.3	0.0	0.0	0.0	0.0	89.0	6.0	0.0	0.0	0.0	0.0
			**DJ695 + 3.3**											
		1.1	80.0		0.0		0.0		72.0		0.0		0.0	
DJ695	UAS-grim;3.3	2.1	80.0		0.0		0.0		80.0		0.0		0.0	
		3.1	80.0		0.0		0.0		60.0		0.0		0.0	
		4.1	84.0		0.0		0.0		72.0		0.0		0.0	
			81.0	2.0	0.0	0.0	0.0	0.0	71.0	8.2	0.0	0.0	0.0	0.0

Parents		Plate #.	%L1	SD	%P	SD	%A	SD	%L1	SD	%P	SD	%A	SD
Male	Female	Exp #	AMP						AMP/TET					
			**DJ695 + 1X146 + 1X147**											
DJ695	UAS-grim;2.1+DJ1077	1.1	76.0		0.0		0.0		80.0		0.0		0.0	
		2.1	88.0		0.0		0.0		76.0		0.0		0.0	
		3.1	96.0		0.0		0.0		80.0		0.0		0.0	
		4.1	92.0		0.0		0.0		80.0		0.0		0.0	
DJ695	UAS-grim;2.1+DJ1079	1.2	40.0		0.0		0.0		68.0		0.0		0.0	
		2.2	52.0		0.0		0.0		56.0		0.0		0.0	
		3.2	48.0		0.0		0.0		76.0		0.0		0.0	
		4.2	28.0		0.0		0.0		72.0		0.0		0.0	
			65.0	26.2	0.0	0.0	0.0	0.0	73.5	8.3	0.0	0.0	0.0	0.0
			**DJ715 + 0 GAL80**											
DJ715	UAS-grim;2.1+DJ1079	1.1	80.0		0.0		0.0		68.0		0.0		0.0	
		2.1	84.0		0.0		0.0		92.0		0.0		0.0	
		3.1	80.0		0.0		0.0		76.0		0.0		0.0	
		4.1	68.0		0.0		0.0		72.0		0.0		0.0	
		1.2	60.0		0.0		0.0		52.0		0.0		0.0	
		2.2	56.0		0.0		0.0		56.0		0.0		0.0	
		3.2	72.0		0.0		0.0		52.0		0.0		0.0	
		4.2	48.0		0.0		0.0		60.0		0.0		0.0	
			68.5	12.9	0.0	0.0	0.0	0.0	66.0	13.9	0.0	0.0	0.0	0.0
			**0 GAL80**											
DJ715	w	1.1	96.0		62.5		93.4		84.0		81.0		100.0	
		2.1	96.0		100.0		95.9		80.0		80.0		100.0	
		3.1	96.0		83.4		100.0		84.0		81.0		100.0	
		4.1	100.0		84.0		100.0		68.0		76.5		92.4	
		1.2	80.0		100.0		100.0		92.0		91.4		100.0	
		2.2	84.0		76.2		100.0		92.0		87.0		100.0	
		3.2	96.0		87.5		100.0		96.0		83.4		100.0	
		4.2	92.0		87.0		100.0		96.0		100.0		100.0	
			92.5	6.9	85.1	12.2	98.7	2.6	86.5	9.5	85.0	7.6	99.1	2.7
			**DJ715 + DJ1077**											
DJ715	UAS-grim;DJ1077	1.1	52.0		0.0		0.0		52.0		0.0		0.0	
		2.1	40.0		0.0		0.0		36.0		0.0		0.0	
		3.1	52.0		0.0		0.0		48.0		0.0		0.0	
		4.1	60.0		0.0		0.0		52.0		0.0		0.0	
			51.0	8.2	0.0	0.0	0.0	0.0	47.0	7.6	0.0	0.0	0.0	0.0
			**DJ715 + 3.3**											
DJ715	UAS-grim;3.3	1.1	32.0		0.0		0.0		56.0		0.0		0.0	
		2.1	40.0		0.0		0.0		56.0		0.0		0.0	
		3.1	44.0		0.0		0.0		56.0		0.0		0.0	
		4.1	68.0		0.0		0.0		48.0		0.0		0.0	
		1.2	44.0		0.0		0.0		48.0		0.0		0.0	
		2.2	32.0		0.0		0.0		36.0		0.0		0.0	
		3.2	44.0		0.0		0.0		48.0		0.0		0.0	
		4.2	32.0		0.0		0.0		24.0		0.0		0.0	
			42.0	11.9	0.0	0.0	0.0	0.0	46.5	11.3	0.0	0.0	0.0	0.0
			**DJ715 + 1X146 + 1X147**											
DJ715	UAS-grim;2.1+DJ1077	1.1	32.0		0.0		0.0		20.0		0.0		0.0	
		2.1	32.0		0.0		0.0		32.0		0.0		0.0	
		3.1	40.0		0.0		0.0		24.0		0.0		0.0	
		4.1	28.0		0.0		0.0		52.0		0.0		0.0	
DJ715	UAS-grim;2.1+DJ1079	1.2	16.0		0.0		0.0		24.0		0.0		0.0	
		2.2	36.0		0.0		0.0		16.0		0.0		0.0	
		3.2	32.0		0.0		0.0		32.0		0.0		0.0	
		4.2	32.0		0.0		0.0		28.0		0.0		0.0	
			31.0	7.0	0.0	0.0	0.0	0.0	28.5	11.0	0.0	0.0	0.0	0.0
			**DJ752 + 0 GAL80**											
DJ752	UAS-grim	1.1	0.0		0.0		0.0		0.0		0.0		0.0	
		2.1	0.0		0.0		0.0		0.0		0.0		0.0	
		3.1	0.0		0.0		0.0		0.0		0.0		0.0	
		4.1	0.0		0.0		0.0		0.0		0.0		0.0	
			0.0	0.0	0.0	0.0	0.0	0.0	0.0	0.0	0.0	0.0	0.0	0.0
			**0 GAL80**											
DJ752	w	1.1	100.0		80.0		95.0		80.0		95.0		94.8	
		2.1	96.0		95.9		100.0		100.0		100.0		100.0	
		3.1	92.0		100.0		100.0		100.0		96.0		100.0	
		4.1	96.0		91.7		100.0		96.0		100.0		100.0	
			96.0	3.3	91.9	8.6	98.8	2.5	94.0	9.5	97.8	2.6	98.7	2.6

Parents		Plate #.	%L1	SD	%P	SD	%A	SD	%L1	SD	%P	SD	%A	SD
Male	Female	Exp #	AMP						AMP/TET					
			**DJ752 + DJ1077**											
DJ752	UAS-grim;DJ1077	1.1	36.0		0.0		0.0		20.0		0.0		0.0	
		2.1	20.0		0.0		0.0		20.0		0.0		0.0	
		3.1	16.0		0.0		0.0		0.0		0.0		0.0	
		4.1	20.0		0.0		0.0		20.0		0.0		0.0	
			23.0	8.9	0.0	0.0	0.0	0.0	15.0	10.0	0.0	0.0	0.0	0.0
			**DJ752 + 3.3**											
DJ752	UAS-grim;3.3	1.1	0.0		0.0		0.0		0.0		0.0		0.0	
		2.1	0.0		0.0		0.0		0.0		0.0		0.0	
		3.1	0.0		0.0		0.0		0.0		0.0		0.0	
		4.1	0.0		0.0		0.0		0.0		0.0		0.0	
			0.0	0.0	0.0	0.0	0.0	0.0	0.0	0.0	0.0	0.0	0.0	0.0
			**DJ752 + 1X146 + 1X147**											
DJ752	UAS-grim;2.1+DJ1077	1.1	84.0		38.1		0.0		88.0		9.1		0.0	
		2.1	84.0		66.7		0.0		80.0		10.0		0.0	
		3.1	68.0		64.8		0.0		92.0		13.1		0.0	
		4.1	88.0		45.5		0.0		76.0		15.8		0.0	
DJ752	UAS-grim;2.1+DJ1079	1.2	36.0		11.2		0.0		56.0		14.3		0.0	
		2.2	48.0		33.4		0.0		72.0		5.6		0.0	
		3.2	52.0		30.8		0.0		48.0		0.0		0.0	
		4.2	40.0		30.0		0.0		44.0		9.1		0.0	
			62.5	21.2	40.1	18.6	0.0	0.0	69.5	18.1	9.6	5.1	0.0	0.0
			**DJ761 + 0 GAL80**											
DJ761	UAS-grim	1.1	0.0		0.0		0.0		0.0		0.0		0.0	
		2.1	0.0		0.0		0.0		0.0		0.0		0.0	
		3.1	0.0		0.0		0.0		0.0		0.0		0.0	
		4.1	0.0		0.0		0.0		0.0		0.0		0.0	
			0.0	0.0	0.0	0.0	0.0	0.0	0.0	0.0	0.0	0.0	0.0	0.0
			**0 GAL80**											
DJ761	w	1.1	96.0		87.5		95.3		96.0		83.4		95.0	
		2.1	100.0		92.0		100.0		92.0		78.3		100.0	
		3.1	100.0		84.0		100.0		96.0		75.0		94.5	
		4.1	96.0		83.4		100.0		92.0		82.7		100.0	
			98.0	2.3	86.7	4.0	98.8	2.4	94.0	2.3	79.9	3.9	97.4	3.0
			**DJ761 + DJ1077**											
DJ761	UAS-grim;DJ1077	1.1	48.0		0.0		0.0		72.0		0.0		0.0	
		2.1	32.0		0.0		0.0		56.0		0.0		0.0	
		3.1	36.0		0.0		0.0		56.0		0.0		0.0	
		4.1	56.0		0.0		0.0		56.0		0.0		0.0	
			43.0	11.0	0.0	0.0	0.0	0.0	60.0	8.0	0.0	0.0	0.0	0.0
			**DJ761 + 3.3**											
DJ761	UAS-grim;3.3	1.1	0.0		0.0		0.0		0.0		0.0		0.0	
		2.1	0.0		0.0		0.0		0.0		0.0		0.0	
		3.1	0.0		0.0		0.0		0.0		0.0		0.0	
		4.1	0.0		0.0		0.0		0.0		0.0		0.0	
			0.0	0.0	0.0	0.0	0.0	0.0	0.0	0.0	0.0	0.0	0.0	0.0
			**DJ761 + 1X146 + 1X147**											
DJ761	UAS-grim;2.1+DJ1077	1.1	76.0		0.0		0.0		56.0		0.0		0.0	
		2.1	72.0		0.0		0.0		80.0		0.0		0.0	
		3.1	80.0		0.0		0.0		76.0		0.0		0.0	
		4.1	80.0		0.0		0.0		56.0		0.0		0.0	
DJ761	UAS-grim;2.1+DJ1079	1.2	52.0		0.0		0.0		52.0		0.0		0.0	
		2.2	60.0		0.0		0.0		44.0		0.0		0.0	
		3.2	60.0		0.0		0.0		48.0		0.0		0.0	
		4.2	64.0		0.0		0.0		52.0		0.0		0.0	
			68.0	10.5	0.0	0.0	0.0	0.0	58.0	13.0	0.0	0.0	0.0	0.0
			**DJ710 + 0 GAL80**											
DJ710	UAS-grim	1.1	100.0		0.0		0.0		100.0		0.0		0.0	
		2.1	92.0		0.0		0.0		100.0		0.0		0.0	
		3.1	96.0		0.0		0.0		100.0		0.0		0.0	
		4.1	100.0		0.0		0.0		96.0		0.0		0.0	
			97.0	3.8	0.0	0.0	0.0	0.0	99.0	2.0	0.0	0.0	0.0	0.0

Parents		Plate #.	%L1	SD	%P	SD	%A	SD	%L1	SD	%P	SD	%A	SD
Male	Female	Exp #	AMP						AMP/TET					
**0 GAL80**														
DJ710	w	1.1	100.0		92.0		100.0		104.0		80.8		100.0	
		2.1	96.0		79.2		100.0		100.0		100.0		88.0	
		3.1	100.0		84.0		100.0		88.0		86.4		100.0	
		4.1	96.0		91.7		100.0		96.0		91.7		100.0	
			98.0	2.3	86.7	6.2	100.0	0.0	97.0	6.8	89.7	8.2	97.0	6.0
**DJ710 + DJ1077**														
DJ710	UAS-grim;DJ1077	1.1	92.0		74.0		64.8		80.0		0.0		0.0	
		2.1	92.0		60.9		35.8		84.0		0.0		0.0	
		3.1	80.0		55.0		54.6		100.0		0.0		0.0	
		4.1	84.0		66.7		42.9		96.0		0.0		0.0	
			87.0	6.0	64.2	8.1	49.5	12.8	90.0	9.5	0.0	0.0	0.0	0.0
**DJ710 + 3.3**														
DJ710	UAS-grim;3.3	1.1	100.0		72.0		0.0		100.0		0.0		0.0	
		2.1	96.0		50.0		0.0		80.0		0.0		0.0	
		3.1	96.0		79.2		0.0		88.0		0.0		0.0	
		4.1	100.0		72.0		0.0		84.0		0.0		0.0	
			98.0	2.3	68.3	12.7	0.0	0.0	88.0	8.6	0.0	0.0	0.0	0.0
**DJ710 + 1X146 + 1X147**														
DJ710	UAS-grim;2.1+DJ1077	1.1	80.0		90.0		38.9		100.0		0.0		0.0	
		2.1	96.0		79.2		36.9		100.0		16.0		0.0	
		3.1	92.0		78.3		33.4		96.0		12.5		0.0	
		4.1	92.0		87.0		35.0		100.0		4.0		0.0	
DJ710	UAS-grim;2.1+DJ1079	1.2	52.0		38.5		40.0		76.0		10.6		0.0	
		2.2	56.0		57.2		12.5		80.0		35.0		1.0	
		3.2	48.0		25.0		0.0		68.0		41.2		2.0	
		4.2	48.0		33.4		25.0		72.0		16.7		3.0	
			70.5	21.5	61.1	26.0	27.7	14.4	86.5	13.8	17.0	14.3	0.8	1.2
**DJ849 + 0 GAL80**														
DJ849	UAS-grim	1.1	92.0		52.2		91.7		100.0		64.0		87.5	
		2.1	92.0		56.6		92.4		92.0		56.6		77.0	
		3.1	88.0		50.0		100.0		92.0		60.9		78.6	
		4.1	92.0		52.2		91.7		92.0		65.3		93.4	
		1.2	76.0		68.5		100.0		84.0		100.0		71.5	
		2.2	68.0		53.0		55.6		76.0		100.0		36.9	
		3.2	60.0		53.4		87.5		76.0		84.3		56.3	
		4.2	68.0		64.8		72.8		80.0		85.0		82.4	
			79.5	13.1	56.3	6.7	86.5	15.1	86.5	8.8	77.0	17.6	73.0	18.3
**0 GAL80**														
DJ849	w	1.1	100.0		100.0		100.0		96.0		95.9		100.0	
		2.1	100.0		100.0		96.0		96.0		95.9		95.7	
		3.1	100.0		96.0		100.0		100.0		92.0		100.0	
		4.1	92.0		87.0		95.0		96.0		95.9		100.0	
		1.2	96.0		91.7		95.5		100.0		88.0		100.0	
		2.2	100.0		96.0		95.9		96.0		87.5		95.3	
		3.2	92.0		100.0		100.0		100.0		96.0		100.0	
		4.2	100.0		100.0		100.0		100.0		96.0		100.0	
			97.5	3.7	96.3	4.8	97.8	2.4	98.0	2.1	93.4	3.7	98.9	2.1
**DJ849 + DJ1077**														
DJ849	UAS-grim;DJ1077	1.1	84.0		62.0		92.4		92.0		69.6		93.8	
		2.1	92.0		56.6		92.4		96.0		66.7		93.8	
		3.1	80.0		55.0		100.0		96.0		83.4		90.0	
		4.1	92.0		69.6		87.5		92.0		65.3		86.7	
			87.0	6.0	60.8	6.6	93.1	5.2	94.0	2.3	71.3	8.3	91.1	3.4
**DJ849 + 3.3**														
DJ849	UAS-grim;3.3	1.1	88.0		59.1		84.7		96.0		79.2		89.5	
		2.1	92.0		69.6		81.3		96.0		54.2		61.6	
		3.1	92.0		74.0		70.6		80.0		60.0		83.4	
		4.1	92.0		69.6		93.8		84.0		81.0		70.6	
		1.2	92.0		65.3		93.4		80.0		55.0		100.0	
		2.2	96.0		70.9		88.3		88.0		86.4		89.5	
		3.2	88.0		68.2		100.0		88.0		86.4		100.0	
		4.2	80.0		65.0		77.0		88.0		50.0		100.0	
			90.0	4.8	67.7	4.5	86.1	9.7	87.5	6.2	69.0	15.6	86.8	14.4

Parents: Male	Female	Plate #. Exp #	AMP %L1	SD	%P	SD	%A	SD	AMP/TET %L1	SD	%P	SD	%A	SD
			**DJ849 + 1X146 + 1X147**											
DJ849	UAS-grim;2.1+DJ1077	1.1	60.0		100.0		100.0		72.0		83.4		100.0	
		2.1	72.0		66.7		100.0		84.0		52.4		100.0	
		3.1	76.0		68.5		100.0		88.0		86.4		100.0	
		4.1	64.0		81.3		100.0		72.0		83.4		100.0	
DJ849	UAS-grim;2.1+DJ1079	1.2	64.0		37.5		100.0		96.0		33.4		100.0	
		2.2	52.0		38.5		100.0		76.0		52.7		100.0	
		3.2	68.0		58.9		100.0		60.0		40.0		83.4	
		4.2	64.0		56.3		88.9		48.0		41.7		100.0	
			65.0	7.3	63.5	20.9	98.6	3.9	74.5	15.4	59.2	21.8	97.9	5.9
			**cha-GAL4 + 0 GAL80**											
cha-GAL4	UAS-grim	1.1	96.0		0.0		0.0		100.0		0.0		0.0	
		2.1	96.0		0.0		0.0		100.0		0.0		0.0	
		3.1	100.0		0.0		0.0		92.0		0.0		0.0	
		4.1	100.0		0.0		0.0		92.0		0.0		0.0	
		1.2	96.0		0.0		0.0		100.0		0.0		0.0	
		2.2	100.0		0.0		0.0		100.0		0.0		0.0	
		3.2	96.0		0.0		0.0		100.0		0.0		0.0	
		4.2	100.0		0.0		0.0		100.0		0.0		0.0	
			98.0	2.1	0.0	0.0	0.0	0.0	98.0	3.7	0.0	0.0	0.0	0.0
			**0 GAL80**											
cha-GAL4	w	1.1	100.0		92.0		100.0		100.0		88.0		86.4	
		2.1	100.0		88.0		100.0		100.0		92.0		87.0	
		3.1	100.0		92.0		91.4		96.0		95.9		47.9	
		4.1	100.0		92.0		100.0		92.0		87.0		75.0	
		1.2	96.0		95.9		100.0		100.0		84.0		100.0	
		2.2	96.0		87.5		100.0		100.0		92.0		100.0	
		3.2	100.0		80.0		100.0		100.0		84.0		100.0	
		4.2	100.0		88.0		100.0		96.0		100.0		100.0	
			99.0	1.9	89.4	4.8	98.9	3.0	98.0	3.0	90.4	5.7	87.0	18.3
			**cha-GAL4 + DJ1077**											
cha-GAL4	UAS-grim;DJ1077	1.1	96.0		0.0		0.0		88.0		0.0		0.0	
		2.1	100.0		0.0		0.0		92.0		0.0		0.0	
		3.1	96.0		0.0		0.0		92.0		0.0		0.0	
		4.1	88.0		0.0		0.0		96.0		0.0		0.0	
			95.0	5.0	0.0	0.0	0.0	0.0	92.0	3.3	0.0	0.0	0.0	0.0
			**cha-GAL4 + 3.3**											
cha-GAL4	UAS-grim;3.3	1.1	88.0		0.0		0.0		92.0		0.0		0.0	
		2.1	88.0		0.0		1.0		100.0		0.0		0.0	
		3.1	100.0		0.0		2.0		100.0		0.0		0.0	
		4.1	92.0		0.0		3.0		100.0		0.0		0.0	
			92.0	5.7	0.0	0.0	1.5	1.3	98.0	4.0	0.0	0.0	0.0	0.0
			**cha-GAL4 + 1X146 + 1X147**											
cha-GAL4	UAS-grim;2.1+DJ1077	1.1	96.0		0.0		0.0		92.0		0.0		0.0	
		2.1	92.0		0.0		0.0		84.0		0.0		0.0	
		3.1	88.0		0.0		0.0		72.0		0.0		0.0	
		4.1	80.0		0.0		0.0		84.0		0.0		0.0	
cha-GAL4	UAS-grim;2.1+DJ1079	1.2	96.0		4.2		0.0		76.0		0.0		0.0	
		2.2	84.0		9.6		0.0		100.0		0.0		0.0	
		3.2	72.0		0.0		0.0		96.0		0.0		0.0	
		4.2	80.0		0.0		0.0		84.0		0.0		0.0	
			86.0	8.6	1.7	3.5	0.0	0.0	86.0	9.6	0.0	0.0	0.0	0.0
			**ddc-GAL4 + 0 GAL80**											
ddc-GAL4	UAS-grim	1.1	76.0		0.0		0.0		76.0		0.0		0.0	
		2.1	80.0		0.0		0.0		72.0		0.0		0.0	
		3.1	76.0		0.0		0.0		76.0		0.0		0.0	
		4.1	64.0		0.0		0.0		72.0		0.0		0.0	
		1.2	84.0		0.0		0.0		96.0		0.0		0.0	
		2.2	92.0		0.0		0.0		84.0		0.0		0.0	
		3.2	92.0		0.0		0.0		84.0		0.0		0.0	
		4.2	92.0		0.0		0.0		88.0		0.0		0.0	
			82.0	10.0	0.0	0.0	0.0	0.0	81.0	8.5	0.0	0.0	0.0	0.0

Parents		Plate #.	%L1	SD	%P	SD	%A	SD	%L1	SD	%P	SD	%A	SD
Male	Female	Exp #	AMP						AMP/TET					
			**0 GAL80**											
ddc-GAL4	w	1.1	92.0		91.4		95.3		96.0		83.4		90.0	
		2.1	96.0		95.9		95.7		100.0		100.0		92.0	
		3.1	96.0		95.9		95.7		96.0		100.0		95.9	
		4.1	100.0		100.0		96.0		100.0		88.0		95.5	
		1.2	100.0		84.0		95.3		88.0		86.4		100.0	
		2.2	100.0		96.0		100.0		88.0		81.9		100.0	
		3.2	100.0		72.0		100.0		96.0		83.4		90.0	
		4.2	100.0		76.0		100.0		96.0		83.4		100.0	
			98.0	3.0	88.9	10.4	97.3	2.3	95.0	4.7	88.3	7.5	95.4	4.4
			**ddc-GAL4 + DJ1077**											
ddc-GAL4	UAS-grim;DJ1077	1.1	92.0		0.0		0.0		92.0		0.0		0.0	
		2.1	88.0		0.0		0.0		100.0		0.0		0.0	
		3.1	88.0		0.0		0.0		92.0		0.0		0.0	
		4.1	80.0		0.0		0.0		80.0		0.0		0.0	
			87.0	5.0	0.0	0.0	0.0	0.0	91.0	8.2	0.0	0.0	0.0	0.0
			**ddc-GAL4 + 3.3**											
ddc-GAL4	UAS-grim;3.3	1.1	92.0		0.0		0.0		100.0		0.0		0.0	
		2.1	92.0		0.0		0.0		88.0		0.0		0.0	
		3.1	96.0		0.0		0.0		100.0		0.0		0.0	
		4.1	84.0		0.0		0.0		100.0		0.0		0.0	
			91.0	5.0	0.0	0.0	0.0	0.0	97.0	6.0	0.0	0.0	0.0	0.0
			**ddc-GAL4 + 1X146 + 1X147**											
ddc-GAL4	UAS-grim;2.1+DJ1077	1.1	88.0		0.0		0.0		88.0		0.0		0.0	
		2.1	88.0		0.0		0.0		84.0		0.0		0.0	
		3.1	88.0		0.0		0.0		88.0		0.0		0.0	
		4.1	88.0		0.0		0.0		88.0		0.0		0.0	
ddc-GAL4	UAS-grim;2.1+DJ1079	1.2	76.0		0.0		0.0		92.0		0.0		0.0	
		2.2	64.0		0.0		0.0		84.0		0.0		0.0	
		3.2	76.0		0.0		0.0		88.0		0.0		0.0	
		4.2	68.0		0.0		0.0		72.0		0.0		0.0	
			79.5	9.9	0.0	0.0	0.0	0.0	85.5	6.0	0.0	0.0	0.0	0.0
			**Nrv2-GAL4 + 0 GAL80**											
Nrv2-GAL4	UAS-grim	1.1	96.0		0.0		0.0		84.0		0.0		0.0	
		2.1	80.0		0.0		0.0		92.0		0.0		0.0	
		3.1	96.0		0.0		0.0		92.0		0.0		0.0	
		4.1	84.0		0.0		0.0		76.0		0.0		0.0	
		1.2	92.0		0.0		0.0		96.0		0.0		0.0	
		2.2	96.0		0.0		0.0		100.0		0.0		0.0	
		3.2	100.0		0.0		0.0		96.0		0.0		0.0	
		4.2	96.0		0.0		0.0		100.0		0.0		0.0	
			92.5	6.9	0.0	0.0	0.0	0.0	92.0	8.3	0.0	0.0	0.0	0.0
			**0 GAL80**											
Nrv2-GAL4	w	1.1	88.0		86.4		89.5		84.0		90.5		100.0	
		2.1	96.0		75.0		100.0		96.0		95.9		95.7	
		3.1	100.0		84.0		100.0		84.0		81.0		82.4	
		4.1	96.0		87.5		95.3		96.0		66.7		100.0	
		1.2	100.0		92.0		100.0		100.0		88.0		100.0	
		2.2	100.0		92.0		100.0		92.0		100.0		100.0	
		3.2	100.0		92.0		95.7		100.0		100.0		100.0	
		4.2	100.0		80.0		100.0		96.0		87.5		95.3	
			97.5	4.2	86.1	6.2	97.6	3.8	93.5	6.4	88.7	11.1	96.7	6.1
			**Nrv2-GAL4 + DJ1077**											
Nrv2-GAL4	UAS-grim;DJ1077	1.1	92.0		0.0		0.0		96.0		0.0		0.0	
		2.1	80.0		0.0		0.0		96.0		0.0		0.0	
		3.1	80.0		0.0		0.0		100.0		0.0		0.0	
		4.1	88.0		0.0		0.0		96.0		0.0		0.0	
			85.0	6.0	0.0	0.0	0.0	0.0	97.0	2.0	0.0	0.0	0.0	0.0
			**Nrv2-GAL4 + 3.3**											
Nrv2-GAL4	UAS-grim;3.3	1.1	76.0		0.0		0.0		100.0		0.0		0.0	
		2.1	72.0		0.0		0.0		96.0		0.0		0.0	
		3.1	68.0		0.0		0.0		96.0		0.0		0.0	
		4.1	76.0		0.0		0.0		92.0		0.0		0.0	
			73.0	3.8	0.0	0.0	0.0	0.0	96.0	3.3	0.0	0.0	0.0	0.0

Parents		Plate #.	%L1	SD	%P	SD	%A	SD	%L1	SD	%P	SD	%A	SD
Male	Female	Exp #	AMP						AMP/TET					
			**Nrv2-GAL4 + 1X146 + 1X147**											
Nrv2-GAL4	UAS-grim;2.1+DJ1077	1.1	80.0		25.0		40.0		72.0		16.7		0.0	
		2.1	84.0		42.9		55.6		72.0		33.4		0.0	
		3.1	68.0		47.1		62.5		76.0		21.1		0.0	
		4.1	84.0		42.9		77.8		88.0		27.3		0.0	
Nrv2-GAL4	UAS-grim;2.1+DJ1079	1.2	88.0		63.7		64.3		68.0		23.6		0.0	
		2.2	68.0		64.8		54.6		80.0		40.0		0.0	
		3.2	84.0		62.0		84.7		80.0		35.0		0.0	
		4.2	80.0		45.0		66.7		76.0		21.1		0.0	
			79.5	7.5	49.2	13.7	63.3	13.9	76.5	6.2	27.3	8.1	0.0	0.0
			**DJ755 + 0 GAL80**											
DJ755	UAS-grim	1.1	0.0		0.0		0.0		0.0		0.0		0.0	
		2.1	0.0		0.0		0.0		0.0		0.0		0.0	
		3.1	0.0		0.0		0.0		0.0		0.0		0.0	
		4.1	0.0		0.0		0.0		0.0		0.0		0.0	
			0.0	0.0	0.0	0.0	0.0	0.0	0.0	0.0	0.0	0.0	0.0	0.0
			**0 GAL80**											
DJ755	w	1.1	96.0		91.7		100.0		96.0		95.9		100.0	
		2.1	100.0		100.0		100.0		96.0		100.0		100.0	
		3.1	92.0		100.0		95.7		96.0		100.0		91.7	
		4.1	100.0		100.0		100.0		84.0		90.5		100.0	
			97.0	3.8	97.9	4.2	98.9	2.2	93.0	6.0	96.6	4.5	97.9	4.2
			**DJ755 + DJ1077**											
DJ755	UAS-grim;DJ1077	1.1	64.0		0.0		0.0		68.0		0.0		0.0	
		2.1	56.0		0.0		0.0		80.0		0.0		0.0	
		3.1	52.0		0.0		0.0		72.0		0.0		0.0	
		4.1	68.0		0.0		0.0		72.0		0.0		0.0	
			60.0	7.3	0.0	0.0	0.0	0.0	73.0	5.0	0.0	0.0	0.0	0.0
			**DJ755 + 3.3**											
DJ755	UAS-grim;3.3	1.1	0.0		0.0		0.0		0.0		0.0		0.0	
		2.1	0.0		0.0		0.0		0.0		0.0		0.0	
		3.1	0.0		0.0		0.0		0.0		0.0		0.0	
		4.1	0.0		0.0		0.0		0.0		0.0		0.0	
			0.0	0.0	0.0	0.0	0.0	0.0	0.0	0.0	0.0	0.0	0.0	0.0
			**DJ755 + 1X146 + 1X147**											
DJ755	UAS-grim;2.1+DJ1077	1.1	84.0		57.2		0.0		24.0		0.0		0.0	
		2.1	80.0		55.0		0.0		84.0		0.0		0.0	
		3.1	80.0		55.0		0.0		76.0		0.0		0.0	
		4.1	76.0		42.2		0.0		76.0		0.0		0.0	
DJ755	UAS-grim;2.1+DJ1079	1.2	40.0		50.0		0.0		48.0		25.0		0.0	
		2.2	80.0		50.0		0.0		32.0		25.0		0.0	
		3.2	76.0		47.4		0.0		40.0		10.0		0.0	
		4.2	48.0		33.4		0.0		68.0		17.7		0.0	
			70.5	16.7	48.8	7.9	0.0	0.0	56.0	22.8	9.7	11.4	0.0	0.0
			**DJ756 + 0 GAL80**											
DJ756	UAS-grim	1.1	0.0		0.0		0.0		0.0		0.0		0.0	
		2.1	0.0		0.0		0.0		0.0		0.0		0.0	
		3.1	0.0		0.0		0.0		0.0		0.0		0.0	
		4.1	0.0		0.0		0.0		0.0		0.0		0.0	
			0.0	0.0	0.0	0.0	0.0	0.0	0.0	0.0	0.0	0.0	0.0	0.0
			**0 GAL80**											
DJ756	w	1.1	92.0		100.0		100.0		96.0		91.7		100.0	
		2.1	92.0		91.4		100.0		84.0		81.0		100.0	
		3.1	96.0		87.5		100.0		92.0		87.0		100.0	
		4.1	96.0		91.7		100.0		100.0		92.0		100.0	
			94.0	2.3	92.7	5.3	100.0	0.0	93.0	6.8	87.9	5.2	100.0	0.0
			**DJ756 + DJ1077**											
DJ756	UAS-grim;DJ1077	1.1	60.0		0.0		0.0		56.0		0.0		0.0	
		2.1	48.0		0.0		1.0		56.0		0.0		1.0	
		3.1	52.0		0.0		2.0		72.0		0.0		2.0	
		4.1	52.0		0.0		3.0		56.0		0.0		3.0	
			53.0	5.0	0.0	0.0	1.5	1.3	60.0	8.0	0.0	0.0	1.5	1.3

Parents		Plate #.	%L1	SD	%P	SD	%A	SD	%L1	SD	%P	SD	%A	SD
Male	Female	Exp #	AMP						AMP/TET					
			**DJ756 + 3.3**											
DJ756	UAS-grim;3.3	1.1	0.0		0.0		0.0		0.0		0.0		0.0	
		2.1	0.0		0.0		0.0		0.0		0.0		0.0	
		3.1	0.0		0.0		0.0		0.0		0.0		0.0	
		4.1	0.0		0.0		0.0		0.0		0.0		0.0	
			0.0	0.0	0.0	0.0	0.0	0.0	0.0	0.0	0.0	0.0	0.0	0.0
			**DJ756 + 1X146 + 1X147**											
DJ756	UAS-grim;2.1+DJ1077	1.1	64.0		43.8		0.0		60.0		6.7		0.0	
		2.1	48.0		50.0		0.0		24.0		0.0		0.0	
		3.1	64.0		31.3		0.0		64.0		6.3		0.0	
		4.1	32.0		37.5		0.0		44.0		9.1		0.0	
DJ756	UAS-grim;2.1+DJ1079	1.2	76.0		52.7		0.0		60.0		6.7		0.0	
		2.2	68.0		47.1		0.0		68.0		0.0		0.0	
		3.2	76.0		57.9		0.0		76.0		10.6		0.0	
		4.2	60.0		53.4		0.0		80.0		20.0		0.0	
			61.0	14.8	46.7	8.8	0.0	0.0	59.5	18.1	7.4	6.4	0.0	0.0
			**DJ785 + 0 GAL80**											
DJ785	UAS-grim	1.1	80.0		0.0		0.0		96.0		0.0		0.0	
		2.1	84.0		0.0		0.0		84.0		0.0		0.0	
		3.1	72.0		0.0		0.0		92.0		0.0		0.0	
		4.1	80.0		0.0		0.0		92.0		0.0		0.0	
			79.0	5.0	0.0	0.0	0.0	0.0	91.0	5.0	0.0	0.0	0.0	0.0
			**0 GAL80**											
DJ785	w	1.1	96.0		91.7		100.0		96.0		91.7		100.0	
		2.1	96.0		100.0		95.9		100.0		96.0		100.0	
		3.1	96.0		95.9		100.0		100.0		96.0		91.7	
		4.1	100.0		92.0		100.0		100.0		92.0		95.7	
			97.0	2.0	94.9	3.9	99.0	2.1	99.0	2.0	93.9	2.4	96.9	4.0
			**DJ785 + DJ1077**											
DJ785	UAS-grim;DJ1077	1.1	84.0		0.0		0.0		80.0		0.0		0.0	
		2.1	64.0		0.0		0.0		88.0		0.0		0.0	
		3.1	84.0		0.0		0.0		88.0		0.0		0.0	
		4.1	100.0		0.0		0.0		80.0		0.0		0.0	
			83.0	14.7	0.0	0.0	0.0	0.0	84.0	4.6	0.0	0.0	0.0	0.0
			**DJ785 + 3.3**											
DJ785	UAS-grim;3.3	1.1	80.0		0.0		0.0		80.0		0.0		0.0	
		2.1	72.0		0.0		0.0		60.0		0.0		0.0	
		3.1	68.0		0.0		0.0		84.0		0.0		0.0	
		4.1	64.0		0.0		0.0		64.0		0.0		0.0	
			71.0	6.8	0.0	0.0	0.0	0.0	72.0	11.8	0.0	0.0	0.0	0.0
			**DJ785 + 1X146 + 1X147**											
DJ785	UAS-grim;2.1+DJ1077	1.1	64.0		56.3		100.0		72.0		5.6		0.0	
		2.1	72.0		50.0		88.9		80.0		15.0		66.7	
		3.1	68.0		41.2		100.0		64.0		0.0		0.0	
		4.1	68.0		41.2		100.0		64.0		18.8		100.0	
DJ785	UAS-grim;2.1+DJ1079	1.2	84.0		66.7		92.9		88.0		18.2		50.0	
		2.2	92.0		78.3		94.5		88.0		22.8		40.0	
		3.2	92.0		56.6		92.4		92.0		17.4		100.0	
		4.2	96.0		54.2		100.0		96.0		16.7		50.0	
			79.5	12.9	55.6	12.5	96.1	4.5	80.5	12.5	14.3	7.6	50.8	38.5
			**DJ946 + 0 GAL80**											
DJ946	UAS-grim	1.1	96.0		0.0		0.0		100.0		0.0		0.0	
		2.1	92.0		0.0		0.0		88.0		0.0		0.0	
		3.1	84.0		0.0		0.0		100.0		0.0		0.0	
		4.1	88.0		0.0		0.0		88.0		0.0		0.0	
		1.2	64.0		0.0		0.0		72.0		0.0		0.0	
		2.2	60.0		0.0		0.0		88.0		0.0		0.0	
		3.2	72.0		0.0		0.0		76.0		0.0		0.0	
		4.2	68.0		0.0		0.0		76.0		0.0		0.0	
			78.0	13.7	0.0	0.0	0.0	0.0	86.0	10.7	0.0	0.0	0.0	0.0

Parents		Plate #.	%L1	SD	%P	SD	%A	SD	%L1	SD	%P	SD	%A	SD
Male	Female	Exp #	AMP						AMP/TET					
**0 GAL80**														
DJ946	w	1.1	92.0		82.7		94.8		96.0		91.7		100.0	
		2.1	96.0		75.0		100.0		96.0		75.0		94.5	
		3.1	88.0		81.9		100.0		88.0		86.4		89.5	
		4.1	88.0		95.5		100.0		88.0		86.4		94.8	
		1.2	88.0		63.7		100.0		80.0		65.0		100.0	
		2.2	92.0		82.7		100.0		96.0		100.0		95.9	
		3.2	92.0		78.3		94.5		88.0		91.0		100.0	
		4.2	80.0		80.0		100.0		100.0		76.0		100.0	
			89.5	4.8	80.0	8.9	98.7	2.5	91.5	6.6	83.9	11.2	96.8	3.9
**DJ946 + DJ1077**														
DJ946	UAS-grim;DJ1077	1.1	68.0		0.0		0.0		84.0		0.0		0.0	
		2.1	72.0		0.0		0.0		84.0		0.0		0.0	
		3.1	60.0		0.0		0.0		72.0		0.0		0.0	
		4.1	72.0		0.0		0.0		64.0		0.0		0.0	
			68.0	5.7	0.0	0.0	0.0	0.0	76.0	9.8	0.0	0.0	0.0	0.0
**DJ946 + 3.3**														
DJ946	UAS-grim;3.3	1.1	56.0		0.0		0.0		72.0		0.0		0.0	
		2.1	72.0		0.0		0.0		80.0		0.0		0.0	
		3.1	56.0		0.0		0.0		92.0		0.0		0.0	
		4.1	48.0		0.0		0.0		76.0		0.0		0.0	
			58.0	10.1	0.0	0.0	0.0	0.0	80.0	8.6	0.0	0.0	0.0	0.0
**DJ946 + 1X146 + 1X147**														
DJ946	UAS-grim;2.1+DJ1077	1.1	72.0		38.9		0.0		80.0		0.0		0.0	
		2.1	76.0		42.2		0.0		68.0		0.0		0.0	
		3.1	60.0		46.7		0.0		68.0		0.0		0.0	
		4.1	68.0		41.2		0.0		80.0		0.0		0.0	
DJ946	UAS-grim;2.1+DJ1079	1.2	64.0		56.3		88.9		64.0		0.0		0.0	
		2.2	44.0		63.7		57.2		68.0		0.0		0.0	
		3.2	56.0		57.2		62.5		36.0		0.0		0.0	
		4.2	80.0		50.0		70.0		36.0		0.0		0.0	
			65.0	11.7	49.5	8.9	34.8	38.3	62.5	17.4	0.0	0.0	0.0	0.0
**DJ1007 + 0 GAL80**														
DJ1007	UAS-grim	1.1	0.0		0.0		0.0		0.0		0.0		0.0	
		2.1	0.0		0.0		0.0		0.0		0.0		0.0	
		3.1	0.0		0.0		0.0		0.0		0.0		0.0	
		4.1	0.0		0.0		0.0		0.0		0.0		0.0	
			0.0	0.0	0.0	0.0	0.0	0.0	0.0	0.0	0.0	0.0	0.0	0.0
**0 GAL80**														
DJ1007	w	1.1	88.0		63.7		100.0		88.0		100.0		91.0	
		2.1	84.0		81.0		100.0		92.0		91.4		100.0	
		3.1	92.0		100.0		100.0		100.0		84.0		100.0	
		4.1	88.0		81.9		94.5		100.0		96.0		100.0	
			88.0	3.3	81.7	14.8	98.6	2.8	95.0	6.0	92.9	6.9	97.8	4.5
**DJ1007 + DJ1077**														
DJ1007	UAS-grim;DJ1077	1.1	48.0		0.0		0.0		72.0		0.0		0.0	
		2.1	68.0		0.0		0.0		64.0		0.0		0.0	
		3.1	60.0		0.0		0.0		64.0		0.0		0.0	
		4.1	80.0		0.0		0.0		68.0		0.0		0.0	
			64.0	13.5	0.0	0.0	0.0	0.0	67.0	3.8	0.0	0.0	0.0	0.0
**DJ1007 + 3.3**														
DJ1007	UAS-grim;3.3	1.1	0.0		0.0		0.0		0.0		0.0		0.0	
		2.1	0.0		0.0		0.0		0.0		0.0		0.0	
		3.1	0.0		0.0		0.0		0.0		0.0		0.0	
		4.1	0.0		0.0		0.0		0.0		0.0		0.0	
			0.0	0.0	0.0	0.0	0.0	0.0	0.0	0.0	0.0	0.0	0.0	0.0
**DJ1007 + 1X146 + 1X147**														
DJ1007	UAS-grim;2.1+DJ1077	1.1	72.0		38.9		0.0		60.0		6.7		0.0	
		2.1	60.0		26.7		0.0		68.0		0.0		0.0	
		3.1	72.0		27.8		0.0		68.0		5.9		0.0	
		4.1	60.0		40.0		0.0		56.0		7.2		0.0	
DJ1007	UAS-grim;2.1+DJ1079	1.2	52.0		15.4		0.0		80.0		0.0		0.0	
		2.2	40.0		20.0		0.0		68.0		0.0		0.0	
		3.2	60.0		33.4		0.0		68.0		0.0		0.0	
		4.2	60.0		33.4		0.0		56.0		0.0		0.0	
			59.5	10.4	29.5	8.7	0.0	0.0	65.5	8.0	2.5	3.4	0.0	0.0

Parents		Plate #.	%L1	SD	%P	SD	%A	SD	%L1	SD	%P	SD	%A	SD
Male	Female	Exp #	AMP						AMP/TET					
			**DJ1027 + 0 GAL80**											
		1.1	80.0		45.0		100.0		52.0		69.3		100.0	
DJ1027	UAS-grim	2.1	92.0		43.5		100.0		48.0		66.7		87.5	
		3.1	88.0		36.4		100.0		48.0		75.0		88.9	
		4.1	76.0		42.2		100.0		40.0		80.0		100.0	
			84.0	7.3	41.8	3.8	100.0	0.0	47.0	5.0	72.8	5.9	94.1	6.8
			**0 GAL80**											
		1.1	96.0		100.0		100.0		92.0		100.0		100.0	
DJ1027	w	2.1	88.0		100.0		100.0		92.0		95.7		91.0	
		3.1	92.0		87.0		100.0		96.0		91.7		100.0	
		4.1	92.0		82.7		100.0		96.0		91.7		100.0	
			92.0	3.3	92.4	8.9	100.0	0.0	94.0	2.3	94.8	4.0	97.8	4.5
			**DJ1027 + DJ1077**											
		1.1	64.0		37.5		100.0		64.0		50.0		100.0	
DJ1027	UAS-grim;DJ1077	2.1	76.0		36.9		100.0		48.0		33.4		100.0	
		3.1	88.0		50.0		100.0		52.0		38.5		100.0	
		4.1	72.0		27.8		100.0		60.0		33.4		100.0	
			75.0	10.0	38.1	9.1	100.0	0.0	56.0	7.3	38.8	7.8	100.0	0.0
			**DJ1027 + 3.3**											
		1.1	76.0		21.1		75.0		40.0		60.0		100.0	
DJ1027	UAS-grim;3.3	2.1	68.0		41.2		100.0		64.0		75.0		91.7	
		3.1	80.0		35.0		100.0		44.0		91.0		100.0	
		4.1	84.0		23.9		100.0		68.0		64.8		91.0	
			77.0	6.8	30.3	9.4	93.8	12.5	54.0	14.0	72.7	13.7	95.7	5.0
			**DJ1027 + 1X146 + 1X147**											
		1.1	92.0		39.2		77.8		92.0		52.2		91.7	
DJ1027	UAS-grim;2.1+DJ1077	2.1	100.0		76.0		89.5		92.0		47.9		100.0	
		3.1	80.0		75.0		66.7		84.0		47.7		100.0	
		4.1	88.0		54.6		41.7		92.0		56.6		92.4	
		1.2	76.0		57.9		27.3		76.0		21.1		100.0	
DJ1027	UAS-grim;2.1+DJ1079	2.2	52.0		46.2		33.4		80.0		50.0		80.0	
		3.2	60.0		73.4		27.3		64.0		56.3		77.8	
		4.2	76.0		42.2		37.5		92.0		26.1		100.0	
			78.0	16.0	58.1	15.1	50.2	24.3	84.0	10.3	44.7	13.5	92.7	9.2
			**DJ1040 + 0 GAL80**											
		1.1	0.0		0.0		0.0		0.0		0.0		0.0	
DJ1040	UAS-grim	2.1	0.0		0.0		0.0		0.0		0.0		0.0	
		3.1	0.0		0.0		0.0		0.0		0.0		0.0	
		4.1	0.0		0.0		0.0		0.0		0.0		0.0	
			0.0	0.0	0.0	0.0	0.0	0.0	0.0	0.0	0.0	0.0	0.0	0.0
			**0 GAL80**											
		1.1	88.0		77.3		100.0		96.0		95.9		100.0	
DJ1040	w	2.1	96.0		91.7		95.5		96.0		95.9		95.7	
		3.1	92.0		95.7		100.0		92.0		87.0		100.0	
		4.1	88.0		86.4		100.0		96.0		75.0		94.5	
			91.0	3.8	87.8	8.0	98.9	2.3	95.0	2.0	88.5	9.9	97.6	2.9
			**DJ1040 + DJ1077**											
		1.1	48.0		0.0		0.0		76.0		0.0		0.0	
DJ1040	UAS-grim;DJ1077	2.1	48.0		0.0		0.0		68.0		0.0		0.0	
		3.1	72.0		0.0		0.0		52.0		0.0		0.0	
		4.1	60.0		0.0		0.0		40.0		0.0		0.0	
			57.0	11.5	0.0	0.0	0.0	0.0	59.0	16.1	0.0	0.0	0.0	0.0
			**DJ1040 + 3.3**											
		1.1	0.0		0.0		0.0		0.0		0.0		0.0	
DJ1040	UAS-grim;3.3	2.1	0.0		0.0		0.0		0.0		0.0		0.0	
		3.1	0.0		0.0		0.0		0.0		0.0		0.0	
		4.1	0.0		0.0		0.0		0.0		0.0		0.0	
			0.0	0.0	0.0	0.0	0.0	0.0	0.0	0.0	0.0	0.0	0.0	0.0

Parents		Plate #.	%L1	SD	%P	SD	%A	SD	%L1	SD	%P	SD	%A	SD
Male	Female	Exp #	AMP						AMP/TET					
			DJ1040 + 1X146 + 1X147											
		1.1	72.0		61.2		0.0		84.0		4.8		0.0	
DJ1040	UAS-grim;2.1+DJ1077	2.1	84.0		38.1		0.0		80.0		0.0		0.0	
		3.1	80.0		50.0		0.0		76.0		0.0		0.0	
		4.1	80.0		50.0		0.0		72.0		0.0		0.0	
		1.2	56.0		92.9		0.0		64.0		0.0		0.0	
DJ1040	UAS-grim;2.1+DJ1079	2.2	64.0		62.5		0.0		60.0		13.4		0.0	
		3.2	68.0		41.2		0.0		60.0		13.4		0.0	
		4.2	64.0		43.8		0.0		48.0		8.4		0.0	
			71.0	9.7	55.0	17.7	0.0	0.0	68.0	12.1	5.0	6.0	0.0	0.0
			DJ1072 + 0 GAL80											
		1.1	96.0		87.5		95.3		88.0		91.0		100.0	
DJ1072	UAS-grim	2.1	84.0		85.8		94.5		88.0		91.0		100.0	
		3.1	96.0		91.7		100.0		84.0		90.5		94.8	
		4.1	92.0		91.4		100.0		84.0		90.5		100.0	
			92.0	5.7	89.1	2.9	97.5	3.0	86.0	2.3	90.8	0.3	98.7	2.6
			0 GAL80											
		1.1	96.0		91.7		100.0		96.0		95.9		100.0	
DJ1072	w	2.1	96.0		79.2		100.0		100.0		92.0		100.0	
		3.1	96.0		75.0		100.0		96.0		95.9		95.7	
		4.1	92.0		100.0		100.0		92.0		100.0		100.0	
			95.0	2.0	86.5	11.5	100.0	0.0	96.0	3.3	96.0	3.3	98.9	2.2
			DJ1072 + 3.3											
		1.1	88.0		72.8		100.0		88.0		100.0		95.5	
DJ1072	UAS-grim;3.3	2.1	92.0		65.3		100.0		88.0		100.0		77.3	
		3.1	92.0		69.6		100.0		80.0		100.0		80.0	
		4.1	92.0		82.7		100.0		80.0		100.0		85.0	
			91.0	2.0	72.6	7.4	100.0	0.0	84.0	4.6	100.0	0.0	84.5	8.0
			DJ817 + 0 GAL80											
		1.1	64.0		0.0		0.0		28.0		0.0		0.0	
DJ817	UAS-grim	2.1	52.0		0.0		0.0		36.0		0.0		0.0	
		3.1	48.0		0.0		0.0		36.0		0.0		0.0	
		4.1	40.0		0.0		0.0		44.0		0.0		0.0	
			51.0	10.0	0.0	0.0	0.0	0.0	36.0	6.5	0.0	0.0	0.0	0.0
			0 GAL80											
		1.1	100.0		84.0		100.0		92.0		87.0		95.0	
DJ817	w	2.1	96.0		100.0		100.0		92.0		100.0		100.0	
		3.1	96.0		95.9		100.0		100.0		88.0		100.0	
		4.1	92.0		82.7		100.0		88.0		91.0		100.0	
			96.0	3.3	90.7	8.6	100.0	0.0	93.0	5.0	91.5	5.9	98.8	2.5
			DJ817 + DJ1077											
		1.1	64.0		0.0		0.0		68.0		0.0		0.0	
DJ817	UAS-grim;DJ1077	2.1	68.0		0.0		0.0		76.0		0.0		0.0	
		3.1	56.0		0.0		0.0		88.0		0.0		0.0	
		4.1	60.0		0.0		0.0		80.0		0.0		0.0	
			62.0	5.2	0.0	0.0	0.0	0.0	78.0	8.3	0.0	0.0	0.0	0.0
			DJ817 + 3.3											
		1.1	64.0		0.0		0.0		72.0		0.0		0.0	
DJ817	UAS-grim;3.3	2.1	52.0		0.0		0.0		64.0		0.0		0.0	
		3.1	60.0		0.0		0.0		60.0		0.0		0.0	
		4.1	56.0		0.0		0.0		68.0		0.0		0.0	
			58.0	5.2	0.0	0.0	0.0	0.0	66.0	5.2	0.0	0.0	0.0	0.0
			DJ817 + 1X146 + 1X147											
		1.1	92.0		26.1		0.0		72.0		0.0		0.0	
DJ817	UAS-grim;2.1+DJ1077	2.1	80.0		25.0		0.0		68.0		0.0		0.0	
		3.1	80.0		35.0		0.0		60.0		0.0		0.0	
		4.1	72.0		44.5		0.0		48.0		0.0		0.0	
		1.2	56.0		21.5		0.0		48.0		0.0		0.0	
DJ817	UAS-grim;2.1+DJ1079	2.2	60.0		26.7		0.0		60.0		0.0		0.0	
		3.2	56.0		35.8		0.0		56.0		0.0		0.0	
		4.2	64.0		25.0		0.0		52.0		0.0		0.0	
			70.0	13.2	30.0	7.7	0.0	0.0	58.0	8.8	0.0	0.0	0.0	0.0

Parents		Plate #.	%L1	SD	%P	SD	%A	SD	%L1	SD	%P	SD	%A	SD
**Male**	**Female**	**Exp #**	**AMP**						**AMP/TET**					
			**GAL4.109 + 0 GAL80**											
GAL4.109	UAS-grim	1.1	84.0		0.0		0.0		100.0		0.0		0.0	
		2.1	92.0		0.0		0.0		100.0		0.0		0.0	
		3.1	88.0		0.0		0.0		84.0		0.0		0.0	
		4.1	88.0		0.0		0.0		96.0		0.0		0.0	
			88.0	3.3	0.0	0.0	0.0	0.0	95.0	7.6	0.0	0.0	0.0	0.0
			**0 GAL80**											
GAL4.109	w	1.1	96.0		91.7		100.0		88.0		100.0		100.0	
		2.1	84.0		95.3		100.0		92.0		100.0		100.0	
		3.1	96.0		91.7		100.0		96.0		91.7		100.0	
		4.1	92.0		91.4		100.0		96.0		95.9		100.0	
			92.0	5.7	92.5	1.9	100.0	0.0	93.0	3.8	96.9	4.0	100.0	0.0
			**GAL4.109 + DJ1077**											
GAL4.109	UAS-grim;DJ1077	1.1	84.0		0.0		0.0		68.0		0.0		0.0	
		2.1	84.0		0.0		0.0		80.0		0.0		0.0	
		3.1	76.0		0.0		0.0		88.0		0.0		0.0	
		4.1	84.0		0.0		0.0		84.0		0.0		0.0	
			82.0	4.0	0.0	0.0	0.0	0.0	80.0	8.6	0.0	0.0	0.0	0.0
			**GAL4.109 + 3.3**											
GAL4.109	UAS-grim;3.3	1.1	88.0		0.0		0.0		76.0		0.0		0.0	
		2.1	84.0		0.0		0.0		72.0		0.0		0.0	
		3.1	68.0		0.0		0.0		92.0		0.0		0.0	
		4.1	76.0		0.0		0.0		72.0		0.0		0.0	
			79.0	8.9	0.0	0.0	0.0	0.0	78.0	9.5	0.0	0.0	0.0	0.0
			**GAL4.109 + 1X146 + 1X147**											
GAL4.109	UAS-grim;2.1+DJ1077	1.1	68.0		0.0		0.0		76.0		0.0		0.0	
		2.1	88.0		0.0		0.0		80.0		0.0		0.0	
		3.1	76.0		10.6		0.0		84.0		0.0		0.0	
		4.1	76.0		0.0		0.0		76.0		0.0		0.0	
GAL4.109	UAS-grim;2.1+DJ1079	1.2	52.0		0.0		0.0		72.0		0.0		0.0	
		2.2	56.0		21.5		0.0		72.0		0.0		0.0	
		3.2	64.0		6.3		0.0		72.0		0.0		0.0	
		4.2	68.0		11.8		0.0		72.0		0.0		0.0	
			68.5	11.6	6.3	7.9	0.0	0.0	75.5	4.5	0.0	0.0	0.0	0.0

Reagents table

Data type	Experimental species	Symbol/name used in publication	Source – public	Source – published	Source – unpublished	Identifiers	New reagent	Comments
genetic reagent (in whole organism)	D. melanogaster	da-GAL4	Bloomington Drosophila Stock Center	PMID:7606787		BDSC:55851		
genetic reagent (in whole organism)	D. melanogaster	tub-GAL4		PMID:10197526		BDSC:5138		
genetic reagent (in whole organism)	D. melanogaster	DJ634	Bloomington Drosophila Stock Center	PMID:12882353		BDSC:8614		
genetic reagent (in whole organism)	D. melanogaster	D42	Bloomington Drosophila Stock Center			BDSC:8816		
genetic reagent (in whole organism)	D. melanogaster	24B	Bloomington Drosophila Stock Center			BDSC:1767		
genetic reagent (in whole organism)	D. melanogaster	ELAV	Bloomington Drosophila Stock Center			BDSC:8760		
genetic reagent (in whole organism)	D. melanogaster	DJ646	Bloomington Drosophila Stock Center	PMID:12882353		BDSC:8169		
genetic reagent (in whole organism)	D. melanogaster	DJ752	Bloomington Drosophila Stock Center	PMID:12882353		BDSC:8182		
genetic reagent (in whole organism)	D. melanogaster	DJ695	Bloomington Drosophila Stock Center	PMID:12882353		BDSC:8177		
genetic reagent (in whole organism)	D. melanogaster	DJ628	Bloomington Drosophila Stock Center	PMID:12882353		BDSC:8167		
genetic reagent (in whole organism)	D. melanogaster	actin	Bloomington Drosophila Stock Center	PMID:9043058		BDSC:3954		
genetic reagent (in whole organism)	D. melanogaster	DJ761	Bloomington Drosophila Stock Center	PMID:12882353		BDSC:8185		
genetic reagent (in whole organism)	D. melanogaster	DJ715	Bloomington Drosophila Stock Center	PMID:12882353		BDSC:8179		
genetic reagent (in whole organism)	D. melanogaster	DJ710		PMID:12882353				
genetic reagent (in whole organism)	D. melanogaster	DJ849		PMID:12882353				
genetic reagent (in whole organism)	D. melanogaster	cha-GAL4		PMID:15018821				
genetic reagent (in whole organism)	D. melanogaster	ddc-GAL4		PMID:10704417				
genetic reagent (in whole organism)	D. melanogaster	Nrv2-GAL4	Bloomington Drosophila Stock Center	PMID:10468627		BDSC:6799		
genetic reagent (in whole organism)	D. melanogaster	DJ755	Bloomington Drosophila Stock Center	PMID:12882353		BDSC:8183		
genetic reagent (in whole organism)	D. melanogaster	DJ756		PMID:12882353				
genetic reagent (in whole organism)	D. melanogaster	DJ785		PMID:12882353				
genetic reagent (in whole organism)	D. melanogaster	DJ946		PMID:12882353				
genetic reagent (in whole organism)	D. melanogaster	DJ1007		PMID:12882353				
genetic reagent (in whole organism)	D. melanogaster	DJ1027		PMID:12882353				
genetic reagent (in whole organism)	D. melanogaster	DJ1040		PMID:12882353				
genetic reagent (in whole organism)	D. melanogaster	DJ817		PMID:12882353				
genetic reagent (in whole organism)	D. melanogaster	GAL4.109		PMID:10521399				
genetic reagent (in whole organism)	D. melanogaster	UAS-lacZ	Bloomington Drosophila Stock Center			BDSC:1777		
genetic reagent (in whole organism)	D. melanogaster	UAS-GFP	Bloomington Drosophila Stock Center			BDSC:5137		
genetic reagent (in whole organism)	D. melanogaster	UAS-grim		PMID:9846179				
genetic reagent (in whole organism)	D. melanogaster	tetO-lacZ		PMID:9671025				
genetic reagent (in whole organism)	D. melanogaster	MHC Geneswitch		PMID:11675495				
genetic reagent (in whole organism)	D. melanogaster	DJ694		PMID:12882353		BDSC:8176		
recombinant DNA reagent	NA	pTub-GAL80		PMID:10197526				
recombinant DNA reagent	NA	pDJ136		this paper			Progenitors: PCR, pTub-GAL80	
recombinant DNA reagent	NA	pJY2000		PMID:11404007				
recombinant DNA reagent	NA	pDJ138		this paper			Progenitors: pDJ136; pJY2000	
recombinant DNA reagent	NA	pDJ139		this paper			Progenitors: pDJ136; linker	
recombinant DNA reagent	NA	pDJ140		this paper			Progenitors: pTub-GAL80; pDJ139	
recombinant DNA reagent	NA	pMS12		PMID:11404007				
recombinant DNA reagent	NA	pDJ143		this paper			Progenitors: pMS12; pDJ140	
recombinant DNA reagent	NA	pDJ146		this paper			Progenitors: pDJ143; pDJ138	
recombinant DNA reagent	NA	pDJ141		this paper			Progenitors: pMS12; pDJ139	
recombinant DNA reagent	NA	pDJ144		this paper			Progenitors: pMS12; pDJ140	
recombinant DNA reagent	NA	pDJ158		this paper			Progenitors: pDJ138	
recombinant DNA reagent	NA	pDJ159		this paper			Progenitors: pDJ158; adapter	
recombinant DNA reagent	NA	pDJ160		this paper			Progenitors: pDJ144; adapter	
recombinant DNA reagent	NA	pDJ147		this paper			Progenitors: pDJ160; pDJ159	
